# Risk prediction models for inadequate bowel preparation before colonoscopy: A systematic review and meta-analysis

**DOI:** 10.1371/journal.pone.0356009

**Published:** 2026-08-14

**Authors:** Hui-ling Yan, Jia-yu Fu, Mao-ting Huang, Wen-ting Yi, Jia-jun Liu, Xiao-yan Huang, Yao Fang, Ling Zhao, Yun-shan Chen, Ying Zeng

**Affiliations:** 1 School of Nursing, Hunan Engineering Research Center for Early Diagnosis and Treatment of Liver Cancer, Hunan Province Key Laboratory of Tumor Cellular & Molecular Pathology, Cancer Research Institute, Hengyang Medical School, University of South China, Hengyang, Hunan Province, China; 2 Department of Gastroenterology, The Second Affiliated Hospital, University of South China, Hengyang, Hunan Province, China; Dalin Tzu Chi Hospital, Buddhist Tzu Chi Medical Foundation, TAIWAN

## Abstract

**Background:**

Inadequate bowel preparation (IBP) can impair the safety, efficiency, and diagnostic accuracy of colonoscopy. Numerous multivariable prediction models have been developed to identify patients at high risk of IBP, yet their reported performance varies substantially.

**Objectives:**

To systematically evaluate the predictive performance and methodological quality of existing risk prediction models for IBP before colonoscopy.

**Methods:**

PubMed, Embase, Web of Science, the Cochrane Library, CINAHL, CNKI, Wanfang, and VIP were systematically searched from inception to December 16, 2024, and updated on December 16, 2025. Data extraction was guided by the Checklist for Critical Appraisal and Data Extraction for Systematic Reviews of Prediction Modelling Studies (CHARMS). Areas under the receiver operating characteristic curves (AUCs) with 95% confidence intervals from internal and external validations were pooled separately using random-effects models (Stata 18). Risk of bias and applicability were evaluated with PROBAST. The protocol was registered in PROSPERO (CRD42024627756).

**Results:**

Thirty-one studies reporting 46 prediction models were included, with sample sizes ranging from 152 to 23,456 participants and IBP event incidence rates of 6.3%–50.0%. Frequently used predictors included constipation, diabetes, age, body mass index (BMI), and history of colorectal surgery. Pooled AUCs were 0.76 (95% CI: 0.73–0.79) for internal validation and 0.72 (95% CI: 0.68–0.76) for external validation, both with substantial heterogeneity (I² > 90%). External validation was reported in ten studies, comprising 15 external validation cohorts, including four independent external validation studies of previously published models. Calibration was infrequently reported. Most studies were at high risk of bias, mainly in the analysis domain, while applicability concerns were generally low.

**Conclusions:**

Existing IBP prediction models show moderate to good discrimination on average, but pooled estimates are accompanied by substantial heterogeneity and widespread risk of bias, and robust external validation remains limited. Future studies should standardize outcome definitions and reporting and conduct large, multicenter, independent external validations to improve clinical utility.

## 1. Introduction

Colorectal cancer (CRC) remains a major global health concern, currently ranking third in incidence and second in cancer-related mortality worldwide [1]. Colonoscopy is widely regarded as the gold standard for CRC screening and prevention because it enables the detection and removal of precancerous lesions, including colorectal adenomas [2, 3]. However, the diagnostic accuracy and procedural safety largely depend on the quality of bowel preparation. Although international guidelines commonly recommend keeping the rate of inadequate bowel preparation (IBP) below 10%, IBP remains common in clinical practice, with reported incidence rates of 18%–35% even with split-dose regimens [4, 5]. IBP reduces adenoma detection rates, prolongs procedure time, increases patient discomfort, and frequently leads to repeat colonoscopies, thereby increasing healthcare utilization and patient burden [6–8].

Evidence suggests that enhanced patient education, optimized bowel preparation protocols, and reminder-based follow-up can reduce IBP by improving adherence and quality of preparation [9–11]. However, implementing intensive strategies for all patients is resource-demanding and may be inefficient, potentially increasing the burden on low-risk populations. In addition, real-world implementation may vary across clinicians and healthcare institutions. Moreover, commonly used bowel cleanliness scales, such as the Boston Bowel Preparation Scale (BBPS), assess achieved cleansing at colonoscopy and are not designed for pre-procedural risk prediction, thereby limiting opportunities for proactive optimization [4, 12]. Therefore, risk prediction tools before colonoscopy may help estimate an individual’s probability of developing IBP and support targeted counseling, preparation regimen selection, and follow-up. Clinical prediction models may provide a practical approach by combining readily available predictors to quantify IBP risk in advance and inform individualized preparation planning [13].

Despite the increasing number of IBP risk prediction models, the evidence base remains fragmented, and their clinical applicability remains uncertain. Existing models differ in outcome definitions, predictor selection, modeling methods, and performance reporting, which limits comparability across studies [14]. Furthermore, many models have been evaluated primarily in development cohorts or internal validations, with limited reports of independent external validation, which introduces uncertainty regarding their generalizability across settings and populations [14–16]. Importantly, previous reviews primarily summarized individual risk factors rather than quantitatively synthesizing and comparing model performance metrics, which limits their value in informing model selection and updating in clinical settings [17, 18].

Therefore, we conducted a comprehensive systematic review and meta-analysis of IBP risk prediction models before colonoscopy, synthesizing the evidence by summarizing model characteristics, quantitatively synthesizing predictive performance, and appraising methodological quality and reporting transparency to inform model selection and future improvements. The findings will provide valuable references for model selection, updating, and future model development.

## 2. Methods

This review was registered with PROSPERO (CRD42024627756). It was conducted in accordance with the PRISMA 2020 statement [19] and the TRIPOD-SRMA guidelines [20] to ensure methodological rigor and transparency in the systematic evaluation of prediction models.

### 2.1. Search strategy

A comprehensive literature search was conducted across eight databases: PubMed, Web of Science, Embase, Cochrane Library, CINAHL, CNKI, Wanfang, and VIP. The time frame spanned from the inception of each database to December 16, 2024, and was updated on December 16, 2025. A combination of MeSH and free-text terms was used to identify studies related to risk prediction models for IBP. Keywords included, but were not limited to, “Colonoscopy,” “Endoscopy,” “Bowel preparation,” “Bowel cleansing,” “Inadequate bowel preparation,” “Risk prediction model,” “Risk factors,” “Predictors,” “Model,” and “Risk score.” Reference lists of included articles and relevant systematic reviews were also manually screened to identify additional eligible studies. We did not impose any language restrictions on the literature searches. For potentially eligible non-English articles, full texts were translated using machine translation (e.g., DeepL/Google Translate) and checked by two reviewers. Any disagreements were resolved by consensus or consultation with a third reviewer when needed. The complete search strategies for each database are detailed in S1 Table.

### 2.2. Inclusion and exclusion criteria

To guide the formulation of research questions and ensure consistency in eligibility criteria, we adopted the PICOTS framework recommended by the Checklist for Critical Appraisal and Data Extraction for Systematic Reviews of Prediction Modelling Studies (CHARMS) [21].

Studies were included if they met the following criteria: (1) P (Population): Adult patients (≥18 years) undergoing colonoscopy, including both outpatients and inpatients; (2) I (Index model): Studies that developed and/or validated multivariable prediction models for identifying the risk of IBP, regardless of modeling method (e.g., logistic regression, machine learning); (3) C (Comparator): No comparator models were required, as this review did not aim to compare models; (4) O (Outcome): The outcome was IBP before colonoscopy, as defined by the original study using a validated scale (e.g., Boston Bowel Preparation Scale (BBPS), Aronchick Scale); (5) T (Timing): Eligible models must have been constructed using information available before the colonoscopy; studies using intra- or post-procedural predictors were excluded; (6) S (Setting): Studies conducted in secondary or tertiary care settings involving patients undergoing colonoscopy in either outpatient or inpatient contexts; (7) Study design: Observational studies (e.g., cross-sectional, cohort, case-control) that reported model performance metrics such as AUC. We excluded studies based on the following exclusion criteria: (1) Investigated risk factors for IBP without developing or validating a prediction model; (2) Predictor variables < 2; (3) Full text was not available; (4) Duplicate publications.

### 2.3. Study selection and screening

All references were managed using EndNote X9. Two reviewers independently screened the studies in a standardized, stepwise process. Duplicates were removed first. The remaining records were then assessed based on titles and abstracts to identify potentially eligible studies. Full texts were subsequently retrieved and reviewed against the predefined inclusion and exclusion criteria for final eligibility determination. To enhance comprehensiveness, the reference lists of all included studies and relevant systematic reviews were manually searched to identify additional studies that may have been missed during the initial database search. Any discrepancies in study selection were resolved through discussion with the third author until consensus was reached.

### 2.4. Data extraction

A standardized data extraction form was developed based on the CHARMS checklist [21]. Extracted information was categorized into two domains: (1) Study characteristics include: first author, year of publication, country, study design, sample size, source of data, participants, outcome assessment tool, IBP prevalence, and bowel preparation regimen. (2) Model characteristics include: methodological details such as handling of continuous variables, treatment of missing data, predictor selection methods, model development, model evaluation, model performance, final predictors included in the model, and model presentation format. Data extraction was performed by one reviewer and independently cross-checked by a second reviewer to ensure accuracy and consistency. Any disagreements were resolved by a third reviewer. For studies with missing or incomplete data, corresponding authors were contacted via email to obtain additional information where possible.

### 2.5. Risk of bias and certainty of evidence assessment

Two reviewers independently assessed the risk of bias, applicability, and certainty of evidence using the Prediction model Risk Of Bias Assessment Tool (PROBAST) [22, 23] and the GRADE framework [24]. Discrepancies were resolved through discussion with a third reviewer, who made the final judgment when necessary. The PROBAST tool evaluates risk of bias and applicability across four domains: participants, predictors, outcome, and analysis, based on 20 signaling questions. If any domain was rated as “No” or “Probably No,” the study was considered at high risk of bias. If one or more domains were rated as “Unclear” and all other domains were at low risk, the overall risk was judged as unclear. Only when all domains were rated as low risk was the overall study judged to have a low risk of bias. Applicability was assessed using PROBAST across three domains, including participants, predictors, and outcome. Each domain was rated as low concern, high concern, or unclear concern. Overall applicability followed a worst domain rule. Overall applicability was rated as high concern if any domain was rated high concern. Overall applicability was rated as unclear concern when no domain was high concern and at least one domain was unclear concern. Overall applicability was rated as low concern only when all three domains were rated low concern.

The certainty of evidence for pooled AUC values was assessed using the GRADE approach [24, 25], considering five domains: risk of bias, inconsistency, indirectness, imprecision, and publication bias. The overall certainty of the evidence was classified as high, moderate, low, or very low.

### 2.6. Data synthesis and statistical analysis

All statistical analyses were conducted in Stata version 18. Study and model characteristics were summarized descriptively. Discrimination was assessed using the area under the receiver operating characteristic curve (AUC). We meta-analyzed AUCs separately for results derived from internal validation and external validation to summarize the overall discriminative performance of current IBP prediction models. For internal validation, each eligible model contributed one AUC estimate. For external validation, each distinct validation cohort contributed one effect estimate. When the same model had been externally validated across multiple cohorts/studies, we additionally synthesized those AUCs to summarize model-specific transportability. Before pooling, AUCs were transformed using the logit function and pooled on the logit scale; summary estimates were back-transformed to the original AUC scale for interpretation. Random-effects meta-analyses were fitted using restricted maximum likelihood (REML) to estimate between-study variance, and 95% confidence intervals were calculated using the Hartung–Knapp–Sidik–Jonkman (HKSJ) method to provide more robust inference under heterogeneity [26]. Calibration was not meta-analyzed due to insufficient reporting and was summarized narratively. When quantitative synthesis was not appropriate (e.g., too few estimates or substantial inconsistency), results were presented descriptively.

Heterogeneity was assessed using Cochran’s Q test and the I^2^ statistic [26]. Publication bias was evaluated using funnel plots and Egger’s regression test for pooled AUCs, in line with GRADE requirements. In accordance with Cochrane guidance, formal publication bias tests were not performed when fewer than 10 studies were included [27]. Sensitivity analyses were conducted using a leave-one-out approach to examine the robustness of the pooled estimates and identify potential influential studies [28]. In cases of substantial heterogeneity (I^2^ > 50%), subgroup analyses and meta-regression were performed to explore potential sources, with key covariates including country, participants, study design, and other relevant study-level characteristics. Statistical significance was defined as *P* < 0.05.

## 3. Results

### 3.1. Study selection

**Fig 1:**
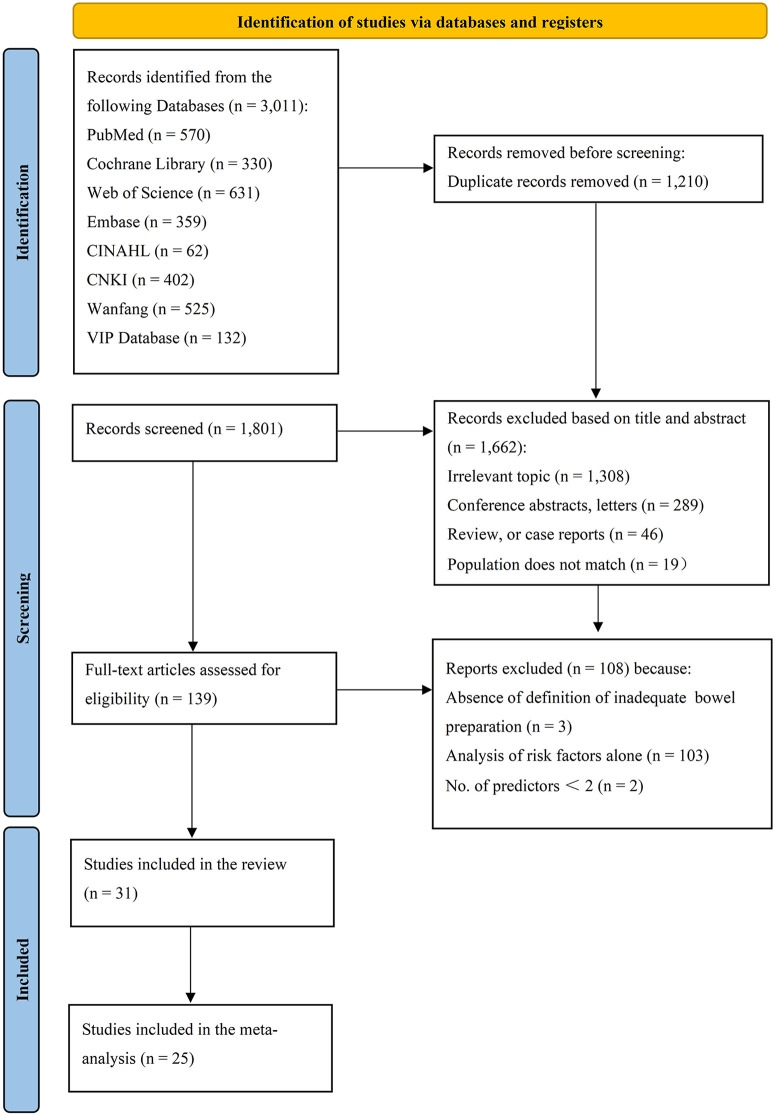
PRISMA flow diagram for the selection of studies.

Fig 1 illustrates the study selection process. A total of 3,011 records were initially retrieved. After removing 1,210 duplicates using EndNote X9, 1,662 records were excluded based on title and abstract screening because they were review articles, irrelevant to the study topic, or otherwise ineligible. The full texts of 139 articles were assessed for eligibility, with 108 studies excluded for reasons including lack of model development, unclear definitions of IBP, or fewer than two predictors. Ultimately, 31 studies were included. Among the included studies, 25 provided sufficient information for inclusion in the meta-analysis.

### 3.2. Study characteristics

Table 1 summarizes the key characteristics of the 31 included studies, covering country, study design, data sources, population, and outcome definition. The studies were published between 2012 and 2025, with 91% published since 2020. The included studies were conducted across 11 countries, with the majority carried out in China (n = 20). Most studies adopted a prospective design (n = 16), followed by retrospective (n = 13) and case–control (n = 2) studies. Data were mainly derived from single-center cohorts (n = 17), with 14 multicenter studies. Several studies focused specifically on outpatient populations (n = 7), inpatients (n = 4), while the rest included general adult colonoscopy populations. The reported proportion of IBP varied widely, ranging from 6.25% to 50%, with sample sizes spanning from 152 to 23,456. IBP was most commonly defined using the Boston Bowel Preparation Scale, with inadequate preparation typically defined as BBPS < 6 and/or any segment score < 2. One study used a modified Aronchick scale, and another used a predefined four-level scale.

**Table 1:** Overview of the basic data of the included studies.

Author (year)	Country	Study design	Study population	Data source	Outcome Measurement	Outcome definition	IBP case/Sample size(%)
Berger 2021 [29]	France	Prospective cohort study	Adult patients with an indication for colonoscopy	Single center (clinical data from a teaching hospital)	BBPS	IBP: BBPS score < 7 or a score ≤ 1 in any segments	140/561(25%)
Chen 2022 [30]	China	Prospective cohort study	Patients aged 18–80 years who received a split-dose regimen of PEG for BP	Multicenter (clinical data from five endoscopic centers; external validation cohort from 12 endoscopic centers)	BBPS	IBP: BBPS score < 6 or any segment score < 2	137/906(15.1%)
Dik 2015 [16]	Netherlands	Prospective cohort study	Adult patients undergoing colonoscopy with split-dose bowel preparation	Multicenter (clinical records from four hospitals)	BBPS	IBP: BBPS score < 6	249/1996(12.5%)
Fuccio 2021 [31]	Italy	Prospective cohort study	Adult inpatients scheduled for a colonoscopy for any indication	Multicenter (clinical data from twelve hospitals)	BBPS	IBP: BBPS score < 2 in any segment	492/1524(32.3%)
Gu 2024 [32]	China	Prospective cohort study	Adult outpatients scheduled for screening colonoscopies	Multicenter (clinical data from four tertiary hospitals)	BBPS	IBP: BBPS score < 6 or any segment score < 2	688/3217(21.14%)
Gimeno-García 2017 [33]	Spain	Prospective cohort study	Patients who underwent an outpatient colonoscopy	Single center (clinical data from a tertiary hospital)	BBPS	IBP: BBPS score < 2 in any segment	200/1076(18.6%)
Hassan 2012 [15]	Italy	Prospective cohort study	Patients who underwent colonoscopy examinations (19–99 years old)	Multicenter (clinical data from 18 endoscopy centers)	The predefined scale*	IBP: Using the predefined scale to rate the preparation level as fair or poor	925/2811(33%)
Gkolfakis 2023 [34]	Greece	Retrospective cohort study	Inpatients undergoing colonoscopy	Multicenter (clinical data from four tertiary hospitals)	BBPS	IBP: BBPS score < 6 or any segment score < 2	89/261(34.1%)
Malkin 2023 [35]	Israel	Prospective cohort study	All patients who underwent elective colonoscopy	Single center (clinical data from a tertiary medical center)	BBPS	IBP: BBPS score < 6	77/324(23.7%)
Okamoto 2022 [36]	Japan	Prospective cohort study	Patients who required a colonoscopy (≥20 years old)	Multicenter(clinical data from a hospital and a clinic)	BBPS	IBP: BBPS score < 6 or any segment score < 2	55/350(15.7%)
Sninsky 2024 [37]	USA	Retrospective cohort study	Adult patients who had an outpatient colonoscopy	Single center (electronic medical records from a hospital)	Modified Aronchick scale	IBP: Modified Aronchick scale (inadequate/fair/poor vs. good/excellent)	1466/23456(6.25%)
Zhang 2024 [38]	China	Prospective cohort study	Adult outpatients who underwent colonoscopy	Single center (clinical data from a tertiary hospital)	BBPS	IBP: BBPS score < 6 or any segment score < 2	270/1115(24.2%)
Zhao 2024 [39]	China	Prospective cohort study	Adult outpatients scheduled for colonoscopy	Multicenter (clinical data from three hospitals)	BBPS	IBP: BBPS score < 2 in any colonic segment	276/1180(23.4%)
Liu 2024 [40]^a^	China	Prospective cohort study	Adult patients (≥20 years old)	Single center (clinical data from a tertiary hospital)	BBPS	IBP: BBPS score < 6 or any segment score < 2	150/781(19.2%)
Huang 2023 [41]^a^	China	Retrospective cohort study	Adult outpatients (18–85 years old) who underwent colonoscopy	Single center (clinical data from a tertiary hospital)	BBPS	IBP: BBPS score < 6 or any segment score < 2	421/1349(31.21%)
Li 2020 [42]^a^	China	Case-control study	Outpatients and inpatients who underwent colonoscopy(18–80 years old)	Single center (clinical data from a tertiary hospital)	BBPS	IBP: BBPS score ≤ 6	195/455(42.8%)
Rao 2021 [43]^a^	China	Prospective cohort study	Adult patients hospitalized for a colonoscopy	Single center (clinical data from a tertiary hospital)	BBPS	IBP: BBPS score ≤ 5 or any segment score < 2	273/1200(22.75%)
Wang 2024 [44]^a^	China	Retrospective cohort study	Patients who underwent colonoscopy	Multicenter (clinical data from two tertiary hospitals)	BBPS	IBP: BBPS score ≤ 5	105/379(27.7%)
Guo 2023 [45]^a^	China	Prospective cohort study	Elderly patients	Single center (clinical data from a tertiary hospital)	BBPS	IBP: BBPS score < 6 or any segment score < 2	198/745(26.6%)
Wang 2024 [46]^a^	China	Retrospective cohort study	The elderly patients who underwent colonoscopy	Single center (clinical data from a tertiary hospital)	BBPS	IBP: BBPS score < 6 or any segment score < 2	146/700(20.9%)
Kutyla 2020 [47]	Australia	Case-control study	Adult outpatients	Single center (clinical data from a tertiary hospital endoscopy unit)	BBPS	IBP: BBPS score < 6	76/152(50%)
Zhao 2022 [48]^a^	China	Retrospective cohort study	Inpatients who underwent colonoscopy(18–80 years)	Single center (clinical data from one tertiary hospital)	BBPS	IBP: BBPS score < 6 or any segment score < 2	99/339(29.2%)
Song 2024 [49]^a^	China	Retrospective cohort study	Patients (over 20 years of age) who underwent colonoscopy	Single center (clinical data from hospitalized patients in a tertiary hospital)	BBPS	IBP: BBPS score < 6 or any segment score < 2	96/373(25.7%)
Afecto 2023 [50]	Portugal	Retrospective cohort study	Adult patients underwent colonoscopy in a tertiary hospital	Single center (clinical data from a tertiary hospital)	BBPS	IBP: BBPS score < 6 or any segment score < 2	73/514(14.2%)
Yuan 2022 [51]	China	Prospective cohort study	Adult patients underwent a non-sedative colonoscopy	Single center (clinical data from an endoscopy center of a tertiary hospital)	BBPS	IBP: BBPS score < 6 or any segment score < 2	110/461(23.9%)
Fang 2025 [52]	China	Retrospective cohort study	Outpatients aged ≥40 years old undergoing initial colonoscopy	Multicenter (electronic medical records from three endoscopic centers)	BBPS	IBP: BBPS score < 6 or any segment score < 2	2132/6860(31.08%)
Apaer 2024 [53]^a^	China	Retrospective cohort study	Adult inpatients	Single center (clinical data from an endoscopy center of a tertiary hospital)	BBPS	IBP: BBPS score < 6 or any segment score < 2	231/1221(18.9%)
Xu 2024 [54]^a^	China	Prospective cohort study	Elderly patients(over 60 years old)	Single center (clinical data from an endoscopy center of a tertiary hospital)	BBPS	IBP: BBPS score < 6 or any segment score < 2	174/610(28.5%)
Liu 2025 [55]	China	Retrospective cohort study	Elderly patients(over 60 years old)	Single center (clinical data from an endoscopy center of a tertiary hospital)	BBPS	IBP: BBPS score < 6 or any segment score < 2	109/471(23.14%)
Wu 2025 [56]	China	Prospective cohort study	Consecutive adult patients who underwent colonoscopy	Multicenter (clinical data from two tertiary hospitals)	BBPS	IBP: BBPS score < 6 or any segment score < 2	114/1165(9.8%)
Yin 2025 [57]	China	Prospective cohort study	Adult outpatients who were scheduled for a colonoscopy	Single center (clinical data from an endoscopy center of a tertiary hospital)	BBPS	IBP: BBPS score < 6 or any segment score < 2	330/1314(25.11%)

**Abbreviations:** BP, bowel preparation; IBP, inadequate bowel preparation; BBPS, Boston Bowel Preparation Scale; NR, not reported; **Note:** a: Studies which were published in Chinese; *: A predefined scale classiﬁed the level of preparation as follows: (1)excellent: no or minimal solid stool and only small amounts of clear ﬂuid requiring suctioning; (2)good: no or minimal solid stool with large amounts of clear ﬂuid requiring suctioning; (3)fair: collection of semisolid material that is cleared with difﬁculty; and (4)poor: solid or semisolid material that cannot be effectively cleared; BBPS: inadequate bowel preparation was defined as BBPS <6 or any segment score <2.

### 3.3. Model development and validation characteristics

Table 2 summarizes the characteristics of model development, validation, and performance evaluation. Most studies employed logistic regression for model construction, while a minority applied machine learning methods such as support vector machines (SVM), decision trees (DT), and extreme gradient boosting (XGBoost). All studies reported performance metrics, primarily the AUC, which ranged from 0.62 to 0.902. Calibration assessment was reported in 18 studies, most commonly using the Hosmer–Lemeshow test, whereas 13 studies did not report calibration assessment. Model presentation varied across studies, including scoring tools, nomograms, risk equations, and mobile applications. Regarding validation, among the 31 included studies, 19 reported both model development and internal validation, 6 conducted external validation following development, 2 focused solely on model development, and 4 were dedicated to external validation of existing models.

**Table 2:** Overview of the information on the included prediction models.

Author(year)	Continuous variable processing method	Missing data handling	Variable selection	Modeling Methods	Validation method	AUC/C-statistic (95% CI)	Calibration method	Model presentation
Afecto 2023 [50]	NR	NR	NR	NR	External validation only(Dik’s and Gimeno’s model)	Dik’s model: C:0.62(0.55–0.69); Gimeno’s model: C:0.62(0.55–0.70)	NR	NR
Chen 2022 [30]	Categorical variable	NR	Multivariate selection after univariate selection	LR	Internal validation(8:2 random split validation)/ External validation	Preparation-related model*: A:0.81(0.76–0.86), B:0.81, C:0.80(0.76–0.84); Patient-related model: A:0.64(0.58–0.70), B:0.68; Combined model: A:0.84(0.80–0.88), B:0.83	HL & Calibration curves	Risk score system
Huang 2023 [41]^a^	Continuous variable	NR	Backwards elimination	LR	Internal validation (random split validation)	A:0.7015, B:0.8386	HL	Nomogram model
Malkin 2023 [35]	Continuous variable	NR	Backward stepwise selection	LR	NR	A:0.737(0.673–0.8)	HL	The formula of the risk score
Xu 2022 [54]^a^	Categorical variable	NR	Backward stepwise selection	LR	Internal validation (random split validation)	A:0.717(0.660–0.773), B:0.720(0.645–0.796)	HL	Risk score system
Rao 2021 [43]^a^	Continuous variable	NR	Multivariate selection after univariate selection	LR	Internal validation (random split validation)	A:0.883(0.859–0.906), B:0.883(0.859–0.907)	HL	Nomogram model
Berger 2021 [29]	Continuous variable	NR	LASSO Logistic Regression	LR	Internal validation (bootstrap cross-validation)	A:0.622, B:0.621(0.558 − 0.689)	NR	Risk score system
Fuccio 2021 [31]	Continuous variable	NR	Backwards elimination	LR	Internal validation	A:0.78(0.74–0.81), B:0.73(0.69–0.78)	Calibration belt	A web-based application
Yuan 2022 [51]	NR	NR	NR	NR	External validation only (Dik’s and Gimeno’s model)	Dik ≥ 3 model: C:0.691(0.646–0.733); Dik ≥ 2 model: C:0.660(0.604–0.717); Gimeno>1.225 model: C:0.645(0.615–0.704)	NR	NR
Guo 2023 [45]^a^	Categorical variable	NR	Multivariate selection after univariate selection	LR	Internal validation (10-fold cross validation)/External validation	A:0.806(0.760–0.852), B:0.785(0.695–0.875), C:0.824(0.760–0.887)	HL	Risk score system
Gkolfakis 2023 [34]	NR	NR	NR	NR	External validation (Fuccio’s, Dik’s, and Gimeno’s model)	Fuccio’s model: C:0.772(0.752–0.788); Dik’s model: C:0.726(0.705–0.742); Gimeno’s model: C:0.575(0.545–0.602)	NR	NR
Okamoto 2022 [36]	Continuous variable	NR	Multivariate selection after univariate selection	LR	Internal validation(bootstrap cross-validation)	A:0.75, B:0.75(0.68–0.82)	Brier score	The formula of the risk score
Hassan 2012 [15]	Continuous variable	NR	Multivariate selection after univariate selection	LR	Internal validation	A: NR, B:0.63(0.62–0.66)	NR	The formula of the risk score
Wang 2024 [46]^a^	Categorical variable	NR	Multivariate selection after univariate selection	LR	Internal validation	A:0.720, B:0.726	HL	Risk score system
Dik 2015 [16]	Categorical variable	NR	Backwards elimination	LR	Internal validation	A:0.72(0.68–0.76), B:0.77(0.71–0.83)	NR	The formula of the risk score
Li 2020 [42]^a^	Categorical variable	NR	Forward stepwise regression & best subset selection	LR	Internal validation	Basic model: B:0.867(0.811–0.922); Optimal subset model*: B:0.873(0.819–0.926)	NR	Nomogram model
Zhao 2022 [48]^a^	Continuous variable	NR	Multivariate selection after univariate selection	LR	Internal validation	A:0.866(0.821–0.911), B:0.770(0.671–0.869)	HL & Calibration curves	Nomogram model
Kutyla 2020 [47]	Continuous variable	NR	Step-wise logistic regression	LR	NR	Full logistic model: AUC = 0.86; B-PriSM model*: AUC = 0.74	NR	B-PriSM tool
Sninsky 2024 [37]	Continuous variable	Median imputation	Multivariate selection after univariate selection	1. LR; 2. LASSO Regression & The reduced LASSO Regression; 3. GBM	Internal validation	1. A:0.64(0.62–0.66); 2. Model 1: A:0.65(0.63–0.67), B:0.66(0.64–0.68) Model 2*: A:0.64(0.62–0.66), B:0.65(0.63–0.67); 3. A:0.65(0.63–0.66), B:0.65(0.63–0.68);	Calibration plot	Fast Healthcare Interoperability Resources (FHIR)APP
Gu 2024 [32]	Continuous variable	NR	Multivariate selection after univariate selection	1. LR; 2. SVM; 3. DT; 4. XGB; 5. BPNet	Internal validation	1. A:0.65(0.62–0.67), B:0.62(0.55–0.66); 2. A:0.84(0.79–0.89), B:0.76(0.71–0.81); 3*. A:0.82(0.81–0.83), B:0.80(0.78–0.82); 4. A:0.77(0.75–0.79), B:0.77(0.72–0.82); 5. A:0.81(0.79–0.83), B:0.71(0.70–0.72)	NR	Decision tree visualization diagram
Zhang 2024 [38]	Categorical variable	NR	Backward stepwise selection	LR	Internal validation	A:0.732(0.687–0.776), B:0.713(0.658–0.769)	HL & Calibration plot	Nomogram model
Song 2024 [49]^a^	Categorical variable	NR	Multivariate selection after univariate selection	LR	Internal validation	A:0.7455, B:0.7709	NR	Nomogram model
Gimeno-García 2017 [33]	Continuous variable	NR	Multivariate selection after univariate selection	LR	Internal validation	A:0.72, B:0.70(0.65–0.74)	NR	The formula of the risk score
Liu 2024 [40]^a^	Categorical variable	NR	Forward stepwise regression	LR	Internal validation (bootstrap cross-validation)/External validation	A:0.751(0.696–0.805), B:0.865(0.815–0.915), C:0.775(0.683–0.867)	HL & Calibration plot	Nomogram model & The formula of risk score
Wang 2024 [44]^a^	Continuous variable	NR	LASSO Logistic Regression	1. LASSO Regression; 2. GBM; 3. DL; 4. Stacked Ensemble 5. DRF	Internal validation(7:3 random split validation)	1. A:0.881, B:0.734; 2. B:0.865; 3. B:0.868; 4. Model 1* B:0.871; Model 2 B: 0.860; 5. NR	Calibration curves	Nomogram model
Zhao 2024 [39]	Continuous variable	NR	Multivariate selection after univariate selection	LR	Internal validation(7:3 random split validation)/External validation	A:0.818(0.781–0.856), B:0.747(0.668–0.827), C:0.728(0.624–0.814)	HL & Calibration curves	Nomogram model & The formula of the risk score
Apaer 2024 [53]^a^	Continuous variable	NR	NR	NR	External validation only(Rao’s model)	C:0.900(0.762–0.902)	NR	Nomogram model
Fang 2025 [52]	Continuous variable	NR	Multivariate selection after univariate selection	LR	Internal validation(6:4 random split validation)	Nomogram prediction model: A: 0.740 (0.723–0.756), B: 0.730 (0.709–0.751); Derived risk-scoring tool*: A: 0.791 (0.774–0.807), B: 0.750 (0.730–0.770)	HL & Calibration curves	Nomogram model & The formula of the risk score
Wu 2025 [56]	Categorical variable	Complete-case analysis	Multivariate selection after univariate selection	LR	NR/External validation	A:0.778(0.719–0.837), B:NR, C:0.774(0.685–0.865)	Calibration curve	Nomogram & Risk score system
Yin 2025 [57]	Categorical variable	NR	Multivariate selection after univariate selection	LR	Internal validation (bootstrap cross-validation)/External validation	A: 0.704(0.667–0.741), B: NR, C: 0.704(0.628–0.779)	Calibration curve	Risk score system
Liu 2025 [55]	Categorical variable	NR	Boruta algorithm	1. LightGBM; 2. SVM; 3. DT; 4. RF; 5. XGBoost	Internal validation(7:3 random split validation)/External validation	1. A:0.945(0.914–0.976), B:0.863(0.772–0.955); 2*. A:0.905(0.858–0.953), B:0.895(0.822–0.969); 3. A:0.968(0.948–0.988), B:0.791(0.686–0.896); 4. A:0.958(0.933–0.983), B:0.877(0.795–0.960); 5. A:0.954(0.927–0.982), B:0.879(0.794–0.963)	NR	A web application

**Abbreviations:** NR, Not reported; LR, Logistic regression; IBP, Inadequate bowel preparation; LASSO Regression, Least absolute shrinkage and selection operator logistic regression; SVM, Support vector machines; DT, Decision trees; XGB, Extreme gradient boosting; BPNet, Bidirectional projection network; GBM, Gradient boosting machine; DL, Deep learning; GLM, Generalized linear model; DRF, Distributed random forest; BMI, Body mass index; HL, Hosmer–Lemeshow Test; AUC, Area under the curve; C-statistic, concordance statistic; CI, confidence interval. **Note:** A: Development cohort; B: Internal validation; C: External validation; *: The optimal model selected by the authors through comparison.

**Fig 2:**
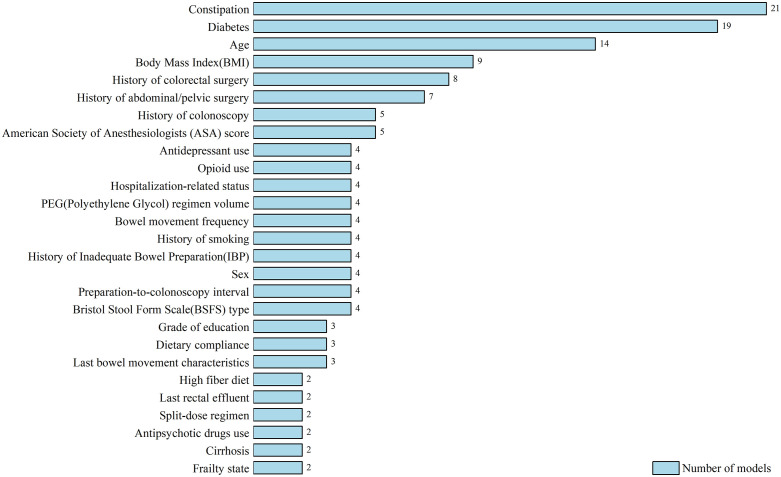
Summary of final predictors included in the prediction model studies.

Across models, the number of final predictors ranged from 3 to 14, which were broadly grouped into patient characteristics, bowel preparation-related factors, and comorbidities. The most frequently included predictors were constipation (n = 21), diabetes (n = 19), age (n = 14), body mass index (BMI) (n = 9), and history of colorectal surgery (n = 8). Detailed information is presented in S2 Table and Fig 2.

### 3.4. Meta-analysis results

Overall, 31 studies were included in the systematic review, and 25 contributed data to the meta-analysis. Studies were excluded from the meta-analysis if they reported model development without validation, or if the information required to derive the standard error of the AUC was unavailable. Ultimately, a total of 17 studies based on internal validation datasets (31 models) and 10 studies based on external validation datasets (15 models) were included in the meta-analysis. Some studies contributed to both internal- and external-validation syntheses. Meta-analyses were conducted separately for internal- and external-validation results.

#### 3.4.1. Models with internal validation cohorts.

**Fig 3:**
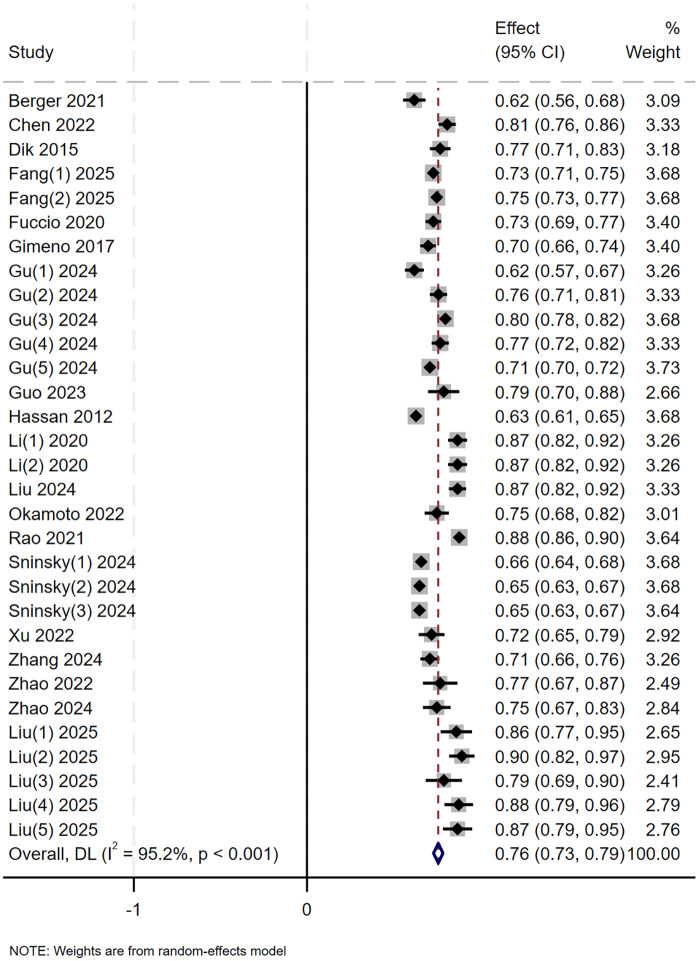
Forest plot of pooled AUCs for internal validation (random-effects).

Internally validated model results were pooled using a random-effects meta-analysis, which yielded a pooled AUC of 0.76 (95% CI, 0.73–0.79) (Fig 3), with substantial heterogeneity (*I²* = 95.2%, *P* < 0.001). Prespecified subgroup analyses were conducted to explore potential sources of heterogeneity (S3 Table).

By modeling method, machine learning-based models (n = 13) had a pooled AUC of 0.75 (95% CI, 0.71–0.79; *I²* = 95.0%), whereas non-machine learning models (n = 18; predominantly logistic regression) had a pooled AUC of 0.76 (95% CI, 0.72–0.80; *I²* = 95.0%). No evidence of subgroup differences was observed by the modeling method (*P* for interaction = 0.745). Discrimination varied by study design (*P* for interaction < 0.001). Prospective cohort studies (n = 23) yielded a pooled AUC of 0.77 (95% CI, 0.73–0.80), compared with 0.70 (95% CI, 0.65–0.74) for retrospective cohort studies (n = 6). Case-control studies reported a higher pooled AUC of 0.87 (95% CI, 0.83–0.91), but this estimate was based on a small number of models (n = 2) and should be interpreted cautiously. When stratified by predictor composition, models using only patient-related predictors yielded a pooled AUC of 0.73 (95% CI, 0.69–0.77), whereas models combining patient- and preparation-related predictors yielded a pooled AUC of 0.77 (95% CI, 0.74–0.81). Subgroup differences by predictor composition were statistically significant (*P* for interaction = 0.039). The preparation-only category was represented by a single model and was therefore not informative for between-subgroup inference.

In contrast, subgroup differences were not significant by participant type (*P* = 0.439) or center type (*P* = 0.158). Sample size showed a borderline subgroup effect (*P* = 0.061), with higher pooled AUCs in models with ≥500 participants. Notably, heterogeneity remained substantial within most subgroups, indicating that multiple factors likely contribute to variability in internally validated discrimination.

#### 3.4.2. Models with external validation cohorts.

**Fig 4:**
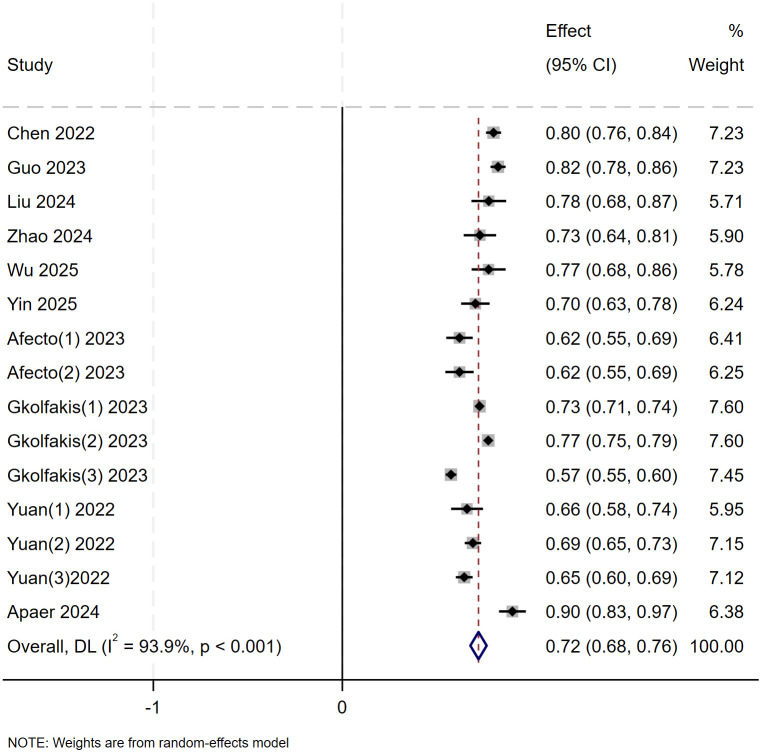
Forest plot of pooled AUCs for external validation (random-effects).

Using a random-effects meta-analysis, externally validated results across 15 validation cohorts yielded a pooled AUC of 0.72 (95% CI, 0.68–0.76) (Fig 4). Substantial heterogeneity was observed (I² = 93.9%, *P* < 0.001), and externally validated AUCs varied widely across cohorts, ranging from approximately 0.57 to 0.902. These findings indicated considerable between-cohort variability in discrimination.

Prespecified subgroup analyses were conducted across 15 external validation cohorts to explore potential sources of heterogeneity (S4 Table). Subgroup differences were statistically significant by bowel preparation regimen (*P* for interaction = 0.002) and predictor composition (*P* for interaction = 0.008). Cohorts using a free-choice volume regimen showed the lowest pooled discrimination, with a pooled AUC of 0.67 (95% CI, 0.57–0.76). In contrast, fixed-volume PEG regimens showed higher pooled discrimination, with pooled AUCs of 0.73 (95% CI, 0.66–0.81) for 3 L PEG and 0.72 (95% CI, 0.64–0.79) for 4 L PEG. The 2 L PEG category included only one external validation cohort, which reported an AUC of 0.82 (95% CI, 0.78–0.86); therefore, this estimate should be interpreted cautiously.

When stratified by predictor composition, cohorts evaluating models with patient-related predictors only yielded a pooled AUC of 0.69 (95% CI, 0.63–0.75), whereas models incorporating combined predictors yielded a higher pooled AUC of 0.77 (95% CI, 0.72–0.82); The preparation-related predictors only category included a single external validation cohort, which reported an AUC of 0.80 (95% CI, 0.76–0.84), limiting interpretation of this category. No statistically significant subgroup differences were observed according to participant setting (*P* = 0.061), study design (*P* = 0.488), sample size (*P* = 0.746), or center type (*P* = 0.398). Heterogeneity remained substantial within most subgroups, indicating that multiple factors likely contribute to variability in externally validated discrimination.

Among externally validated models, only a limited number were evaluated across multiple independent cohorts. Pooled results for repeatedly externally validated models (e.g., Dik and Gimeno-García) are presented in S1 Fig. Meta-regression results suggested that predictor composition was associated with heterogeneity (*β* = −0.118; *P* = 0.031). Sample size also showed a statistically significant association (*β* = 0.0002; *P* = 0.047), although the effect size was negligible (S5 Table). Given the limited number of external validation cohorts and sparse categories, these meta-regression findings should be interpreted as exploratory.

#### 3.4.3. Comparison of pooled AUCs from internal and external validation.

In the subgroup comparison by validation type, internally validated results yielded a pooled AUC of 0.76 (95% CI, 0.73–0.79) with substantial heterogeneity (*I²* = 95.2%, *P* < 0.001). Externally validated results yielded a pooled AUC of 0.72 (95% CI, 0.68–0.76), also with substantial heterogeneity (*I²* = 93.9%, *P* < 0.001). The test for subgroup differences indicated no significant difference between internal and external validation (*P* = 0.144), suggesting broadly comparable discrimination across validation types despite marked within-group heterogeneity (S4 Table). The pooled AUC across all validation results was 0.75 (95% CI, 0.72–0.77), but this overall estimate should be interpreted as a descriptive summary given the methodological differences between internal and external validation.

### 3.5. Sensitivity analysis and publication bias

Sensitivity analyses showed that, for both internal and external validation, the pooled AUC estimates remained stable when each study was sequentially excluded (S2 Fig, S3 Fig). The variations were minimal and fell within the 95% confidence intervals of the overall estimates, indicating that the results were robust and not unduly influenced by any single study.

Egger’s tests for both internal and external validation studies indicated no significant publication bias (*P* = 0.233 and *P* = 0.960, respectively). The funnel plots appeared approximately symmetric (S4 Fig, S5 Fig).

### 3.6. Risk of bias and evidence certainty assessment

Risk of bias and applicability were assessed using the PROBAST tool (Table 3) [22, 23]. All included studies were judged to have a high overall risk of bias, indicating methodological shortcomings in the development or validation of the prediction models.

**Table 3:** PROBAST results of the included studies.

Study ID	Study type	ROB				Applicability			Overall	
**Participants**	**Predictors**	**Outcome**	**Analysis**	**Participants**	**Predictors**	**Outcome**	**ROB**	**Applicability**
Berger 2021 [29]	B	+	+	?	–	+	+	+	–	+
Chen 2022 [30]	B	+	+	?	–	+	+	+	–	+
Dik 2015 [16]	B	+	+	?	–	+	+	+	–	+
Fuccio 2021 [31]	B	+	+	?	–	+	+	+	–	+
Gu 2024 [32]	B	+	+	?	–	+	+	+	–	+
Gimeno-García 2017 [33]	B	+	+	?	–	+	+	+	–	+
Hassan 2012 [15]	B	+	+	–	–	+	+	+	–	+
Kutyla 2020 [47]	A	–	+	?	–	+	+	+	–	+
Malkin 2023 [35]	A	+	+	?	–	+	+	+	–	+
Okamoto 2022 [36]	B	+	+	?	–	+	+	+	–	+
Sninsky 2024 [37]	B	–	–	?	–	+	+	+	–	+
Zhang 2024 [38]	B	+	+	?	–	+	+	+	–	+
Zhao 2024 [39]	B	+	+	?	–	+	+	+	–	+
Guo 2023 [45]	B	+	+	?	–	–	+	+	–	–
Huang 2023 [41]	B	–	–	?	–	+	+	+	–	+
Li 2020 [42]	B	+	+	?	–	+	+	+	–	+
Liu 2024 [40]	B	+	+	?	–	–	+	+	–	–
Rao 2021 [43]	B	+	+	?	–	+	+	+	–	+
Wang 2024 [44]	B	–	–	?	–	+	+	+	–	+
Wang 2024 [46]	B	–	–	?	–	–	+	+	–	–
Xu 2022 [54]	B	–	–	?	–	–	+	+	–	–
Zhao 2022 [48]	B	–	–	?	–	+	+	+	–	+
Song 2024 [49]	B	–	–	?	–	+	+	+	–	+
Fang 2025 [52]	B	+	+	+	–	–	+	+	–	–
Afecto 2023 [50]	C	–	–	?	–	+	+	+	–	+
Yuan 2022 [51]	C	–	–	?	–	+	+	+	–	+
Gkolfakis 2023 [34]	C	–	–	?	–	+	+	+	–	+
Apaer 2024 [53]	C	–	–	+	–	+	+	+	–	+
Liu 2025 [55]	B	–	?	?	–	–	–	+	–	–
Wu 2025 [56]	B	+	?	+	–	+	+	+	–	+
Yin 2025 [57]	B	+	+	?	–	+	+	+	–	+

**Abbreviations:** PROBAST, Prediction model Risk of Bias Assessment Tool; ROB, risk of bias; A indicates “development only”; B indicates “development and validation in the same publication”; C indicates “external validation only”; + indicates low ROB/low concern regarding applicability; – indicates high ROB/high concern regarding application;? indicates unclear ROB/unclear concern regarding applicability.

In the Participants domain, nine retrospective studies were judged at high risk of bias because participants were assembled from pre-existing records without clearly defined consecutive (or population-based) sampling, raising concerns about selection bias and limited representativeness [37, 41, 42, 44, 46, 48, 49, 53, 54]. In the predictor domain, the risk of bias was rated high, as no studies reported quality control measures for predictor assessment. For studies lacking specified assessment methods or clear definitions of predictors, the associated risk of bias was rated unclear. For the outcome domain, one study was rated as high risk due to the use of a non-standardized, investigator-defined bowel preparation scale [15]. Although the remaining studies used standardized tools to assess bowel preparation quality, none explicitly reported blinded outcome assessment, resulting in an overall “unclear” risk rating in this domain.

All studies were rated as high risk of bias in the analysis domain. Several recurring methodological limitations were identified. Five studies failed to meet the recommended minimum events-per-variable (EPV ≥ 20) criterion [29,35,36,48,49], raising concerns about model overfitting. Twelve studies categorized continuous variables without providing a clear rationale or justification [16,30,38,40,42,45,46,49,54–57]. Eight studies did not report appropriate handling of missing data [16, 29, 35, 36, 43, 45, 49, 54], and more than half of the studies used univariate analysis to select predictors. Additionally,  thirteen studies did not assess model calibration and reported only discrimination metrics such as the AUC [15,16,29,32–34,42,47,49–51,53,55]. Eleven studies conducted internal validation using random data splitting, a method that may underestimate the risk of overfitting. In terms of applicability, six studies were judged to raise high concerns because they included only subjects aged ≥ 60 years old or ≥ 40 years old, which restricted the sample case-mix and thus limited generalizability. The remaining studies were considered to raise low concerns regarding applicability.

The certainty of evidence for the pooled AUCs from external validation was assessed using the GRADE framework [25]. All three pooled estimates, including those for all models combined, Dik’s model, and Gimeno-García’s model, were rated as having very low certainty. The primary reasons for downgrading included a high risk of bias and substantial inconsistency across studies. In some cases, concerns related to indirectness and imprecision were also present (S6 Table).

## 4. Discussion

Bowel preparation quality is a critical determinant of colonoscopy effectiveness, as IBP directly compromises the accuracy and completeness of the procedure [58]. Identifying patients at high risk of IBP before colonoscopy is therefore important for delivering targeted interventions and optimizing preparation pathways. In this systematic review and meta-analysis of 31 studies encompassing multivariable prediction models for IBP, discrimination was moderate to good on average in both internal and external validations, with reported AUCs ranging from 0.620 to 0.902 across studies. Overall, these findings suggest that pre-procedural risk stratification may inform individualized preparation and follow-up by targeting enhanced support for patients at higher risk of IBP. However, PROBAST assessments indicated that all studies were at high risk of bias, largely due to limitations in the analysis domain, and independent external validation was often lacking, which may limit model reliability and transportability in routine practice.

In quantitative synthesis, we separately aggregated the discriminatory capabilities of internal and external validation. The pooled AUC for internal validation model results was 0.76 (95% CI, 0.73–0.79), while the pooled AUC for external validation results was 0.72 (95% CI, 0.68–0.76). Although the difference was not statistically significant (*P* = 0.144), the trend toward slightly higher performance in internal validation is consistent with optimism commonly observed in development or internal validation settings [60], which may reflect overfitting and differences in case-mix and outcome ascertainment. This underscores the necessity of independent external validation for assessing a model’s generalizability and clinical utility. Notably, both pooled results exhibited substantial heterogeneity (I² > 90%), indicating significant variability in model performance across different cohorts. The very high heterogeneity indicates that the pooled AUC represents an average across highly diverse settings rather than a single transportable estimate.

Exploratory subgroup analyses and meta-regression suggested that heterogeneity in both internal and external validation performance may be partly related to predictor composition. Models incorporating only patient-related factors exhibited relatively lower AUC compared with models that additionally included preparation-related factors. This may relate to preparation-related variables capturing process signals closer to the outcome that are amenable to intervention. Moreover, externally validated performance varied across PEG volume regimens (2 L/ 3 L/ 4 L), raising the possibility that variation in volume-based protocols contributes to between-cohort differences in model performance. Given the limited sample sizes in certain subgroups, these findings should be interpreted with caution and require further confirmation in larger, multi-center, independent validation studies.

From the perspective of predictor composition, models that combined baseline patient characteristics (e.g., age, diabetes, constipation history) with preparation-process variables tended to show higher discrimination than models relying on patient factors alone. Patient factors primarily reflect underlying susceptibility (e.g., age, diabetes, history of constipation), while preparation-related factors indicate modifiable aspects of clinical pathways and implementation processes (e.g., time interval between preparation and examination, preparation type and dosing regimen, patient compliance, and degree of bowel clearance). Such process variables are more closely consistent with the mechanisms underlying outcomes. They not only enhance the discriminatory power in external validation but also provide more direct guidance for nursing interventions and process optimization measures, thereby improving the model’s feasibility for implementation. Future models should standardize the collection and incorporate preparatory process variables based on patients’ baseline risk factors, and assess their incremental value for discrimination, calibration, and clinical net benefit across different healthcare settings.

In the subgroup analysis of external validation, we stratified patients according to PEG bowel preparation solution volume regimens. The results demonstrated significant differences in model discrimination between volume regimens (*P* = 0.002). Specifically, free-choice volume protocols were associated with lower discriminative performance than fixed-volume PEG regimens, with the lowest pooled AUC observed in free-choice cohorts. Several mechanisms may explain this variation. First, the volume of bowel preparation solution itself may serve as an important predictor of IBP [59]. Second, the preparation-solution volume may affect patient adherence, which in turn influences bowel cleanliness and may compromise predictive accuracy [60]. Although models developed under standardized dosing conditions demonstrated better performance, such uniform regimens are often impractical in real-world settings where individualized dosing is more common. Based on these considerations, we recommend that future studies develop prediction models under diverse dosing contexts or explicitly incorporate preparation volume as a predictor variable to enhance their clinical adaptability and applicability.

To assess the clinical utility of existing IBP risk prediction models and identify those with potential for broader implementation, we systematically compared models that had undergone external validation. Although some models (e.g., Dik’s model and Gimeno’s model) have undergone multiple external validations [34, 51] and reported moderate to good AUC values in individual studies, these models generally showed reduced predictive accuracy across studies and were constrained by methodological limitations. Besides, most models underwent only a single, small-sample external validation and were judged to have a high risk of bias. According to GRADE assessments, none of the models reached moderate or high certainty levels. Taken together, the current evidence does not support universal adoption of a single IBP model across settings. Future research should prioritize large-scale, high-quality, multicenter external validation studies, with careful consideration of the original development context to ensure model applicability and generalizability.

While some IBP prediction models demonstrated acceptable discriminative performance, their overall methodological quality remains suboptimal. According to the PROBAST assessment, most studies revealed a high risk of bias across key domains, including participants, predictors, outcomes, and analysis. Common methodological concerns included small sample sizes with low event-per-variable ratios (EPV < 20), inappropriate handling of missing data, the absence of external validation, and insufficient reporting of essential model performance metrics, such as discrimination and calibration. GRADE assessments further rated the overall certainty of evidence as “very low.” These findings are consistent with prior systematic reviews of IBP prediction models in colonoscopy and further underscore the importance of adopting standardized, methodologically sound practices in future model development and validation [61].

In model evaluation, we observed that existing models generally exhibit shortcomings in reporting standards, with very few studies explicitly adhering to the Transparency Reporting of Multivariate Prediction Models for Individual Prognosis or Diagnosis (TRIPOD) statement. This insufficient reporting undermines the transparency of model development and validation, increasing uncertainty and potentially amplifying the risk of bias. On the one hand, many studies provide insufficient reporting of key methodological details, such as missing data handling and treatment of predictors, making it difficult to assess potential issues within the models, including overfitting, information leakage, or selective reporting. Moreover, the frequent absence of critical reproducible information and comprehensive performance metrics (e.g., calibration) hinders the independent replication and validation of models. This lack of transparency compromises not only the implementability of individual models but also the validity of cross-study comparisons and evidence synthesis, ultimately limiting the value of systematic reviews for informing clinical practice. Future research should strictly adhere to the TRIPOD reporting guidelines to mitigate associated risks, reduce bias, and enhance the certainty of evidence for clinical decision-making.

## 5. Implications

This study systematically evaluated the performance and quality of the IBP risk prediction model, providing a reference for its evidence base in clinical application and future research. Early identification of patients at high risk for IBP is essential for developing tailored health education and intervention strategies. Such efforts can enhance bowel preparation adequacy and improve the overall quality of colonoscopy. Risk prediction models for IBP may serve as valuable tools to assist pre-procedural assessments, particularly in settings with high patient volumes and limited staffing, by supporting data-informed decision-making and improving workflow efficiency. However, given the current limitations, such as insufficient external validation and limited generalizability, these models should be considered as complementary tools rather than substitutes for clinical judgment. Future research should prioritize large, multicenter prospective external validations, integrate modifiable preparation-related process variables, and report full performance metrics (including calibration and clinical utility) in accordance with TRIPOD to improve transparency, transportability, and real-world applicability.

## 6. Strengths and limitations

To our knowledge, this is the first systematic review and meta-analysis to quantitatively synthesize discrimination performance (AUC) of multivariable prediction models for inadequate bowel preparation. Following the guidelines of PRISMA and TRIPOD-SRMA, we systematically summarized the characteristics of model development and used PROBAST to assess the risk of bias, providing information for model selection and future optimization.

Nevertheless, several limitations should be acknowledged. First, most included studies were rated as high risk of bias, with generally suboptimal methodological quality, resulting in an overall low certainty of evidence. Second, our meta-analysis focused primarily on discrimination because AUC was the most consistently reported performance measure across the included studies. Calibration information was frequently unavailable or inconsistently reported, limiting our ability to assess whether predicted risks agreed with observed risks. Accordingly, the pooled AUC estimates should be interpreted as summaries of discriminative performance rather than comprehensive evaluations of model performance or clinical applicability. Third, variations in outcome definitions and assessment tools for IBP across studies may have introduced additional heterogeneity, limiting comparability and interpretability. Finally, some studies contributed multiple model estimates, which may challenge the independence assumption of conventional meta-analytic pooling. These limitations highlight the need for future high-quality, standardized studies to confirm and extend our findings.

## 7. Conclusion

This systematic review included 31 studies reporting 46 IBP prediction models. The findings indicate that existing models demonstrate moderate to good discriminatory performance (AUC) in both internal and external validations, suggesting their potential value in risk stratification and informing individualized preparation pathways. However, confidence in their clinical use is limited by a high risk of bias (mainly in the analysis domain), limited independent external validation, and incomplete TRIPOD reporting. Future studies should prioritize large, prospective multicenter external validation and transparent reporting to support reliable implementation.

## Supporting information

S1 Fig Meta-analysis results for repeatedly externally validated models. (DOCX)

S2 Fig Leave-one-out sensitivity analysis (internal validation). (DOCX)

S3 Fig Leave-one-out sensitivity analysis (external validation). (DOCX)

S4 Fig Publication bias assessment (internal validation). (DOCX)

S5 Fig Publication bias assessment (external validation). (DOCX)

S1 Table Searching strategy(full electronic search strategies). (DOCX)

S2 Table Predictors included in each model (final predictors). (DOCX)

S3 Table Meta-analysis and subgroup analyses of internal validation results. (DOCX)

S4 Table Meta-analysis and subgroup analyses of external validation results. (DOCX)

S5 Table Meta-regression for external validation results. (DOCX)

S6 Table GRADE assessment of certainty of evidence. (DOCX)

S7 Table PRISMA 2020 checklist. (DOCX)

S8 Table Study selection details for included and excluded records. (DOCX)
