# Supplementary material for: Risk prediction models for inadequate bowel preparation before colonoscopy: A systematic review and meta-analysis
Source: PLoS One. 2026 Aug 14;21(8):e0356009. doi: 10.1371/journal.pone.0356009 (PMC13475969; doi:10.1371/journal.pone.0356009)
Supplement: Supplementary file 4 [file pone.0356009.s004.docx]

**S4 Fig.** Publication bias assessment (internal validation)

**S4A Fig. Egger’s test for internal validation.**

**S4B Fig. Funnel plot for internal validation.**
