# Supplementary material for: Risk prediction models for inadequate bowel preparation before colonoscopy: A systematic review and meta-analysis
Source: PLoS One. 2026 Aug 14;21(8):e0356009. doi: 10.1371/journal.pone.0356009 (PMC13475969; doi:10.1371/journal.pone.0356009)
Supplement: Supplementary file 5 [file pone.0356009.s005.docx]

**S5 Fig.** Publication bias assessment (external validation)

**S5A Fig. Egger’s test for external validation.**

**S5B Fig. Funnel plot for external validation.**
