# Supplementary material for: Risk prediction models for inadequate bowel preparation before colonoscopy: A systematic review and meta-analysis
Source: PLoS One. 2026 Aug 14;21(8):e0356009. doi: 10.1371/journal.pone.0356009 (PMC13475969; doi:10.1371/journal.pone.0356009)
Supplement: Supplementary file 6 [file pone.0356009.s006.docx]

**S1 Table.** Searching strategy (full electronic search strategies) **Database:** PubMed

**Time:** From the database construction to December 2025

| **PubMed** | | |
| --- | --- | --- |
| **#** | **Query** | **Result** |
| **Colonoscopy** | | |
| 1 | "colonoscopy"[MeSH Terms] | 38,217 |
| 2 | "colonoscopy"[Title/Abstract] OR "colonoscopic"[Title/Abstract] OR "enteroscopy"[Title/Abstract] OR "endoscopy gastrointestinal"[Title/Abstract] | 45,457 |
| 3 | #1 OR #2 | 62,434 |
| **Bowel preparation** | | |
| 4 | "bowel preparation"[Title/Abstract] OR "bowel prep"[Title/Abstract] OR "bowel cleansing"[Title/Abstract] OR "bowel cleaning"[Title/Abstract] OR "colon cleansing"[Title/Abstract] OR "colon cleaning"[Title/Abstract] OR "colonic cleansing"[Title/Abstract] OR "intestinal preparation"[Title/Abstract] OR "inadequate bowel preparation"[Title/Abstract] OR "poor bowel preparation"[Title/Abstract] | 6,020 |
| 5 | #3 AND #4 | 3,535 |
| **Predictive model** | | |
| 6 | "risk assessment"[MeSH Terms] | 349,979 |
| 7 | "risk factors"[MeSH Terms] | 1,088,624 |
| 8 | "predict*"[Title/Abstract] OR "prediction model"[Title/Abstract] OR "predictive model"[Title/Abstract] OR "predict model"[Title/Abstract] OR "prognostic model"[Title/Abstract] OR "risk prediction"[Title/Abstract] OR "risk prediction model"[Title/Abstract] OR "risk assessment"[Title/Abstract] OR "risk factors"[Title/Abstract] OR "risk score"[Title/Abstract] | 3,237,506 |
| 9 | #6 OR #7 OR #8 | 3,987,058 |
| 10 | #5 AND #9 | 570 |

**Database:** Cochrane Library

**Time:** From the database construction to December 2025

| **Cochrane Library** | | |
| --- | --- | --- |
| **#** | **Query** | **Result** |
| **Colonoscopy** | | |
| **1** | MeSH descriptor: [Colonoscopy] explode all trees | 3,189 |
| **2** | ("colonoscopy" OR "colonoscopic" OR "enteroscopy" OR "endoscopy gastrointestinal"):ti, ab, kw | 11,843 |
| **3** | #1 OR #2 | 12,075 |
| **Bowel preparation** | | |
| **4** | ("bowel preparation" OR “bowel prep” OR “bowel cleansing” OR “bowel cleaning” OR “colon cleansing” OR "colon cleaning" OR “colonic cleansing” OR “intestinal preparation” OR “inadequate bowel preparation” OR “poor bowel preparation”): ti, ab, kw | 3,994 |
| **5** | #3 AND #4 | 2,917 |
| **Predictive model** | | |
| **6** | MeSH descriptor: [Risk Assessment] explode all trees | 13,910 |
| **7** | MeSH descriptor: [Risk Factors] explode all trees | 38,830 |
| **8** | (predict*): ti, ab, kw | 136,165 |
| **9** | ("prediction model" OR "predictive model" OR "predict model" OR "prognostic model" OR "risk prediction" OR "risk prediction model" OR "risk assessment" OR "risk factors" OR "risk score"): ti, ab, kw | 126,354 |
| **10** | #6 OR #7 OR #8 OR #9 | 238,490 |
| **11** | #5 AND #10 | 330 |

**Database:** Web of Science

**Time:** From the database construction to December 2025

| **Web of Science** | | |
| --- | --- | --- |
| **#** | **Query** | **Result** |
| Colonoscopy | | |
| **1** | colonoscopy (Topic) or colonoscopies (Topic) or enteroscopy (Topic) or gastrointestinal endoscopy (Topic) or colonoscopic (Topic) | 42,052 |
| **Bowel preparation** | | |
| **2** | bowel preparation (Topic) or bowel cleansing (Topic) or bowel cleaning (Topic) or colon cleansing (Topic) or colon cleaning (Topic) or intestinal preparation (Topic) or intestine preparation (Topic) or inadequate bowel preparation (Topic) or poor bowel preparation (Topic) | 6,100 |
| **Predictive model** | | |
| **3** | predict* (Topic) or prediction model (Topic) or predictive model (Topic) or prognostic model (Topic) or risk prediction model (Topic) or risk assessment (Topic) or risk factors (Topic) or risk factors model (Topic) or risk assessment model (Topic) or predict (Topic) | 4,717,736 |
| **4** | #1 AND #2 AND #3 | 631 |

**Database:** Embase

**Time:** From the database construction to December 2025

| **Embase** | | |
| --- | --- | --- |
| # | **Query** | **Result** |
| **Colonoscopy** | | |
| 1 | 'colonoscopy'/exp | 140,019 |
| 2 | 'colonoscopy':ti,ab,kw OR 'colonoscopies':ti,ab,kw OR 'colonoscopic':ti,ab,kw OR 'intestine endoscopy':ti,ab,kw OR 'gastrointestinal endoscopy':ti,ab,kw | 111,404 |
| 3 | #1 OR #2 | 159,385 |
| **Bowel preparation** | | |
| 4 | 'intestine preparation'/exp | 13,283 |
| 5 | 'intestine preparation':ti,ab,kw OR 'bowel preparation':ti,ab,kw OR 'bowel cleansing':ti,ab,kw OR 'inadequate bowel preparation':ti,ab,kw OR 'poor bowel preparation':ti,ab,kw | 12,841 |
| 6 | #4 OR #5 | 16,061 |
| **Predictive model** | | |
| 7 | 'predictive model'/exp | 56,078 |
| 8 | 'predictive model':ti,ab,kw OR 'predict':ti,ab,kw OR 'predict model':ti,ab,kw OR 'risk prediction':ti,ab,kw OR 'risk prediction model':ti,ab,kw OR 'risk assessment':ti,ab,kw OR 'risk assessment model':ti,ab,kw OR 'risk score':ti,ab,kw OR 'risk score model':ti,ab,kw | 1,127,466 |
| 9 | #7 OR #8 | 1,151,557 |
| 10 | #3 AND #6 AND #9 | 359 |

**Database:** CINAHL

**Time:** From the database construction to December 2025

| **CINAHL** | | |
| --- | --- | --- |
| **#** | **Query** | **Result** |
| **Colonoscopy** | | |
| 1 | MH (colonoscopy) OR XB (colonoscopies) OR XB (colonoscopic) OR XB (enteroscopy) OR XB (gastrointestinal endoscopy) | 23,076 |
| **Bowel preparation** | | |
| 2 | MH (bowel preparation) OR MH (bowel cleansing) OR MH (bowel cleaning) OR XB (colon cleansing) OR XB (colon cleaning) OR XB (intestinal preparation) OR XB (intestine preparation) OR XB (inadequate bowel preparation) OR XB (poor bowel preparation) | 2,198 |
| **Predictive model** | | |
| 3 | MH (prediction model) OR MH (risk assessment) OR MH (risk factors) OR XB (predictive) OR XB (predictive model) OR XB (predict model) OR XB (risk prediction) OR XB (risk prediction model) OR XB (risk score) OR XB (risk score model) | 817,469 |
| 4 | #1 AND #2 AND #3 | 62 |

**Database:** China National Knowledge Infrastructure(CNKI)

**Time:** From the database construction to December 2025

| **CNKI** | **Result** |
| --- | --- |
| TKA=("colonoscopy" OR "colonoscopy examination" OR "colonoscope") AND TKA=("inadequate bowel preparation" OR "insufficient bowel preparation" OR "bowel cleansing" OR "bowel preparation") AND TKA=("prediction" OR "prediction model" OR "risk prediction model" OR "predictive factor" OR "prediction score" OR "risk score" OR "nomogram" OR "risk factor" OR "influencing factor") | 402 |

**Database:** Wan Fang

**Time:** From the database construction to December 2025

| **Search formula** | **Result** |
| --- | --- |
| Topic: ("colonoscopy" OR "colonoscopy examination" OR "gastrointestinal endoscopy") AND Topic: ("bowel preparation" OR "bowel cleansing" OR "inadequate bowel preparation" OR "insufficient bowel preparation") AND Topic: ("model" OR "risk factor" OR "predictive factor" OR "influencing factor" OR "risk prediction" OR "risk prediction model" OR "nomogram" OR "risk prediction tool" OR "risk prediction score") | 525 |

**Database:** VIP Database

**Time:** From the database construction to December 2025

| **Search formula** | **Result** |
| --- | --- |
| (Title = "colonoscopy examination" OR Title = "colonoscopy" OR Keyword = "colonoscopy examination" OR Keyword = "colonoscopy" OR Abstract = "colonoscopy examination" OR Abstract = "colonoscopy") AND (Title = "inadequate bowel preparation" OR Title = "insufficient bowel preparation" OR Title = "bowel cleansing" OR Title = "bowel preparation" OR Keyword = "inadequate bowel preparation" OR Keyword = "insufficient bowel preparation" OR Keyword = "bowel cleansing" OR Keyword = "bowel preparation" OR Abstract = "inadequate bowel preparation" OR Abstract = "insufficient bowel preparation" OR Abstract = "bowel cleansing" OR Abstract = "bowel preparation") AND (Title = "prediction" OR Title = "prediction model" OR Title = "risk prediction model" OR Title = "predictive factor" OR Title = "prediction score" OR Title = "risk score" OR Title = "nomogram" OR Title = "risk factor" OR Title = "influencing factor" OR Keyword = "prediction" OR Keyword = "prediction model" OR Keyword = "risk prediction model" OR Keyword = "predictive factor" OR Keyword = "prediction score" OR Keyword = "risk score" OR Keyword = "nomogram" OR Keyword = "risk factor" OR Keyword = "influencing factor" OR Abstract = "prediction" OR Abstract = "prediction model" OR Abstract = "risk prediction model" OR Abstract = "predictive factor" OR Abstract = "prediction score" OR Abstract = "risk score" OR Abstract = "nomogram" OR Abstract = "risk factor" OR Abstract = "influencing factor") | 132 |
