# Supplementary material for: Risk prediction models for inadequate bowel preparation before colonoscopy: A systematic review and meta-analysis
Source: PLoS One. 2026 Aug 14;21(8):e0356009. doi: 10.1371/journal.pone.0356009 (PMC13475969; doi:10.1371/journal.pone.0356009)
Supplement: Supplementary file 7 [file pone.0356009.s007.docx]

**S2 Table.** Predictors included in each model (final predictors)

| **Author(year)** | **Final predictors** |
| --- | --- |
| Afecto et al. (2023) | The predictors of Dik’s model; the predictors of Gimeno-García’s model |
| Chen et al. (2022) | Drinking-to-stool interval, last bowel movement characteristics, preparation-to-colonoscopy interval (3) |
| Huang et al. (2023) | Hypertension, history of constipation, statin use, number of long-term medications, volume of preparation (5) |
| Song et al. (2024) | Diabetes, constipation, history of IBP, history of colorectal surgery, dietary compliance, ASA score (6) |
| Liu et al. (2024) | Hospitalization status, age, history of colonoscopy, BSFS type (4) |
| Malkin et al. (2023) | Prior cerebrovascular accident, Grade of education, bowel preparation regimen, consumption of bisacodyl, bowel movement frequency, bisacodyl use before colonoscopy (6) |
| Xu et al. (2022) | Age, BMI, constipation, diabetes, history of colorectal surgery, history of colonoscopy (6) |
| Rao et al. (2021) | Age, BMI, ethnic group, history of smoking, constipation, history of abdominal/pelvic surgery, history of IBP (7) |
| Guo et al. (2023) | Age, diabetes, exercise habits, BSFS type, frailty state, walking function status (6) |
| Yuan et al. (2022) | The predictors of Dik’s model; The predictors of Gimeno’s model |
| Gkolfakis et al. (2023) | The predictors of Dik’s model; The predictors of Fuccio’s model; The predictors of Gimeno’s model |
| Okamoto et al. (2022) | ASA score, diabetes, use of laxatives, defecation habits, use of drugs for mental disorders (5) |
| Zhang et al. (2024) | Age, sex, BMI, history of smoking, chronic constipation, diabetes, history of colorectal surgery (7) |
| J X. Wang et al. (2024) | Age, BMI, diabetes, history of colorectal surgery, history of colonoscopy, BSFS type (6) |
| Hassan et al. (2012) | Sex, age, BMI, diabetes, cirrhosis, parkinson disease, previous colorectal surgery, positive fecal occult blood test (8) |
| Dik et al. (2015) | ASA score, tricyclic antidepressant use, opioid use, diabetes, chronic constipation, history of intra-abdominal/pelvic surgery, current hospitalization, history of IBP (8) |
| Berger et al. (2021) | Diabetes and/or BMI ≥30 kg/m2, irregular physical activity, cirrhosis, antidepressant or neuroleptic use, opioid use, history of abdominal and/or pelvic surgery, history of IBP (7) |
| Fuccio et al. (2021) | Bedridden status, constipation, diabetes, antipsychotic drugs use, split-dose regimen, PEG regimen volume, compliance to preparation (7) |
| Li et al. (2020) | Optimal subset method model*: high fiber diet before colonoscopy, diabetes, history of constipation, preoperative activity (4) |
| Zhao et al. (2022) | Age, length of hospitalization, diabetes, constipation, bowel movement frequency (5) |
| Kutyla et al. (2020) | Employment status, full amount of bowel preparation not consumed, use of opioids or other constipating medications, last rectal efﬂuent (4) |
| Sninsky et al. (2024) | History of smoking, race, edicaid insurance, medicare insurance, employed status, diabetes, inflammatory bowel disease, constipation, insulin use, opioid use, oral diabetic medications use, antipsychotics use, antidepressants use, the “percent of fair or poor health” within the patient’s county (14) |
| G H. Wang et al. (2024) | Age, Preparation-to-colonoscopy interval, history of constipation, completion of the laxative regimen, companion situation during the procedure (5) |
| Zhao et al. (2024) | Volume of preparation; split-dose regimen; dietary compliance; additional water intake during preparation; preparation-to-colonoscopy interval; bowel movement frequency; last rectal efﬂuent (7) |
| Gimeno-García et al. (2017) | Antidepressant use, comorbidity, constipation, history of abdominal and/or pelvic surgery (4) |
| Gu et al. (2024) | Age, BMI, Grade of education, simethicone use, diabetes, history of IBP, preparation-to-colonoscopy interval (7) |
| Fang et al.(2025) | BMI, comorbidity, constipation history, frailty state, prescribing doctor's specialty, nonpatient's own prescribing, anxiety level, history of abdominal surgery (8) |
| Apaer et al.(2024) | The predictors of Rao’s model |
| Yin et al.(2025) | the high fiber diet used at 24 h before examination, PC time > 5 h, male sex, chronic constipation, diabetes, history of colorectal surgery (6) |
| Wu et al.(2025) | BSFS type, PEG regimen volume, delayed last bowel movement, bowel movement frequency (4) |
| Liu et al.(2025) | split-dose regimen, in-hospital bowel preparation, grade of education, use of calcium channel blockers (CCB), bowel movement status, activity level, and last bowel movement characteristics (7) |
