# Supplementary material for: Risk prediction models for inadequate bowel preparation before colonoscopy: A systematic review and meta-analysis
Source: PLoS One. 2026 Aug 14;21(8):e0356009. doi: 10.1371/journal.pone.0356009 (PMC13475969; doi:10.1371/journal.pone.0356009)
Supplement: Supplementary file 8 [file pone.0356009.s008.docx]

**S3 Table.** Meta-analysis and subgroup analyses of internal validation results

| **Subgroup** | **No. of models** | **Summary of AUC (95%CI)** | **I^2^ (%)** | ***P* for interaction** |
| --- | --- | --- | --- | --- |
| **Machine learning** | | | | 0.745 |
| Yes | 13 | 0.75 (0.71–0.79) | 95.0 |  |
| No | 18 | 0.76 (0.72–0.80) | 95.0 |  |
| **Participants** | | | | 0.439 |
| Not specified | 20 | 0.76 (0.72–0.78) | 94.2 |  |
| Inpatients | 3 | 0.80 (0.68–0.91) | 94.2 |  |
| Outpatients | 8 | 0.74 (0.71–0.77) | 91.8 |  |
| **Sample size** | | | | 0.061 |
| <500 | 12 | 0.73 (0.68–0.77) | 97.2 |  |
| ≥500 | 19 | 0.78 (0.75–0.81) | 90.5 |  |
| **Study design type** | | | | **< 0.001** |
| Prospective cohort study | 23 | 0.77 (0.73–0.80) | 95.4 |  |
| Retrospective cohort study | 6 | 0.70 (0.65–0.74) | 94.2 |  |
| Case-control study | 2 | 0.87 (0.83–0.91) | - |  |
| **Center type** | | | | 0.158 |
| Single center | 18 | 0.78 (0.73–0.83) | 96.1 |  |
| Multi-center | 13 | 0.74 (0.70–0.77) | 93.6 |  |
| **Predictor factors type** | | | | **0.039** |
| Patient-related only | 12 | 0.73 (0.69–0.77) | 93.1 |  |
| Preparation-related only | 1 | 0.81 (0.76–0.86) | - |  |
| Combined | 18 | 0.77 (0.74–0.81) | 94.2 |  |

**Note**: Summary AUCs were pooled using random-effects meta-analysis. P-for-interaction indicates the between-subgroup difference (test of subgroup differences). I² was not estimated when fewer than two models were available.
