# Supplementary material for: Risk prediction models for inadequate bowel preparation before colonoscopy: A systematic review and meta-analysis
Source: PLoS One. 2026 Aug 14;21(8):e0356009. doi: 10.1371/journal.pone.0356009 (PMC13475969; doi:10.1371/journal.pone.0356009)
Supplement: Supplementary file 9 [file pone.0356009.s009.docx]

**S4 Table.** Meta-analysis and subgroup analyses of external validation results

| **Subgroup** | **No. of models** | **Summary of AUC (95% CI)** | **I^2^ (%)** | ***P* for interaction** |
| --- | --- | --- | --- | --- |
| **Validation method** | | | | 0.144 |
| External validation | 15 | 0.72 (0.68–0.76) | **93.9** |  |
| Internal validation | 31 | 0.76 (0.73–0.79) | **95.2** |  |
| **External validation (n=15)** | | | | |
| **Participants** | | | | 0.061 |
| Not specified | 9 | 0.71 (0.66–0.77) | 88.9 |  |
| Inpatients | 4 | 0.74 (0.65–0.83) | 98.1 |  |
| Outpatients | 2 | 0.71 (0.66–0.77) | 0.0 |  |
| **Study design type** | | | | 0.488 |
| Prospective cohort study | 9 | 0.73 (0.68–0.78) | 85.0 |  |
| Retrospective cohort study | 6 | 0.70 (0.63–0.78) | 97.1 |  |
| **Sample size** | | | | 0.746 |
| <500 | 11 | 0.71 (0.67–0.76) | 94.3 |  |
| ≥500 | 4 | 0.74 (0.61–0.86) | 93.7 |  |
| **Bowel preparation regimen** | | | | **0.002** |
| 2L PEG | 1 | 0.82 (0.78–0.86) | - |  |
| 3L PEG | 6 | 0.73 (0.66–0.81) | 87.5 |  |
| 4L PEG | 5 | 0.72 (0.64–0.79) | 97.3 |  |
| Free-choice regimen | 3 | 0.67 (0.57–0.76) | 76.5 |  |
| **Center type** | | | | 0.398 |
| Single center | 7 | 0.74 (0.67–0.81) | 90.6 |  |
| Multi-center | 8 | 0.70 (0.64–0.76) | 95.7 |  |
| **Predictor factors type** | | | | **0.008** |
| Patient-related only | 9 | 0.69 (0.63–0.75) | 95.7 |  |
| Preparation-related only | 1 | 0.80 (0.76–0.84) | - |  |
| Combined | 5 | 0.77 (0.72–0.82) | 59.4 |  |

**Note:** Each record represents an external validation cohort (not a unique model). Summary AUCs were pooled using a random-effects meta-analysis. *P* for interaction indicates between-subgroup differences. I² was not estimated when fewer than two cohorts were available.
