# Supplementary material for: Risk prediction models for inadequate bowel preparation before colonoscopy: A systematic review and meta-analysis
Source: PLoS One. 2026 Aug 14;21(8):e0356009. doi: 10.1371/journal.pone.0356009 (PMC13475969; doi:10.1371/journal.pone.0356009)
Supplement: Supplementary file 10 [file pone.0356009.s010.docx]

**S5 Table.** Meta-regression for external validation results

| **Predictor** | **Coefficient** | **Std. err** | **t** | **P > \|t\|** | **95% CI** |
| --- | --- | --- | --- | --- | --- |
| Predictors(combined factors vs patient-related factors only) | -0.1185089 | 0.0478017 | -2.98 | **0.031** | -0.2237197, -0.0132982 |
| Sample size | 0.0002024 | 0.0000904 | 2.24 | **0.047** | 3.57e-06, 0.0004013 |

Number of obs = 15

Tau² = 0.004783

I² = 94.20%

Adjusted R² = 32.13%

Prob > F = 0.0730
