# Supplementary material for: Risk prediction models for inadequate bowel preparation before colonoscopy: A systematic review and meta-analysis
Source: PLoS One. 2026 Aug 14;21(8):e0356009. doi: 10.1371/journal.pone.0356009 (PMC13475969; doi:10.1371/journal.pone.0356009)
Supplement: Supplementary file 11 [file pone.0356009.s011.docx]

**S6 Table.** GRADE assessment of certainty of evidence

Based on the GRADE framework and Grade concept paper 8 [1], we evaluated the certainty of evidence for each pooled discrimination performance estimate according to the following criteria:

**Risk of Bias:** We assessed the risk of bias using the PROBAST tool. If all contributing studies were judged to be at low risk of bias across the four domains—participants, predictors, outcome, and analysis—the level of concern was rated as “No concern.” If some studies had methodological limitations or at least one domain was rated as “High risk of bias,” but the overall risk was still acceptable, the rating was “Some concern.” If most studies were at high risk of bias, the concern was rated as “Major concern.”

**Inconsistency**: We assessed between-study consistency using the I² statistic and the variability of AUC values across studies. If I² was <40% and the AUC estimates were similar, we rated inconsistency as “No concern.” If I² was between 40% and 75%, or there was moderate variation in AUCs across studies, we rated it as “Some concern.” If I² exceeded 75% with substantial differences in AUCs, inconsistency was rated as “Major concern.”

**Indirectness:** We assessed whether the included studies directly addressed the review question, including whether the target population comprised adults undergoing colonoscopy, whether the outcome was IBP, and whether only pre-procedural predictors were used. If all studies were highly consistent with the target question, indirectness was rated as “No concern.” If some studies used different preparation scales or included special populations (e.g., elderly or hospitalized patients), we rated indirectness as “Some concern.” If multiple studies substantially differed from the intended clinical context, we rated it as “Major concern.”

**Imprecision**: According to the GRADE concept paper 8 [1], imprecision was assessed by referencing AUC = 0.5 as the minimum threshold indicating performance superior to chance. If the 95% CI of the pooled AUC included 0.5, this was rated as a “Major concern.” If the 95% CI was entirely above 0.5 but the lower bound was below the commonly accepted threshold of 0.7 [2], it was rated as a “Some concern.” If the entire 95% CI was above 0.7, imprecision was considered to pose “No concern.”

**Publication bias**: Publication bias was assessed using Egger’s regression test and visual inspection of funnel plot symmetry. If Egger’s test was non-significant and asymmetry was mild, considering the limited interpretability of funnel plots in prediction model research, we rated it as “No concern”. In cases where fewer than 10 studies were included, publication bias was rated as “Not clear”, in accordance with GRADE [3] and Cochrane recommendations [4].

| **Pooled model** | **Risk of Bias** | **Inconsistency** | **Indirectness** | **Imprecision** | **Publication bias** | **Confidence rating** |
| --- | --- | --- | --- | --- | --- | --- |
| The pooled AUC from external validations of all models | Major concern | Major concern | Some concern | Some concern | No concern | Very low |
| The pooled AUC from external validations of Dik’s model | Major concern | Major concern | No concern | Some concern | Not clear | Very low |
| The pooled AUC from external validations of Gimeno’s model | Major concern | Some concern | No concern | Some concern | Not clear | Very low |

**References**

1.	Foroutan F, Mayer M, Guyatt G, Riley RD, Mustafa R, Kreuzberger N, et al. GRADE concept paper 8: judging the certainty of discrimination performance estimates of prognostic models in a body of validation studies. J Clin Epidemiol. 2024;170:111344. doi: 10.1016/j.jclinepi.2024.111344.

2.	Hosmer DW, Lemeshow S. Applied logistic regression. 2nd ed. New York: Wiley; 2000. Available from: <https://doi.org/10.1002/0471722146>.

3.	Guyatt GH, Oxman AD, Vist GE, Kunz R, Falck-Ytter Y, Alonso-Coello P, et al. GRADE: an emerging consensus on rating quality of evidence and strength of recommendations. BMJ (Clinical research ed). 2008;336(7650):924–6. doi: 10.1136/bmj.39489.470347.AD.

4.	Higgins JPT, Thomas J, Chandler J, Cumpston M, Li T, Page MJ, et al. Cochrane Handbook for Systematic Reviews of Interventions Version 6.5 (updated August 2024). Cochrane; 2024. Available from: <www.cochrane.org/handbook>.
