# Supplementary material for: Risk prediction models for inadequate bowel preparation before colonoscopy: A systematic review and meta-analysis
Source: PLoS One. 2026 Aug 14;21(8):e0356009. doi: 10.1371/journal.pone.0356009 (PMC13475969; doi:10.1371/journal.pone.0356009)
Supplement: Supplementary file 13 [file pone.0356009.s013.docx]

**S8 Table.** Study selection details for included and excluded records

**S8 Table 1.** Records excluded after title and abstract screening (n = 1,662)

| **No.** | **Reference** | **Reason for exclusion** |
| --- | --- | --- |
| 1 | Stewart BT, Keck JO, Duncan AV, Santamaria NM, Allen P. Difficult or incomplete flexible sigmoidoscopy: Implications for a screening programme[J]. Australian and New Zealand Journal of Surgery, 1999, 69(1): 19-21. doi: 10.1046/j.1440-1622.1999.01499.x. | Irrelevant Topic |
| 2 | Sun YF, Wang Q, Han RN, et al. Influencing factors for successful cecal intubation in MSCT virtual colonoscopy[J]. Imaging Science and Photochemistry, 2023, 41(1): 48-52. doi: 10.7517/issn.1674-0475.221105. | Irrelevant Topic |
| 3 | Ramakrishnan K, Scheid DC. Predictors of incomplete flexible sigmoidoscopy[J]. The Journal of the American Board of Family Practice, 2003, 16(6): 478-484. doi: 10.3122/jabfm.16.6.478. | Irrelevant Topic |
| 4 | Bell GD, Quine A. Preparation, premedication, and surveillance[J]. Endoscopy, 2006, 38(2): 105-109. doi: 10.1055/s-2005-921205. | Irrelevant Topic |
| 5 | Jiao FF, Liu ZF, Shen YC, et al. Factors influencing the quality of bowel preparation with polyethylene glycol electrolyte powder combined with dietary control before colonoscopy in children[J]. Chinese Journal of Contemporary Pediatrics, 2022, 24(4): 366-371. doi: 10.7499/j.issn.1008-8830.2111019. | Irrelevant Topic |
| 6 | Jensch S, Bipat S, Peringa J, et al. Prospective assessment of patient acceptance of CT colonography with limited bowel preparation vs. cathartic colonoscopy[J]. International Journal of Medical Radiology, 2010, 33(2): 193. | Irrelevant Topic |
| 7 | Takahashi Y, Tanaka H, Kinjo M, Sakumoto K, Zhao M. Prospective evaluation of predictors of difficulty and pain during sedation-free colonoscopy[J]. World Core Medical Journal Abstracts (Gastroenterology), 2005, (11): 21-22. | Irrelevant Topic |
| 8 | Abudukeyoumu A, Maihemiti D, Alimujiang A, et al. Clinical application of CT virtual colonoscopy in colorectal cancer[J]. Chinese Journal of Medical Innovation, 2019, 16(7): 133-136. doi: 10.3969/j.issn.1674-4985.2019.07.034. | Irrelevant Topic |
| 9 | Li L, Wang H, Wang LM. Investigation and analysis of current status of knowledge, attitude and practice of bowel preparation before colonoscopy among gastroenterology nurses[J]. Clinical Nursing Research, 2024, 33(14): 82-84. doi: 10.3969/j.issn.2097-1958.2024.14.028. | Irrelevant Topic |
| 10 | Bai AL, Hou LY, Zhang HL, et al. Analysis of influencing factors and nursing countermeasures for unqualified bowel preparation before colonoscopy in patients with chronic constipation[J]. Chinese Journal of Modern Nursing, 2022, 28(1): 86-90. doi: 10.3760/cma.j.cn115682-20210607-02464. | The population does not match |
| 11 | Tu YH. Effect of nursing intervention on bowel preparation before colonoscopy[J]. Diet and Health, 2018, 5(18): 176-177. doi: 10.3969/j.issn.2095-8439.2018.18.220. | Irrelevant Topic |
| 12 | Song X. Exploring the effect of exercise factors on bowel preparation for colonoscopy[J]. Special Health, 2019, (35): 173-174. doi: 10.3969/j.issn.2095-6851.2019.35.231. | Irrelevant Topic |
| 13 | Cai LH, Li H, Wang LZ, et al. Research progress on nursing intervention for bowel preparation quality before colonoscopy in diabetic patients[J]. Chinese General Practice Nursing, 2021, 19(23): 3206-3209. doi: 10.12104/j.issn.1674-4748.2021.23.010. | Review |
| 14 | Wang YJ, Liu HS, Wang X, et al. Effect of bowel preparation failure risk prediction model combined with time-based incentive theory on bowel preparation in patients undergoing first colonoscopy[J]. Journal of Clinical General Surgery (Electronic Edition), 2025, 13(4): 9-18,33. doi: 10.3969/j.issn.2095-5308.2025.04.004. | Irrelevant Topic |
| 15 | Cai XL, Shen YH, Li QL, et al. Feasibility study of bowel preparation for colonoscopy in ambulatory surgery ward for digestive endoscopy[J]. Chinese Journal of Digestive Endoscopy, 2019, 36(5): 334-338. doi: 10.3760/cma.j.issn.1007-5232.2019.05.007. | Irrelevant Topic |
| 16 | Cai YL, Zhou ST, Zhang MQ. Current status, influencing factors and strategies of inadequate bowel preparation for colonoscopy in diabetic patients[J]. Modern Clinical Nursing, 2023, 22(11): 26-31. doi: 10.3969/j.issn.1671-8283.2023.11.004. | The population does not match |
| 17 | Lyu Z, Min H. Application of dietary control in bowel preparation for colonoscopy[J]. Medical Journal of Peking Union Medical College Hospital, 2021, 12(2): 244-248. doi: 10.3969/j.issn.1674-9081.2020.00.017. | Irrelevant Topic |
| 18 | Li YM. Exploration and research progress of nursing techniques for cleaning enema[J]. Chinese General Practice Nursing, 2013, 11(21): 2002-2003. doi: 10.3969/j.issn.1674-4748.2013.21.057. | Review |
| 19 | Liu H. Nursing progress of bowel preparation before colonoscopy[J]. Chinese General Practice Nursing, 2009, 7(16): 1481-1483. doi: 10.3969/j.issn.1674-4748.2009.16.058. | Irrelevant Topic |
| 20 | Cao Y, Zhao JT, Chen DF, et al. Effect of different education methods on bowel preparation in outpatients undergoing colonoscopy[J]. Laboratory Medicine and Clinic, 2022, 19(22): 3085-3089. doi: 10.3969/j.issn.1672-9455.2022.22.016. | Irrelevant Topic |
| 21 | Chen CH, Chen ML, Zhang J, et al. Effect of waiting time on physical and mental status and bowel cleanliness in patients undergoing colonoscopy for health examination[J]. International Journal of Nursing, 2023, 42(12): 2226-2229. doi: 10.3760/cma.j.cn221370-20200605-00546. | Irrelevant Topic |
| 22 | Chen G, Sun J, Li XL. Diagnosis, treatment and follow-up of colorectal polyps in 2667 patients[J]. Chinese Journal of Gastroenterology and Hepatology, 2018, 27(3): 308-313. doi: 10.3969/j.issn.1006-5709.2018.03.015. | Irrelevant Topic |
| 23 | Chen HD, Luo ZM, Pan JR. Observation on detection rate of colorectal adenoma in left and right colon by colonoscopy and its influencing factors[J]. Journal of Hunan Normal University (Medical Science), 2022, 19(4): 16-19. doi: 10.3969/j.issn.1673-016X.2022.04.004. | Irrelevant Topic |
| 24 | Tang YF, Xiao XL, Bi ES, et al. Effect of family participation on colorectal cancer screening in middle-aged health examination population[J]. Clinical Medical Engineering, 2014(6): 801-802. doi: 10.3969/j.issn.1674-4659.2014.06.0801. | Irrelevant Topic |
| 25 | Shao RL. Effect of education methods on bowel preparation quality and adverse reactions in patients undergoing colonoscopy[J]. Journal of Shandong Medical College, 2018, 40(6): 479-480. doi: 10.3969/j.issn.1674-0947.2018.06.039. | Irrelevant Topic |
| 26 | Wang W, Chen XH, Xu DX. Technical factors affecting the efficacy of colonic hydrotherapy and clinical experience[J]. Journal of Coloproctological Surgery, 2005, 11(4): 305-306. doi: 10.3969/j.issn.1674-0491.2005.04.033. | Irrelevant Topic |
| 27 | Ma YP, Shen XF, Qian MP, et al. Effect of multi-mode health education on bowel preparation in elderly patients undergoing colonoscopy[J]. Chinese Journal of Modern Doctor, 2024, 62(24): 50-53, 63. doi: 10.3969/j.issn.1673-9701.2024.24.010. | Irrelevant Topic |
| 28 | Li J. Diagnostic value of emergency colonoscopy under different bowel cleanliness for acute lower gastrointestinal bleeding[D]. Guangdong: Southern Medical University, 2017. doi: 10.7666/d.Y3280976. | Irrelevant Topic |
| 29 | Lin JH, Liu YW, Lin CQ, et al. Analysis of factors influencing the accuracy of colonoscopy in children with colorectal polyps[J]. Nursing Practice and Research, 2020, 17(4): 142-144. doi: 10.3969/j.issn.1672-9676.2020.04.055. | Irrelevant Topic |
| 30 | Liu YH, Zhan LL, Chen GQ, et al. Detection rate of colorectal sessile serrated adenoma and its related influencing factors[J]. Journal of Tropical Medicine, 2019, 19(1): 65-67, third cover. doi: 10.3969/j.issn.1672-3619.2019.01.016. | Irrelevant Topic |
| 31 | Ji HH, Zhang S, Zhang YL, et al. Research progress on the effect of bowel preparation for colonoscopy on intestinal microecology[J]. Modern Digestion & Intervention, 2024, 29(8): 986-990. doi: 10.3969/j.issn.1672-2159.2024.08.022. | Review |
| 32 | Li Q, Wang DD, Zhou Y, et al. Effect of actual medication speed of compound polyethylene glycol electrolyte powder on bowel preparation[J]. Modern Digestion & Intervention, 2021, 26(10): 1220-1224, 1229. doi: 10.3969/j.issn.1672-2159.2021.10.003. | Irrelevant Topic |
| 33 | Kuang JY, Liu JT. Research progress of pre-gastroscopy medication[J]. Modern Digestion & Intervention, 2018, 23(1): 124-126. doi: 10.3969/j.issn.1672-2159.2018.01.044. | Review |
| 34 | Cheng CS, Xue SG, Zhang HD, et al. Comparison of residual gastric fluid volume between traditional single and split bowel preparation in patients undergoing sedated colonoscopy[J]. Chinese Journal of Digestion, 2017, 37(12): 812-815. doi: 10.3760/cma.j.issn.0254-1432.2017.12.005. | Irrelevant Topic |
| 35 | Wang B, Zhuang XY, Cui XZ, et al. Effect of modified oral administration of polyethylene glycol electrolyte powder on bowel cleanliness[J]. Nursing and Rehabilitation Journal, 2008, 7(4): 302-303. doi: 10.3969/j.issn.1671-9875.2008.04.034. | Irrelevant Topic |
| 36 | Cong DM, Wu DJ, Luo C. Comparison of four bowel cleaning methods for colonoscopy and treatment and influencing factors of ineffective bowel preparation[J]. Practical Clinical Medicine, 2010, 11(5): 21-23. doi: 10.3969/j.issn.1009-8194.2010.05.010. | Irrelevant Topic |
| 37 | Li EC, Shao SD, Hu YR. Effect of diversified management mode on improving bowel preparation quality in patients undergoing colonoscopy[J]. Chongqing Medicine, 2024, 53(20): 3060-3064. doi: 10.3969/j.issn.1671-8348.2024.20.004. | Irrelevant Topic |
| 38 | Sun WH. Preparation and scanning method of CT virtual colonoscopy with air insufflation[J]. Modern Medicine, 2003, 31(2): 125-126. doi: 10.3969/j.issn.1671-7562.2003.02.025. | Irrelevant Topic |
| 39 | Ding C, Zhou YF, Wang X, et al. Analysis of factors related to missed diagnosis of multiple colorectal polyps[J]. Zhejiang Clinical Medical Journal, 2023, 25(11): 1676-1678. | Irrelevant Topic |
| 40 | Ding MZ, Lin YF, Hu PX, et al. Influencing factors of complete relief time of abdominal distension after painless colonoscopy[J]. Health Medicine Research and Practice, 2021, 18(5): 63-66, 75. doi: 10.11986/j.issn.1673-873X.2021.05.013. | Irrelevant Topic |
| 41 | Qin SW, Xie LY, Liu L. Research status of correlation analysis between bowel preparation quality before colonoscopy and clinical laboratory indicators[J]. World Latest Medicine Information (Continuous Electronic Journal), 2024, 24(14): 52-56, 65. doi: 10.3969/j.issn.1671-3141.2024.014.010. | Irrelevant Topic |
| 42 | Ding YY. Effect of different bowel preparation regimens guided by Bristol Stool Form Scale[D]. Anhui: Anhui Medical University, 2018. doi: 10.7666/d.D01503481. | Irrelevant Topic |
| 43 | Wang WJ, Tan LL, Zeng XE, et al. A qualitative investigation of influencing factors of inpatient participation in bowel preparation[J]. Modern Health (First Half), 2022, 22(3): 394-397. doi: 10.3969/j.issn.1671-0223(s).2022.03.026. | Irrelevant Topic |
| 44 | Nie XS, Xiong DS, Xie Q, et al. Missed diagnosis of colorectal polyps under continuous high-quality colonoscopy[J]. Anhui Medical and Pharmaceutical Journal, 2015(5): 961-963. doi: 10.3969/j.issn.1009-6469.2015.05.047. | Irrelevant Topic |
| 45 | Dong HC, Chen L. How to prepare the bowel before electronic colonoscopy[J]. Health Home, 2024(2): 68-69. | Irrelevant Topic |
| 46 | Diao Y, Zong Y, Zhou YH, et al. Effect of WeChat push re-education on bowel preparation quality for colonoscopy[J]. Chinese Journal of Gastroenterology, 2025, 30(12): 655-662. doi: 10.3969/j.issn.1008-7125.2025.12.002. | Irrelevant Topic |
| 47 | Jiang LY, Zhang KW, Zhou F, et al. Value of repeat colonoscopy during colorectal polypectomy in reducing polyp miss rate[J]. Chinese Journal of Gastroenterology, 2023, 28(8): 462-466. doi: 10.3969/j.issn.1008-7125.2023.08.003. | Irrelevant Topic |
| 48 | Liu CQ, Zheng Y, Sun XM, et al. Risk factors for difficult unsedated colonoscopy[J]. Chinese Journal of Gastroenterology, 2020, 25(1): 13-17. doi: 10.3969/j.issn.1008-7125.2020.01.003. | Irrelevant Topic |
| 49 | Jiang M, Tian PY, Li HQ, et al. Comparison of the cleansing efficacy and tolerability of split-dose versus single-dose oral polyethylene glycol electrolyte solution for bowel preparation. Gastroenterology. 2014;(12):712–715. doi: 10.3969/j.issn.1008-7125.2014.12.002. | Irrelevant Topic |
| 50 | Jiang QF, Li WS. Univariate analysis of factors associated with colonoscopy detection rate. Chinese Community Doctors. 2020;36(25):105–106. doi: 10.3969/j.issn.1007-614x.2020.25.052. | Irrelevant Topic |
| 51 | Wang JP, Lin WL, Zou YL, et al. Analysis of factors associated with colorectal polyp detection after repeated insertion during colonoscopy. China Journal of Endoscopy. 2018;24(1):45–49. doi: 10.3969/j.issn.1007-1989.2018.01.009. | Irrelevant Topic |
| 52 | Liu NZ, Liu SH, Luo HY. Experience with the application of cisapride in colonoscopy. Journal of Internal Intensive Medicine. 2001;7(3):171. doi: 10.3969/j.issn.1007-1024.2001.03.026. | Irrelevant Topic |
| 53 | Lin XH, Chen GX, Zeng CY, et al. Application of the KTA evidence-based practice model in bowel preparation before colonoscopy in elderly patients. Journal of Qilu Nursing. 2022;28(6):124–126. doi: 10.3969/j.issn.1006-7256.2022.06.041. | Irrelevant Topic |
| 54 | Li TY, Yu ZF, Shang LQ. Research progress in nursing methods for bowel preparation before colonoscopy in elderly patients. Journal of Qilu Nursing. 2019;25(12):101–104. doi: 10.3969/j.issn.1006-7256.2019.12.039. | Review |
| 55 | Liu ZS, Jin SZ, Yang NN, et al. Basic quality-control factors affecting colonoscopy. Chinese Journal of Gastroenterology and Hepatology. 2019;28(9):1038–1041. doi: 10.3969/j.issn.1006-5709.2019.09.016. | Irrelevant Topic |
| 56 | Gao F, Li FX, Cui JJ. Factors associated with colorectal polyp detection in patients undergoing repeated colonoscopy. Journal of Medical Forum. 2021;42(9):104–107. | Irrelevant Topic |
| 57 | Liu D, Yao P. Current status of research on the effects of diet on bowel preparation before colonoscopy. Clinical Focus. 2019;34(1):85–87. doi: 10.3969/j.issn.1004-583X.2019.01.013. | Irrelevant Topic |
| 58 | Qin YY. Application of the knowledge-behavior model-based evidence-based practice model combined with focused psychological nursing in bowel preparation before colonoscopy. Henan Medical Research. 2021;30(28):5358–5361. doi: 10.3969/j.issn.1004-437X.2021.28.057. | Irrelevant Topic |
| 59 | Li XL, Wen S. Research progress on compound polyethylene glycol electrolyte powder combined with dietary intervention for bowel preparation before colonoscopy. Medical Journal of the Chinese People’s Armed Police Forces. 2020;31(2):163–167. doi: 10.3969/j.issn.1004-3594.2020.02.020. | Irrelevant Topic |
| 60 | Ren W, Wang C, Zhang W, et al. Effects of different bowel preparation methods on colonoscopy outcomes in elderly patients. Practical Geriatrics. 2017;31(9):888–890. doi: 10.3969/j.issn.1003-9198.2017.09.026. | Irrelevant Topic |
| 61 | Gu J, Zhu YY, Zou JW, et al. Differences in bowel preparation quality among patients of different age groups undergoing colonoscopy diagnosis and treatment and improvement measures. Modern Digestion & Intervention. 2024;29(6):653–659. doi: 10.3969/j.issn.1672-2159.2024.06.002. | Irrelevant Topic |
| 62 | Li JX. Factors influencing adenoma detection rate during colonoscopy. Medical Equipment. 2019;32(6):197–198. doi: 10.3969/j.issn.1002-2376.2019.06.128. | Irrelevant Topic |
| 63 | Wei JH, Wei QF, An D, et al. Compliance with colonoscopy screening among high-risk populations for colorectal cancer in Nanchang. Journal of Gannan Medical University. 2019;39(11):1137–1140,1148. doi: 10.3969/j.issn.1001-5779.2019.11.013. | Irrelevant Topic |
| 64 | Li D, Guo MR, Liu HF, et al. Factors influencing the quality of bowel preparation before colonoscopy in children. Journal of Clinical Pediatrics. 2020;38(6):438–442. doi: 10.3969/j.issn.1000-3606.2020.06.009. | The population does not match |
| 65 | Song YP, Guo YM, Lu LZ. Evaluation of the application effect of all-media health education among people undergoing painless colonoscopy for the first time. Shanxi Medical Journal. 2022;51(21):2489–2493. doi: 10.3969/j.issn.0253-9926.2022.21.028. | Irrelevant Topic |
| 66 | Wan XY, Yang L. Analysis of health education for bowel preparation in 1868 patients undergoing gastrointestinal endoscopy under intravenous anesthesia. Chinese Journal of Health Care and Medicine. 2013;15(6):500–502. doi: 10.3969/j.issn.1674-3245.2013.06.016. | Irrelevant Topic |
| 67 | Shao P, Pi Z, Zhong Y, et al. Application of the water infusion method in colonoscopy in infants and young children. Chinese Journal of Gastrointestinal Endoscopy (Electronic Edition). 2022;9(1):25–29. doi: 10.3877/cma.j.issn.2095-7157.2022.01.017. | Irrelevant Topic |
| 68 | Ding YM, Bai P, Zhang L, et al. Factors influencing the detection rates of colorectal polyps and adenomas during colonoscopy. Chinese Journal of Clinicians (Electronic Edition). 2022;16(10):989–994. doi: 10.3877/cma.j.issn.1674-0785.2022.10.012. | Irrelevant Topic |
| 69 | Sun C, Wang C, He WY, et al. Research progress on the effect of administration time management of compound polyethylene glycol electrolyte powder on bowel preparation. Journal of Nursing Science. 2018;33(12):95–98. doi: 10.3870/j.issn.1001-4152.2018.12.095. | Review |
| 70 | He H. Diagnostic effect and influencing factors of emergency colonoscopy in patients with acute massive lower gastrointestinal bleeding. Journal of Kunming Medical University. 2018;39(5):100–105. doi: 10.3969/j.issn.1003-4706.2018.05.020. | Irrelevant Topic |
| 71 | Li M, Lu XP, Bing WW, et al. Development and evaluation of a risk prediction model for hypoglycemia in patients undergoing colonoscopy. Chinese Journal of Nursing. 2024;59(1):64–70. doi: 10.3761/j.issn.0254-1769.2024.01.009. | Irrelevant Topic |
| 72 | Sun YQ, Wang WR, Men HL, et al. Research progress on the current status of bowel preparation and intervention strategies in elderly people undergoing colonoscopy. Chinese Journal of Modern Nursing. 2020;26(6):836–840. doi: 10.3760/cma.j.issn.1674-2907.2020.06.027. | Review |
| 73 | Hou J, Zhang YQ, Zhang LH, et al. What preparations should be made before colonoscopy. Medical Aesthetics and Cosmetology. 2015;(4):598. | Irrelevant Topic |
| 74 | Abudoukeyoumujiang Abulizi, Maihemitijiang Damola, Alimujiang Abudukeyoumu, et al. Clinical application of CT virtual colonoscopy in colorectal cancer. Medical Innovation of China. 2019;16(7):133–136. doi: 10.3969/j.issn.1674-4985.2019.07.034. | Irrelevant Topic |
| 75 | Li SH, Zhou HM, Zhang AQ, et al. Analysis of factors associated with missed and misdiagnosed cases during electronic gastroscopy. China Medicine. 2010;5(4):330–332. doi: 10.3760/cma.j.issn.1673-4777.2010.04.020. | Irrelevant Topic |
| 76 | Hu XP, Xie J, Yang J, et al. Analysis of factors associated with ineffective bowel preparation using polyethylene glycol electrolyte powder. China Journal of Endoscopy. 2015;21(8):861–863. | Irrelevant Topic |
| 77 | Hu XD. How to perform bowel preparation for colonoscopy. Health Care and Life. 2020;(18):17. | Irrelevant Topic |
| 78 | Li FJ. Effect of nursing intervention on bowel cleanliness before colonoscopy in hospitalized patients. Chinese Journal of Practical Nursing. 2011;27(7):25–26. doi: 10.3760/cma.j.issn.1672-7088.2011.07.010. | Irrelevant Topic |
| 79 | Li W. Nursing care of 85 cases undergoing capsule endoscopy. Chinese Journal of Practical Nursing. 2005;21(2):19. doi: 10.3760/cma.j.issn.1672-7088.2005.02.010. | Irrelevant Topic |
| 80 | Wei J, Du JZ, Liu LH, et al. Analysis of factors influencing bowel cleansing quality after oral compound polyethylene glycol electrolyte powder. Chinese Journal of Digestive Endoscopy. 2019;36(3):204–207. doi: 10.3760/cma.j.issn.1007-5232.2019.03.011. | Irrelevant Topic |
| 81 | Huang H, Cai Y, Chen WJ, et al. Research progress on oral medications for bowel preparation before colonoscopy in patients with chronic constipation. New Medicine. 2024;55(4):255–259. doi: 10.3969/j.issn.0253-9802.2024.04.003. | Review |
| 82 | Jiang YX, Chen Y, Li X, et al. Factors influencing polyp and adenoma detection rates during colonoscopy in high-risk patients undergoing colorectal cancer screening. Chinese Journal of Digestive Endoscopy. 2015;32(10):649–652. doi: 10.3760/cma.j.issn.1007-5232.2015.10.001. | Irrelevant Topic |
| 83 | Ji DN, Xiang P, Huang RX, et al. Effect of cap-assisted colonoscopy on colorectal adenoma detection rate. Chinese Journal of Digestive Endoscopy. 2015;32(7):444–447. doi: 10.3760/cma.j.issn.1007-5232.2015.07.006. | Irrelevant Topic |
| 84 | Huang MJ, Zhang Q, He HX, et al. Consistency between the Boston Bowel Preparation Scale and visual stool observation before colonoscopy. Modern Clinical Nursing. 2023;22(10):53–57. doi: 10.3969/j.issn.1671-8283.2023.10.009. | Irrelevant Topic |
| 85 | Li JX. Factors influencing adenoma detection rate during colonoscopy. Medical Equipment. 2019;32(6):197–198. doi: 10.3969/j.issn.1002-2376.2019.06.128. | Irrelevant Topic |
| 86 | Huang MQ, Han D, Yang F, et al. Analysis of factors influencing bowel preparation quality before outpatient colonoscopy under a pharmaceutical care model. China Medical Herald. 2021;18(32):58–61. | Irrelevant Topic |
| 87 | Huang QX, Wen XY, Jiang SJ, et al. Factors influencing compliance with follow-up colonoscopy after colon polypectomy. Doctor. 2022;7(7):85–87. | Irrelevant Topic |
| 88 | Huang RW, Jia L, Sun YC, et al. Research progress on lactulose combination regimens for bowel preparation. International Medicine and Health Guidance News. 2024;30(19):3211–3214. doi: 10.3760/cma.j.issn.1007-1245.2024.19.010. | Review |
| 89 | Wang MY, Xu N. Research progress on bowel preparation quality before colonoscopy. International Medicine and Health Guidance News. 2022;28(13):1908–1911. doi: 10.3760/cma.j.issn.1007-1245.2022.13.027. | Review |
| 90 | Huang YB. Application and research progress of sodium phosphate in bowel preparation for colonoscopy. China Health Care & Nutrition. 2014;24(4):2113–2114. | Review |
| 91 | Jiang XH, Ding WQ, Luo ZG, et al. Influencing factors of bowel preparation regimens on colonoscopy quality. Chinese Journal of Digestion. 2019;39(6):384–389. doi: 10.3760/cma.j.issn.0254-1432.2019.06.008. | Irrelevant Topic |
| 92 | Leng X, Liu F, Yang ZW, et al. Clinical significance of peripheral blood methylated septin 9 gene methylation detection in the diagnosis of colorectal cancer and adenoma. Chinese Journal of Digestion. 2018;38(6):405–407. doi: 10.3760/cma.j.issn.0254-1432.2018.06.009. | Irrelevant Topic |
| 93 | Ma XL, Liu ZL, Li XL, et al. Application value of repeated withdrawal examination of the left colon in improving the detection rate of left-sided colonic adenomas. Chinese Journal of Digestive Endoscopy. 2025;42(12):974–977. doi: 10.3760/cma.j.cn321463-20250513-00502. | Irrelevant Topic |
| 94 | Ji DN, Xiang P, Zhou Y, et al. Analysis of factors associated with missed colorectal adenomas during conventional white-light endoscopy. Chinese Journal of Digestive Endoscopy. 2017;34(7):490–494. doi: 10.3760/cma.j.issn.1007-5232.2017.07.008. | Irrelevant Topic |
| 95 | Sui XY, Zhang S, Wei JH, et al. Research status of adverse reactions associated with osmotic laxatives used for bowel preparation before colonoscopy. Chinese Journal of Digestive Endoscopy. 2025;42(1):78–81. doi: 10.3760/cma.j.cn321463-20240702-00446. | Irrelevant Topic |
| 96 | Su QY, Zhang YJ, Wang ZY, et al. Analysis of risk factors associated with adverse reactions during bowel preparation. Chinese Journal of Digestive Endoscopy. 2025;42(2):115–119. doi: 10.3760/cma.j.cn321463-20240522-00117. | Irrelevant Topic |
| 97 | Wang W, Yao LW, Xiong HZ, et al. Application of deep learning-based artificial intelligence technology in bowel preparation assessment. Chinese Journal of Digestive Endoscopy. 2025;42(2):109–114. doi: 10.3760/cma.j.cn321463-20231115-00297. | Irrelevant Topic |
| 98 | Li BB, Sun R, Yang XY, et al. Current status and influencing factors of bowel preparation failure in colonoscopy populations based on different colonic segments. Chinese Journal of Digestive Endoscopy. 2023;40(7):539–544. doi: 10.3760/cma.j.cn321463-20230214-00036. | Irrelevant Topic |
| 99 | Jiang LP. Effect of taking compound polyethylene glycol electrolyte powder at different time periods before colonoscopy on bowel cleansing. Pharmacy Weekly. 2021;30(6):187,193. | Irrelevant Topic |
| 100 | Wang JX, Yao LW, Wu LL, et al. Study on an artificial intelligence-based quality assessment system for intestinal mucosal observation. Chinese Journal of Digestive Endoscopy. 2024;41(4):269–274. doi: 10.3760/cma.j.cn321463-20230116-00646. | Irrelevant Topic |
| 101 | Liang L, Zhong WL, Wang BM. Meta-analysis of the effects of dietary intervention on blood glucose during bowel preparation in patients undergoing colonoscopy. Chinese Journal of Digestive Endoscopy. 2021;38(4):310–314. doi: 10.3760/cma.j.cn321463-20200707-00880. | Irrelevant Topic |
| 102 | Li HQ, Li YK, Qi XS, et al. Efficacy and safety of linaclotide combined with polyethylene glycol for bowel preparation in patients with functional constipation. Chinese Journal of Digestion. 2024;44(9):605–612. doi: 10.3760/cma.j.cn311367-20240131-00046. | Irrelevant Topic |
| 103 | Qin LN, Gu YJ, Ji L. Risk factors and nursing countermeasures for bowel preparation failure before colon polypectomy. International Journal of Nursing. 2025;44(21):3892–3896. doi: 10.3760/cma.j.cn221370-20241213-00829. | Irrelevant Topic |
| 104 | Liu L, Zhang CH, Zhu L, et al. Best evidence summary for bowel preparation before colonoscopy in children. Chinese Journal of Modern Nursing. 2022;28(35):4939–4946. doi: 10.3760/cma.j.cn115682-20220228-00912. | Irrelevant Topic |
| 105 | Wang SS, Guo XX, Li ZK. Research progress on factors influencing bowel preparation quality in patients undergoing colonoscopy. Chinese Journal of Modern Nursing. 2021;27(14):1942–1946. doi: 10.3760/cma.j.cn115682-20200908-05276. | Review |
| 106 | Qiao YQ, Chen HL. Application of artificial intelligence in quality control of colonoscopy. Guangzhou Medical Journal. 2025;56(5):581–590. doi: 10.20223/j.cnki.1000-8535.2025.05.002. | Irrelevant Topic |
| 107 | Li TY, Chen XZ, Xie Z, et al. Effects of family accompaniment on bowel preparation in outpatients undergoing colonoscopy diagnosis and treatment. Journal of Binzhou Medical University. 2019;42(2):146–148. doi: 10.19739/j.cnki.issn1001-9510.2019.02.019. | Irrelevant Topic |
| 108 | Wang CL, Zhao ZY, Wang H, et al. Research progress on quality control of colonoscopy. Journal of Colorectal & Anal Surgery. 2021;27(6):527–530,535. doi: 10.19668/j.cnki.issn1674-0491.2021.06.001. | Review |
| 109 | Wang CL, Wang H, Zhao ZY, et al. Current status and influencing factors of polyp and cancer detection rates during colonoscopy among relatively asymptomatic populations undergoing colorectal cancer screening in a tertiary hospital in Shanghai. Journal of Colorectal & Anal Surgery. 2019;25(2):131–136. doi: 10.19668/j.cnki.issn1674-0491.2019.02.003. | Irrelevant Topic |
| 110 | Sun WR, Ye YN, Zhang YP, et al. Value of hydrogen breath test in predicting bowel preparation quality before colonoscopy. Jiangsu Medical Journal. 2016;42(23):2576–2579. | Irrelevant Topic |
| 111 | Ma R, Zhang LL, Ren M, et al. Application effect and safety analysis of walking exercise in bowel preparation for colonoscopy in patients with diabetes. Clinical Research and Practice. 2025;10(6):57–60. doi: 10.19347/j.cnki.2096-1413.202506015. | Irrelevant Topic |
| 112 | Liu ZM, Zou C, Chen T. Effect of bowel cleanliness on colonoscopic diagnosis and treatment in patients with acute lower gastrointestinal bleeding. Clinical Journal of Medical Officers. 2018;46(8):968–969. doi: 10.16680/j.1671-3826.2018.08.42. | Irrelevant Topic |
| 113 | Li H, Wu CX, Liu ZX, et al. Research progress on bowel preparation for colonoscopy: current status, influencing factors, and optimization strategies. Advances in Clinical Medicine. 2025;15(9):1506–1512. doi: 10.12677/acm.2025.1592650. | Review |
| 114 | Liu YL, Wang YP, Zhang C, et al. Analysis of risk factors for poor bowel preparation. Medical Research and War Trauma Treatment. 2024;37(11):1148–1152. doi: 10.16571/j.cnki.2097-2768.2024.11.005. | Irrelevant Topic |
| 115 | Ding ZK, Su X, Li WF. Analysis of adenoma detection rate with Pentax colonoscopy and risk factors for postoperative polyp recurrence. Shenzhen Journal of Integrated Traditional Chinese and Western Medicine. 2025;35(20):18–21. doi: 10.16458/j.cnki.1007-0893.2025.20.005. | Irrelevant Topic |
| 116 | Li LY. Analysis of factors influencing adverse events during painless gastrointestinal endoscopy. China Health Care & Nutrition. 2022;32(10):94–96. | Irrelevant Topic |
| 117 | Jiang L, Liu J, Dong C, et al. A real-world study of bowel preparation with a 2 L polyethylene glycol regimen. Sichuan Medical Journal. 2024;45(3):245–250. doi: 10.16252/j.cnki.issn1004-0501-2024.03.005. | Irrelevant Topic |
| 118 | Li P, Li YY, Li YQ. Effect of individualized bowel preparation on bowel preparation quality. Journal of Shandong University (Health Sciences). 2020;58(3):113–117. doi: 10.6040/j.issn.1671-7554.0.2019.1286. | Irrelevant Topic |
| 119 | Li Q, Sun Y, Shang YL. Research progress on the relationship between bowel preparation and intestinal microecology. China Medical Herald. 2019;16(31):42–45. | Review |
| 120 | Dong GF, Feng L, Zhang YN, et al. Risk factors for bowel preparation efficacy in patients with liver cirrhosis complicated with diabetes. Beijing Medical Journal. 2020;42(6):587–589. doi: 10.15932/j.0253-9713.2020.06.030. | The population does not match |
| 121 | Li J, Qu C, Zhao Q, et al. Current status and influencing factors of bowel preparation adequacy before colonoscopy. Guide of China Medicine. 2020;18(27):1–2. | Irrelevant Topic |
| 122 | Qian C, Xu Z. Characteristics of colorectal adenomas and factors influencing detection rate. Clinical Rational Drug Use. 2019;12(16):23–24. doi: 10.15887/j.cnki.13-1389/r.2019.16.011. | Irrelevant Topic |
| 123 | Li CY, Cai PH, Ouyang D. Effect of nursing intervention on bowel preparation before colonoscopy. China Practical Medicine. 2017;12(14):162–163. doi: 10.14163/j.cnki.11-5547/r.2017.14.092. | Irrelevant Topic |
| 124 | Lin YF, Zhao XH, Fu ZL, et al. Evaluation of bowel preparation before colonoscopy using an artificial intelligence intestinal image recognition model. Chinese Journal of Medical Imaging Technology. 2023;39(7):1034–1038. doi: 10.13929/j.issn.1003-3289.2023.07.018. | Irrelevant Topic |
| 125 | Li WL. Quality analysis of colonoscopy using adenoma detection rate as the primary evaluation indicator. Zhejiang Clinical Medical Journal. 2020;22(9):1336–1338. | Irrelevant Topic |
| 126 | Qian YQ, Weng LJ, Ye LP, et al. Application of a novel group standard to improve the qualified rate of bowel preparation for colonoscopy in outpatients. Journal of Bengbu Medical University. 2025;50(7):965–970. doi: 10.13898/j.cnki.issn.2097-5252.2025.07.023. | Irrelevant Topic |
| 127 | Nie D, Xie YZ, Liu SQ, et al. Influencing factors of bowel preparation quality before colonoscopy in children and application effect of WeChat re-education. Practical Clinical Medicine. 2021;22(5):87–89,92. doi: 10.13764/j.cnki.lcsy.2021.05.025. | Irrelevant Topic |
| 128 | Li XM, Li XL, Li J, et al. Study on insertion efficiency and influencing factors of transanal double-balloon enteroscopy in elderly patients. Practical Geriatrics. 2024;38(9):958–961. doi: 10.3969/j.issn.1003-9198.2024.09.022. | Irrelevant Topic |
| 129 | Li YL, Guan X, Dou LZ, et al. Diagnostic value of combined fecal multitarget DNA and fecal occult blood testing for early diagnosis and screening of colorectal cancer. National Medical Journal of China. 2022;102(33):2607–2613. doi: 10.3760/cma.j.cn112137-20220430-00974. | Irrelevant Topic |
| 130 | Li QY, Liu XN, Li ZJ. Application and research progress of mirabilite for bowel preparation before colonoscopy diagnosis and treatment. Journal of Practical Traditional Chinese Internal Medicine. 2022;36(12):137–139. doi: 10.13729/j.issn.1671-7813.Z20212286. | Review |
| 131 | Luo XL, Gao Q. Research progress on improving the effectiveness and safety of bowel preparation before colonoscopy in patients with diabetes. Advances in Clinical Medicine. 2022;12(8):7473–7478. doi: 10.12677/ACM.2022.1281078. | Review |
| 132 | Shi JX, Wang WJ, Zhang XL, et al. Effectiveness and safety of water-exchange colonoscopy in elderly patients. China Journal of Endoscopy. 2025;31(5):58–65. doi: 10.12235/E20240499. | Irrelevant Topic |
| 133 | Ma YJ, Yang XH, Gao HL, et al. Analysis of predictors of cecal intubation rate during colonoscopy. China Journal of Endoscopy. 2021;27(10):46–51. doi: 10.12235/E20210423. | Irrelevant Topic |
| 134 | Liang R, Hao WJ, Liu MX. A qualitative study on poor bowel preparation before colonoscopy in elderly patients. Modern Vocational Education. 2021;(23):158–159. | Irrelevant Topic |
| 135 | Liang SH. Effect of standardized nursing on bowel preparation during colonoscopy in health examination. Advances in Clinical Nursing. 2025;4(8). doi: 10.12208/j.jacn.20250413. | Irrelevant Topic |
| 136 | Lin C, Wang FR. Current status and influencing factors of hypoglycemia in patients undergoing colonoscopy. Chinese Evidence-Based Nursing. 2025;11(17):3658–3662. doi: 10.12102/j.issn.2095-8668.2025.17.032. | Irrelevant Topic |
| 137 | Ding M, Zhang M, Li JJ, et al. Scoping review of evidence on influencing factors of bowel preparation before colonoscopy diagnosis and treatment. Chinese Evidence-Based Nursing. 2025;11(6):1059–1064. doi: 10.12102/j.issn.2095-8668.2025.06.007. | Irrelevant Topic |
| 138 | Pi JF, Lu LL, Guo F. Best evidence summary of chewing gum during bowel preparation in patients undergoing colonoscopy. Chinese Evidence-Based Nursing. 2023;9(1):44–49. doi: 10.12102/j.issn.2095-8668.2023.01.007. | Irrelevant Topic |
| 139 | Wang TT, You LL. Meta-analysis of factors influencing bowel preparation quality before colonoscopy. Chinese Nursing Research. 2021;35(2):223–230. doi: 10.12102/j.issn.1009-6493.2021.02.006. | Review |
| 140 | Su D, Kang Y, Li X. Compliance with colonoscopy and influencing factors among 1075 individuals with positive preliminary colorectal cancer screening results. Chinese Journal of the Frontiers of Medical Science (Electronic Version). 2021;13(7):83–88. doi: 10.12037/YXQY.2021.07-12. | Irrelevant Topic |
| 141 | Tu HL, Ge YG, Li XP. Investigation of the effective rate of bowel preparation for colonoscopy and related influencing factors. Chinese Remedies & Clinics. 2019;19(12):1988–1990. doi: 10.11655/zgywylc2019.12.014. | Irrelevant Topic |
| 142 | Wang JZ, Zhang Y, Guo Q. Reflections on high-quality colonoscopy. World Chinese Journal of Digestology. 2021;29(17):977–983. doi: 10.11569/wcjd.v29.i17.977. | Irrelevant Topic |
| 143 | Liu HR, Yang YL. Influencing factors and solutions for difficult colonoscopy. Laboratory Medicine and Clinic. 2016;13(16):2393–2395. doi: 10.3969/j.issn.1672-9455.2016.16.065. | Irrelevant Topic |
| 144 | Liu HX, Chen YH. Application of quality control circle to explore the causes of inadequate bowel preparation and nursing countermeasures. Home Medicine. 2018;(11):263. doi: 10.3969/j.issn.1671-4954.2018.11.343. | Irrelevant Topic |
| 145 | Nie D, Xie YZ, Liu SQ, et al. Influencing factors of bowel preparation quality before colonoscopy in children and application effect of WeChat re-education. Practical Clinical Medicine. 2021;22(5):87–89,92. doi: 10.13764/j.cnki.lcsy.2021.05.025. | Irrelevant Topic |
| 146 | Zueco CZ, Sampedro CS, Corroto JD, Fernández PR, Fontanillo MF. CT colonography without cathartic preparation: positive predictive value and patient experience in clinical practice. European Radiology. 2012;22(6):1195-1204. doi: 10.1007/s00330-011-2367-0. | Irrelevant Topic |
| 147 | Wang X, Zhu XQ. Research progress on comfort in patients undergoing bowel preparation. World Chinese Journal of Digestology. 2018;26(17):1015–1021. doi: 10.11569/wcjd.v26.i17.1015. | Review |
| 148 | Liu XH, Wang XQ, Cai YL. Research progress on factors influencing bowel preparation quality and nursing intervention strategies in elderly patients undergoing colonoscopy. Chinese General Practice Nursing. 2024;22(2):257–261. doi: 10.12104/j.issn.1674-4748.2024.02.012. | Review |
| 149 | Liu XQ. Research progress on bowel preparation before colonoscopy in patients with diabetes. Journal of Colorectal & Anal Surgery. 2014;20(3):221–222. | Review |
| 150 | Liu XP, Zhao WY, Cui JY, et al. Effects of enteral nutrition combined with compound polyethylene glycol electrolyte powder on bowel preparation quality in populations with different risk stratifications. Chinese Journal of Clinical Gastroenterology. 2025;37(1):35–38. doi: 10.3870/lcxh.j.issn.1005-541X.2025.01.008. | Irrelevant Topic |
| 151 | Gao DM, Liu ZH, Chen YW. Current status and influencing factors of knowledge, attitude, and practice regarding bowel preparation before colonoscopy among nurses in geriatric medicine departments. Journal of Clinical Medicine in Practice. 2022;26(22):119–122. doi: 10.7619/jcmp.20222211. | Irrelevant Topic |
| 152 | Chen XY, Chen F, Yang LQ, et al. Risk factors associated with pain during colonoscopy. Journal of Clinical and Pathological Research. 2019;39(3):570–575. doi: 10.3978/j.issn.2095-6959.2019.03.018. | Irrelevant Topic |
| 153 | Zhao M. Detection rate of colonoscopy in colonic adenoma and analysis of related factors affecting examination results. Kangyi. 2025;(23):163–165. doi: 10.3969/j.issn.2095-6525.2025.23.070. | Irrelevant Topic |
| 154 | Yin J. Clinical observation and nursing of colonoscopy in patients with constipation. Journal of Frontiers of Medicine. 2014;(27):215–216. doi: 10.3969/j.issn.2095-1752.2014.27.207. | Irrelevant Topic |
| 155 | Lou QL, Gao F, Zhang M. Multivariate logistic regression analysis of bowel preparation with compound polyethylene glycol electrolyte powder. Chinese Journal of Clinicians (Electronic Edition). 2017;(9):1505–1508. | Irrelevant Topic |
| 156 | Luo WC. Application value of narrow-band imaging technology in the diagnosis of early colorectal cancer and precancerous lesions. World Latest Medicine Information. 2015;15(76):10–12. doi: 10.3969/j.issn.1671-3141.2015.76.007. | Irrelevant Topic |
| 157 | Zhao FY. Effect of defecation before bowel preparation on the efficacy of different bowel preparation methods. Medical Journal of North China National Defense. 2005;17(5):332–333. doi: 10.3969/j.issn.2095-140X.2005.05.013. | Irrelevant Topic |
| 158 | Lu J, Duan GY, Guo YN, et al. Clinical study on screening high-risk populations for early colorectal cancer in a certain region. China Health Care & Nutrition. 2023;33(20):28–30. | Irrelevant Topic |
| 159 | Gao Y, Zhang BB. Discussion on related factors affecting bowel cleansing quality before colonoscopy. China Health Standard Management. 2023;14(15):83–87. doi: 10.3969/j.issn.1674-9316.2023.15.018. | Irrelevant Topic |
| 160 | Ma ZL, Li T, Zhang PL, et al. Analysis of polyp detection rate in cap-assisted single-operator colonoscopy. Yunnan Medical Journal. 2018;39(1):41–43. | Irrelevant Topic |
| 161 | Ma GR, Ruan CF, Zhao XX, et al. Scoping review of prediction models for inadequate bowel preparation in adults undergoing colonoscopy. Chinese Journal of Modern Nursing. 2025;31(11):1520–1528. doi: 10.3760/cma.j.cn115682-20240116-00200-1. | Review |
| 162 | Han YD, Miao J, Zhou Y. Clinical value of narrow-band imaging endoscopy in the diagnosis of early colorectal cancer. Modern Digestion & Intervention. 2016;21(4):583–585. doi: 10.3969/j.issn.1672-2159.2016.04.021. | Irrelevant Topic |
| 163 | Yang SB, Guo X. Clinical value of narrow-band imaging endoscopy in the diagnosis of early colorectal cancer. China Continuing Medical Education. 2017;9(30):42–44. doi: 10.3969/j.issn.1674-9308.2017.30.024. | Review |
| 164 | Cao H, Gui Y. Influencing factors of bowel preparation quality in patients undergoing colonoscopy in an endoscopy center. China Modern Medicine. 2025;32(13):152–155. doi: 10.3969/j.issn.1674-4721.2025.13.34. | Irrelevant Topic |
| 165 | Yang WF. Application of colonic lavage and oral 20% mannitol for bowel preparation in colonoscopy. Chinese Journal of Clinical Rational Drug Use. 2009;2(18):89. doi: 10.3969/j.issn.1674-3296.2009.18.075. | The population does not match |
| 166 | Ma YL, Zhang F. Influencing factors of bowel preparation before colonoscopy in children. Journal of Practical Clinical Nursing Science. 2020;5(10):25. | Irrelevant Topic |
| 167 | Gao YX, Wang ZN, Yao Y, et al. Effects of different medication and health education methods on bowel preparation compliance before colonoscopy in elderly patients. Chinese Journal of Health Care and Medicine. 2021;23(4):412–414. doi: 10.3969/j.issn.1674-3245.2021.04.030. | Irrelevant Topic |
| 168 | Mao YH, Wang YR, Zhang Y. Correlation analysis between different bowel preparation regimens and the success rate of colonoscopy preparation. Tibetan Medicine. 2025;46(2):47–49. | Irrelevant Topic |
| 169 | Wang ZM. Effect analysis of bowel cleansing methods before colonoscopy [dissertation]. Henan: Zhengzhou University; 2014. | Irrelevant Topic |
| 170 | Ni Y, Shen ZX. Cause analysis and countermeasures for ineffective bowel preparation in patients undergoing colonoscopy. Chinese Manipulation & Rehabilitation Medicine. 2011;(3):96. | Irrelevant Topic |
| 171 | Pan XM. Effect of Bristol stool form assessment on bowel preparation quality for colonoscopy. Today Nurse. 2019;26(10):120–121. | Irrelevant Topic |
| 172 | Han Q, Tu HM. Analysis of bowel preparation cleanliness compliance and related influencing factors in patients undergoing gastrointestinal endoscopy. Chinese Medical Record. 2025;26(12):103–106. doi: 10.3969/j.issn.1672-2566.2025.12.031. | Irrelevant Topic |
| 173 | Qi GF. Analysis of bowel cleansing effect and influencing factors before colonoscopy. Guide of China Medicine. 2019;17(16):124–125. | Irrelevant Topic |
| 174 | Han XQ, Zhang Y. Observation of influencing factors of oral mannitol for bowel preparation in elderly patients. Journal of Clinical Medicine in Practice. 2011;15(6):61–62. doi: 10.3969/j.issn.1672-2353.2011.06.026. | Irrelevant Topic |
| 175 | Qian CG, Yang QF. Analysis of qualified bowel preparation rate and influencing factors before colonoscopy in patients with chronic constipation. Medical Diet and Health. 2021;19(17):51–52,54. | The population does not match |
| 176 | Zhang YF, Xue XQ, Zhang DQ, et al. Analysis of abdominal distension and influencing factors in outpatients undergoing gastrointestinal examination. Modern Digestion & Intervention. 2025;30(1):67–70. doi: 10.3969/j.issn.1672-2159.2025.01.013. | Irrelevant Topic |
| 177 | Zhang SE, Wu JW, Lu ZW. Influencing factors of bowel preparation quality and their differential effects on segmental bowel preparation quality. Modern Digestion & Intervention. 2023;28(2):145–150,155. doi: 10.3969/j.issn.1672-2159.2023.02.002. | Irrelevant Topic |
| 178 | Huang KZ, Xiang L. Research progress on factors related to colonoscopy screening quality. Modern Digestion & Intervention. 2022;27(2):237–240. doi: 10.3969/j.issn.1672-2159.2022.02.024. | Review |
| 179 | Yuan XG, Xue J, Xiang L, et al. Analysis of controllable influencing factors for endoscopic detection of colorectal flat adenomas. Modern Digestion & Intervention. 2017;22(5):631–634. doi: 10.3969/j.issn.1672-2159.2017.05.004. | Irrelevant Topic |
| 180 | Qin XJ, Lan YT. Study on factors related to missed colorectal adenomas during colonoscopy. Health Literature. 2022;23(5):161–162. | Irrelevant Topic |
| 181 | Yin HF, Huang LH. Research progress on assessment tools and influencing factors for inadequate bowel preparation before colonoscopy. Nursing and Rehabilitation. 2023;22(9):98–102. doi: 10.3969/j.issn.1671-9875.2023.09.023. | Review |
| 182 | He HM, Cao LY. Analysis of causes affecting the bowel preparation effect of compound polyethylene glycol electrolyte powder in hospitalized patients. Chongqing Medicine. 2013;(21):2564–2565. doi: 10.3969/j.issn.1671-8348.2013.21.053. | Irrelevant Topic |
| 183 | Zhang YY, Niu ME. Research progress on risk factors for inadequate bowel preparation in patients undergoing colonoscopy. Modern Clinical Nursing. 2020;19(6):67–72. doi: 10.3969/j.issn.1671-8283.2020.06.012. | Review |
| 184 | She FH, Zeng JL, Tan NF. Study on related factors affecting bowel cleanliness before electronic colonoscopy. Journal of Practical Clinical Nursing Science. 2020;5(36):93,98. | Irrelevant Topic |
| 185 | Wu DD, Zhang LX, Yu Y. Analysis of factors influencing abdominal pain during conventional colonoscopy. Journal of Anhui Health Vocational & Technical College. 2020;19(1):136–137,139. doi: 10.3969/j.issn.1671-8054.2020.01.064. | Irrelevant Topic |
| 186 | Shi ZY. Exploration of influencing factors and nursing care for inadequate bowel preparation before colonoscopy in patients with chronic constipation. Chinese Science and Technology Journal Database (Full-text Edition): Medicine and Health. 2023;(10) [cited 2023 Oct 1]. Available from: https://www.cqvip.com/doc/journal/1000003869136. | The population does not match |
| 187 | Zhang HL, Liu HJ, Zhao HT, et al. Blood glucose management during bowel preparation for colonoscopy in patients with diabetes. World Latest Medicine Information. 2023;23(35):77–81. doi: 10.3969/j.issn.1671-3141.2023.35.015. | Irrelevant Topic |
| 188 | Song JF, Jin P, Yu JW, et al. Analysis of risk factors associated with missed colorectal adenomas during colonoscopy. Chinese Journal of Digestive Endoscopy. 2016;33(3):145–150. doi: 10.3760/cma.j.issn.1007-5232.2016.03.004. | Irrelevant Topic |
| 189 | Zhao LH, Pu JF, Wu YL, et al. Investigation and analysis of factors influencing bowel cleanliness in patients undergoing colonoscopy. World Latest Medicine Information. 2020;20(57):175–176,182. doi: 10.3969/j.issn.1671-3141.2020.57.094. | Irrelevant Topic |
| 190 | Ge ZZ, Li ZT, Zhao YY, et al. Research progress on factors influencing the quality of bowel preparation with compound polyethylene glycol electrolyte powder. World Latest Medicine Information. 2020;20(50):98–100. doi: 10.3969/j.issn.1671-3141.2020.50.038. | Review |
| 191 | Su HB, Sun LM, Wei N, et al. Research progress on bowel preparation methods before colonoscopy in patients with constipation. Special Health. 2019;(24):224. | Review |
| 192 | Huang JY, Zeng JD, Hou XY. Investigation of influencing factors leading to ineffective bowel preparation for colonoscopy. World Latest Medicine Information. 2020;20(31):46–47. doi: 10.3969/j.issn.1671-3141.2020.31.024. | Irrelevant Topic |
| 193 | Huang CW, Zhu FC, Pan D. Study on factors influencing willingness for repeat examination in patients with inadequate bowel preparation. Modern Practical Medicine. 2017;29(9):1160–1162. doi: 10.3969/j.issn.1671-0800.2017.09.019. | Irrelevant Topic |
| 194 | Wu X. Influencing factors and solutions for difficult colonoscopy. Modern Health Preservation. 2017;(4):45–46. doi: 10.3969/j.issn.1671-0223.2017.04.034. | Irrelevant Topic |
| 195 | Yang F, Huang MQ, Chen YH, et al. Practice of pharmaceutical care for bowel preparation in outpatients undergoing colonoscopy. Anhui Medical and Pharmaceutical Journal. 2020;24(9):1875–1879. doi: 10.3969/j.issn.1009-6469.2020.09.046. | Irrelevant Topic |
| 196 | Yang L, Zhang JQ, Yue PP, et al. Construction and performance evaluation of a nomogram prediction model for factors influencing missed colon polyps. Journal of Xinjiang Medical University. 2025;48(2):210–214,228. doi: 10.3969/j.issn.1009-5551.2025.02.014. | Irrelevant Topic |
| 197 | Cai WZ, Zhi FC, Li FL, et al. Factors influencing the ineffective rate of bowel preparation for colonoscopy. World Chinese Journal of Digestology. 2005;13(14):1785–1787. doi: 10.3969/j.issn.1009-3079.2005.14.036. | Irrelevant Topic |
| 198 | Han XQ, Zhang Y. Effect observation of improved oral mannitol for bowel preparation in elderly patients. Zhejiang Clinical Medical Journal. 2011;13(4):399–400. doi: 10.3969/j.issn.1008-7664.2011.04.016. | Irrelevant Topic |
| 199 | Zhang W, Dai J. Retrospective study of colonoscopy withdrawal time and colorectal polyp detection rate. Gastroenterology. 2020;(7):405–408. doi: 10.3969/j.issn.1008-7125.2020.07.005. | Irrelevant Topic |
| 200 | Wu R, Wang J, Ji WY, et al. Influencing factors and control of colonoscopy quality. Gastroenterology. 2019;24(12):758–761. doi: 10.3969/j.issn.1008-7125.2019.12.011. | Irrelevant Topic |
| 201 | Zhang WH, Lao JM, Wu XP, et al. Research progress on nursing interventions for bowel preparation quality before colonoscopy in patients with diabetes. Chinese Community Doctors. 2024;40(5):5–7. doi: 10.3969/j.issn.1007-614x.2024.05.003. | Review |
| 202 | Fan ZL, Wang YP. Effect observation of magnesium sulfate mixture for bowel preparation before colonoscopy. Chinese Community Doctors. 2012;14(6):321–322. doi: 10.3969/j.issn.1007-614x.2012.06.318. | Irrelevant Topic |
| 203 | Han MY, Li CF, Zhang H. Comparison of the effects of different bowel preparation regimens and transparent caps on colonoscopy quality. Chinese Journal of Laboratory Diagnosis. 2021;25(7):1035–1039. doi: 10.3969/j.issn.1007-4287.2021.07.026. | Irrelevant Topic |
| 204 | Tang ML. Eat fewer residue-producing foods before colonoscopy. Health Digest. 2024;(7):55. | Irrelevant Topic |
| 205 | Tong Y, Zuo GQ, Mei ZC. Influencing factors of bowel preparation quality before colonoscopy in special populations. Advances in Clinical Medicine. 2023;13(11):17129–17138. doi: 10.12677/ACM.2023.13112401. | The population does not match |
| 206 | Gao T, Cao JR, Liu YY, et al. Effect of an intensified intervention program based on the Dik VK model on bowel preparation quality in hospitalized patients. Journal of Qilu Nursing. 2023;29(24):85–88. doi: 10.3969/j.issn.1006-7256.2023.24.026. | Irrelevant Topic |
| 207 | Gu HZ, Zheng ZM, Liu LY, et al. Preparation and nursing care before spiral CT colonography. Today Nurse. 2006;(9):12. doi: 10.3969/j.issn.1006-6411.2006.09.008. | Irrelevant Topic |
| 208 | Chen XY, Wang C, Nie Y. Construction of prediction models for bowel preparation quality and minor adverse events in colonoscopy diagnosis and treatment. Journal of Practical Medicine. 2025;41(19):3016–3025. doi: 10.3969/j.issn.1006-5725.2025.19.009. | Irrelevant Topic |
| 209 | Zhou JW, Xu ZD, Yang DY, et al. Comparative study of bowel preparation with written instruction combined with telephone education versus written instruction alone. Chinese Journal of Gastroenterology and Hepatology. 2024;33(9):1167–1171. doi: 10.3969/j.issn.1006-5709.2024.09.011. | Irrelevant Topic |
| 210 | Zhao YG, Zhao L, Qin M, et al. Effects of psychological factors on bowel preparation quality before colonoscopy. Chinese Journal of Gastroenterology and Hepatology. 2022;31(4):428–433. doi: 10.3969/j.issn.1006-5709.2022.04.015. | Irrelevant Topic |
| 211 | Wang CL. Construction of a nomogram model for hypoglycemia in patients undergoing colonoscopy. Modern Scientific Instruments. 2024;41(6):195–199. | Irrelevant Topic |
| 212 | Xie L, Wu YL, Chen P, et al. Characteristics of 835 colorectal advanced adenomas and analysis of causes of missed diagnosis. Chinese Journal of Gastroenterology and Hepatology. 2022;31(1):40–45. doi: 10.3969/j.issn.1006-5709.2022.01.009. | Irrelevant Topic |
| 213 | Yang TT, Han SY, Yang YX. Application of prucalopride in bowel preparation before colonoscopy in patients with chronic constipation. Chinese Journal of Gastroenterology and Hepatology. 2018;27(1):100–104. doi: 10.3969/j.issn.1006-5709.2018.01.023. | Irrelevant Topic |
| 214 | Wang HL, Zhang L. Comparative study of the effects of video education and conventional education on bowel preparation quality. Journal of Diseases Monitor & Control. 2019;13(2):104–109. | Irrelevant Topic |
| 215 | Wang JJ, Zhao R, Xiao SL, et al. Application of process management in improving bowel preparation quality before colonoscopy. Journal of Practical Clinical Nursing Science. 2022;7(36):24–26. | Irrelevant Topic |
| 216 | Fu KD. Observation of colonoscopic detection rate of colorectal adenomas and analysis of influencing factors. Medical Information. 2024;37(23):98–101. doi: 10.3969/j.issn.1006-1959.2024.23.023. | Irrelevant Topic |
| 217 | Zhang JF, Song L, Liu N, et al. Effect analysis of capsule endoscopy in colon examination. Journal of Changzhi Medical College. 2013;27(2):135–137. | Irrelevant Topic |
| 218 | Yang SP, Li ZT, Xu LD, et al. Patient-related factors influencing bowel preparation quality. Chinese Journal of Gerontology. 2018;38(22):5469–5471. doi: 10.3969/j.issn.1005-9202.2018.22.038. | Irrelevant Topic |
| 219 | Tong Y, Zuo GQ, Mei ZC. Influencing factors of bowel preparation quality before colonoscopy in special populations. Advances in Clinical Medicine. 2023;13(11):17129–17138. doi: 10.12677/ACM.2023.13112401. | Irrelevant Topic |
| 220 | Chen J, Zhang ZH, Wang GH, et al. Establishment of an artificial intelligence-assisted system for colonoscopy quality control based on different deep learning architectures. Chinese Journal of Medical Physics. 2024;41(11):1443–1452. doi: 10.3969/j.issn.1005-202X.2024.11.018. | Irrelevant Topic |
| 221 | Yang LT, Zhao DZ. Commonly used clinical bowel cleansing methods and their evaluation. Clinical Focus. 2008;23(14):1057–1059. doi: 10.3969/j.issn.1004-583X.2008.14.042. | Irrelevant Topic |
| 222 | Zhao YZ, Wu MJ, Wang AX, et al. Influencing factors and nursing care for bowel cleansing efficacy before colonoscopy. Hebei Medical Journal. 2012;34(23):3667–3668. doi: 10.3969/j.issn.1002-7386.2012.23.085. | Irrelevant Topic |
| 223 | Yang QL, Yang TH, Shi ZY, et al. Observation of bowel preparation and nursing intervention effects in elderly patients. Journal of Nurses Training. 2007;22(6):519–520. doi: 10.3969/j.issn.1002-6975.2007.06.016. | Irrelevant Topic |
| 224 | Wang XW, Jia KM. Application of continuous quality improvement in bowel cleanliness for patients undergoing colonoscopy. Journal of Nursing Administration. 2016;16(10):743–745. | Irrelevant Topic |
| 225 | Wang Y. Analysis of factors influencing bowel cleanliness before colonoscopy in patients with chronic constipation. Medicine and Health. 2022;(5). | The population does not match |
| 226 | Zhao XT. Preoperative preparation status and related risk factors in patients undergoing colonoscopy. Medical Equipment. 2023;36(3):143–145. doi: 10.3969/j.issn.1002-2376.2023.03.047. | Irrelevant Topic |
| 227 | Wu JL. Causes of ineffective bowel preparation in patients undergoing colonoscopy and nursing countermeasures. Medical Equipment. 2019;32(20):172–173. doi: 10.3969/j.issn.1002-2376.2019.20.116. | Irrelevant Topic |
| 228 | Zhu J, Zhang XQ. Construction and validation of a LASSO regression-based risk prediction model for bowel preparation failure in children undergoing colonoscopy. Journal of Qiqihar Medical University. 2025;46(4):395–400, insert 1. doi: 10.3969/j.issn.1002-1256.2025.4.017. | The population does not match |
| 229 | Yan Y. Observation of the effects of different bowel cleansing methods. Journal of Qiqihar Medical University. 2011;32(16):2722. doi: 10.3969/j.issn.1002-1256.2011.16.105. | Irrelevant Topic |
| 230 | Wei NN, Chu Y, Li Y, et al. Analysis of bowel preparation efficacy and influencing factors before colonoscopy in patients with chronic constipation. Journal of Tongji University (Medical Science). 2020;41(4):502–506. doi: 10.16118/j.1008-0392.2020.04.018. | The population does not match |
| 231 | Wen H, Wang LH, Huang XM, et al. Construction and evaluation of a risk prediction model for hypoglycemia during colonoscopy in patients with diabetes. Modern Digestion & Intervention. 2024;29(12):1405–1407,1413. doi: 10.3969/j.issn.1672-2159.2024.12.004. | Irrelevant Topic |
| 232 | Chen HL, Tang JG, Duan YL. Application of lactulose in bowel preparation before colonoscopy in elderly patients. Journal of Clinical Internal Medicine. 2015;(3):206–207. doi: 10.3969/j.issn.1001-9057.2015.03.019. | Irrelevant Topic |
| 233 | Cao Y. How to efficiently complete gastrointestinal endoscopy. Friends of Science. 2025;(11):38–39. doi: 10.3969/j.issn.1000-8136.2025.11.018. | Irrelevant Topic |
| 234 | Xue G, Wang QH, Li Y. Analysis of factors influencing bowel preparation quality before colonoscopy. Guizhou Medical Journal. 2018;42(6):749–751. doi: 10.3969/j.issn.1000-744X.2018.06.050. | Irrelevant Topic |
| 235 | Wu ZR. Investigation and analysis of bowel cleansing efficacy and influencing factors before colonoscopy. The Road to Health. 2018;(9). | Irrelevant Topic |
| 236 | Hu P. Effect observation of single-dose versus split-dose compound polyethylene glycol electrolyte solution before colonoscopy. Shanxi Medical Journal. 2022;51(4):406–408. doi: 10.3969/j.issn.0253-9926.2022.04.012. | Irrelevant Topic |
| 237 | Chen HJ, Xie CH, Zhou CL, et al. Analysis of influencing factors for bowel cleansing efficacy using PEG-ELP combined with simethicone in hospitalized patients. New Medicine. 2023;54(6):442–446. doi: 10.3969/j.issn.0253-9802.2023.06.013. | Irrelevant Topic |
| 238 | Xiong JY, Song FQ, Kuang GB, et al. Clinical application of narrow-band imaging technology in the diagnosis and treatment of early colorectal cancer and precancerous lesions. Contemporary Medicine. 2012;18(33):154–155. | Irrelevant Topic |
| 239 | Xiong YJ, Luo HS. Analysis of factors influencing colorectal adenoma detection rate and its clinical significance. Journal of Clinical Internal Medicine. 2016;33(11):756–758. doi: 10.3969/j.issn.1001-9057.2016.11.010. | Irrelevant Topic |
| 240 | Zhang Y, Yang YL. Research progress on non-pharmacological sedation colonoscopy. New Medicine. 2015;(5):273–278. doi: 10.3969/g.issn.0253-9802.2015.05.001. | Review |
| 241 | Ye SJ, Liu Y, Ma LJ. Research progress on bowel preparation and colorectal polyp detection rate. Chinese Journal of Gastrointestinal Endoscopy (Electronic Edition). 2022;9(2):104–107. doi: 10.3877/cma.j.issn.2095-7157.2022.02.019. | Review |
| 242 | Yang D, Tao K, Li M, et al. Study on factors associated with insertion difficulty during electronic colonoscopy. Chinese Journal of Gastrointestinal Endoscopy (Electronic Edition). 2022;9(1):30–35. doi: 10.3877/cma.j.issn.2095-7157.2022.01.018. | Irrelevant Topic |
| 243 | Xu MM, Fu XR. Research progress on risk prediction of bowel preparation failure in patients undergoing colonoscopy. Chinese Evidence-Based Nursing. 2022;8(11):1469–1472. doi: 10.12102/j.issn.2095-8668.2022.11.009. | Review |
| 244 | Zhang LL, Wang QQ, Zou J, et al. Research progress on improving bowel preparation quality before colonoscopy in hospitalized patients. Chinese Journal of Clinical Gastroenterology. 2023;35(1):79–83. doi: 10.3870/lcxh.j.issn.1005-541X.2023.01.019. | Review |
| 245 | Xu T, Huang XL. Research progress on prediction models for bowel preparation quality. Medicine and Health. 2022;(9). | Review |
| 246 | Xu ZR, Zhou HZ, Qiu GC, Wang LX. Sigmoidoscopy without enema. Chinese Journal of Internal Medicine. 1961;9(2):77. | Irrelevant Topic |
| 247 | Zhang W, Lu JY, Wang D, et al. Effect of evidence-based continuous quality improvement on bowel preparation quality in elderly hospitalized patients. Chinese Journal of Geriatrics Research (Electronic Edition). 2025;12(1):35–40. doi: 10.3760/cma.j.issn.2095-8757.2025.01.007. | Irrelevant Topic |
| 248 | Zhang YY, Niu ME, Wang QY, et al. Qualitative study on factors influencing non-adherence to bowel preparation in patients undergoing colonoscopy. Chinese Journal of Modern Nursing. 2018;24(13):1535–1538. doi: 10.3760/cma.j.issn.1674-2907.2018.13.012. | Irrelevant Topic |
| 249 | Zhang HY, Li KX, Gulibahaer Simayi. Comparative study of the effects of three laxatives in bowel preparation. Chinese Journal of Modern Nursing. 2014;20(4):467–470. doi: 10.3760/cma.j.issn.1674-2907.2014.04.032. | Irrelevant Topic |
| 250 | Zou J, Wang L, Liu LX. Research progress on bowel preparation methods and nursing care in patients with chronic constipation. Chinese Journal of Modern Nursing. 2011;17(8):978–980. doi: 10.3760/cma.j.issn.1674-2907.2011.08.048. | Review |
| 251 | Yang J, Li JN, Duan AL, et al. Effect of quality control circle on the qualified rate of bowel cleanliness in hospitalized patients taking oral polyethylene glycol electrolyte powder before colonoscopy. China Medicine. 2017;12(10):1552–1556. doi: 10.3760/cma.j.issn.1673-4777.2017.10.028. | Irrelevant Topic |
| 252 | Xue P, Jia P, Wang Y, et al. Intervention effect of psychological nursing in patients undergoing colonoscopy. Journal of Practical Nursing. 2001;17(2):48–49. doi: 10.3760/cma.j.issn.1672-7088.2001.02.029. | Irrelevant Topic |
| 253 | Zhu BX. Comparison of the effects of four bowel preparation regimens before colonoscopy [dissertation]. Shandong: Qingdao University; 2017. | Irrelevant Topic |
| 254 | Yu ZW, Gu Y, Huang Y, et al. Application of the Bristol Stool Form Scale in bowel preparation before colonoscopy in children. Chinese Journal of Digestive Endoscopy. 2019;36(1):25–30. doi: 10.3760/cma.j.issn.1007-5232.2019.01.006. | Irrelevant Topic |
| 255 | Feng Y, Gao F, Lu JJ, et al. Analysis of factors associated with colorectal polyp detection rate using repeated insertion observation during colonoscopy. Chinese Journal of Digestive Endoscopy. 2018;35(7):477–481. doi: 10.3760/cma.j.issn.1007-5232.2018.07.005. | Irrelevant Topic |
| 256 | Zhang SY, Li J, Wu D, et al. Characteristics of colonoscopic perforation cases and risk factors for complications caused by surgical treatment. Chinese Journal of Digestive Endoscopy. 2018;35(7):465–469. doi: 10.3760/cma.j.issn.1007-5232.2018.07.003. | Irrelevant Topic |
| 257 | Fang J, Ma D, Wang SL, et al. Research progress on patient-related factors influencing bowel preparation quality. Chinese Journal of Digestive Endoscopy. 2016;33(4):265–267. doi: 10.3760/cma.j.issn.1007-5232.2016.04.022. | Review |
| 258 | Deng SX, Cai QC, Li ZS. Analysis and progress of factors influencing bowel preparation quality before colonoscopy. Chinese Journal of Digestive Endoscopy. 2011;28(2):118–120. doi: 10.3760/cma.j.issn.1007-5232.2011.02.026. | Irrelevant Topic |
| 259 | Gao G, Cao JB, Wang XW, et al. Observation of different bowel preparation methods before colonoscopy examination and treatment. Chinese Journal of Digestive Endoscopy. 2008;25(8):435–436. doi: 10.3760/cma.j.issn.1007-5232.2008.08.015. | Irrelevant Topic |
| 260 | Fu Y, Tian Y, Nian BX, et al. Comparison of polyethylene glycol electrolyte powder and sulfate solution in bowel preparation and colonoscopy quality. Chinese Journal of Digestive Endoscopy. 2025;42(10):803–808. doi: 10.3760/cma.j.cn321463-20250604-00228. | Irrelevant Topic |
| 261 | Zhu XJ, Wang Q, Dai HM, et al. Effect of withdrawal time on adenoma detection rate in patients with adequate bowel preparation. Chinese Journal of Digestive Endoscopy. 2025;42(12):985–991. doi: 10.3760/cma.j.cn321463-20250313-00376. | Irrelevant Topic |
| 262 | Bai X, Song ZY, Guo Q. Necessity and practical considerations of including colorectal polyp and interval cancer detection rates in routine quality control indicators for colonoscopy. Chinese Journal of Digestive Endoscopy. 2025;42(6):448–451. doi: 10.3760/cma.j.cn321463-20241221-00533. | Irrelevant Topic |
| 263 | Fan N, Li YX, Jiang X. Influencing factors and research progress of bowel preparation quality in children. Chinese Journal of Digestive Endoscopy. 2024;41(3):248–252. doi: 10.3760/cma.j.cn321463-20230721-00590. | Review |
| 264 | Zhao QC, Chi TY. Effect of an “Internet + medical care” platform on bowel preparation quality in outpatients undergoing colonoscopy. Chinese Journal of General Practitioners. 2022;21(3):243–249. doi: 10.3760/cma.j.cn114798-20211006-00744. | Irrelevant Topic |
| 265 | Qian HL. Relationship between withdrawal time and adenoma detection rate among endoscopists with different years of experience [dissertation]. Gannan Medical University; 2025. doi: 10.27959/d.cnki.ggnyx.2025.000258. | Irrelevant Topic |
| 266 | You HQ, Wan CG, Lei JC, et al. Observation of the effect of sequential administration of Jiechang decoction and polyethylene glycol electrolyte powder for bowel preparation before colonoscopy. Chinese Journal of Traditional Medical Science and Technology. 2018;25(5):761–762. | Irrelevant Topic |
| 267 | Zhang XQ. Correlation between personality traits, health beliefs, and bowel preparation quality in populations undergoing colonoscopy [dissertation]. Hubei University of Medicine; 2024. | Irrelevant Topic |
| 268 | Yu H. Accuracy analysis of CT colonography for detecting adenomas and cancer. Chinese Journal of Practical Medicine. 2013;40(2):68–70. doi: 10.3760/cma.j.issn.1674-4756.2013.02.031. | Irrelevant Topic |
| 269 | Chen Haisheng. Analysis of influencing factors and improvement strategies for bowel preparation quality before colonoscopy[D]. Youjiang Medical University for Nationalities,2023.doi:10.27908/d.cnki.gymzy.2023.000220. | Irrelevant Topic |
| 270 | Liu Jiana. Evaluation of colonoscopy quality control based on colorectal adenoma detection rates among physicians with different years of experience[D]. Dali University,2021.doi:10.27811/d.cnki.gdixy.2021.000289. | Irrelevant Topic |
| 271 | Zhang Cheng. Research progress on bowel preparation before colonoscopy in special patients[J]. Diet Health,2020(38):297. | Review |
| 272 | Shao Jia. Analysis of factors related to bowel preparation quality in patients with diabetes[D]. Hebei North University,2023. | Irrelevant Topic |
| 273 | Shu Xia. Pathological characteristics and detection-related factors of colorectal flat adenomas[D]. North Sichuan Medical College,2021.doi:10.27755/d.cnki.gcbyx.2021.000082. | Irrelevant Topic |
| 274 | Wang Xiaobo. Establishment and validation of a nomogram for predicting the risk of missed colorectal polyps[D]. North Sichuan Medical College,2021.doi:10.27755/d.cnki.gcbyx.2021.000032. | Irrelevant Topic |
| 275 | Jiang Shuyi. Analysis of influencing factors of colorectal polyp detection rate and missed detection rate[D]. Binzhou Medical University,2019.doi:10.27749/d.cnki.gbzyx.2019.000050. | Irrelevant Topic |
| 276 | Han Dan. Differences in intestinal microbiota between benign and malignant colorectal tumors in Tibet[D]. Tibet University,2023.doi:10.27735/d.cnki.gxzdx.2023.000355. | Irrelevant Topic |
| 277 | Peng Emin. Current status and influencing factors of bowel preparation quality before colonoscopy[D]. Central South University,2022.doi:10.27661/d.cnki.gzhnu.2022.005447. | Irrelevant Topic |
| 278 | Zhang Zhenning. Analysis of related factors influencing adenoma detection rate during colonoscopy[D]. China Medical University,2024.doi:10.27652/d.cnki.gzyku.2024.000653. | Irrelevant Topic |
| 279 | She Yumo. Diagnostic value of endoscopic ultrasonography for colorectal invasion of pelvic masses[D]. China Medical University,2024.doi:10.27652/d.cnki.gzyku.2024.000605. | Irrelevant Topic |
| 280 | Zhou Yuhang. Effects of functional oligosaccharides on Helicobacter pylori infection and regulation of gastrointestinal microecology[D]. Peking Union Medical College,2025.doi:10.27648/d.cnki.gzxhu.2025.000351. | Irrelevant Topic |
| 281 | Zhang Yue. Characteristics and related factors of pathological types of polyps detected in health examination populations undergoing colonoscopy[D]. Chinese Academy of Medical Sciences,2022. | Irrelevant Topic |
| 282 | Ma Juan. Investigation of factors influencing bowel preparation quality in patients undergoing endoscopy[D]. Zhengzhou University,2023. | Irrelevant Topic |
| 283 | Jia Lina. Analysis of optimal bowel preparation regimens and influencing factors of bowel preparation quality in patients with chronic constipation[D]. Yan’an University,2020.doi:10.27438/d.cnki.gyadu.2020.000529. | Irrelevant Topic |
| 284 | Sheng Ruli. Establishment and preliminary validation of an artificial intelligence-assisted bowel preparation application system[D]. Xinxiang Medical University,2022.doi:10.27434/d.cnki.gxxyc.2022.000201. | Irrelevant Topic |
| 285 | Zhang Yanfei, Cao Chunli, Gao Wa, et al. Current status of the safety and effectiveness of colonoscopy in elderly patients[J]. Journal of Inner Mongolia Medical University,2023,45(2):213-216,221. | Irrelevant Topic |
| 286 | Zhang Yongliang. Application effect of a multidimensional intervention model based on a WeChat public platform in colonoscopy[J]. Chinese and Foreign Medical Research,2024,3(33):57-59. | Irrelevant Topic |
| 287 | Zhu Liaoxiang. Study on the causal relationship between immune cells and colorectal cancer based on Mendelian randomization[D]. Wenzhou Medical University,2024. | Irrelevant Topic |
| 288 | Zhou Jie. Research and application of artificial intelligence in quality control and lesion recognition in digestive endoscopy[D]. Wuhan University,2021.doi:10.27379/d.cnki.gwhdu.2021.001557. | Irrelevant Topic |
| 289 | Wang Yuhong. Effect of waiting time for colonoscopy on bowel preparation quality and polyp detection rate[D]. Wannan Medical College,2022.doi:10.27374/d.cnki.gwnyy.2022.000231. | Irrelevant Topic |
| 290 | Zhang Chenxu. Analysis of influencing factors of inadequate bowel preparation before colonoscopy in patients with ulcerative colitis[D]. Tianjin Medical University,2022.doi:10.27366/d.cnki.gtyku.2022.001193. | The population does not match |
| 291 | Fu Yan. Study on whole-intestinal CT enterography based on an improved bowel preparation method[D]. Tianjin Medical University,2019.doi:10.27366/d.cnki.gtyku.2019.000241. | Irrelevant Topic |
| 292 | Huang Hongyu. Correlation between bowel preparation quality and bowel cleansing solution dose in populations with high body mass index: a multicenter, single-blind, randomized controlled study[D]. Sichuan University,2022.doi:10.27342/d.cnki.gscdu.2022.007021. | Irrelevant Topic |
| 293 | Jin Shuang. Effectiveness of linked color imaging mode in improving the detection rate of right-sided colonic polypoid lesions[D]. Shanxi Medical University,2023.doi:10.27288/d.cnki.gsxyu.2023.000684. | Irrelevant Topic |
| 294 | Zhu Hongbin, Wang Dongxu, Zhang Sujuan, et al. Application study of an intensified medication regimen for bowel preparation before same-day anesthesia colonoscopy[C]//Chinese Association of Integrative Medicine, Digestive Endoscopy Professional Committee of Chinese Association of Integrative Medicine. Abstracts of the First Fourth Academic Exchange Meeting of the Digestive Endoscopy Professional Committee of the Chinese Association of Integrative Medicine,2019:253-255.doi:10.26914/c.cnkihy.2019.001310. | Conference Abstract, etc |
| 295 | Wang Xiaoqin. Meta-analysis of risk factors for colorectal adenoma occurrence[D]. Shanxi Medical University,2022.doi:10.27288/d.cnki.gsxyu.2022.000594. | Irrelevant Topic |
| 296 | Liu Yanxiu. Effect evaluation of quantitative fecal immunochemical testing in population-based colorectal cancer screening[D]. Shandong University,2025.doi:10.27272/d.cnki.gshdu.2025.004481. | Irrelevant Topic |
| 297 | Li Shuying. Clinical characteristics of colorectal tumors initially presenting with symptoms and/or elevated tumor markers[D]. Shandong University,2022.doi:10.27272/d.cnki.gshdu.2022.004901. | Irrelevant Topic |
| 298 | Qin Siwen. Correlation analysis between bowel preparation quality before colonoscopy and clinical laboratory indicators[D]. China Three Gorges University,2024.doi:10.27270/d.cnki.gsxau.2024.000482. | Irrelevant Topic |
| 299 | Zhang Chengcheng. Clinical study of image intervention on improving intestinal cleanliness and polyp detection rate[D]. China Three Gorges University,2019.doi:10.27270/d.cnki.gsxau.2019.000302. | Irrelevant Topic |
| 300 | Zhao Yangyang, Li Ziqiong, Ma Xin, Mukeremu Yimingniyazi, Gao Feng. Analysis of factors affecting bowel preparation quality in patients with type 2 diabetes mellitus[J]. Journal of Clinical Internal Medicine,2024,41(7):480-482. | Irrelevant Topic |
| 301 | Shao Yanru. Construction and validation of a risk prediction model for hypoglycemia during the peri-colonoscopy period in elderly patients[D]. Qingdao University,2024.doi:10.27262/d.cnki.gqdau.2024.000988. | Irrelevant Topic |
| 302 | Zheng Yuting, Miao Xueqin, Kong Jiehui, et al. Optimization of nursing parameters for bowel preparation before painless electronic colonoscopy[J]. Nursing Practice and Research,2016,13(8):28-29.doi:10.3969/j.issn.1672-9676.2016.08.012. | Irrelevant Topic |
| 303 | Zhang Yunmei. Construction and application of a bowel cleansing nursing program for elderly people before colonoscopy based on the FMEA model[D]. Qingdao University,2022.doi:10.27262/d.cnki.gqdau.2022.000114. | Irrelevant Topic |
| 304 | Yang Lingjuan. Construction and application of a hypoglycemia management program for colonoscopy in elderly patients with diabetes based on intervention mapping[D]. Ningxia Medical University,2025.doi:10.27258/d.cnki.gnxyc.2025.000251. | Irrelevant Topic |
| 305 | An Jinhui. Study on factors affecting compliance with colorectal cancer screening in health examination populations[D]. Nanjing Medical University,2020.doi:10.27249/d.cnki.gnjyu.2020.000196. | Irrelevant Topic |
| 306 | Zhou Yu, Gan Tao. Analysis of senile factors in 12 cases of colonoscopy perforation[J]. Chinese Journal of Comprehensive Clinical Medicine,2004,6(8):99-100. | Irrelevant Topic |
| 307 | Chen Gong, Li Xueliang. Analysis of missed polyps during colonoscopy and related factors[C]//Proceedings of the 17th National Conference on Digestive Diseases of the Chinese Medical Association and the 2017 China Digestive Disease Conference,2017:1355-1355. | Irrelevant Topic |
| 308 | Wang Jiaqi. Effect of the Hawthorne effect on colon adenoma detection rate and multivariate analysis[D]. University of South China,2025.doi:10.27234/d.cnki.gnhuu.2025.000403. | Irrelevant Topic |
| 309 | Le Qianqian. Psychological intervention study on anxiety in patients undergoing colonoscopy[D]. University of South China,2024.doi:10.27234/d.cnki.gnhuu.2024.001300. | Irrelevant Topic |
| 310 | Tang Yinliang. Development and application of a knowledge-attitude-practice questionnaire for nurses providing bowel preparation education before colonoscopy in elderly inpatients[D]. University of South China,2024.doi:10.27234/d.cnki.gnhuu.2024.000963. | Irrelevant Topic |
| 311 | Cheng Hebo. Effects of endoscopist experience and withdrawal time on colonoscopy quality[D]. Nanchang University,2025.doi:10.27232/d.cnki.gnchu.2025.001269. | Irrelevant Topic |
| 312 | Zhang Yun. Analysis of factors influencing pain in patients undergoing colonoscopy and application of five-elements music combined with acupoint massage during examination[D]. Nanchang University,2023.doi:10.27232/d.cnki.gnchu.2023.000193. | Irrelevant Topic |
| 313 | Liu Xiangyan. Clinical application of low-radiation-dose CT virtual colonoscopy in screening colorectal tumors[D]. Inner Mongolia Medical University,2019.doi:10.27231/d.cnki.gnmyc.2019.000345. | Irrelevant Topic |
| 314 | Tan Shali. Randomized controlled study on the effect of cartoon education booklet on bowel preparation in patients[D]. Southwest Medical University,2021.doi:10.27215/d.cnki.glzyu.2021.000257. | Irrelevant Topic |
| 315 | Li Yuting. Characteristics of intestinal microbiota and metabolites in patients with metabolic associated fatty liver disease and colorectal adenomatous polyps[D]. Kunming Medical University,2022.doi:10.27202/d.cnki.gkmyc.2022.000815. | Irrelevant Topic |
| 316 | Cai Shanrong, Zhang Suzhan, Zheng Shu. Case-control study of factors influencing compliance with colorectal cancer screening among urban residents[C]//Chinese Anti-Cancer Association Cancer Epidemiology Professional Committee, Chinese Anti-Cancer Association Cancer Etiology Professional Committee, Chinese Journal of Preventive Medicine. Proceedings of the National Academic Conference on Cancer Epidemiology and Cancer Etiology. [publisher unknown],2007:113. | Conference Abstract, etc |
| 317 | Zhou Yuanyu. Clinical study on the association between plasma HDL-C, serum TBA and colorectal cancer[D]. Guangzhou Medical University,2015.doi:10.7666/d.D715034. | Irrelevant Topic |
| 318 | Cao Qiujun, Qi Birong, Sun Yi, et al. Evidence-based practice of bowel preparation before colonoscopy in hospitalized patients[J]. Chinese Journal of Nursing Education,2023,20(1):49-55.doi:10.3761/j.issn.1672-9234.2023.01.009. | Irrelevant Topic |
| 319 | Zeng Junke. Design and implementation of a virtual colonoscopy system[D]. Huazhong University of Science and Technology,2024. | Irrelevant Topic |
| 320 | Chen Chengqian. Research on bowel preparation quality assessment and colon polyp segmentation based on convolution and Transformer[D]. China University of Mining and Technology,2024. | Irrelevant Topic |
| 321 | Wang Xinyou. Multivariate analysis of bowel cleanliness and its correlation with polyp detection[D]. Kunming University of Science and Technology,2022.doi:10.27200/d.cnki.gkmlu.2022.000261. | Irrelevant Topic |
| 322 | He Bin. Study on influencing factors of bowel preparation before colonoscopy in elderly postoperative colorectal cancer patients and construction of an intervention program[D]. Jiangnan University,2025.doi:10.27169/d.cnki.gwqgu.2025.001164. | Irrelevant Topic |
| 323 | Chen Haizhen. Observation of the effect of standard-dose polyethylene glycol for bowel preparation in Qinghai region[D]. Qinghai University,2020.doi:10.27740/d.cnki.gqhdx.2020.000090. | Irrelevant Topic |
| 324 | Chen Jinmin. Diagnostic efficacy of noninvasive indicators in assessing inflammatory bowel disease activity[D]. Southern Medical University,2015. | Irrelevant Topic |
| 325 | Chen Man. Study on the effect of bowel preparation quality grading on adenoma detection rate in outpatient colonoscopy[D]. Guangdong Medical University,2021. | Irrelevant Topic |
| 326 | Chen Wanying. Optimization management study of guidance methods for bowel preparation before colonoscopy[D]. Southern Medical University,2018.doi:10.7666/d.Y3475159. | Irrelevant Topic |
| 327 | Chen Wei. Development and reliability and validity testing of a nursing assessment scale for bowel preparation in electronic colonoscopy diagnosis and treatment[D]. Wuhan Polytechnic University,2017. | Irrelevant Topic |
| 328 | Chen Ying. Clinical characteristics of Meckel’s diverticulum and diagnostic value of multislice spiral CT[D]. Soochow University,2019.doi:10.27351/d.cnki.gszhu.2019.004071. | Irrelevant Topic |
| 329 | Deng Ruichong. Efficacy of same-day split-dose compound polyethylene glycol electrolyte powder in bowel preparation[D]. Jinan University,2023.doi:10.27167/d.cnki.gjinu.2023.002813. | Irrelevant Topic |
| 330 | Feng Han. Analysis of factors influencing the defoaming effect of simethicone during colonoscopy[D]. Jilin University,2021.doi:10.27162/d.cnki.gjlin.2021.005805. | Irrelevant Topic |
| 331 | Yan Chunhao. Clinical application of cold and hot snare resection in tiny colorectal polyps[D]. Henan University,2024.doi:10.27114/d.cnki.ghnau.2024.001637. | Irrelevant Topic |
| 332 | Deng Shangxin. Individual colorectal cancer screening survey and expression study of the differentially expressed gene MMP23b in colorectal cancer[D]. Second Military Medical University,2011.doi:10.7666/d.d151369. | Irrelevant Topic |
| 333 | Ding Yangyang. Effect of using the Bristol Stool Form Scale to guide different bowel preparation regimens[D]. Anhui Medical University,2018.doi:10.7666/d.D01503481. | Irrelevant Topic |
| 334 | Dong Junqiang. Application of 64-VCT and multiple post-processing techniques in the diagnosis of colorectal cancer[D]. Zhengzhou University,2009. | Irrelevant Topic |
| 335 | Fan Xin. Application of multislice spiral CT in the diagnosis of colon tumors[D]. Dalian Medical University,2012. | Irrelevant Topic |
| 336 | Fang Jun. Analysis of influencing factors of bowel preparation quality and randomized controlled study on improving bowel preparation quality[D]. Second Military Medical University,2016. | Irrelevant Topic |
| 337 | Wang Hejie. Effect of functional gastrointestinal disorders with anxiety and depression on bowel preparation before colonoscopy[D]. Henan University,2023.doi:10.27114/d.cnki.ghnau.2023.000421. | Irrelevant Topic |
| 338 | Shen Hang. Study on individualized guidance for colonoscopy bowel preparation in different populations[D]. Hebei Medical University,2024.doi:10.27111/d.cnki.ghyku.2024.001491. | Irrelevant Topic |
| 339 | Fu Fengyihuan. The PON2-CXCL2 axis promotes the growth and invasion of colon cancer cells through the PI3K/AKT pathway[D]. Guangzhou Medical University,2020.doi:10.27043/d.cnki.ggzyc.2020.000291. | Irrelevant Topic |
| 340 | Fu Haizhen. Study on the effect of colonoscopy withdrawal time on colorectal polyp detection rate[C].Proceedings of the 2017 Academic Annual Meeting of Chinese Journal of Hospital Pharmacy,2017:376-376. | Irrelevant Topic |
| 341 | Zhu Y, Zhang DF, Wu HL, Fu PY, Feng L, Zhuang K, et al. Improving bowel preparation for colonoscopy with a smartphone application driven by artificial intelligence. NPJ Digit Med. 2023;6(1). doi: 10.1038/s41746-023-00786-y. | Irrelevant Topic |
| 342 | Gan Huoye. Effect of sedation on small bowel capsule endoscopy[D]. Southern Medical University,2016.doi:10.7666/d.Y3117003. | Irrelevant Topic |
| 343 | Gao Hui. Effects of face-to-face education and individualized regimens on bowel cleanliness in hospitalized patients[D]. Ningbo University,2023. | Irrelevant Topic |
| 344 | Gao Tao. Nursing intervention improves the success rate of bowel preparation[C].Proceedings of the National Seminar on Standardized Cleaning and Disinfection of Endoscopes, Infection Control and New Advances in Endoscopic Diagnosis and Treatment Technology,2015:138-143. | Irrelevant Topic |
| 345 | Ge Zhongzhong. Analysis of the relationship between administration time of compound polyethylene glycol electrolyte powder and bowel preparation quality before colonoscopy[D]. North China University of Science and Technology,2021. | Irrelevant Topic |
| 346 | Gong Feiyue, Qu Jiexin, Zhu Daqi, et al. Value of indigo carmine chromoendoscopy in detecting diminutive colorectal adenomas[C].Chinese Society of Digestive Endoscopy, Hong Kong Society of Digestive Endoscopy. Proceedings of the 9th International Conference on Therapeutic Endoscopy and Digestive Diseases. [publisher unknown],2008:691. | Irrelevant Topic |
| 347 | Gu Yuanting. Clinical application of a novel magnetically controlled capsule endoscopy: analysis of 500 cases from a single center[D]. Second Military Medical University,2016.doi:10.7666/d.Y3157707. | Irrelevant Topic |
| 348 | Guo Chuanguo. Segmental re-examination of the proximal colon to improve adenoma detection rate: a randomized controlled clinical trial[D]. Shandong University,2017.doi:10.7666/d.Y3242614. | Irrelevant Topic |
| 349 | Guo Jingyu. Effect of body mass index on dexmedetomidine combined with remifentanil for colonoscopy[D]. Hebei Medical University,2014.doi:10.7666/d.Y2582650. | Irrelevant Topic |
| 350 | Xie Meng. Analysis of influencing factors of colorectal polyp detection during colonoscopy[D]. Guangzhou Medical University,2023. | Irrelevant Topic |
| 351 | Guo Xiaoyang. Effects of prokinetic drugs and patient education on bowel preparation quality and preliminary study of gastric motility testing[D]. Air Force Medical University,2017. | Irrelevant Topic |
| 352 | Guo Xiaoyang, Yang Zhiping, Zhao Lina. Additional guidance can improve bowel preparation quality: a meta-analysis of randomized controlled trials[C].Proceedings of the 11th National Conference on Intestinal Diseases and 2015 Tianjin Digestive Endoscopy Annual Meeting,2016:104-104. | Irrelevant Topic |
| 353 | Liu Danlei. Comparison of the effects of normal diet and low-fiber diet before colonoscopy on bowel cleanliness[D]. Guangzhou Medical University,2020. | Irrelevant Topic |
| 354 | Han Ming. Comparative study of narrow-band imaging, chromoendoscopy and conventional endoscopy in detecting aberrant crypt foci in the distal colorectum[D]. Southern Medical University,2012.doi:10.7666/d.Y2257128. | Irrelevant Topic |
| 355 | Han Zemin. Analysis of risk factors for surgery caused by capsule endoscopy retention[D]. Southern Medical University,2018. | Irrelevant Topic |
| 356 | Sun Jianqing, Zhu Baoshan, Dai Chunhong, et al. Strategy and compliance study of colonoscopy screening for colorectal cancer[J]. Heilongjiang Science and Technology Information,2017(20):118-119.doi:10.3969/j.issn.1673-1328.2017.20.068. | Irrelevant Topic |
| 357 | Hu Jingjing. Changes in Hedgehog signaling pathway activity in rectal aberrant crypt foci and its warning effect for colorectal cancer[D]. Southern Medical University,2011.doi:10.7666/d.y1997553. | Irrelevant Topic |
| 358 | Hu Liqiong. Application value of the prokinetic drug mosapride citrate in capsule endoscopy for obscure gastrointestinal bleeding[D]. Southwest Medical University,2012.doi:10.7666/d.d226604. | Irrelevant Topic |
| 359 | Hu Yuanchao. Application of PEG-ELS combined with lactulose in bowel preparation before colonoscopy[D]. Xi’an Medical University,2019.doi:10.7666/d.D01727010. | Irrelevant Topic |
| 360 | Cai Lihua. Construction and clinical empirical study of a comprehensive nursing intervention program for bowel preparation quality in patients with diabetes[D]. Fujian Medical University,2021. | Irrelevant Topic |
| 361 | Huang Kaizhen. Analysis of missed colorectal and right-sided colonic adenomas and influencing factors during colonoscopy[D]. Guangdong Medical University,2022. | Irrelevant Topic |
| 362 | Huang Liyun. Warning effect of rectal aberrant crypt foci for colorectal cancer and advanced adenoma and analysis of related risk factors[D]. Southern Medical University,2010.doi:10.7666/d.y1770075. | Irrelevant Topic |
| 363 | Huang Shengqiu. Study on capsule endoscopy transit and image quality in the colon[D]. Southwest Medical University,2013. | Irrelevant Topic |
| 364 | Huang Wenqi. Study on changes in immunoglobulin-bound microbiota before and after fecal microbiota transplantation therapy in inflammatory bowel disease[D]. South China University of Technology,2022. | Irrelevant Topic |
| 365 | Li Wanjun. Artificial intelligence-based optimization of laxative dosage for bowel preparation: a prospective, multicenter, endoscopist-blinded, randomized controlled study[D]. Southern Medical University,2025.doi:10.27003/d.cnki.gojyu.2025.001605. | Irrelevant Topic |
| 366 | Zheng Yuying. Evaluation of the effect of linaclotide combined with compound polyethylene glycol electrolyte powder for bowel preparation before colonoscopy in patients with chronic constipation[D]. Southern Medical University,2023.doi:10.27003/d.cnki.gojyu.2023.000857. | Irrelevant Topic |
| 367 | Jian Haixu. Effectiveness of an individualized regimen based on a risk prediction model for bowel preparation failure before colonoscopy: a randomized controlled trial[D]. Shandong University,2020. | Irrelevant Topic |
| 368 | Lin Yangfan. Effects of bowel preparation and colonoscopy on intestinal microbiota and the intervention effect of Clostridium butyricum[D]. Southern Medical University,2022.doi:10.27003/d.cnki.gojyu.2022.001305. | Irrelevant Topic |
| 369 | Jin Keyin. Evaluation of the effect of combined rhubarb acupoint application on bowel preparation quality in patients with Bristol stool form types 1 and 2[D]. Wenzhou Medical University,2019. | Irrelevant Topic |
| 370 | Jin Tian. Effect of a patient follow-up management system on bowel preparation quality in patients undergoing colonoscopy[D]. Nanjing University of Chinese Medicine,2019. | Irrelevant Topic |
| 371 | Jin Yueping. Analysis of factors associated with cecal intubation rate and insertion time in unsedated colonoscopy[D]. Soochow University,2015. | Irrelevant Topic |
| 372 | Zhou Jieqiong. Influencing factors of colonoscopy quality and its application in special populations[D]. Southern Medical University,2017.doi:10.27003/d.cnki.gojyu.2017.000033. | Irrelevant Topic |
| 373 | Jing Yang. Value of 64-slice spiral CT three-dimensional imaging in preoperative evaluation of colorectal cancer[D]. Jilin University,2010. | Irrelevant Topic |
| 374 | Kang Lei, Feng Wenqing. Effect of intensified nursing intervention before colonoscopy on colonoscopy efficiency in health examination populations[C]. Shanghai Nursing Association. Proceedings of the Sixth Shanghai International Nursing Conference,2024:468-469.doi:10.26914/c.cnkihy.2024.059772. | Irrelevant Topic |
| 375 | Kang Xiaoyu. Study on patient education for bowel preparation and simplified regimens for low-risk populations[D]. Air Force Medical University,2019. | Irrelevant Topic |
| 376 | Kong Qingli. Effect of WeChat combined with Weiyou Assistant-based health education on bowel preparation in outpatients undergoing colonoscopy[D]. Shihezi University,2019. | Irrelevant Topic |
| 377 | Kou Guanjun. Study on Lactobacillus acidophilus reducing the risk of Citrobacter rodentium infection induced by polyethylene glycol bowel cleansing in mice[D]. Shandong University,2023. | Irrelevant Topic |
| 378 | Kuai Yaxian. Big data investigation of colorectal polyps and geometry-guided intelligent detection of colorectal polyps[D]. Anhui Medical University,2023. | Irrelevant Topic |
| 379 | Kuang Lei. Missed diagnosis causes of obscure gastrointestinal bleeding by capsule endoscopy and preliminary application of novel auxiliary diagnostic techniques[D]. Southern Medical University,2013.doi:10.7666/d.Y2405530. | Irrelevant Topic |
| 380 | Lan Haiyun. Study on the effect of bowel preparation quality grading on adenoma detection rate[D]. Dalian Medical University,2018. | Irrelevant Topic |
| 381 | Wang Limei. Study on high-risk factors and prediction models for several indicators of colonoscopy quality evaluation[D]. Air Force Medical University,2021.doi:10.27002/d.cnki.gsjyu.2021.000040. | Irrelevant Topic |
| 382 | Li Chuan. Clinical and experimental study on the diagnostic value of multislice spiral CT for colorectal space-occupying lesions[D]. Third Military Medical University,2006. | Irrelevant Topic |
| 383 | Li Aiyin. Preliminary study on the diagnostic efficacy of different IVIM measurement methods and parameters for preoperative staging of rectal cancer[D]. Shandong University,2017. | Irrelevant Topic |
| 384 | Li Lirong. Prospective study of different administration methods of polyethylene glycol electrolyte powder (PEG-ELS) for bowel preparation before colonoscopy in elderly patients[D]. Guilin Medical University,2022.doi:10.27806/d.cnki.gglyx.2022.000149. | Irrelevant Topic |
| 385 | Li Qian. Study on improved bowel preparation methods before colonoscopy in elderly patients[D]. Chinese PLA General Hospital,2017. | Irrelevant Topic |
| 386 | Jia Hui. Effect of water-exchange colonoscopy on adenoma detection rate and factors affecting pain in unsedated colonoscopy patients[D]. Air Force Medical University,2019. | Irrelevant Topic |
| 387 | Wei Haoqi. Effect of Bacillus subtilis dual live bacteria on intestinal microbiota and abdominal symptoms after bowel cleansing: a multicenter, randomized, double-blind, placebo-controlled clinical study[D]. Army Medical University,2023. | Irrelevant Topic |
| 388 | Fang Qiwen. Application value of narrow-band imaging in improving endoscopic diagnosis of sessile serrated lesions[D]. Naval Medical University,2024. | Irrelevant Topic |
| 389 | Liang Rong. Effect of nursing intervention based on the KTA model on bowel preparation before colonoscopy in elderly patients[D]. Shanxi University of Chinese Medicine,2019. | Irrelevant Topic |
| 390 | Liang Yao. Clinical and mechanistic study of modified Chaishao Liujun granules in the treatment of ulcerative colitis based on harmonizing the liver and spleen[D]. Guangzhou University of Chinese Medicine,2016. | Irrelevant Topic |
| 391 | Liao Jingyuan. Meta-analysis comparing water-assisted colonoscopy and traditional air-insufflation colonoscopy[D]. Sichuan Medical University,2015. | Irrelevant Topic |
| 392 | Lin Qiao, Liu Meijuan. Evidence summary for bowel preparation before colonoscopy in adults[C]. Proceedings of the China Medical Equipment Conference and 2021 Medical Equipment Exhibition,2021:156-157. | Irrelevant Topic |
| 393 | Lin Tingting. Study on influencing factors of colorectal adenoma detection rate[D]. Binzhou Medical University,2018. | Irrelevant Topic |
| 394 | Lin Wei. Analysis of redetection rate and related risk factors after endoscopic treatment of colorectal adenomatous polyps[D]. Fujian Medical University,2014. | Irrelevant Topic |
| 395 | Wang Rundong. Investigation of preliminary screening and colonoscopy application in high-risk populations for colorectal cancer[D]. Naval Medical University,2021.doi:10.26998/d.cnki.gjuyu.2021.000228. | Irrelevant Topic |
| 396 | Pan Peng. Optimization of bowel preparation for colonoscopy and establishment and preliminary validation of an artificial intelligence-assisted system for differentiating colorectal polyp properties[D]. Naval Medical University,2020.doi:10.26998/d.cnki.gjuyu.2020.000019. | Irrelevant Topic |
| 397 | Cheng Peng. Clinical study on the effects of dietary management and bowel cleansing strategies on bowel preparation quality[D]. Naval Medical University,2018.doi:10.26998/d.cnki.gjuyu.2018.000116. | Irrelevant Topic |
| 398 | Liu Jindian. Effect of bowel cleanliness self-assessment on bowel preparation and colonoscopy[D]. Qinghai University,2018. | Irrelevant Topic |
| 399 | Liu Jing. Optimization and application of an intelligent quality control system for colonoscopy[D]. Shandong University,2024.doi:10.27272/d.cnki.gshdu.2024.000098. | Irrelevant Topic |
| 400 | Liu Jun. Application of confocal laser endomicroscopy in molecular imaging and functional imaging of lower gastrointestinal lesions[D]. Shandong University,2014. | Irrelevant Topic |
| 401 | Liu Wenjun. Effects of different education methods on bowel preparation quality in colonoscopy examinees[D]. Jilin University,2017. | Irrelevant Topic |
| 402 | Liu Wenlong. Effects of different doses of simethicone emulsion on bowel defoaming and colonoscope cleaning difficulty[D]. Jilin University,2023.doi:10.27162/d.cnki.gjlin.2023.006192. | Irrelevant Topic |
| 403 | Zhang Bowei. Effects of different regimens of compound polyethylene glycol electrolyte powder on bowel cleansing efficacy and polyp detection rate[D]. Dalian Medical University,2021.doi:10.26994/d.cnki.gdlyu.2021.000145. | Irrelevant Topic |
| 404 | Liu Xiaodong. Telephone re-education can improve bowel preparation quality and polyp detection rate before colonoscopy[D]. Fourth Military Medical University,2013. | Irrelevant Topic |
| 405 | Liu Xiaoqiu. Effects of different administration methods of lactulose combined with PEG-ELS in bowel preparation[D]. North China University of Science and Technology,2022.doi:10.27108/d.cnki.ghelu.2022.000505. | Irrelevant Topic |
| 406 | Zhou Xiaobin. Effect analysis of oral polyethylene glycol electrolyte powder at different times in bowel preparation[D]. Dalian Medical University,2019.doi:10.26994/d.cnki.gdlyu.2019.000046. | Irrelevant Topic |
| 407 | Liu Zhu. Prospective study of an intensified medication regimen for bowel preparation before colonoscopy in patients after colorectal resection[D]. Shandong University,2017. | Irrelevant Topic |
| 408 | Lu Guomin. Study on endoscopic and pathological characteristics of colorectal polyps and application evaluation of narrow-band imaging[D]. Soochow University,2013. | Irrelevant Topic |
| 409 | Yu Zhijin, Luo Cheng, Xu Angao, Jiang Po. Correlation study of proximal and distal colorectal tumors[J]. Journal of Medical Research,2011,40(7):47-50. | Irrelevant Topic |
| 410 | Luo Minghan. Clinical study on the effects of different doses of simethicone on bowel preparation quality and colorectal adenoma detection rate[D]. Jinan University,2020. | Irrelevant Topic |
| 411 | Luo Wen. Study on the influence of bowel preparation quality on colonoscopy quality[D]. University of South China,2017. | Irrelevant Topic |
| 412 | Luo Xiaohui. Study on traditional Chinese medicine compounds for improving colonoscopy quality[D]. Guangzhou University of Chinese Medicine,2009. | Irrelevant Topic |
| 413 | Luo Zhuang. Expression analysis and clinical significance of COL12A1 in colon cancer[D]. Yangzhou University,2023. | Irrelevant Topic |
| 414 | Lu Tianmin. Comparison of the effects of two methods for bowel preparation before colonoscopy in female patients[D]. Huzhou University,2016. | Irrelevant Topic |
| 415 | Lu Yihao. Clinical study of Baochangqing oral liquid for bowel preparation before colonoscopy[D]. North China University of Science and Technology,2021. | Irrelevant Topic |
| 416 | Yao Dan. Current status of colonoscopy screening and service preferences among first-degree relatives of patients with colorectal cancer[D]. Chengdu University of Traditional Chinese Medicine,2024. | Irrelevant Topic |
| 417 | Ma Juan, Zeng Zhigang, Deng Weiping, et al. Effect analysis of simethicone in bowel preparation before colonoscopy in the oldest-old patients[C]. Proceedings of the 2017 Annual Meeting of the Digestive Physician Branch of the Chinese Medical Doctor Association,2017:424-424. | Conference Abstract, etc |
| 418 | Ma Zhijiao. Application value of low-dose polyethylene glycol combined with castor oil and senna in bowel cleansing[D]. Xinxiang Medical University,2016. | Irrelevant Topic |
| 419 | Mi Yu. Study on the preparation process and quality standards of Qingchang Xiaopao emulsion[D]. Jiangsu Ocean University,2022. | Irrelevant Topic |
| 420 | Huang Yongquan. Analysis of high-risk factors for colorectal adenomatous polyps and construction of a prediction model[D]. Yangtze University,2024. | Irrelevant Topic |
| 421 | Pan Weidong. Comparative study of CT colonography and colonoscopy in the diagnosis of polyps[D]. Peking Union Medical College,2005. | Irrelevant Topic |
| 422 | Pang Qiuping. Effect observation of different administration methods of polyethylene glycol electrolyte powder for bowel preparation before colonoscopy[D]. Shandong University,2017.doi:10.7666/d.Y3240735. | Irrelevant Topic |
| 423 | Cai Yi. Role and mechanism of methylation- and Micro-RNA-related genes in colorectal cancer screening and diagnosis[D]. Anhui Medical University,2021. | Irrelevant Topic |
| 424 | Peng Xinxuan. Analysis of factors associated with survival after pulmonary metastasectomy in patients with colorectal cancer[D]. Guangzhou Medical University,2021. | Irrelevant Topic |
| 425 | Feng Lei. Investigation of compliance with colonoscopy screening and influencing factors in high-risk populations for colorectal cancer[J]. Famous Doctor,2024(18):30-32.doi:10.20255/j.cnki.issn1674-9561.2024.18.004. | Irrelevant Topic |
| 426 | Bian Xuqiang, Zhang Yangyang, Geng Xianhui, et al. Effect of bowel preparation in low-risk populations guided by a WeChat mini program[J]. China Medicine and Pharmacy,2024,14(13):156-159,172.doi:10.20116/j.issn2095-0616.2024.13.37. | Irrelevant Topic |
| 427 | Cheng Rong, Wu Cheng. Analysis of influencing factors of poor bowel preparation quality before colonoscopy in children[J]. China Medical Herald,2024,21(2):104-107,123.doi:10.20047/j.issn1673-7210.2024.02.21. | The population does not match |
| 428 | Sha Jie. Diagnostic value of acetic acid combined with narrow-band imaging magnifying endoscopy in gastrointestinal mucosal lesions[D]. Nanjing Medical University,2017. | Irrelevant Topic |
| 429 | Hua Yi, Jiang Hua, Xu Yanling, et al. Evidence-based construction of a sensitive indicator system for nursing quality of bowel preparation in hospitalized patients[J]. Today Nurse,2025,32(29):146-151.doi:10.19792/j.cnki.1006-6411.2025.29.030. | Irrelevant Topic |
| 430 | Dong Zhenyin, Yue Shixia, Wang Meigui, et al. Influencing factors and nursing intervention strategies for bowel preparation quality before colonoscopy in preschool children[J]. Today Nurse,2023,30(31):94-98.doi:10.19791/j.cnki.1006-6411.2023.31.024. | The population does not match |
| 431 | Han Dan, Ciren Yangjin, Li Qiuhong, et al. Differences in intestinal microbiota between patients with benign and malignant colorectal tumors in high-altitude areas and normal populations in low-altitude areas[J]. Journal of Peking University (Health Sciences),2025,57(3):578-583.doi:10.19723/j.issn.1671-167X.2025.03.023. | Irrelevant Topic |
| 432 | Luo Xueting. Comparative clinical study of face-to-face re-education and telephone re-education on bowel preparation quality before colonoscopy[D]. Shandong University,2017. | Irrelevant Topic |
| 433 | Shen Dawei. Application study of deep learning-assisted quality control for magnetically controlled capsule gastroscopy[D]. Shandong University,2022.doi:10.27272/d.cnki.gshdu.2022.001153. | Irrelevant Topic |
| 434 | Shen Haiyan. Evaluation of intestinal mucosal functional status in patients with ulcerative colitis using confocal laser endomicroscopy[D]. Zhejiang University,2017. | Irrelevant Topic |
| 435 | Ayimukedisi Yalikong, Zhuang Huijun, Cai Shilun, et al. Application of deep learning-based artificial intelligence in colonoscopy[J]. Chinese Journal of Practical Surgery,2020,40(3):353-357.doi:10.19538/j.cjps.issn1005-2208.2020.03.28. | Irrelevant Topic |
| 436 | Shi Yongsheng. Clinical endoscopic characteristics and diagnosis and treatment strategies of Mallory-Weiss syndrome[D]. Shanxi Medical University,2023.doi:10.27288/d.cnki.gsxyu.2023.001650. | Irrelevant Topic |
| 437 | Shi Liwei, Liang Yan. Research progress on influencing factors and intervention measures for bowel preparation quality before colonoscopy[C]. Proceedings of the First Training Course on Clinical Medical Research Topic Selection, Research Design Statistics and Reporting Standards of Progress in Anatomical Sciences,2024:1-3. | Review |
| 438 | Cui Nailing, Li Wenwen, Zhang Mohan, et al. Study on bowel preparation quality based on a bowel preparation failure prediction model[J]. Journal of Weifang Medical University,2020,42(4):297-299.doi:10.16846/j.issn.1004-3101.2020.04.019. | Irrelevant Topic |
| 439 | Song Jiefei. Analysis of risk factors associated with missed colorectal adenomas during colonoscopy[D]. Dalian Medical University,2014. | Irrelevant Topic |
| 440 | Su Jingran. Development and application of an artificial intelligence-based colonoscopy quality control model[D]. Shandong University,2020. | Irrelevant Topic |
| 441 | Sun Chang. Effects of different intervention methods on bowel preparation quality in hospitalized patients undergoing colonoscopy diagnosis and treatment[D]. Harbin Institute of Technology,2019. | Irrelevant Topic |
| 442 | Sun Wenrong. Study on hydrogen breath test for predicting bowel preparation quality before colonoscopy[D]. Naval Medical University,2018. | Irrelevant Topic |
| 443 | Sun Yining. Individualized application of oral contrast agent dosage for small bowel CT enterography based on BMI[D]. Shandong University,2020. | Irrelevant Topic |
| 444 | Han Xuli, Hu Yuanchao, Guo Wei, et al. Application of lactulose in colonoscopy in elderly patients with constipation and analysis of related influencing factors[J]. Journal of Shenyang Medical College,2020,22(3):243-246.doi:10.16753/j.cnki.1008-2344.2020.03.013. | Irrelevant Topic |
| 445 | Tang Wanlin. Analysis of related factors for missed colorectal polyps[D]. Wannan Medical College,2018. | Irrelevant Topic |
| 446 | Han Feng, Xue Deshun, Li Huajin. Main influencing factors of adenoma detection rate in asymptomatic colonoscopy screening populations[J]. China Foreign Medical Treatment,2025,44(9):42-45.doi:10.16662/j.cnki.1674-0742.2025.09.042. | Irrelevant Topic |
| 447 | Tong Yao. Clinical observation of linaclotide combined with compound polyethylene glycol electrolyte powder for bowel preparation in elderly patients[D]. Chongqing Medical University,2023. | Irrelevant Topic |
| 448 | Tong Yao. Clinical observation of linaclotide combined with compound polyethylene glycol electrolyte powder for bowel preparation in elderly patients[D]. Chongqing Medical University,2023. | Irrelevant Topic |
| 449 | Wang Chenglong. Application value of lesion detection rate and missed detection rate in colonoscopy quality control[D]. Naval Medical University,2019. | Irrelevant Topic |
| 450 | Wang Chunhua. Study on methods for improving lesion detection rate under digestive endoscopy[D]. Third Military Medical University,2015. | Irrelevant Topic |
| 451 | Fu Qiying, Fang Yingying, Wang Fang, et al. Effect of online animated educational videos on bowel preparation before colonoscopy[J]. Chinese Preventive Medicine,2018,19(8):633-636.doi:10.16506/j.1009-6639.2018.08.018. | Irrelevant Topic |
| 452 | Wang Houli. Comparative study of the effects of video education and conventional education on bowel preparation quality in patients undergoing colonoscopy[D]. Inner Mongolia Medical University,2018. | Irrelevant Topic |
| 453 | Guo Shengli, Yuan Wei, Zhu Ting, et al. Systematic review of risk prediction models for bowel preparation failure before colonoscopy[J]. Journal of Nursing,2022,29(1):35-40.doi:10.16460/j.issn1008-9969.2022.01.035. | Review |
| 454 | Wang Jingjing. Research progress on nursing intervention in bowel preparation with oral compound polyethylene glycol electrolyte powder[C]. Proceedings of the Fifth Shanghai International Nursing Conference,2022:6-7. | Review |
| 455 | Chen Ruiyan, Chen Xiaodong, Wu Jiachuan, et al. Comparison of bowel preparation efficacy between single-dose and split-dose oral polyethylene glycol electrolyte solution[J]. Journal of Clinical Medical Literature,2019,6(50):15-16.doi:10.16281/j.cnki.jocml.2019.50.009. | Irrelevant Topic |
| 456 | Wang Meiling. Construction and application of an education program for outpatients with diabetes undergoing painless gastrointestinal endoscopy[D]. Qingdao University,2023.doi:10.27262/d.cnki.gqdau.2023.002364. | Irrelevant Topic |
| 457 | Wang Qingjiao. Clinical application of virtual colonoscopy in the diagnosis of colorectal masses[D]. Southern Medical University,2013. | Irrelevant Topic |
| 458 | Chen Puan. Analysis of risk factors and corresponding preventive measures for intestinal perforation caused by painless fiberoptic colonoscopy[J]. Journal of Clinical Medical Literature,2015,2(1):3-4.doi:10.16281/j.cnki.jocml.2015.01.090. | Irrelevant Topic |
| 459 | Wang Shuai. Diagnostic value of magnetically controlled capsule endoscopy in gastric and small intestinal diseases[D]. Hebei Medical University,2018. | Irrelevant Topic |
| 460 | Wang Ting. Effect of polycarbophil calcium tablets combined with compound electrolyte powder on bowel preparation efficacy[D]. Jiangsu University,2023.doi:10.27170/d.cnki.gjsuu.2023.001521. | Irrelevant Topic |
| 461 | Wang Xianbin. Study on 64-slice spiral CT three-dimensional reconstruction for preoperative staging prediction and surgical approach evaluation of colorectal cancer[D]. Hebei United University,2011. | Irrelevant Topic |
| 462 | Wu Meng, Chen Jing, Gao Zhi, et al. Evaluation of colonoscopy polyp miss rate and its influencing factors using repeat examination in day treatment[J]. Journal of Army Medical University,2025,47(23):2943-2952.doi:10.16016/j.2097-0927.202509098. | Irrelevant Topic |
| 463 | Zhu Lili, Yang Hong, Yang Aiming. Research progress on the application of compound polyethylene glycol electrolyte powder for bowel preparation before colonoscopy[J]. Beijing Medical Journal,2021,43(11):1117-1119.doi:10.15932/j.0253-9713.2021.11.018. | Review |
| 464 | Feng Li, Song Yanming, Zhang Li, et al. Analysis of bowel preparation quality and influencing factors before colonoscopy in patients with liver cirrhosis[J]. Beijing Medical Journal,2020,42(10):1032-1035.doi:10.15932/j.0253-9713.2020.10.029. | The population does not match |
| 465 | Wang Yaxin. Analysis of influencing factors of adenoma detection rate during colonoscopy and clinicopathological characteristics of colorectal polyps[D]. Jilin University,2015. | Irrelevant Topic |
| 466 | Zhao Yanzhen, Wu Mingjie, Wang Aixiang, et al. Influencing factors and nursing care for bowel cleansing efficacy before colonoscopy[J]. Hebei Medical Journal,2012,34(23):3667-3668.doi:10.3969/j.issn.1002-7386.2012.23.085. | Irrelevant Topic |
| 467 | Wang Zhenyu. Diagnostic value and influencing factors of capsule endoscopy in overt obscure gastrointestinal bleeding[D]. Shantou University,2018. | Irrelevant Topic |
| 468 | Zhou Tao, Zhang Menghui, Xie Rui, et al. Comparison of intestinal polyp detection rates between urban and rural populations: a propensity score matching-based study[J]. Huaihai Medicine,2023,41(5):490-494.doi:10.14126/j.cnki.1008-7044.2023.05.013. | Irrelevant Topic |
| 469 | Wu Dan. Study on predictors of inadequate bowel preparation in elderly people[D]. Qingdao University,2018. | Irrelevant Topic |
| 470 | Wu Yali. Research progress on bowel preparation regimens for colorectal endoscopy[C]. Nanjing Rehabilitation Medical Association. Proceedings of the Seventh National Academic Exchange Conference on Rehabilitation and Clinical Pharmacy,2024:567-571.doi:10.26914/c.cnkihy.2024.004116. | Review |
| 471 | Xiang Li. Study on missed detection and detection of flat adenomas during colonoscopy and related influencing factors[D]. Southern Medical University,2014. | Irrelevant Topic |
| 472 | Xie Li. Performance validation of human septin9 gene methylated DNA detection based on bisulfite modification combined with TaqMan probe technology and its application value in colorectal cancer diagnosis[D]. Tongji University,2017. | Irrelevant Topic |
| 473 | Zhang Meidan, Ding Aiqin, Wu Yixue. Effect of pathway nursing based on analysis of bowel preparation interference factors on bowel preparation satisfaction in patients with chronic constipation undergoing colonoscopy[J]. Chinese and Foreign Medical Research,2025,23(23):157-160.doi:10.14033/j.cnki.cfmr.2025.23.042. | Irrelevant Topic |
| 474 | Xie Xuanyu. Clinical, pathological, and risk factor characteristics of colorectal serrated lesions[D]. Guangdong Medical University,2024. | Irrelevant Topic |
| 475 | Xie Yan. Application of multiplex DNA methylation detection based on peripheral blood mononuclear cells in early diagnosis of colorectal cancer[D]. Shandong University,2024. | Irrelevant Topic |
| 476 | Guo Jingyu. Effect of body mass index on dexmedetomidine combined with remifentanil for colonoscopy[D]. Hebei Medical University,2014.doi:10.7666/d.Y2582650. | Irrelevant Topic |
| 477 | Xu Dong. Comparative study of endoluminal ultrasound and spiral CT for preoperative staging of rectal cancer with postoperative pathology[D]. Zhejiang University,2007. | Irrelevant Topic |
| 478 | Xie Yuxian. Investigation of influencing factors of adenoma detection rate during colonoscopy and pathological characteristics of colorectal polyps[J]. Chinese and Foreign Medical Research,2019,17(11):55-56.doi:10.14033/j.cnki.cfmr.2019.11.023. | Irrelevant Topic |
| 479 | Chen Qingqing, Wang Xinyan, Fan Huizhen, et al. Analysis of bowel preparation effects and influencing factors of two bowel cleansing agents in patients with constipation[J]. Practical Clinical Journal of Integrated Traditional Chinese and Western Medicine,2017,17(8):1-2,54.doi:10.13638/j.issn.1671-4040.2017.08.001. | Irrelevant Topic |
| 480 | Xu Wenmin. Evaluation of colorectal cancer screening strategies and analysis of colonoscopy results in Guangzhou[D]. Guangzhou Medical University,2020. | Irrelevant Topic |
| 481 | Yan Fangbo, Chao Shuaiheng, Li Yan, et al. Value and influencing factors of artificial intelligence-assisted colonoscopy diagnostic technology in diagnosing intestinal adenomas[J]. Journal of Laparoscopic Surgery,2024,29(9):678-682.doi:10.13499/j.cnki.fqjwkzz.2024.09.678. | Irrelevant Topic |
| 482 | Yan Feihu. Prospective randomized controlled study on the correlation between abdominal compression anti-looping technique and colonoscope insertion process and diagnostic and therapeutic quality[D]. Naval Medical University,2017.doi:10.7666/d.Y3247238. | Irrelevant Topic |
| 483 | Yang Lei. Clinical application value of abdominal CTA, CTC and fusion images in colon and rectal tumors[D]. Capital Medical University,2008. | Irrelevant Topic |
| 484 | Zhang Haiyan, Tan Jingyu, Liang Ling, et al. Compliance and influencing factors of free colorectal endoscopy screening among high-risk populations for colorectal cancer in Chongqing[J]. Journal of Chongqing Medical University,2016,41(12):1263-1267.doi:10.13406/j.cnki.cyxb.001124. | Irrelevant Topic |
| 485 | Yang Wenbo. Multicenter observational study on the effect of waiting time after completion of bowel preparation on bowel preparation quality[D]. Fujian Medical University,2019. | Irrelevant Topic |
| 486 | Yang Ya. Clinical observation and analysis of the effects of different PEG administration methods on bowel preparation in patients undergoing morning colonoscopy[D]. Kunming Medical University,2018. | Irrelevant Topic |
| 487 | Yang Xiaoming, Jia Xiaodong, Shen Bing, et al. Influencing factors of compliance with colonoscopy screening among high-risk populations for colorectal cancer in Jing’an District, Shanghai[J]. Journal of Environmental and Occupational Medicine,2016,33(5):421-426.doi:10.13213/j.cnki.jeom.2016.15440. | Irrelevant Topic |
| 488 | Yao Hui. Therapeutic effect and related mechanisms of autologous bone marrow mesenchymal stem cell transplantation in canine acute radiation enteropathy[D]. Fourth Military Medical University,2017. | Irrelevant Topic |
| 489 | Ye Leping. Series study on bowel preparation regimens for intestinal endoscopy[D]. Anhui Medical University,2015. | Irrelevant Topic |
| 490 | Ye Qianyun. Analysis of related factors of colorectal polyps and study on syndrome type distribution[D]. Guangzhou University of Chinese Medicine,2018. | Irrelevant Topic |
| 491 | Ye Yuanning. Diagnostic value of fecal neopterin and calprotectin in assessing inflammatory bowel disease activity[D]. Southern Medical University,2016. | Irrelevant Topic |
| 492 | Yu Shuxia. Expression of HIF-1α in ulcerative colitis and molecular mechanism of the lncRNA HNF4A-AS1/miF 671-5p/HNF4A axis in improving inflammatory injury[D]. Shandong University,2023.doi:10.27272/d.cnki.gshdu.2023.007960. | Irrelevant Topic |
| 493 | Yu Jinlong. Construction and visualization of a three-dimensional digital anatomical model of the colorectum based on CTA images and its basic and preliminary clinical application[D]. Southern Medical University,2009. | Irrelevant Topic |
| 494 | Yuan Jiawei. Diagnostic efficacy of serological indicators in assessing mucosal healing and predicting recurrence in Crohn’s disease[D]. Southern Medical University,2018. | Irrelevant Topic |
| 495 | Yuan Youhong. Effects of ulinastatin on cellular immunity and liver and kidney function in patients undergoing laparoscopic colorectal cancer surgery[D]. Southern Medical University,2013. | Irrelevant Topic |
| 496 | Xiong Yuanqin, Ai Yaowei, Li Haiyang. Research progress on bowel preparation in populations with some high-risk factors[J]. Advances in Clinical Medicine,2024,14(2):2641-2648.doi:10.12677/ACM.2024.142371. | Review |
| 497 | Yao Jiaqi, Yang Cheng, Zhang Leyao. Effect of artificial intelligence technology on polyp detection rate among high-risk populations for colorectal cancer screening in communities[J]. China Journal of Endoscopy,2023,29(9):63-69.doi:10.12235/E20220393. | Irrelevant Topic |
| 498 | Chen Haisheng, Tao Yunxiu, Cen Chao. Research progress on bowel preparation regimens before colorectal endoscopy[J]. Medical Innovation of China,2023,20(3):152-156.doi:10.3969/j.issn.1674-4985.2023.03.037. | Review |
| 499 | Zhang Jie. Diagnostic value of air-based small bowel CT virtual endoscopy for small bowel lesions[D]. Capital Medical University,2010. | Irrelevant Topic |
| 500 | Zhang Ke. Analysis of related influencing factors of colorectal adenoma miss rate based on repeated colonoscopy within a short period[D]. Dalian Medical University,2023. | Irrelevant Topic |
| 501 | Cai Ye, Duan Nimei, Zhang Jing, et al. Analysis of influencing factors of qualified bowel preparation rate in patients undergoing colonoscopy[J]. International Nursing Research,2025,7(3).doi:10.12208/j.ijnr.20250163. | Irrelevant Topic |
| 502 | Zhang Shuai. Application of new CT and MR techniques in colorectal lesions[D]. Fudan University,2007.doi:10.7666/d.y1273701. | Irrelevant Topic |
| 503 | Zhang Xiaodan. Diagnostic value of MRC combined with serum and immunohistochemical indicators for infiltrative colon cancer[D]. Tianjin Medical University,2015.doi:10.7666/d.D798204. | Irrelevant Topic |
| 504 | Huang Shuhan, Zhong Kebo, Gao Peichun, et al. Effect evaluation of an improved administration method of laxatives for bowel preparation before colonoscopy[J]. International Nursing Research,2025,7(3).doi:10.12208/j.ijnr.20250119. | Irrelevant Topic |
| 505 | Zhang Yan. Value of optical enhancement endoscopy in the optical diagnosis of colorectal polyps[D]. Shandong University,2017.doi:10.7666/d.Y3241226. | Irrelevant Topic |
| 506 | Yin Jie, Yang Hua, Teng Yufang, et al. Compliance with colonoscopy screening and influencing factors among high-risk populations for colorectal cancer in Nanjing[J]. South China Journal of Preventive Medicine,2025,51(1):25-29.doi:10.12183/j.scjpm.2025.0025. | Irrelevant Topic |
| 507 | Zhu Lili, Shi Change, Zhan Jun, et al. Clinical application and influencing factors analysis of an artificial intelligence digestive endoscopy system-assisted diagnosis of colon polyps[J]. Chinese Journal of Robotic Surgery,2024,5(3):478-483.doi:10.12180/j.issn.2096-7721.2024.03.027. | Irrelevant Topic |
| 508 | Yang Qianqian, Yuan Wei, Zhang Xuemin, et al. Research progress on the risk of hypoglycemia during colonoscopy in patients with diabetes[J]. Chinese General Practice Nursing,2025,23(16):3104-3108.doi:10.12104/j.issn.1674-4748.2025.16.023. | Review |
| 509 | Guo Fan, Lu Lingling, Pi Junfang, et al. Best evidence summary for improving bowel preparation quality and patient tolerance before colonoscopy[J]. Chinese General Practice Nursing,2023,21(10):1306-1310.doi:10.12104/j.issn.1674-4748.2023.10.003. | Irrelevant Topic |
| 510 | Zhao Caihan. Analysis of influencing factors of recurrence after endoscopic resection of colorectal adenomatous polyps[D]. Jilin University,2017. | Irrelevant Topic |
| 511 | Zhao Shengbing. Series study on optimizing colonoscopy quality and validating the diagnostic efficacy of an artificial intelligence-assisted colon polyp detection system[D]. Naval Medical University,2019. | Irrelevant Topic |
| 512 | Zheng Jianshan, Wu Guanhong, Zhang Liyan, et al. Analysis of influencing factors and model construction for unqualified bowel preparation before colonoscopy in children[J]. Chinese General Practice Nursing,2023,21(1):99-103.doi:10.12104/j.issn.1674-4748.2023.01.023. | The population does not match |
| 513 | Zhao Juan. Analysis of related factors affecting bowel cleansing efficacy in hospitalized patients taking oral compound polyethylene glycol electrolyte powder[J]. Chinese General Practice Nursing,2020,18(22):2873-2876.doi:10.12104/j.issn.1674-4748.2020.22.029. | Irrelevant Topic |
| 514 | Zhang Na, Xu Miaomiao, Zhang Yafeng, et al. Meta-analysis of subject-related influencing factors of bowel preparation quality before colonoscopy[J]. Chinese Nursing Research,2023,37(6):963-969.doi:10.12102/j.issn.1009-6493.2023.06.005. | Irrelevant Topic |
| 515 | Hu Lu, He Rongpan, Zhou Mengjiao, et al. Research progress on bowel preparation regimens before colonoscopy in special populations[J]. Chinese Nursing Research,2020,34(5):853-857.doi:10.12102/j.issn.1009-6493.2020.05.025. | Review |
| 516 | Chen Weidong, Liu Lewei, Wu Suna, et al. Value of intestinal microbiota testing in the diagnosis of colorectal adenoma and colorectal cancer[J]. Zhejiang Medical Journal,2024,46(15):1618-1624,1631,insert 3-insert 4.doi:10.12056/j.issn.1006-2785.2024.46.15.2024-683. | Irrelevant Topic |
| 517 | Zhou Lei, Zhang Heng, Sun Shengbin, et al. Clinical, endoscopic and pathological characteristics of colorectal polyps in elderly people[C]. Proceedings of the 2018 China Digestive Physician Annual Meeting,2018:189-189. | Conference Abstract, etc |
| 518 | Yang Jin, Wang Wei, Zhang Xiaoyan, et al. Influencing factors and prediction model construction for bowel preparation failure in elderly patients undergoing painless gastrointestinal endoscopy[J]. Chinese Journal of Multiple Organ Diseases in the Elderly,2025,24(5):341-345.doi:10.11915/j.issn.1671-5403.2025.05.071. | The population does not match |
| 519 | Yang Fan, Xu Jizong, Zhang Xian. Comparative analysis of high-risk factors and treatment regimens for intestinal perforation after colonoscopy treatment[J]. World Chinese Journal of Digestology,2021,29(13):715-719.doi:10.11569/wcjd.v29.i13.715. | Irrelevant Topic |
| 520 | Guo XY, Shi X, Kang XY, Luo H, Wang XP, Jia H, et al. Risk factors associated with inadequate bowel preparation in patients with functional constipation. Dig Dis Sci. 2020;65(4):1082-1091. doi: 10.1007/s10620-019-05847-5. | The population does not match |
| 521 | Adris N, Ayonrinde O. Prevalence and associations of sessile serrated polyps in a general colonoscopy population. J Gastroenterol Hepatol. 2019;34:233. doi: 10.1111/jgh.14806. | Conference Abstract, etc |
| 522 | Al Dulaijan B, Shah R, Davitkov P, Falck-Ytter YT, Macaron C. One week polyethylene glycol (PEG) 3350 followed by split dose PEG electrolyte solution with or without ascorbic acid improved colonoscopy bowel preparation in patients with constipation: a quality improvement project at a large veterans affairs hospital. Gastrointest Endosc. 2020;91(6):AB480. doi: 10.1016/j.gie.2020.03.2941. | Conference Abstract, etc |
| 523 | Alasfarq F, Almuhanna Y, Alotaibi F. Factors affecting colonoscopy completion. Surgical Endoscopy and Other Interventional Techniques. 2015;29:S447. doi: 10.1007/s00464-015-4130-0. | Conference Abstract, etc |
| 524 | Allen A, Ektefaie Y, Garikipati A, Lam C, Green-Saxena A, Siefkas A, et al. A machine learning algorithm to predict gastrointestinal bleeding requiring intervention. Gastroenterology. 2021;160(6):S-422. doi: 10.1016/S0016-5085(21)01715-7. | Conference Abstract, etc |
| 525 | Alvarez Gonzalez MA, Pantaleon MA, Bernard Cabredo B, Garcia-Rodriguez A, Frago Larramona S, Nogales Rincon O, et al. Nurse-led educational telephone intervention for bowel preparation for repeat colonoscopy after bowel preparation failure. United European Gastroenterology Journal. 2019;7(8):829. doi: 10.1177/205064061985467. | Conference Abstract, etc |
| 526 | Alvarez-Gonzalez MA, Elosua A, Diez-Redondo P, Nogales O, Busto V, Pantaleon Sanchez MA, et al. High quality bowel cleansing with ultralow volume laxatives: a multicenter noninferiority randomized clinical trial. Gastrointest Endosc. 2025;101(5):S285. doi: 10.1016/j.gie.2025.03.502. | Conference Abstract, etc |
| 527 | Alvarez-Gonzalez MA, Fernandez-Clotet A, Casals G, Pantaleon MA, Amorós J, Ibañez I, et al. Improvement of tolerability of bowel preparation for colonoscopy in diabetic patients (study idimeprep). A randomised controlled trial. Gastrointest Endosc. 2016;83(5):AB222. | Conference Abstract, etc |
| 528 | Amano T, Nishida T, Shimakoshi H, Shimoda A, Sugimoto A, Takahashi K, et al. The number of endoscopically detected polyps during baseline colonoscopy can predict the risk of having advanced colorectal neoplasm. Gastrointest Endosc. 2017;85(5):AB395. | Conference Abstract, etc |
| 529 | Amer L, Kaouk N, Yaacoub T, Slim R, Amara J, Honein K, et al. Unveiling colon cleansing quality: a face-off between artificial intelligence and conventional methods using bowel image features. United European Gastroenterology Journal. 2024;12:1329-1330. doi: 10.1002/ueg2.12615. | Conference Abstract, etc |
| 530 | Amornsawadwattana S, Anthony H, Patel RS, Sloan I, Hollander T, Chen CH. Patients’ self-identified stool color is comparable to patients’ description of rectal effluent for assessing quality of bowel preparation at colonoscopy (poster presentation). Gastroenterology. 2019;156(6):S-146. doi: 10.1016/S0016-5085(19)37154-9. | Conference Abstract, etc |
| 531 | Anderson JC, Mott LA, Cole B, Baron JA, Robertson DJ. Surveillance of low risk adenomas at 3 versus 5 year intervals: data to support current surveillance guidelines. Gastroenterology. 2015;148(4):S153‐S154. | Conference Abstract, etc |
| 532 | Antonelli G, Desideri F, Scarozza P, Andrisani G, Zerboni G, Furnari M, et al. Artificial intelligence for leaving in situ colorectal polyps: results from a randomised trial. Gastrointest Endosc. 2025;101(5):S23‐S24. doi: 10.1016/j.gie.2025.03.046. | Conference Abstract, etc |
| 533 | Archer T, Baig F, Baxter A, Franklin J, Amin M, Parra-Blanco A, et al. A comprehensive validation of the accuracy of two predictive models for inadequate bowel preparation. Gut. 2023;72:A150. doi: 10.1136/gutjnl-2023-BSG.255. | Conference Abstract, etc |
| 534 | Archer T, Dear K, Foley S, Cole A, Andreyev J, Fateen W, et al. Does an educational video improve bowel preparation in patients first colonoscopy? A UK multi-centre RCT. Gut. 2021;70(SUPPL 1):A45. doi: 10.1136/gutjnl-2020-bsgcampus.83. | Conference Abstract, etc |
| 535 | Archer T, Dear K, Thoufeeq M, Foley S, Cole A, Andreyev J, et al. Can an educational video improve the adequacy of bowel preparation for patients undergoing their first colonoscopy the ebops study. United European Gastroenterology Journal. 2021;9(SUPPL 8):831‐832. doi: 10.1002/ueg2.12144. | Conference Abstract, etc |
| 536 | Aryan M, Ramapathy CK, Cooper JA, Norwood DA, Culp P, Holyfield A, et al. Patient perception of stool color predicts bowel preparation quality prior to outpatient colonoscopy at a tertiary care center. 2025. p. S172. | Conference Abstract, etc |
| 537 | Balch J, Rashid A, Mazirka P, Ehresmann K, Goldstein L, Terracina K, et al. Can machine learning models predict inadequate bowel preparation at colonoscopy? Diseases of the colon and rectum. 2024;67(6):e991. | Conference Abstract, etc |
| 538 | Baraty B, Yau Y, Leong R. Colonoscopy bowel preparation assessment using transabdominal ultrasound examination. J Gastroenterol Hepatol. 2018;33:153‐154. doi: 10.1111/jgh.14399. | Conference Abstract, etc |
| 539 | Barkun AN, Martel M, Epstein IL, Halle P, Hilsden RJ, James P, et al. The bowel cleansing national initiative (BCLEAN): predictors of inadequate bowel preparation. J Can Assoc Gastroenterol. 2020;3. doi: 10.1093/jcag/gwz047.001. | Conference Abstract, etc |
| 540 | Beran A, Aboursheid T, Ali AH, Albunni H, Mohamed MF, Vargas A, et al. Risk factors for inadequate bowel preparation in colonoscopy: a comprehensive systematic review and meta-analysis. Am J Gastroenterol. 2024;119(12):2389-2397. doi: 10.14309/ajg.0000000000003073. | Conference Abstract, etc |
| 541 | Berger A, Cesbron Metivier E, Bertrais S, Dib N, Luet D, Olivier A, et al. A prediction score to identify patients at risk for inadequate bowel preparation evaluated by a selfadministered questionnaire. United European Gastroenterology Journal. 2016;4(5):A210. doi: 10.1177/2050640616663689. | Conference Abstract, etc |
| 542 | Berger A, Cesbron-Metivier E, Bertrais S, Olivier A, Becq A, Boursier J, et al. A predictive score of inadequate bowel preparation based on a selfadministred questionnaire. Endoscopy. 2018;50(4):S64. doi: 10.1055/s-0038-1637216. | Conference Abstract, etc |
| 543 | Bertiger G, Hookey L, Johnson KL, Ando M, Stong L, Khan N, et al. Age-dependent tolerability and adenoma detection with a ready-to-drink low-volume bowel preparation: subgroup analysis from a randomized, assessor-blinded, multicenter study. American Journal of Gastroenterology. 2019;114:S190. doi: 10.14309/01.ajg.0000590824.38344.9d. | Conference Abstract, etc |
| 544 | Borg B, Gupta N, Zuckerman G, Banerjee B. Predictors of poor bowel preparation in colonoscopy. American Journal of Gastroenterology. 2008;103:S512-S513. doi: 10.14309/00000434-200809001-01310. | Conference Abstract, etc |
| 545 | Brand EC, Crook JE, Thomas CS, Siersema PD, Rex DK, Wallace MB. Predicted adenoma detection rate (ADR) and comparison to actual physicians' ADR. United European Gastroenterology Journal. 2016;4(5):A586-A587. doi: 10.1177/2050640616663689. | Conference Abstract, etc |
| 546 | Buisson A, Hordonneau C, Goutorbe F, Allimant C, Goutte M, Pereira B, et al. MRI remission after therapeutic intervention is associated with more time spent in clinical corticosteroids-free remission and decreased risk of surgery in Crohn's disease. Gastroenterology. 2017;152(5):S39-S40. | Conference Abstract, etc |
| 547 | Buisson A, Hordonneau C, Goutorbe F, Allimant C, Goutte M, Petitcolin V, et al. MRI remission after therapeutic intervention is associated with more time spent in clinical corticosteroids-free remission and decreased risk of surgery in Crohn's disease. United European Gastroenterology Journal. 2016;4(5):A250. doi: 10.1177/2050640616663689. | Conference Abstract, etc |
| 548 | Buisson A, Hordonneau C, Goutorbe F, Allimant C, Goutte M, Reymond M, et al. MRI remission after therapeutic intervention is associated with more time spent in clinical corticosteroids-free remission and decreased risk of surgery in Crohn's disease. Journal of Crohn's and Colitis. 2017;11:S138-S139. doi: 10.1093/ecco-jcc/jjx002.249. | Conference Abstract, etc |
| 549 | Burke CA, Mankaney GN, Scott TA, Ando M, Stong L, Boules M. Polyp detection and colon cleansing efficacy in younger adults (<50 yr) with a sodium picosulfate, magnesium oxide, and citric acid bowel preparation: subanalysis from a randomized, assessor-blinded study. Gastrointest Endosc. 2020;91(6):AB441‐AB442. doi: 10.1016/j.gie.2020.03.2859. | Conference Abstract, etc |
| 550 | Cadoni S, Falt P, Rondonotti E, Radaelli F, Fojtik P, Gallittu P, et al. A randomized, controlled trial comparing air insufflation, water immersion and water exchange for adenoma detection in screening colonoscopy patients. United European Gastroenterology Journal. 2016;4(5):A69. doi: 10.1177/2050640616663688. | Conference Abstract, etc |
| 551 | Cadoni S, Falt P, Sanna S, Argiolas M, Fanari V, Gallittu P, et al. 939 impact of colonoscopy insertion techniques on adenoma detection. Gastrointest Endosc. 2016;83(5):AB189. doi: 10.1016/j.gie.2016.03.197. | Conference Abstract, etc |
| 552 | Cadoni S, Falt P, Sanna S, Argiolas M, Fanari V, Gallittu P, et al. Detection of colonic adenomas using different colonoscopy insertion techniques. Digestive and Liver Disease. 2016;48:e110. | Conference Abstract, etc |
| 553 | Calderwood AH, Jacobson BC. Comprehensive validation of the Boston bowel preparation scale. Gastrointest Endosc. 2009;69(5):AB120-AB121. doi: 10.1016/j.gie.2009.03.126. | Conference Abstract, etc |
| 554 | Calderwood AH, Jacobson BC, Williams JL, Lieberman DA, Schroy PC. Use of the Boston bowel preparation scale to predict risk of missed colon polyps. Gastrointest Endosc. 2015;81(5):AB155. doi: 10.1016/j.gie.2015.03.1921. | Conference Abstract, etc |
| 555 | Capela T, Ferreira AI, Macedo Silva V, Freitas M, Arieira C, Cúrdia Gonçalves T, et al. Modified multiplier SES-CD (MM-SES-CD) and laboratorial Crohn's disease activity parameters: is there any association? Journal of Crohn's and Colitis. 2023;17:i335-i336. doi: 10.1093/ecco-jcc/jjac190.0312. | Conference Abstract, etc |
| 556 | Cardoso DM, Lima M, Filho RF, Coutinho J, Andrade S, Calabria I, et al. Quality assessment in colonoscopy. A focused analysis on adenoma detection rate and photodocumentation. 2024. p. AB622. | Conference Abstract, etc |
| 557 | Carlsen K, Goll R, Elsberg H, Thorkilgaard T, Maagaard L, Wergeland SS, et al. Test characteristics of commonly used clinical and biochemical markers predicting mucosal healing and active inflammation in ulcerative colitis: faecal calprotectin is clearly superior. Journal of Crohn's and Colitis. 2016;10:S398-S399. doi: 10.1093/ecco-jcc/jjw019. | Conference Abstract, etc |
| 558 | Cash BD, Allen C, Sharma P. ID: 3518948 NER1006 1 liter polyethylene glycol (PEG)–based bowel preparation safety profile in patients with mild or moderate renal impairment: a pooled analysis of two phase 3 trials. Gastrointest Endosc. 2021;93(6):AB53. doi: 10.1016/j.gie.2021.03.163. | Conference Abstract, etc |
| 559 | Cash BD, Oneto C, Allen C, Poppers D, Bisschops R. Low risk of hypokalemia in adults treated with the 1 liter polyethylene glycol (PEG)–based bowel preparation NER1006: a pooled analysis of 2 randomized phase 3 trials. Gastrointest Endosc. 2022;95(6):AB71‐AB72. doi: 10.1016/j.gie.2022.04.205. | Conference Abstract, etc |
| 560 | Cash BD, Parasa S, Lorenzen S, Sharma P. Efficacy of 1 L NER1006 bowel preparation for colonoscopy in adults with diabetes: a pooled analysis of two randomized, phase 3 studies. Gastrointest Endosc. 2020;91(6):AB553‐AB554. doi: 10.1016/j.gie.2020.03.3293. | Conference Abstract, etc |
| 561 | Cash BD, Schoenfeld PS, Parasa S, Allen C, Sharma P. A pooled safety analysis of the 1 L polyethylene glycol-based bowel preparation NER1006 in adults with vs without diabetes mellitus: a pooled analyses of 2 randomized, phase 3 studies. American Journal of Gastroenterology. 2020;115(SUPPL):S91. doi: 10.14309/01.ajg.0000702804.37023.9d. | Conference Abstract, etc |
| 562 | Chaaya A, Copeland B, Khoury JPEL, Peikin S, Deitch C. Improving bowel preparation for colonoscopy. American Journal of Gastroenterology. 2010;105:S519. doi: 10.1038/ajg.2010.320-11. | Conference Abstract, etc |
| 563 | Chae MH, Hong WK, Kim HS, Kim JW, Park HJ. Colon transit time test for predicting the poor bowel preparation in patients with constipation. J Gastroenterol Hepatol. 2013;28:566. doi: 10.1111/jgh.12363_2. | Conference Abstract, etc |
| 564 | Chan MY, Chang JI, Birnstein EA, Patel N, Chan LS, Laine LA, et al. Colonoscopy queue position not associated with adenoma detection among GI trainees. Gastroenterology. 2012;142(5):S742. | Conference Abstract, etc |
| 565 | Chen L, Kang X, Pan Y. Individualized interference based on a novel procedure-related prediction model improves adequacy of bowel preparation: a prospectively randomized controlled study. Gastrointest Endosc. 2022;95(6):AB90. doi: 10.1016/j.gie.2022.04.252. | Conference Abstract, etc |
| 566 | Chen TH, Sung CM, Hsu CM, Su MY, Chen PC, Chiu CT. Factors predictive of painful colonoscopy-a multivariate analysis. J Gastroenterol Hepatol. 2009;24:A100-A101. | Conference Abstract, etc |
| 567 | Chen X, Bingxin X, Cheng Y, Qiang Z. The machine learning model for predicting inadequate bowel preparation of diabetic patients: a single-center retrospective study. United European Gastroenterology Journal. 2025;13:244. doi: 10.1002/ueg2.70035. | Conference Abstract, etc |
| 568 | Cheng CL, Tang JH, Hsieh YH, Kuo YL, Fang KC, Tseng CW, et al. Water exchange colonoscopy decreased right colon serrated polyp miss rate: a multicenter prospective randomized tandem study. American Journal of Gastroenterology. 2023;118(10):S188‐S189. | Conference Abstract, etc |
| 569 | Cho JH, Choi JY, Lee SK, Kim HM, Jeon TJ, Kim JH, et al. A randomized prospective study for procedural sequence in same-day upper and lower endoscopy; focus on colonoscopic performance. Gastrointest Endosc. 2012;75(4):AB295. doi: 10.1016/j.gie.2012.03.753. | Conference Abstract, etc |
| 570 | Chua S, Rosenthaler M, Wakim MY, Huang C, Connolly J, Chiu L. Yield of terminal ileal intubation in evaluating iron deficiency anemia. American Journal of Gastroenterology. 2023;118(10):S553-S555. | Conference Abstract, etc |
| 571 | Coe S, Thomas C, Diehl N, Wallace M. A randomized controlled trial of an endoscopic quality improvement program (EQUIP) results in improved detection of colorectal adenomas ACG /Olympus award. American Journal of Gastroenterology. 2011;106:S576. doi: 10.1038/ajg.2011.336_13. | Conference Abstract, etc |
| 572 | Corte CJ, Kim A, Lawler J, Davison S, Singh S, Tee HP, et al. A double-blind randomised placebo controlled trial of hyoscine butylbromide given during colonoscopy to increase polyp detection: an interim analysis. J Gastroenterol Hepatol. 2010;25:A47. doi: 10.1111/j.1440-1746.2010.06452.x. | Conference Abstract, etc |
| 573 | David P, Caterina O, Christopher A, Gregory AC. Efficacy and safety of 1-L NER1006 bowel preparation in patients with inflammatory bowel disease: analysis of 2 phase 3 studies. American Journal of Gastroenterology. 2020;115:S10. doi: 10.14309/01.ajg.0000722944.00010.ea. | Conference Abstract, etc |
| 574 | De Jong M, Degens J, Van Den Heuvel T, Romberg-Camps M, Winkens B, Markus T, et al. Development of a patient reported disease activity score to screen for mucosal inflammation in inflammatory bowel disease. Gastroenterology. 2015;148(4):S451. | Conference Abstract, etc |
| 575 | De Sousa Magalhães R, Boal Carvalho P, Rosa B, Moreira MJ, Cotter J. The prediction of inadequate colon capsule cleansing: a cohort selection guided by CC-clear. Endoscopy. 2021;53(SUPPL 1):S138. doi: 10.1055/s-0041-1724625. | Conference Abstract, etc |
| 576 | DeBerry R, Rahman A, Dubois P, Chung-Faye G, Hayee B. Calprotectin predicts relapse of IBD even in the presence of a 'normal' colonoscopy. United European Gastroenterology Journal. 2014;2(1):A524. doi: 10.1177/2050640614548980. | Conference Abstract, etc |
| 577 | Deberry R, Rahman A, Dubois P, Chung-Faye G, Hayee B. Calprotectin predicts relapse of IBD even in the presence of a 'normal' colonoscopy. Gut. 2015;64:A95-A96. doi: 10.1136/gutjnl-2015-309861.195. | Conference Abstract, etc |
| 578 | Dhillon A, Alshankiti S, Khorasani A, Sultanian R, Sandha GS, Ali KV, et al. L-menthol during colonoscopy for adenoma detection in an intermediate risk patient population: a double-blind, randomized controlled trial. Gastrointest Endosc. 2018;87(6):AB132. | Conference Abstract, etc |
| 579 | Dhillon HS, Healy P, Tabbara D, Mohamedrashed M, Nedumannil L, Walia N, et al. “Cracking the code: predictors and strategies for optimizing bowel preparation – a large single-center retrospective study”. 2025. p. S337-S338. | Conference Abstract, etc |
| 580 | Díaz Alcázar MDM. Which factors might help to predict a capsule endoscopy exploration with adequate cleanliness? United European Gastroenterology Journal. 2025;13:901. doi: 10.1002/ueg2.70036. | Conference Abstract, etc |
| 581 | Dilmaghani S, Coelho-Prabhu N. Role of artificial intelligence in colonoscopy: a literature review of the past, present, and future directions. Tech Innov Gastrointest Endosc. 2023;25(4):399-412. doi: 10.1016/j.tige.2023.03.002. | Conference Abstract, etc |
| 582 | Do A, Kakkar A, Calderwood AH, Jacobson BC. Lower adenoma detection rates in afternoon colonoscopies may be due to rushing and not fatigue. Gastrointest Endosc. 2012;75(4):AB282. doi: 10.1016/j.gie.2012.03.717. | Conference Abstract, etc |
| 583 | Dominitz JA, Robertson DJ, Beed A, Ahnen D, Boardman KD, Del Curto B, et al. Baseline findings on screening colonoscopy in a multicenter study of colorectal cancer screening. Gastroenterology. 2019;156(6):S‐149‐S‐150. doi: 10.1016/S0016-5085(19)37162-8. | Conference Abstract, etc |
| 584 | Dominitz JA, Robertson DJ, Beed A, Ahnen D, Boardman KD, Del Curto B, et al. Serrated polyp findings on screening colonoscopy in a multi-center study of colorectal cancer screening. Gastrointest Endosc. 2020;91(6):AB425‐AB426. doi: 10.1016/j.gie.2020.03.2494. | Conference Abstract, etc |
| 585 | Dornblaser DW, Tafani E, Liang PS. Correlating quantitative fecal immunochemical test (FIT) values with histologic findings on follow-up colonoscopy. Gastroenterology. 2019;156(6):S-814. doi: 10.1016/S0016-5085(19)38986-3. | Conference Abstract, etc |
| 586 | Drügg Hahn G, Flores C, Afif W, Wild G, Bitton A, Lakatos PL, et al. Faecal calprotectin as a predictor of endoscopic and histologic remission in patients with ulcerative colitis. Journal of Crohn's and Colitis. 2024;18:i843-i844. doi: 10.1093/ecco-jcc/jjad212.0542. | Conference Abstract, etc |
| 587 | Dwyer JP, Braithwaite A, Secomb R, Brown G. Predictors and outcomes of poor bowel preparation at colonoscopy. J Gastroenterol Hepatol. 2017;32:19-20. | Conference Abstract, etc |
| 588 | Early D, Larue S, Bhat T, Gyawali P, Hollander T, Kodali D, et al. The impact of trendelenberg positioning on ease of colonoscope insertion. American Journal of Gastroenterology. 2016;111:S145‐S146. doi: 10.1038/ajg.2016.354. | Conference Abstract, etc |
| 589 | Elvas L, Brito D, Areia M, Carvalho R, Alves S, Saraiva S, et al. Impact of personalized patient education on bowel preparation for colonoscopy-prospective randomized controlled trial. United European Gastroenterology Journal. 2015;3(5):A375‐A376. doi: 10.1177/2050640615601623. | Conference Abstract, etc |
| 590 | Enestvedt B, Brian Fennerty M, Eisen GM. MiraLAX, golytely, adenoma detection and bowel prep quality: is thinner better? Gastrointest Endosc. 2011;73(4):AB384. doi: 10.1016/j.gie.2011.03.851. | Conference Abstract, etc |
| 591 | Enestvedt B, Fennerty MB, Eisen GM. Golytely vs miralax: is there is difference in the adenoma detection rate? Gastrointest Endosc. 2011;73(4):AB124. doi: 10.1016/j.gie.2011.03.045. | Conference Abstract, etc |
| 592 | Epstein M, Manning J, Allen C, Alvarez-Gonzalez MA. Same-day morning-only dosing of 1L NER1006, a polyethylene glycol bowel preparation, nearly doubles the chance of high-quality cleansing versus standard 2L polyethylene glycol and ascorbate. Gastrointest Endosc. 2022;95(6):AB87. doi: 10.1016/j.gie.2022.04.247. | Conference Abstract, etc |
| 593 | Epstein M, Shah ED, Allen C, Bisschops R. NER1006, a 1 liter polyethylene glycol–based bowel preparation, is an independent predictor of adequate and high-quality cleansing success in adults undergoing colonoscopy: a pooled analysis of 2 randomized phase 3 trials. Gastrointest Endosc. 2022;95(6):AB47‐AB48. doi: 10.1016/j.gie.2022.04.152. | Conference Abstract, etc |
| 594 | Epstein MS, Lorenzen S, Franklin H, Cash BD. Electrolyte shifts of NER1006 bowel preparation for colonoscopy in the elderly are transient and well-tolerated. American Journal of Gastroenterology. 2019;114:S306‐S307. doi: 10.14309/01.ajg.0000591652.20290.57. | Conference Abstract, etc |
| 595 | Fayad NF, Imperiale TF, El-Jawad KA, Shin A, Shah S, Lane KA, et al. Association of body-mass index and split bowel preparation quality-interim analysis. Gastrointest Endosc. 2012;75(4):AB297. doi: 10.1016/j.gie.2012.03.760. | Conference Abstract, etc |
| 596 | Feng LN, Guan JL, Dong RN, Zhao K, Zhang MY, Xia SH, et al. Risk factors for inadequate bowel preparation before colonoscopy: a meta-analysis. Journal of Evidence Based Medicine. 2024;17(2):341-350. doi: 10.1111/jebm.12607. | Conference Abstract, etc |
| 597 | Ferreira LE, Dos Santos CE, Ferreira VHP, Silva VS, Carvalho BE, Ferreira JP, et al. Can new image-enhancing technologies improve adenoma detection rates in cancer screening colonoscopies? A randomized clinical trial. Gastrointest Endosc. 2020;91(6):AB232‐AB233. doi: 10.1016/j.gie.2020.03.1769. | Conference Abstract, etc |
| 598 | Ferreiro R, Barreiro-De Acosta M, Vallejo-Senra N, De La Iglesia Garcia D, Uribarri-González L, Baston-Rey I, et al. Accuracy of a rapid fecal calprotectin test as a predictor of mucosal healing in patients with ulcerative colitis (UC). Gastroenterology. 2015;148(4):S451-S452. | Conference Abstract, etc |
| 599 | Frazzoni L, Spada C, Radaelli F, Mussetto A, Fabbri C, Laterza L, et al. 1-liter polyethilene glycol (1L-PEG) bowel preparation vs 4L-PEG for colon cleansing among inpatients: a propensity score matching analysis from a large multicenter prospective cohort. Digestive and Liver Disease. 2020;52:S141. doi: 10.1016/S1590-8658(20)30810-0. | Conference Abstract, etc |
| 600 | Gandhi K, Daskalakis C, Tofani C, Sokach C, Patel D, Kastenberg D. Patient predictors for inadequate bowel preparation in colonoscopy: a meta-analysis. American Journal of Gastroenterology. 2016;111:S92-S92. doi: 10.14309/00000434-201610001-00190. | Conference Abstract, etc |
| 601 | Gao H, Liu C, Yuan X, Lu H, Xia Y, Zhang Z, et al. Face-to-face instruction and personalized bowel preparation regimens improve quality of preparation for inpatient colonoscopy: a randomized controlled trial. Gut. 2021;70(SUPPL 2):A126‐A127. doi: 10.1136/gutjnl-2021-IDDF.144. | Conference Abstract, etc |
| 602 | Georgostathis D, Deng L, Martinez M, Al-Ani F, Aytaman Y, Batool A, et al. Positive correlation between methane breath levels and adequate bowel preparation for colonoscopy. American Journal of Gastroenterology. 2015;110:S596-S597. | Conference Abstract, etc |
| 603 | Geyer M, Leiner D. Comparison of new colonoscopy devices with standard forward viewing (SFV) high definition colonoscopes in daily practice. Swiss Medical Weekly. 2018;148:8S. | Conference Abstract, etc |
| 604 | Geyer M, Leiner D, Bannwart F. Comparison of newer colonoscopy devices with standard forward viewing (SFV) high-definition colonoscopes in daily practice. United European Gastroenterology Journal. 2018;6(8):A396‐A397. doi: 10.1177/2050640618792819. | Conference Abstract, etc |
| 605 | Gluskin AB, Keswani RN, Johnston ER, Gregory DL, Cyrus R, Werth L, et al. The nursing bowel preparation assessment tool is a reliable and accurate inpatient colonoscopy clinical decision support tool. Gastrointest Endosc. 2016;83(5):AB212-AB213. | Conference Abstract, etc |
| 606 | Glyn T, Lam D, D'Souza B. Randomized controlled trial of probiotic use for post-colonoscopy symptoms. Diseases of the colon and rectum. 2017;60(6):e401‐e402. doi: 10.1097/DCR.0000000000000854. | Conference Abstract, etc |
| 607 | Grigg E, Schubert M, Hall J, Sridhar S, Chamberlain S. Efficacy of lubiprostone used with polyethylene glycol to enhance colonoscopy preparation quality in diabetic patients: a randomized single-blind controlled trial. American Journal of Gastroenterology. 2010;105:S505. doi: 10.1038/ajg.2010.320-11. | Conference Abstract, etc |
| 608 | Guardiola Arevalo A, Granja Navacerrada A, Garcia-Alonso FJ, Bernal Checa P, Hernandez Tejero M, Bonillo D, et al. Randomized clinical trial evaluating the effect of a visual educational booklet on the preparation of colonoscopies in hospitalized patients. United European Gastroenterology Journal. 2017;5(5):A678‐A679. doi: 10.1177/2050640617725676. | Conference Abstract, etc |
| 609 | Guo CG, Ji R, Li YY, Li LX, Zuo XL, Li YQ. Improved detection rate of proximal adenomas with segmental re-examination in the proximal colon: a randomized controlled trial. Journal of Digestive Diseases. 2017;18:62. doi: 10.1111/1751-2980.12518. | Conference Abstract, etc |
| 610 | Hadlock S, Paszat L, Rabeneck L, Wilton D, Sutradhar R, Tinmouth JM. Colonoscopy completion in open access procedures in Ontario: a nested case control study. Gastrointest Endosc. 2009;69(5):AB309. doi: 10.1016/j.gie.2009.03.868. | Conference Abstract, etc |
| 611 | Haider SA, Bopp PA, Genere JR. Risk factors for unplanned cancellations of colonoscopy appointments: a prospective study in a tertiary academic center. American Journal of Gastroenterology. 2023;118(10):S304-S305. | Conference Abstract, etc |
| 612 | Halonen J, Cheriyamkunnel SJ, Amlani B. Shorter colonoscopy procedure times in adequately cleansed patients who also attain Harefield cleansing scale high-quality colon segments. United European Gastroenterology Journal. 2021;9(SUPPL 8):821. doi: 10.1002/ueg2.12144. | Conference Abstract, etc |
| 613 | Hassan C, Ratanachuek T, Ge N, Kashin SV, Neumann H. A comparison of performance parameters for colonoscopy for using different endoscopy systems: a multicenter randomized controlled trial. United European Gastroenterology Journal. 2018;6(8):A389. doi: 10.1177/2050640618792819. | Conference Abstract, etc |
| 614 | Hassan C, Ratanachu-ek T, Nan G, Kashin SV, Neumann H. Multicenter randomized controlled trial assessing performance parameters of different endoscopy systems. Gastrointest Endosc. 2019;89(6):AB507. doi: 10.1016/j.gie.2019.03.842. | Conference Abstract, etc |
| 615 | Hernandez PV, Horsley-Silva JL, Snyder DL, Baffy N, Atia M, Koepke L, et al. Effect of bowel preparation volume size in inpatient colonoscopy. Results of a prospective, randomized, comparative pilot study. American Journal of Gastroenterology. 2019;114:S101‐S102. doi: 10.14309/01.ajg.0000590192.04241.e5. | Conference Abstract, etc |
| 616 | Heron V, Martel M, Bessissow T, Chen Y, Desilets E, Dube C, et al. Comparison of the Boston bowel preparation scale with an auditable application of the US multi-society task force guidelines. J Can Assoc Gastroenterol. 2018;1:86-87. doi: 10.1093/jcag/gwy008.052. | Conference Abstract, etc |
| 617 | Heron V, Martel M, Bessissow T, Chen YI, Désilets E, Dube C, et al. Validation of the Montreal bowel preparation scale: an auditable application of the MSTF guidelines. American Journal of Gastroenterology. 2016;111:S160. doi: 10.1038/ajg.2016.354. | Conference Abstract, etc |
| 618 | Higashimori A, Maeda N, Fujiwara Y. Does linaclotide have high predictability of defecation? A post-hoc analysis of APPLE trial. Gut. 2024;73:A380. doi: 10.1136/gutjnl-2024-IDDF.341. | Conference Abstract, etc |
| 619 | Hookey L, Bertiger G, Seifu Y, Ayala J, Brogadir S, Dubcenco E. A randomized, assessor-blinded, multicenter study demonstrates the efficacy and safety of a new ready-to-drink low volume bowel preparation prior to colonoscopy. Gastroenterology. 2018;154(6):S‐1368. doi: 10.1016/S0016-5085(18)34469-X. | Conference Abstract, etc |
| 620 | Hookey L, Johnson KL, Ando M, Boules M, Stong L, Bertiger G. Impact of body mass index on the efficacy and safety of a ready-to-drink low volume bowel preparation: subgroup analysis from a randomized, assessor-blinded, multicenter study. Gastrointest Endosc. 2019;89(6):AB519‐AB520. doi: 10.1016/j.gie.2019.03.872. | Conference Abstract, etc |
| 621 | Hookey L, Johnson KL, Ando M, Chacar C, Stong L, Bertiger G. Efficacy and safety of a ready-to-drink low volume bowel preparation in adults 65 years and older: subgroup analysis from a randomized, assessor-blinded, multicenter study. Gastrointest Endosc. 2019;89(6):AB515‐AB516. doi: 10.1016/j.gie.2019.03.863. | Conference Abstract, etc |
| 622 | Humm SD, Singla A, Jacobson BC. An attempt to validate the “comprehensive colonoscopy documentation” quality measure. Gastroenterology. 2012;142(5):S231. | Conference Abstract, etc |
| 623 | Hunleth J, Hughes BO, Smith S, McCray N, Bowen D, James A. Cost and consequences: ways in which cost is a barrier to colon cancer screening. Cancer Epidemiology Biomarkers and Prevention. 2015;24(10). doi: 10.1158/1538-7755.DISP14-A50. | Conference Abstract, etc |
| 624 | Hyun YS, Han DS, Bae JH, Park HS, Ahn SB, Kim TY, et al. A randomized tandem colonoscopy trial of transparent hood attached versus conventional examination to compare polyp miss rate. Gastrointest Endosc. 2010;71(5):AB332. doi: 10.1016/j.gie.2010.03.864. | Conference Abstract, etc |
| 625 | Ianiro G, Masucci L, Valerio L, Nagel D, D'Aversa F, Poto R, et al. Fecal microbiota transplantation for recurrent C. difficile infection: analysis of factors associated with the need for multiple fecal infusions. United European Gastroenterology Journal. 2016;4(5):A96‐A97. doi: 10.1177/2050640616663688. | Conference Abstract, etc |
| 626 | Idigoras Rubio I, Arana-Arri E, Bilbao I, Fernandez J, Fernandez I, Martinez-Indart L, et al. Predictors related to colonoscopy complications in an organised colorectal cancer screening programme with immunochemical faecal occult blood test. United European Gastroenterology Journal. 2016;4(5):A393-A394. doi: 10.1177/2050640616663689. | Conference Abstract, etc |
| 627 | Imaz-Ayo N, Idigoras Rubio I, Portillo Villares MI, Bilbao Iturribarria I, Fernández Landa MJ, Guierrez-Ibarluzea I, et al. Which factors are related to a risk of developing a perforation after a colonoscopy in an organised population-based colorectal cancer screening programme? A nested case-control study. United European Gastroenterology Journal. 2018;6(8):A403. doi: 10.1177/2050640618792819. | Conference Abstract, etc |
| 628 | Jacobs J, Tong M, Ruegger P, Li D, Haritunians T, Fleshner P, et al. The microbiome of the colonic mucosal-luminal interface in Crohn's disease patients in endoscopic remission. Inflammatory Bowel Diseases. 2017;23:S78. doi: 10.1097/01.MIB.0000512752.22740.1a. | Conference Abstract, etc |
| 629 | Jacobs J, Tong M, Ruegger P, Li D, Haritunians T, Fleshner P, et al. Crohn's disease, obesity, and high Crohn's disease genetic risk are associated with parallel changes in the microbiome of the cecal and sigmoid mucosal-luminal interface. Gastroenterology. 2017;152(5):S111. | Conference Abstract, etc |
| 630 | Jaoua H, Zghidi SM, Ammar N, Ben Fadhel K. Patient controlled sedation (PCS) during colonoscopy. Regional Anesthesia and Pain Medicine. 2010;35(5):E113. doi: 10.1097/AAP.0b013e3181f3582c. | Conference Abstract, etc |
| 631 | Javidi DJ, Butterly LF, Anderson J. Risk factors for left and right sided advanced colorectal neoplasia in young adults. Gastroenterology. 2025;169(1):S-366. doi: 10.1016/S0016-5085(25)01778-0. | Conference Abstract, etc |
| 632 | Jia H, Koo M, Hsieh YH, Tseng CW, Hu CT, Zhang L, et al. Factors associated with adenoma detection in propofol-sedated patients. Gastrointest Endosc. 2018;87(6):AB166. | Conference Abstract, etc |
| 633 | Jia H, Pan Y, Leung FW, Guo X, Wu K. Water exchange method significantly improves adenoma detection rate: a multi-center, randomized controlled trial. Gastrointest Endosc. 2016;83(5):AB146‐AB147. | Conference Abstract, etc |
| 634 | John GK, Thuluvath AJ, Gupta E, Carrier H, Ahuja NK, Stein E. Patient reported stool color and perception of bowel preparation predicts success of outpatient bowel preparation in an urban tertiary care setting. Gastroenterology. 2018;154(6):S-143. doi: 10.1016/S0016-5085(18)30896-5. | Conference Abstract, etc |
| 635 | Johnston ER, Keswani RN, Cyrus R, Werth L, Grande D, Yadlapati R. The nursing bowel preparation assessment tool (NBPAT) is highly predictive of inpatient bowel preparation adequacy: a prospective pilot study. Gastrointest Endosc. 2015;81(5):AB154. doi: 10.1016/j.gie.2015.03.1918. | Conference Abstract, etc |
| 636 | Jukic I, Vukovic J. Bowel preparation for colonoscopy in patients with diabetes mellitus-a gap we have to bridge: a review. J Clin Med. 2025;14(10). doi: 10.3390/jcm14103336. | Conference Abstract, etc |
| 637 | Kahn SA, Goeppinger SR, Vaughn BP, Moss AC, Rubin DT. Tolerability of colonoscopic fecal microbiota transplantation in IBD. Gastroenterology. 2014;146(5):S-581. doi: 10.1016/S0016-5085(14)62103-X. | Conference Abstract, etc |
| 638 | Kale A, Kuruthukulangara M, Garg L, Satai M, Patwardhan N, Jain G, et al. Development and validation of ‘ColonPrep score’ to assess adequacy of bowel preparation prior to colonoscopy. Gastrointest Endosc. 2023;97(6):AB201-AB202. doi: 10.1016/j.gie.2023.04.338. | Conference Abstract, etc |
| 639 | Karnes WE, Ninh A, Dao T, Requa J, Samarasena JB. Unambiguous real-time scoring of bowel preparation using artificial intelligence. Gastrointest Endosc. 2018;87(6):AB258. | Conference Abstract, etc |
| 640 | Khaing M, Lin L, Boyd P, Croese J, Peter Kini G, Hodgson R, et al. Clinical service and quality improvement: can the immunochemical fecal occult blood test for bowel cancer screening be safely deferred for 5 years after a colonoscopy? J Gastroenterol Hepatol. 2019;34:211. doi: 10.1111/jgh.14806. | Conference Abstract, etc |
| 641 | Khaing MM, Lin L, Rahman T, Peter-Kini G, Croese J, Hodgson R, et al. Clinical service and quality improvement: can iFOBT (immunochemical faecal occult blood test) for bowel cancer screening be safely deferred for five years after a colonoscopy? J Gastroenterol Hepatol. 2019;34:104. doi: 10.1111/jgh.14879. | Conference Abstract, etc |
| 642 | Khaing MM, Lin L, Rahman T, Peter-Kini G, Hodgson R, Ea V, et al. Can iFOBT (immunochemical faecal occult blood test) for bowel cancer screening be safely deferred for five years after a colonoscopy? American Journal of Gastroenterology. 2020;115(SUPPL):S134. doi: 10.14309/01.ajg.0000703152.13142.fc. | Conference Abstract, etc |
| 643 | Khan K, Merali A, Forbes N, Fergani H, Ganguli S, Jalali S, et al. Colonoscopy preparation optimization for inpatients (COIN study). A randomized controlled trial comparing 4L PEGlyte to regular dose Pico Salax and split dose Pico Salax for colonoscopy bowel preparation in hospitalized patients. J Can Assoc Gastroenterol. 2018;1:347. doi: 10.1093/jcag/gwy009.237. | Conference Abstract, etc |
| 644 | Khan K, Nehvi A, Khan M, Fergani H, Ganguli S, Jalali S, et al. Baseline bowel routine and other predictors of bowel preparation in patients undergoing out-patient colonoscopy; results from a prospective single-centre study. J Can Assoc Gastroenterol. 2019;2:455-456. doi: 10.1093/jcag/gwz006.232. | Conference Abstract, etc |
| 645 | Kim A, Gill R, Lim P, Dahlenburg L, Tamilarasan G, Kaffes AJ. Evaluation of resect and discard strategy in clinical practice at a single tertiary centre: a prospective cohort study of diminutive polyps. J Gastroenterol Hepatol. 2013;28:19. doi: 10.1111/jgh.12365-3. | Conference Abstract, etc |
| 646 | Kim BJ, Kim JW, Choi CH, Chang SK, Kim SJ. Proper interval to second colonoscopy after preparation failure in the first procedure-prospective randomized trial. United European Gastroenterology Journal. 2018;6(8):A185‐A186. doi: 10.1177/2050640618792819. | Conference Abstract, etc |
| 647 | Kim BJ, Kim SJ, Kang KJ, Kim JG. Prospective comparison of interval to second colonoscopy after preparation failure in the first procedure. American Journal of Gastroenterology. 2012;107:S737. doi: 10.1038/ajg.2012.277. | Conference Abstract, etc |
| 648 | Kim EY, Ou G, Lakzadeh P, Tong J, Hemmati I, Enns RA, et al. A randomized controlled trial comparing dynamic patient position change during colonoscope withdrawal to usual care for the detection of colonic polyps. Gastrointest Endosc. 2013;77(5):AB502. | Conference Abstract, etc |
| 649 | Kim KO, Jeong DE, Hwang MJ, Lee SH, Jang BI, Kim TN. Risk factors of inadequate bowel preparation before colonoscopy in outpatients clinics: a single center prospective study. Gastrointest Endosc. 2016;83(5):AB228-AB228. doi: 10.1016/j.gie.2016.03.304. | Conference Abstract, etc |
| 650 | Kligman E, Kahi CJ, Li W, Eckert G. ADR in asymptomatic patients with positive fecal immunochemical tests. Gastrointest Endosc. 2017;85(5):AB119. | Conference Abstract, etc |
| 651 | Kroijer R, Baatrup G. Booster medication to achieve capsule excretion in colon capsule endoscopy, a randomized controlled trial on three regimens. Colorectal Disease. 2018;20:68. doi: 10.1111/(ISSN)1463-1318. | Conference Abstract, etc |
| 652 | Kurlander JE, Saini SD, Lipson R, Menees SB, Sultan S, Kokaly AN, et al. Development and validation of a machine-learning model to predict adequate bowel preparation in a sample of US veterans. Gastroenterology. 2019;156(6):S-263-S-264. doi: 10.1016/S0016-5085(19)37465-7. | Conference Abstract, etc |
| 653 | Laiyemo A, Burnside C, Laiyemo M, Kibreab A, Begum R, Scott V, et al. The association of beverage intake preference with bowel preparation laxative taste preference: results of public taste tests. American Journal of Gastroenterology. 2013;108:S486. doi: 10.1038/ajg.2013.268. | Conference Abstract, etc |
| 654 | Laiyemo AO, Burnside C, Laiyemo MA, Owusu J, Pessu O, Ajanaku F, et al. Personalizing the choice of bowel preparation laxative for your patients: results of public taste tests. Gastrointest Endosc. 2013;77(5):AB514. | Conference Abstract, etc |
| 655 | Lall V, Ismail AGM, Ayonrinde OT. Patient and procedure characteristics of patients with conventional adenomas and sessile serrated lesions in a general colonoscopy population. Journal of Gastroenterology and Hepatology (Australia). 2020;35(SUPPL 1):215. doi: 10.1111/jgh.15277. | Conference Abstract, etc |
| 656 | Lasa JS, Senderovsky MT, Fanjul I, Moore R, Dima G, Peralta AD, et al. Patient-related factors affecting bowel preparation prior to colonoscopy. Gastrointest Endosc. 2014;79(5):AB214-AB215. doi: 10.1016/j.gie.2014.02.305. | Conference Abstract, etc |
| 657 | Leal T, Mendes SDS, Antunes PB, Gonçalves M, Arroja B, Gonçalves R. Predictive factors of adequate bowel preparation in hospitalized patients. Dig Liver Dis. 2021;53(2):256-257. doi: 10.1016/j.dld.2020.11.009. | Conference Abstract, etc |
| 658 | Lee DW, Koo JS, Choe JW, Kim SY, Hyun JJ, Jung YK, et al. Comparison of colonoscopic quality indicators for predicting adenoma miss rate. Gastrointest Endosc. 2017;85(5):AB537‐AB538. | Conference Abstract, etc |
| 659 | Lee HS. How can you predict the adequate colon preparation? Gastroenterological Endoscopy. 2012;54:1151. | Conference Abstract, etc |
| 660 | Lee J, Kim TH, Park YE, Park J, Kim TO. Efficacy of mosapride citrate with a split dose of PEG+ascorbic acid for bowel preparation in elderly patients. Turkish Journal of Gastroenterology. 2019;30:S308‐S309. doi: 10.5152/tjg.2019.050919. | Conference Abstract, etc |
| 661 | Lee LHA, Smale M, Humphris J. 543: a randomised controlled trial assessing the clinical utility of extended preparation at index colonoscopy (EPIC) for high risk patients using a novel and validated scoring system. Gastroenterology. 2025;169(1):S‐135‐S‐136. doi: 10.1016/S0016-5085(25)01255-7. | Conference Abstract, etc |
| 662 | Lee YJ, Kim ES, Cho KB, Park KS, Kim KO, Lee HS, et al. The impact of additional oral preparation on the quality of bowel preparation for colonoscopy. J Gastroenterol Hepatol. 2018;33:163. doi: 10.1111/jgh.14482. | Conference Abstract, etc |
| 663 | Lee YJ, Lee SY, Cho KB, Kim ES, Park KS, Kim KO, et al. The impact of additional oral preparation on the quality of bowel preparation for colonoscopy. Endoscopy. 2019;51(4):S57. doi: 10.1055/s-0039-1681337. | Conference Abstract, etc |
| 664 | Lei II, O'Connell N, Adu-Darko M, Suresh V, McDonnell K, Newville J, et al. Iron deficiency anaemia pathway: leveraging colonoscopy findings to predict panenteric capsule endoscopy outcomes with FIT as a triage tool: interim analysis of the CLEAR IDA multicentre study. 2025. | Conference Abstract, etc |
| 665 | Lei II, Parisi I, Bhandare A, Perez F, Lee T, Shehkar C, et al. From capsule to scope: predicting colon capsule endoscopy conversion to optical endoscopy - insights from the CESCAIL study. Gut. 2025;74:A269-A270. doi: 10.1136/gutjnl-2025-BSG.428. | Conference Abstract, etc |
| 666 | Leitão C, Ribeiro H, Pinto J, Caldeira A, Sousa R, Tristan J, et al. Predictors of inadequate bowel preparation for colonoscopy: a prospective study. United European Gastroenterology Journal. 2015;3(5):A193. doi: 10.1177/2050640615601623. | Conference Abstract, etc |
| 667 | Leon MA, Morla LF, Romero CH, Leon CU, Neira MF, Veloz MJ, et al. CO2 insufflation vs air insufflation for elective colonoscopy at two reference centers in Ecuador. American Journal of Gastroenterology. 2020;115(SUPPL):S144. doi: 10.14309/01.ajg.0000703220.85405.ff. | Conference Abstract, etc |
| 668 | Leung F, Chen Y, Cheung R, Dixit V, Elashoff D, Friedland S, et al. Patient and procedural factors that impact on real time maximum insertion pain in unsedated water exchange colonoscopy - a single (patient) blinded multi-site, randomized controlled trial in veterans. Gastrointest Endosc. 2024;99(6):AB460‐AB461. doi: 10.1016/j.gie.2024.04.1440. | Conference Abstract, etc |
| 669 | Loo KF, Cock C, Young G, Fraser R, Symonds E. Predicting advanced colorectal neoplasia using quantitative fecal immunochemical testing in symptomatic patients in the Australian population. Journal of Gastroenterology and Hepatology (Australia). 2020;35(SUPPL 1):178-179. doi: 10.1111/jgh.15273. | Conference Abstract, etc |
| 670 | Lu T, Khan SI, Hookey LC. Predictive overbooking to prevent endoscopy clinic nonattendance: model development. J Can Assoc Gastroenterol. 2019;2:414-415. doi: 10.1093/jcag/gwz006.211. | Conference Abstract, etc |
| 671 | Lucendo AJ, Molina-Infante J. Treatment of eosinophilic esophagitis with diets. Minerva Gastroenterologica E Dietologica. 2020;66(2):124-135. doi: 10.23736/S1121-421X.19.02634-5. | Conference Abstract, etc |
| 672 | Luo H, Pan Y, Zhang L, Liu X, Leung FW, Guo X, et al. Water exchange enhanced cecal intubation in potentially difficult colonoscopy-unsedated patients with prior abdominal or pelvic surgery: a prospective randomized, controlled trial. Gastrointest Endosc. 2013;77(5):AB498‐AB499. | Conference Abstract, etc |
| 673 | Luo H, Pan YL, Guo XG. Bowel preparation score identifies patients with inadequate bowel preparation before colonoscopy. Journal of Digestive Diseases. 2014;15:54. doi: 10.1111/1751-2980.12194. | Conference Abstract, etc |
| 674 | Ma C, Lumb R, Foshaug R, Dang TNT, Verma S, Huang V, et al. Fecal immunochemical testing and fecal calprotectin predict mucosal healing in inflammatory bowel disease: a prospect study. Gastroenterology. 2016;150(4):S555-S556. | Conference Abstract, etc |
| 675 | Madhoun MF, Chaudrey K, Chisholm SS, Ahmed A, Frost B, Tierney WM. Efficacy and tolerability of various bowel preparations in patients with diabetes: a randomized controlled trial. Gastroenterology. 2017;152(5):S548. | Conference Abstract, etc |
| 676 | Maeda N, Higashimori A, Maruyama H, Ominami M, Fukunaga S, Otani K, et al. 1211: Efficacy of linaclotide as a bowel cleansing agent in patients with chronic constipation: a severity-based analysis of the APPLE trial. Gastroenterology. 2025;169(1):S‐295. doi: 10.1016/S0016-5085(25)01624-5. | Conference Abstract, etc |
| 677 | Maeda N, Higashimori A, Nakatani M, Jinnai K, Kin D, Sugita N, et al. Efficacy and tolerability of 1-L polyethylene glycol plus ascorbic acid with senna versus 2-L polyethylene glycol plus ascorbic acid for colonoscopic bowel preparation: a prospective, randomized, investigator-blinded trial. Gastrointest Endosc. 2023;97(6):AB266. doi: 10.1016/j.gie.2023.04.438. | Conference Abstract, etc |
| 678 | Maeda N, Higashimori A, Nakatani M, Yamamoto I, Yanagida T, Kin D, et al. Does linaclotide have high predictability of defecation? A post-hoc analysis of APPLE trial. Gastroenterology. 2024;166(5):S-784. doi: 10.1016/S0016-5085(24)02284-4. | Conference Abstract, etc |
| 679 | Maekawa H, Hiwatashi N, Kinouchi Y, Noguchi M, Nagase K, Watanabe H, et al. Bowel preparation of colonoscopy and barium enema for inflammatory bowel disease in our department. Therapeutic Research. 1996;17(SUPPL. 2):109-112. | Conference Abstract, etc |
| 680 | Magalhaes R, Boal Carvalho P, Rosa B, Moreira MJ, Cotter J. Predicting an inadequate colon capsule cleansing: a study approaching predictive factors. United European Gastroenterology Journal. 2020;8(8 SUPPL):824. doi: 10.1177/2050640620927345. | Conference Abstract, etc |
| 681 | Mahmood S, Farooqui SM, Tajammal R, Nusrat S, Madhoun MF. Predictors of inadequate bowel preparation for colonoscopy: a systematic review and meta-analysis. Gastroenterology. 2017;152(5):S731-S731. doi: 10.1016/S0016-5085(17)32543-X. | Conference Abstract, etc |
| 682 | Maida M, Vassallo R, Zullo A, Venezia L, Facciorusso A. Impact of bowel cleansing on adenoma detection rate: post hoc analysis of a randomised clinical trial. Digestive and Liver Disease. 2024;56:S275‐S276. doi: 10.1016/S1590-8658(24)00657-1. | Conference Abstract, etc |
| 683 | Maida M, Vassallo R, Zullo A, Venezia L, Facciorusso A. Exploring gender differences in quality of bowel preparation for colonoscopy: post hoc analysis of a randomised clinical trial. Digestive and Liver Disease. 2024;56:S227‐S228. doi: 10.1016/S1590-8658(24)00571-1. | Conference Abstract, etc |
| 684 | Mandava S, Abbas A, Rani A, Gudleski G. Quality improvement of colonoscopy at a tertiary care academic medical center. American Journal of Gastroenterology. 2014;109:S654. doi: 10.1038/ajg.2014.280. | Conference Abstract, etc |
| 685 | Manes G, Repici A, Hassan C, Battaglia G, Virgilio C, Muscatiello N, et al. Split dosage and other predictors of quality of bowel cleansing in patients assuming a “very low volume” preparation for colonoscopy: an Italian multicenter study using the association of sodium picosulphate and magnesium citrate. Digestive and Liver Disease. 2013;45:S194‐S195. | Conference Abstract, etc |
| 686 | Mangira D, Ket S, Braithwaite A, Dwyer J, Noguchi M, Brown G. Does addition of Movicol to standard bowel preparation improve the quality of bowel preparation in patients undergoing routine colonoscopy? Journal of Gastroenterology and Hepatology (Australia). 2017;32:166. doi: 10.1111/jgh.13896. | Conference Abstract, etc |
| 687 | Mankaney GN, Burke CA, Scott TA, Ando M, Stong L, Boules M. 434 Safety and efficacy of a low volume bowel preparation in patients with pre-existing renal dysfunction: subanalysis from a phase 3 study of ready-to-drink sodium picosulfate, magnesium oxide, and citric acid preparation. Gastrointest Endosc. 2020;91(6):AB42‐AB43. doi: 10.1016/j.gie.2020.03.289. | Conference Abstract, etc |
| 688 | Manning J, Amlani B, Hassan C, Ponchon T. High-quality colon cleansing improves real-world identification of highrisk patients: post hoc analysis of randomised clinical trials using two validated cleansing scales. Endoscopy. 2019;51(4):S152. doi: 10.1055/s-0039-1681618. | Conference Abstract, etc |
| 689 | Manoj N, Abdolmaleki D, Miller M, Woolfson J, Zizzo A. Method of instruction delivery does not affect quality of bowel preparation for pediatric colonoscopy. Journal of Pediatric Gastroenterology and Nutrition. 2022;75:S22-S23. | Conference Abstract, etc |
| 690 | Mansvelders MS, Kleif J, Kahn CL, Andersen MR, Feng F, Jackson L, et al. Use of fecal immunochemical testing and blood-based biomarkers for colorectal cancer risk prediction in symptomatic patients referred for colonoscopy. Gastroenterology. 2024;166(5):S-979. doi: 10.1016/S0016-5085(24)02725-2. | Conference Abstract, etc |
| 691 | Marquez EO, Chiplunker AJ, Colditz GA, Lobb R, Wang JS. Predictive validity of the Boston bowel preparation score within a hospital network comprised of various practice settings. Gastrointest Endosc. 2015;81(5):AB222. doi: 10.1016/j.gie.2015.03.1289. | Conference Abstract, etc |
| 692 | Modi R, Hood R, Dasani D, Blandon C, Kumar S. Abstract PR001: Risk prediction modeling for colorectal adenomas in persons under age 50: a risk-stratified approach to early onset colorectal cancer prevention. Clinical Cancer Research. 2025;31(23). doi: 10.1158/1557-3265.EARLYONSETCA25-PR001. | Conference Abstract, etc |
| 693 | Modi R, Hood R, Dasani D, Blandon C, Kumar S. Risk prediction modeling for colorectal adenomas in persons under age 50: a risk-stratified approach to early onset colorectal cancer prevention. AACR Special Conference in Cancer Research: The Rise in Early-Onset Cancers - Knowledge Gaps and Research Opportunities. 2025;31(23). doi: 10.1158/1557-3265.EARLYONSETCA25-PR001. | Conference Abstract, etc |
| 694 | Modi R, Wang HS, Sharma GN, Ocampo LH, Baek MD, Lee A, et al. Development of an online calculator to predict sub-optimal bowel preparation among patients scheduled for colonoscopy. Gastrointest Endosc. 2012;75(4):170-170. | Conference Abstract, etc |
| 695 | Moein HR, Faidhalla S, Khalid M, Naylor P, Mohamad B. Utilizing colorectal cancer predicting model in detecting colorectal adenomas in African American patients undergoing screening colonoscopy. American Journal of Gastroenterology. 2020;115(SUPPL):S1645. doi: 10.14309/01.ajg.0000714568.83387.82. | Conference Abstract, etc |
| 696 | Moen S, Vuik FER, Voortman T, Kuipers E, Spaander MCW. Predictors of gastrointestinal transit times in colon capsule endoscopy. Endoscopy. 2021;53(SUPPL 1):S250-S251. doi: 10.1055/s-0041-1724955. | Conference Abstract, etc |
| 697 | Mooiweer E, Severs M, Schipper ME, Fidder H, Siersema PD, Laheij R, et al. Fecal calprotectin can predict relapse in inflammatory bowel disease patients in endoscopic remission: a plea for extreme deep remission? Gastroenterology. 2014;146(5):S-233. doi: 10.1016/S0016-5085(14)60819-2. | Conference Abstract, etc |
| 698 | Moraveji S, Casner N, Dwivedi A, Bashashati M, Zuckerman M, Carrion AF, et al. Does addition of high dose simethicone to standard bowel preparation for screening colonoscopy have an effect on adenoma detection rate: a single-blind clinical trial. Gastroenterology. 2018;154(6):S‐141. doi: 10.1016/S0016-5085(18)30892-8. | Conference Abstract, etc |
| 699 | Morrison S, Chiu C, Cao R, Corte C, Lim P, Catriona M, et al. Video vision transformer for quantification of ulcerative colitis endoscopic and histologic disease activity. Journal of Gastroenterology and Hepatology (Australia). 2024;39:221. doi: 10.1111/jgh.16702. | Conference Abstract, etc |
| 700 | Mosby D, McGraw P, Duffalo C, Drees M, Depalma F, Herdman C, et al. Factors affecting effectiveness of fecal microbiota transplant. Open Forum Infectious Diseases. 2017;4:S386. doi: 10.1093/ofid/ofx163.959. | Conference Abstract, etc |
| 701 | Moy BT, Ahmad I, Devers TJ. Endoscopic features of mucous-capped polyps during colonoscopy: a retrospective study predicting sessile serrated adenomas. Gastroenterology. 2016;150(4):S764-S765. | Conference Abstract, etc |
| 702 | Murakami T, Day LW, Bhuket TP, Sewell JL, Kamal A. A pilot multi-center, single-blind, randomized controlled trial of split vs. whole bowel prep for inpatient colonoscopy. Gastrointest Endosc. 2013;77(5):AB517. | Conference Abstract, etc |
| 703 | Murthy S, Le Gal G, Benchimol EI, Hae R, Burke S, Rostom A, et al. A prediction model of risk of harbouring advanced colorectal neoplasms in low to moderate risk persons over age 50. J Can Assoc Gastroenterol. 2018;1:290-291. doi: 10.1093/jcag/gwy009.197. | Conference Abstract, etc |
| 704 | Mussetto A, Frazzoni L, Paggi S, Dari S, Laterza L, Radaelli F, et al. The low-volume preparation is not inferior to the high-volume preparation for bowel cleansing in patients with history of colorectal resection. Digestive and Liver Disease. 2015;47:e93. | Conference Abstract, etc |
| 705 | Mussetto A, Frazzoni L, Paggi S, Dari S, Laterza L, Radaelli F, et al. The low-volume mixed preparation (2-L PEG + bisacodyl) is not inferior to the high-volume standard preparation (4-L PEG) for bowel cleansing in patients with history of colorectal resection: a randomized controlled trial (the PEGOP trial). Gastrointest Endosc. 2015;81(5):AB377‐AB378. doi: 10.1016/j.gie.2015.03.1531. | Conference Abstract, etc |
| 706 | Nagar AB, Vance SR. Use of a validated bowel preparation questionnaire to evaluate tolerability of two large volume purges in patients at risk for poor bowel preparation. Gastrointest Endosc. 2018;87(6):AB491. | Conference Abstract, etc |
| 707 | Nassri AB, Smotherman C, Schey R, Ghali PM. Colonoscopy after holidays is an independent predictor for inadequate bowel preparation. Gastroenterology. 2019;156(6):S101-S101. | Conference Abstract, etc |
| 708 | Natsumi M, Higashimori A, Maruyama H, Ominami M, Fukunaga S, Otani K, et al. Adding linaclotide to 1-L PEG-ASC regimen: efficacy across age groups in a subgroup analysis of the APPLE trial. Gastrointest Endosc. 2025;101(5):S149. doi: 10.1016/j.gie.2025.03.255. | Conference Abstract, etc |
| 709 | Natsumi M, Higashimori A, Nakatani M, Jinnai K, Kin D, Sugita N, et al. Efficacy and tolerability of 1-L polyethylene glycol plus ascorbic acid with senna versus 2-L polyethylene glycol plus ascorbic acid for colonoscopic bowel preparation: a prospective, randomized, investigator-blinded trial. Gastrointest Endosc. 2023;97(6):AB161. doi: 10.1016/j.gie.2023.04.273. | Conference Abstract, etc |
| 710 | Niedermaier T, Alwers E, Chen X, Heisser T, Hoffmeister M, Brenner H. A single measurement of fecal hemoglobin concentration outperforms polygenic risk score in colorectal cancer risk assessment. Cancer Communications. 2023;43(8):947-950. doi: 10.1002/cac2.12448. | Conference Abstract, etc |
| 711 | Nsibi S, Ennaifer R, Ayari M, Bouchabou B, Farhat FB, Nejma HB. Is the split dose better than conventional bowel preparation in Tunisian patients? Digestive Endoscopy. 2020;32:139. doi: 10.1111/den.13597. | Conference Abstract, etc |
| 712 | Nsibi S, Ennaifer R, Bouchabou B, Romdhane HB, Nejma HB. Is the split dose better than conventional bowel preparation in Tunisian patients? Turkish Journal of Gastroenterology. 2019;30:S595. doi: 10.5152/tjg.2019.050919. | Conference Abstract, etc |
| 713 | Ono S, Ono Y, Matsumoto M, Ishibe E, Inoue M, Sakamoto N. Development of a smartphone application by artificial intelligence to predict bowel preparation quality for colonoscopy. J Gastroenterol Hepatol. 2024;39:192-192. | Conference Abstract, etc |
| 714 | Orkoonsawat P, Aniwan S, Rerknimitr R, Viriyautsahakul V, Angsuwatcharakon P, Pittayanon R, et al. The quality indicator to predict adenoma miss rate (AMR) during back-to-back colonoscopy that is better than adenoma detection rate (ADR). Gastrointest Endosc. 2015;81(5):AB170. doi: 10.1016/j.gie.2015.03.128. | Conference Abstract, etc |
| 715 | Orton CB, Powell SM, Stine JG. Preventing poor bowel preps before they become a problem. Gastroenterology. 2016;150(4):S149-S150. | Conference Abstract, etc |
| 716 | Ozden Y, Saglam O, Omurca F, Yuzugulen O, Tekis D, Buyukberber NM. Comparison of three bowel cleansing agents for colonoscopy and factors affecting their efficacy. Turkish Journal of Gastroenterology. 2025;36:S70. doi: 10.5152/tjg.2025.101124. | Conference Abstract, etc |
| 717 | Palma R, Panetta C, Eberspacher C, Pontone P, Angelini R, Pontone S. A pilot study analyzing a 1L PEG bowel preparation before colonoscopy for selected inpatients. Digestive and Liver Disease. 2017;49:e220‐e221. | Conference Abstract, etc |
| 718 | Pan Y, Chen L, Kang X, Wang L, Zhao J, Zhang R, et al. ID: 3525189 Superiority of a procedure-related model for predicting inadequate bowel preparation in patients undergoing colonoscopy: a multicenter prospective study. Gastrointest Endosc. 2021;93(6):AB41. doi: 10.1016/j.gie.2021.03.144. | Conference Abstract, etc |
| 719 | Paranjape SY, Grigorian A, Selby LA, Flomenhoft DR, Mardini HE. Colonoscopy indication and gender may predict colonic prep quality. Gastrointest Endosc. 2009;69(5):AB213. doi: 10.1016/j.gie.2009.03.490. | Conference Abstract, etc |
| 720 | Park S, Kang Y, Koh H, Kim S. Combining faecal calprotectin and sigmoidoscopy can predict mucosal healing in paediatric ulcerative colitis. Journal of Crohn's and Colitis. 2019;13:S406-S407. doi: 10.1093/ecco-jcc/jjy222.708. | Conference Abstract, etc |
| 721 | Pathipati MP, Silvernale CJ, Barshop K, Staller K. Disordered defecation associated with poor bowel preparation in patients with chronic constipation. Gastroenterology. 2020;158(6):S-397. doi: 10.1016/S0016-5085(20)31683-8. | Conference Abstract, etc |
| 722 | Peng Y, Liu X. Factor screening to improve the quality of bowel preparation: a prospective single-centre study from China. J Gastroenterol Hepatol. 2024;39:194. doi: 10.1111/jgh.16780. | Conference Abstract, etc |
| 723 | Peng Y, Liu X. Factors that affect a patient's decision to undergo anesthetic colonoscopy: prospective single-center study from China. J Gastroenterol Hepatol. 2024;39:194-195. doi: 10.1111/jgh.16780. | Conference Abstract, etc |
| 724 | Pereyra L, Gomez EJ, Criniti JM, Bentolila FE, Nunez AM, Roel M, et al. Effectiveness of screening colonoscopy in reducing the incidence of colorectal cancer according to anatomic location, endoscopists specialty and colonoscopy quality. Gastroenterology. 2018;154(6):S‐772. doi: 10.1016/S0016-5085(18)32669-6. | Conference Abstract, etc |
| 725 | Pietrak S, Kang M, Patel D, Colangelo T, Ahmad AS. BMI is not a significant risk factor for colorectal adenomas in a predominantly African American, female inner-city population. Gastrointest Endosc. 2017;85(5):AB387. | Conference Abstract, etc |
| 726 | Pietrzak AM, Kaminski M, Kraszewska E, Rupinski M, Regula J. 30 days hospitalization rate after screening colonoscopy-case-control study nested in prospective randomized controlled study. United European Gastroenterology Journal. 2013;1(1):A322. doi: 10.1177/2050640613502900. | Conference Abstract, etc |
| 727 | Pontone S, Palma R, Panetta C, Eberspacher C, Pontone P. 1L PEG bowel preparation before colonoscopy for selected high-risk inpatients in a pilot study. Gastroenterology. 2017;152(5):S1298‐S1299. | Conference Abstract, etc |
| 728 | Portincasa P, Altomare DF, Berardino M, Sasso C, Moschetta R, Maurogiovanni G, et al. Hydrogen breath test (HBT) with inulin supplementation discriminates between good and poor bowel preparation for colonoscopy. Colorectal Disease. 2009;11:19. doi: 10.1111/j.1463-1318.2009.01978.x. | Conference Abstract, etc |
| 729 | Prasad N, Mullins PD. A novel scoring system to predict poor bowel preparation for colonoscopy. Gastrointest Endosc. 2009;69(5):AB233-AB233. doi: 10.1016/j.gie.2009.03.571. | Conference Abstract, etc |
| 730 | Quach DT. Evaluation of Asia-Pacific colorectal screening score in predicting risk of advanced colorectal neoplasia in Vietnamese with lower bowel symptoms. J Gastroenterol Hepatol. 2012;27:178. doi: 10.1111/jgh.12006. | Conference Abstract, etc |
| 731 | Radaelli F, Paggi S, Imperiali G, Amato A, Rondonotti E, Spinzi G, et al. The impact of a “resect and discard” strategy on surveillance times after colorectal polypectomy: preliminary data of a prospective study. American Journal of Gastroenterology. 2011;106:S542. doi: 10.1038/ajg.2011.336_11. | Conference Abstract, etc |
| 732 | Radford-Smith D, Yates A, Hageman I, Joustra V, Li Yim A, Davids M, et al. An inflammatory serum metabolomic signature predicts response to vedolizumab treatment in people with Crohn's disease. Journal of Crohn's and Colitis. 2022;16:i257-i258. doi: 10.1093/ecco-jcc/jjab232.325. | Conference Abstract, etc |
| 733 | Rattan A. Predictive factors for interval colorectal cancer. J Gastroenterol Hepatol. 2012;27:42. doi: 10.1111/j.1440-1746.2011.07251-3.x. | Conference Abstract, etc |
| 734 | Rejeski J, Pleasant T, Jahann DA, Green P, Fina MF, Rudnick S, et al. A pilot study of using smartphone toilet bowl photography to assess the quality of pre-colonoscopy bowel cleansing. Gastrointest Endosc. 2022;95(6):AB66-AB67. doi: 10.1016/j.gie.2022.04.187. | Conference Abstract, etc |
| 735 | Reynauld, Rasouli KN, Gofton C, Gilbert D. ID: 3524989 Does formal polyp histology alter surveillance intervals predicted at the time of colonoscopy / polypectomy with high definition endoscopes? A prospective cohort study. Gastrointest Endosc. 2021;93(6):AB25-AB26. doi: 10.1016/j.gie.2021.03.116. | Conference Abstract, etc |
| 736 | Rivero SnL, Arnau Colell C, Balaguer F, Herrero Rivas JM, Remedios Espino D, Alvarez Sanchez MV, et al. High-definition white-light colonoscopy versus chromoendoscopy for surveillance of Lynch syndrome. A multicenter, randomized and controlled study (ENDOLYNCH study). United European Gastroenterology Journal. 2018;6(8):A117. doi: 10.1177/2050640618792817. | Conference Abstract, etc |
| 737 | Riverso M, Perbtani YB, Shuster JJ, Chakraborty J, Brar TS, Zhang H, et al. Carbon dioxide insufflation during screening colonoscopy is associated with higher serrated adenoma detection rate: a single-center retrospective analysis. American Journal of Gastroenterology. 2016;111:S115. doi: 10.1038/ajg.2016.353. | Conference Abstract, etc |
| 738 | Robles-Medranda C, Del Valle RS, Lukashok HP, Abarca F, Robles-Jara C. Pentax i-Scan™ with electronic magnification for the real-time histological prediction of colonic polyps: a prospective study using a new digital chromoendoscopy setting. Gastrointest Endosc. 2013;77(5):AB463. | Conference Abstract, etc |
| 739 | Roghani RS, Amirbeigy MK, Baghbanian M, Salmanroghani H. Erythromycin as an adjunctive therapy for improved bowel preparation in diabetic patients: a pilot study. American Journal of Gastroenterology. 2023;118(10):S519‐S520. | Conference Abstract, etc |
| 740 | Sanchez A, Navarro M, Moreno L, Ocaña T, Pineda M, Rodriguez-Moranta F, et al. Identification of clinical, genetic and endoscopic predictors of incident colorectal cancer in Lynch syndrome. Gastroenterology. 2018;154(6):S-789-S-790. doi: 10.1016/S0016-5085(18)32718-5. | Conference Abstract, etc |
| 741 | Satorres C, García-Campos M, García-Morales N, Alonso N, Ponce M, Argüello L, et al. Influence of bowel preparation (BP) quality on the detection of serrated polyps (SP): a prospective study in a regional FOBT-based colorectal cancer screening program. Endoscopy. 2019;51(4):S134. doi: 10.1055/s-0039-1681564. | Conference Abstract, etc |
| 742 | Schick BA, McLean CA, Driman DK. Negative colorectal polyp biopsies: the utility of cutting deeper levels. Laboratory Investigation. 2014;94:202A. doi: 10.1038/labinvest.2014.22. | Conference Abstract, etc |
| 743 | Schindler AE, Chan WW, Ryou M, Obstein KL. Patient understanding of bowel preparation instructions directly correlates with quality of colonoscopy bowel preparation. Gastroenterology. 2012;142(5):S233. | Conference Abstract, etc |
| 744 | Schoenfeld PS, Poppers DM, Cash BD, Allen C, Sharma P. One liter polyethylene glycol-based NER1006 is efficacious as a bowel preparation for colonoscopy in patients with or without diabetes mellitus: a pooled analysis of 2 randomized, phase 3 studies. American Journal of Gastroenterology. 2020;115(SUPPL):S115. doi: 10.14309/01.ajg.0000702996.21249.3e. | Conference Abstract, etc |
| 745 | Sekmen M, Polatkan SAV. Effect of colonoscopy on atrial parameters. European Research Journal. 2025;11:S88-S90. | Conference Abstract, etc |
| 746 | Shah JM, Forlemu A, Kumar V, Gayam V, Then E. Predictors of inadequate bowel preparation for inpatient colonoscopies in a single center community hospital. Gastroenterology. 2024;166(5):S-653. doi: 10.1016/S0016-5085(24)01954-1. | Conference Abstract, etc |
| 747 | Shah S, Hove JT, Shaffer S, Bernstein CN, Castaneda D, Palmela C, et al. Consecutive negative findings on colonoscopy during surveillance predict a low risk of advanced neoplasia in patients with longstanding colitis: results of a 15-year multicenter, multinational cohort study. Gastroenterology. 2018;154(6):S-129. doi: 10.1016/S0016-5085(18)30857-6. | Conference Abstract, etc |
| 748 | Sharma P, Schoenfeld PS, Allen C, Cash BD. NER1006 1 liter polyethylene glycol (PEG)-based bowel preparation safety profile in patients with mild or moderate renal impairment: a pooled analysis of two phase 3 trials. American Journal of Gastroenterology. 2020;115(SUPPL):S89. doi: 10.14309/01.ajg.0000702780.25653.0a. | Conference Abstract, etc |
| 749 | Sharma S, Duong E, Davies H, Tan T. Ketosis in patients undergoing elective colonoscopy: more common than you think. J Gastroenterol Hepatol. 2022;37:240-241. doi: 10.1111/jgh.15959. | Conference Abstract, etc |
| 750 | Shavrov A, Koshurnikova A, Starostina I, Ibragimov S, Lightdale J. A prospective, randomized, dual-center study investigating preparation and patient-related risk factors for inadequate bowel cleansing in 450 children undergoing colonoscopy. Journal of Pediatric Gastroenterology and Nutrition. 2022;75:S373‐S374. | Conference Abstract, etc |
| 751 | Shenoy V, Kumar A, Buckley MC, Durbin L, Mackey J, Mone A, et al. Endoscopic disease activity and biologic therapy predict quality of bowel preparation for colonoscopies in inflammatory bowel disease. American Journal of Gastroenterology. 2021;116(SUPPL):S383. doi: 10.14309/01.ajg.0000776836.80221.88. | Conference Abstract, etc |
| 752 | Shin JE, Choi SC, Park KS, Lee TH, Lee BE, Park SY, et al. Can we predict the degree of colon preparation with constipated symptoms? Results from a nationwide multicenter study. Journal of Gastroenterology and Hepatology (Australia). 2015;30:193. doi: 10.1111/jgh.13187/abstract. | Conference Abstract, etc |
| 753 | Shin JE, Lee HY, Yun SU, Lee BH, Shin HD, Yun SY, et al. Factors affecting adenoma detection during colonoscopy: a prospective study. J Gastroenterol Hepatol. 2009;24:A115. | Conference Abstract, etc |
| 754 | Shin JE, Shin HD, Bang KB, Nam GW. Can we predict the presence of colonic bubbles with bowel symptoms during colonoscopy? Neurogastroenterology and Motility. 2017;29:134. doi: 10.1111/nmo.13180. | Conference Abstract, etc |
| 755 | Shin SY, Lee YJ, Jung Y, Lee J, Choi CH, Park JJ. Efficacy and safety of D-sorbitol and picosulfate plus ascorbic acid, a new bowel cleansing solution for colonoscopy: prospective multicenter randomized study. Gut and Liver. 2019;13:63. | Conference Abstract, etc |
| 756 | Shingina A, Svarta S, Ou G, Takach O, Kwok RW, Tong J, et al. Identification of factors associated with sedation tolerance during colonoscopy: a retrospective review of 5000 patients. Gastrointest Endosc. 2014;79(5):AB224. doi: 10.1016/j.gie.2014.02.332. | Conference Abstract, etc |
| 757 | Siddique DA, Gupta A, Howard G, Shin AS, Bohm M, Siwiec RM, et al. Differences in clinical presentation between patients with aerodigestive tract small intestinal bacterial overgrowth (SIBO) and coliform SIBO. Gastroenterology. 2020;158(6):S‐639. doi: 10.1016/S0016-5085(20)32294-0. | Conference Abstract, etc |
| 758 | Siersema PD, Rastogi A, A ML, Akerman PA, Azzouzi K, Rothstein RI, et al. Improved adenoma detection rate during colonoscopy with use of a retrograde-viewing auxiliary imaging device. Gastrointest Endosc. 2012;75(4):AB216‐AB217. doi: 10.1016/j.gie.2012.04.391. | Conference Abstract, etc |
| 759 | Sim JS, Koo JS. Predictors of inadequate bowel preparation and salvage options on colonoscopy. Clin Endosc. 2016;49(4):346-349. doi: 10.5946/ce.2016.094. | Conference Abstract, etc |
| 760 | Singhal S, Singh M, Bahga H, Momeni M, Krishnaiah M, Anand S. Can we predict quality of bowel preparation in colonoscopy by stool frequency and form using Bristol Stool Scale? Gastrointest Endosc. 2011;73(4):AB433. doi: 10.1016/j.gie.2011.03.1005. | Conference Abstract, etc |
| 761 | Smale M, Lee L, Humphris J. EPIC: a novel scoring system to identify patients likely to benefit from extended bowel preparation at index colonoscopy. Journal of Gastroenterology and Hepatology (Australia). 2024;39:338-339. doi: 10.1111/jgh.16708. | Conference Abstract, etc |
| 762 | Sondhi A, Kurlander JE, Saini SD. A systematic review of hydrogen breath testing as a predictor of bowel preparation quality. American Journal of Gastroenterology. 2015;110:S950. | Conference Abstract, etc |
| 763 | Souto M, Ferreira AI, Lima Capela T, MacEdo Silva V, Arieira C, Rosa B, et al. Colon capsule endoscopy: what factors predict an incomplete examination? United European Gastroenterology Journal. 2024;12:1169. doi: 10.1002/ueg2.12615. | Conference Abstract, etc |
| 764 | Srinivasamaharaj S, Chaudhary D, Liu N, Ahmed S, Kadle N, Mehta MJ, et al. Association of vitamin D deficiency and pre-cancerous colonic polyps in initial colonoscopies. Gastroenterology. 2017;152(5):S550. | Conference Abstract, etc |
| 765 | Stasinos I, Britto J, Gulati S, Chang-Faye G, Joshi D, Dubois P, et al. The role of preassessment in bowel preparation. Gut. 2016;65:A229. doi: 10.1136/gutjnl-2016-312388.429. | Conference Abstract, etc |
| 766 | Su MY, Hsu CM. Factors predictive of painful colonoscopy-a multivariate analysis. Journal of Gastroenterology and Hepatology (Australia). 2016;31:312. doi: 10.1111/jgh.13540. | Conference Abstract, etc |
| 767 | Symonds E, Cock C, Bampton P, Fraser R, Young G. Does the hemoglobin concentration in a negative fecal immunochemical test result predict future surveillance colonoscopy outcomes? J Gastroenterol Hepatol. 2019;34:215. doi: 10.1111/jgh.14806. | Conference Abstract, etc |
| 768 | Tan J, Kwan C, Ling KL, Ooi CJ, Mesenas S, Lui HF, et al. Patient age, male gender and endoscopists' experience were independent predictors of adenoma detection in Asian patients. American Journal of Gastroenterology. 2010;105:S136-S137. doi: 10.1038/ajg.2010.320-6. | Conference Abstract, etc |
| 769 | Tan JC, La Nauze R, Bunn C, Secomb R, Gibson P, Brown GJ. Oral rehydration solution orange vs blackcurrant in addition to standard bowel preparation for colonoscopy: a pilot study. Journal of Gastroenterology and Hepatology (Australia). 2014;29:43‐44. doi: 10.1111/jgh.12736-3. | Conference Abstract, etc |
| 770 | Tang CP, Lin TL, Hsieh YH, Hsieh CH, Leung FW. Computer-aided diagnosis (CADx) of right colon adenomas during withdrawal phase of colonoscopy in a randomized controlled trial of water exchange versus air insufflation based on analysis of video recordings. Gastrointest Endosc. 2022;95(6):AB227. doi: 10.1016/j.gie.2022.04.581. | Conference Abstract, etc |
| 771 | Taveira F, Areia M, Elvas L, Alves S, Brito D, Saraiva S, et al. A 3-day versus 1-day low residue diet to improve colonoscopy preparation result and patient tolerability, a randomized, single-blinded, controlled trial. United European Gastroenterology Journal. 2019;7(8):547. doi: 10.1177/205064061985467. | Conference Abstract, etc |
| 772 | Taylor A, De Barros M, Holtmann G, O'Connor S, Burger D. When to stop surveillance colonoscopy in the older population. Journal of Gastroenterology and Hepatology (Australia). 2020;35(SUPPL 1):232-233. doi: 10.1111/jgh.15277. | Conference Abstract, etc |
| 773 | Taylor K, Gilbert D. Incidental terminal ileal erosions do not predict development of Crohn's disease in asymptomatic individuals. J Gastroenterol Hepatol. 2018;33:181. doi: 10.1111/jgh.14399. | Conference Abstract, etc |
| 774 | Tee HP, Corte C, Al-Ghamdi H, Prakoso E, Chettier R, Darke J, et al. A prospective randomized controlled trial evaluating cap-assisted colonoscopy versus standard colonoscopy - a western experience. J Gastroenterol Hepatol. 2009;24:A250‐A251. doi: 10.1111/j.1440-1746.2009.06053.x. | Conference Abstract, etc |
| 775 | Tejaswi S, Wilson M. Prevalence of colorectal adenomatous polyps among Asian Indians versus Caucasians. American Journal of Gastroenterology. 2014;109:S606. doi: 10.1038/ajg.2014.285. | Conference Abstract, etc |
| 776 | Thabane M, Chauhan N, Chauhan U, Tse F, Dahab ST, Wolfe MA, et al. Predictors of bowel preparation quality in patients undergoing colonoscopy - a preliminary analysis. Gastroenterology. 2008;134(4):A319-A319. | Conference Abstract, etc |
| 777 | Thin LWY, Lawrance IC. Survival outcomes after a colonoscopy ± polypectomy in the elderly. J Gastroenterol Hepatol. 2010;25:A29. doi: 10.1111/j.1440-1746.2010.06451.x. | Conference Abstract, etc |
| 778 | Toro B, Dawkins G, Friedenberg FK, Ehrlich AC. Risk factors for failure to return after a poor preparation colonoscopy: experience in a safety-net hospital. American Journal of Gastroenterology. 2016;111:S120. doi: 10.1038/ajg.2016.353. | Conference Abstract, etc |
| 779 | Triantafyllou K, Gkolfakis P, Gralnek IM, Oakland K, Manes G, Radaelli F, et al. Diagnosis and management of acute lower gastrointestinal bleeding: European Society of Gastrointestinal Endoscopy (ESGE) guideline. Endoscopy. 2021;53(8):850-868. doi: 10.1055/a-1496-8969. | Conference Abstract, etc |
| 780 | Triantafyllou K, Gkolfakis P, Skamnelos A, Diamantopoulou G, Dagas A, Tziatzios G, et al. Validation of a score predicting inadequate bowel preparation before colonoscopy in hospitalized patients. Endoscopy. 2019;51(4):S197-S198. doi: 10.1055/s-0039-1681755. | Conference Abstract, etc |
| 781 | Triantafyllou K, Gkolfakis P, Skamnelos A, Diamantopoulou G, Dagas A, Tziatzios G, et al. The index evaluating individuals' independency in activities of daily living does not satisfactorily predict inadequate bowel preparation in inpatients undergoing colonoscopy. Endoscopy. 2019;51(4):S151-S152. doi: 10.1055/s-0039-1681617. | Conference Abstract, etc |
| 782 | Uddin FS, Iqbal R, Harford W, Dunbar KB, Cryer BL, Spechler SJ, et al. Prone vs standard, left-lateral decubitus position colonoscopy for obese patients: a randomized, prospective study. Gastrointest Endosc. 2012;75(4):AB155. doi: 10.1016/j.gie.2012.04.101. | Conference Abstract, etc |
| 783 | Valiante F, Bellumat A, De Bona M, Hassan C, De Boni M. A randomised controlled trial of a new 2 litre polyethylene glycol solution (PEG) versus a standard 4-liter PEG for bowel cleansing prior to colonoscopy. Digestive and Liver Disease. 2011;43:S249‐S250. doi: 10.1016/S1590-8658(11)60534-3. | Conference Abstract, etc |
| 784 | Vallejo Senra N, Monteserin-Ron L, Lopez-Diaz J, Olmos JM, Dominguez-Munoz JE. Free diet vs low-residue diet in the quality of outpatient colonoscopy: a prospective, randomized, single blind clinical trial. United European Gastroenterology Journal. 2019;7(8):542. doi: 10.1177/205064061985467. | Conference Abstract, etc |
| 785 | Van Wassenaer EA, Benninga MA, Van Limbergen JL, D'Haens GR, Griffiths AM, Koot BGP. Intestinal ultrasound in pediatric inflammatory bowel disease: promising, but work in progress. Inflammatory Bowel Diseases. 2022;28(5):783-787. doi: 10.1093/ibd/izab125. | Conference Abstract, etc |
| 786 | Varadarajulu SS. Exam 2: A predictive model identifies patients most likely to have inadequate bowel preparation for colonoscopy. Clinical Gastroenterology and Hepatology. 2012;10(5):e49. doi: 10.1016/j.cgh.2012.03.003. | Conference Abstract, etc |
| 787 | Vassallo R, Maida M, Zullo A, Venezia L, Montalbano LM, Di Mitri R, et al. Effectiveness of 1-L polyethylene glycol plus ascorbate versus 4-L polyethylene glycol for colonoscopy cleansing in split-dose: a multicentre, randomized, clinical trial (OVER2019). Gastrointest Endosc. 2023;97(6):AB408‐AB409. doi: 10.1016/j.gie.2023.04.677. | Conference Abstract, etc |
| 788 | Veeraprasit T, Piyanirun W, Sanpajit T, Opuchar K, Puttapitakpong C. Effectiveness of smart phone application to predict the quality of bowel preparation in colonoscopy. Gastrointest Endosc. 2019;89(6):AB619-AB620. doi: 10.1016/j.gie.2019.03.1077. | Conference Abstract, etc |
| 789 | Veldhuijzen G, Klemt-Kropp M, Terhaar sive Droste JS, van Balkom B, van Esch A, Drenth J. E-patient counseling trial (E-PACO): computer based patient education is non-inferior to nurse counseling prior to colonoscopy, a multicenter randomized controlled trial. Gastrointest Endosc. 2019;89(6):AB426‐AB427. doi: 10.1016/j.gie.2019.03.665. | Conference Abstract, etc |
| 790 | Vinall A, Siddiqui U, Alvarez J, Umapathy C. Developing a novel tool to attempt to predict inadequate bowel preparation. 2024. p. AB188-AB189. | Conference Abstract, etc |
| 791 | Wadehra A, Antaki F, Kanaan Z, Rifkin S, Lilley K. Debunking endoscopy myths: GLP1-RA use and GI symptoms do NOT predict bowel preparation success. American Journal of Gastroenterology. 2024;119(10):S642-S643. doi: 10.14309/01.ajg.0001033044.23298.e6. | Conference Abstract, etc |
| 792 | Wang T, He Z, Fan X, Li Z, Bai Y. Current status and progress of research in risk prediction models for colonoscopic bowel preparation failure. Chinese Journal of Digestive Endoscopy. 2024;41(9):741-745. doi: 10.3760/cma.j.cn321463-20231118-00178. | Conference Abstract, etc |
| 793 | Wangrattanapranee P, Jensen DM, Ellen Jensen M. Which is the more likely diagnosis for patients hospitalized with severe hematochezia: internal hemorrhoids or diverticular hemorrhage? 2025. p. S351-S352. | Conference Abstract, etc |
| 794 | Weiner CS, Cooper GS. Yield of follow up colonoscopy is not associated with initial bowel preparation. Gastrointest Endosc. 2009;69(5):AB273-AB274. doi: 10.1016/j.gie.2009.03.730. | Conference Abstract, etc |
| 795 | Xiao A, Anandabaskaran S, Ow M. Clinical variables that predict for and against colorectal cancer at colonoscopy in the very elderly. Journal of Gastroenterology and Hepatology (Australia). 2017;32:31. doi: 10.1111/jgh.13889. | Conference Abstract, etc |
| 796 | Xu B, Xu C, Zhang H, Chen X, Wei B, Cheng Y. Development and validation of a risk prediction model for colorectal adenoma based on patient and colonoscopy characteristics. United European Gastroenterology Journal. 2025;13:554. doi: 10.1002/ueg2.70032. | Conference Abstract, etc |
| 797 | Yuan X, Gao H, Zheng S, Liu C, Wang W, Xie J, et al. Nomogram of development and validation for estimation of inadequate bowel preparation risk before colonoscopy in adults. Gut. 2021;70(SUPPL 2):A124-A125. doi: 10.1136/gutjnl-2021-IDDF.141. | Conference Abstract, etc |
| 798 | Yung SH, Coughlan J, Everett SM, Ford Leeds AC. Low yield of colonoscopy for melaena following a normal upper gastrointestinal endoscopy: a 5-year analysis. Gut. 2011;60:A127. doi: 10.1136/gut.2011.239301.270. | Conference Abstract, etc |
| 799 | Zad M, Al-Ansari M, Hussain A, Smith N, Tung A, Urbanavicious D. Predicting inadequate bowel preparation for colonoscopy. J Gastroenterol Hepatol. 2015;30:67-67. | Conference Abstract, etc |
| 800 | Zhang J, Chen M, Xiao Y, Zhang H, Long H, Li S, et al. How to optimize bowel preparation for high-risk population: a multicenter, endoscopist-blinded randomized controlled trial of lactulose combined with polyethylene glycol on bowel preparation quality in different risk-stratified populations. Gastrointest Endosc. 2023;97(6):AB127. doi: 10.1016/j.gie.2023.04.218. | Conference Abstract, etc |
| 801 | Zhang L, Mohsen W, Wark G, Williams AJ, Sechi A, Koo JH, et al. Is quality of bowel preparation affected by ambient temperature? Journal of Gastroenterology and Hepatology (Australia). 2020;35(SUPPL 1):222. doi: 10.1111/jgh.15277. | Conference Abstract, etc |
| 802 | Zhang Y, Wang L, Wu W, Zhang S, Zhang M, She W, et al. Predictors of inadequate bowel preparation in older patients undergoing colonoscopy: a systematic review and meta-analysis. Int J Nurs Stud. 2024;149:104631. doi: 10.1016/j.ijnurstu.2023.104631. | Conference Abstract, etc |
| 803 | Zhao Y, Xie F, Bai X, Yang A, Wu D. Effect of educational virtual reality videos on improving bowel preparation quality and satisfaction of outpatients undergoing colonoscopy. Turkish Journal of Gastroenterology. 2019;30:S253‐S254. doi: 10.5152/tjg.2019.050919. | Conference Abstract, etc |
| 804 | Zhou H, Zhang X, Yang J. Correlation study of bowel cleanliness in various areas of the Boston bowel preparation scale. Gastrointest Endosc. 2020;91(6):AB558-AB559. doi: 10.1016/j.gie.2020.03.3303. | Conference Abstract, etc |
| 805 | Zhou JQ, Tang XW, Wei ZJ, Gong W, Bo J. Factors predicting inadequate bowel preparation for colonoscopy: a prospective observational study. Journal of Digestive Diseases. 2017;18:155-156. doi: 10.1111/1751-2980.12518. | Conference Abstract, etc |
| 806 | Abaskharoun R, Depew W, Vanner S. Changes in renal function following administration of oral sodium phosphate or polyethylene glycol for colon cleansing before colonoscopy. Can J Gastroenterol. 2007;21(4):227-231. doi: 10.1155/2007/630639. | Irrelevant Topic |
| 807 | Abdul-Baki H, Hashash JG, Elhajj, II, Azar C, El Zahabi L, Mourad FH, et al. A randomized, controlled, double-blind trial of the adjunct use of tegaserod in whole-dose or split-dose polyethylene glycol electrolyte solution for colonoscopy preparation. Gastrointest Endosc. 2008;68(2):294-300; quiz 334, 336. doi: 10.1016/j.gie.2008.01.044. | Irrelevant Topic |
| 808 | Abu-Freha N, Guterman R, Elhayany R, Cohen DL, Munteanu D, Dizengof V, et al. Sex differences in colonoscopy indications and findings: results from a large multicenter database. Journal of Women's Health. 2024;33(11):1442-1448. doi: 10.1089/jwh.2024.0301. | Irrelevant Topic |
| 809 | Abu-Freha N, Yitzhak A, Shirin H, Nevo-Shor A, Abu-Jaffar J, Abu-Rafe S, et al. Glucagon-like peptide-1 receptor agonists significantly affect the quality of bowel preparation for colonoscopy. Endoscopy. 2025;57(2):126-133. doi: 10.1055/a-2419-3875. | Irrelevant Topic |
| 810 | Adamek HE, Bergmann L, Müssig K. [Bowel preparation in patients with diabetes mellitus: development of a procedure model]. Z Gastroenterol. 2022;60(5):784-789. doi: 10.1055/a-1791-1627. | Irrelevant Topic |
| 811 | Adamek HE, Bergmann L, Müssig K. Bowel preparation in patients with diabetes mellitus: development of a procedure model. Diabetologie und Stoffwechsel. 2025;20(03):205-210. doi: 10.1055/a-2537-2150. | Irrelevant Topic |
| 812 | Adler A, Wegscheider K, Lieberman D, Aminalai A, Aschenbeck J, Drossel R, et al. Factors determining the quality of screening colonoscopy: a prospective study on adenoma detection rates, from 12 134 examinations (Berlin colonoscopy project 3, BECOP-3). Gut. 2013;62(2):236-241. doi: 10.1136/gutjnl-2011-300167. | Irrelevant Topic |
| 813 | Adler J, Robertson DJ. Interval colorectal cancer after colonoscopy: exploring explanations and solutions. American Journal of Gastroenterology. 2015;110(12):1657-1664. doi: 10.1038/ajg.2015.365. | Irrelevant Topic |
| 814 | Agazzi S, Lenti MV, Klersy C, Strada E, Pozzi L, Rovedatti L, et al. Incidence and risk factors for preneoplastic and neoplastic lesions of the colon and rectum in patients under 50 referred for colonoscopy. Eur J Intern Med. 2021;87:36-43. doi: 10.1016/j.ejim.2021.02.008. | Irrelevant Topic |
| 815 | Ahammed SM, Das K, Sarkar R, Dasgupta J, Bandopadhyay S, Dhali GK. Patient-posture and ileal-intubation during colonoscopy (PIC): a randomized controlled open-label trial. Endosc Int Open. 2014;2(2):E105-110. doi: 10.1055/s-0034-1365541. | Irrelevant Topic |
| 816 | Ahmad W, Yoshida N, Cardoso L, Rehman S, Kobayashi R, Iwai N, et al. Retrospective analysis of bowel bubbles during colonoscopy following bowel preparation with oral sulfate solution or 2 L polyethylene glycol-electrolyte solution with ascorbic acid. Dig Dis Sci. 2025;70(12):4207-4217. doi: 10.1007/s10620-025-09277-4. | Irrelevant Topic |
| 817 | Ajeesh S, Luis R. A comprehensive electronic health record based patient navigation module including technology driven colorectal cancer outreach and education. J Cancer Educ. 2018;33(3):627-633. doi: 10.1007/s13187-017-1184-9. | Irrelevant Topic |
| 818 | Akgul G, Yeniova AO, Ozsoy Z, Yenidogan E, Kefeli A, Dasiran MF, et al. Effect and tolerability of same-day repeat colonoscopy. Journal of Investigative Surgery. 2020;33(5):459-465. doi: 10.1080/08941939.2018.1513611. | Irrelevant Topic |
| 819 | Aksan F, Tanriverdi LH, Figueredo CJ, Barrera LC, Hasham A, Jariwala SP. The impact of smartphone applications on bowel preparation, compliance with appointments, cost-effectiveness, and patients' quality of life for the colonoscopy process: a scoping review. Saudi Journal of Gastroenterology. 2023;29(2):71-87. doi: 10.4103/sjg.sjg_207_22. | Irrelevant Topic |
| 820 | Al Alawi S, Al Dhahab H, Al Salmi I. Split dose bowel preparation before colonoscopy of PEG (Nulytely) in comparison to routine single dose bowel preparation. Saudi J Gastroenterol. 2021;27(4):234-239. doi: 10.4103/sjg.sjg_563_20. | Irrelevant Topic |
| 821 | Alali AA, Alrashidi R, Allahow F, Dangi A, Alfadhli A. Predictive factors of significant findings on capsule endoscopy in patients with suspected small bowel bleeding. Diagnostics. 2024;14(21). doi: 10.3390/diagnostics14212352. | Irrelevant Topic |
| 822 | Alberti LR, De Lima DC, Rodrigues KC, Taranto MP, Gonçalves SH, Petroianu A. The impact of colonoscopy for colorectal cancer screening. Surg Endosc. 2012;26(11):3157-3162. doi: 10.1007/s00464-012-2308-2. | Irrelevant Topic |
| 823 | Alharfi S, Furey N, Al-Shakhshir H, Ghannoum M, Cooper GS. Fecal microbiome associated with both colon adenomas and lifetime colorectal cancer risk. Dig Dis Sci. 2023;68(4):1492-1499. doi: 10.1007/s10620-022-07673-8. | Irrelevant Topic |
| 824 | Allen ML, Kluger M, Schneider F, Jordan K, Xie JH, Leslie K. Fluid responsiveness and hypotension in patients undergoing propofol-based sedation for colonoscopy following bowel preparation: a prospective cohort study. Canadian Journal of Anesthesia. 2025;72(4):529-539. doi: 10.1007/s12630-025-02939-x. | Irrelevant Topic |
| 825 | Almadi MA, Alharbi O, Azzam N, Altayeb M, Thaniah S, Aljebreen A. Bowel preparation quality between hospitalized patients and outpatient colonoscopies. Saudi J Gastroenterol. 2018;24(2):93-99. doi: 10.4103/sjg.SJG_485_17. | Irrelevant Topic |
| 826 | Almasoud A, Almalaq AA, Aldiebany B, AlMeghaiseeb E, Alamro R, Albishi A, et al. Correlation of pre-colonoscopy blood hemoglobin levels with significant colorectal pathology among fecal immunochemical test-positive patients. Cureus. 2025;17(11):e96710. doi: 10.7759/cureus.96710. | Irrelevant Topic |
| 827 | Altomare DF, Bonfrate L, Krawczyk M, Lammert F, Caputi-Jambrenghi O, Rizzi S, et al. The inulin hydrogen breath test predicts the quality of colonic preparation. Surg Endosc. 2014;28(5):1579-1587. doi: 10.1007/s00464-013-3354-0. | Irrelevant Topic |
| 828 | Alvarez-Gonzalez MA, Flores-Le Roux JA, Seoane A, Pedro-Botet J, Carot L, Fernandez-Clotet A, et al. Efficacy of a multifactorial strategy for bowel preparation in diabetic patients undergoing colonoscopy: a randomized trial. Endoscopy. 2016;48(11):1003‐1009. doi: 10.1055/s-0042-111320. | Irrelevant Topic |
| 829 | Alvarez-Gonzalez MA, Sánchez MAP, Cabredo BB, García-Rodríguez A, Larramona SF, Nogales O, et al. Educational nurse-led telephone intervention shortly before colonoscopy as a salvage strategy after previous bowel preparation failure: a multicenter randomized trial. Endoscopy. 2020;52(11):1026-1035. doi: 10.1055/a-1178-9844. | Irrelevant Topic |
| 830 | Amano T, Nishida T, Shimakoshi H, Shimoda A, Osugi N, Sugimoto A, et al. Number of polyps detected is a useful indicator of quality of clinical colonoscopy. Endosc Int Open. 2018;6(7):E878-E884. doi: 10.1055/a-0605-3264. | Irrelevant Topic |
| 831 | Amin MF, Hassanin AM. Diagnostic performance of CT colonography with limited cathartic preparation in colorectal cancer screening; comparison with conventional colonoscopy. Egyptian Journal of Radiology and Nuclear Medicine. 2015;46(3):591‐598. doi: 10.1016/j.ejrnm.2015.05.012. | Irrelevant Topic |
| 832 | Anam AK, Karia K, Jesudian AB, Bosworth BP. Cirrhotic patients have worse bowel preparation at screening colonoscopy than chronic liver disease patients without cirrhosis. J Clin Exp Hepatol. 2016;6(4):297-302. doi: 10.1016/j.jceh.2016.08.009. | Irrelevant Topic |
| 833 | Anastassopoulos K, Farraye FA, Knight T, Colman S, Cleveland MV, Pelham RW. A comparative study of treatment-emergent adverse events following use of common bowel preparations among a colonoscopy screening population: results from a post-marketing observational study. Dig Dis Sci. 2016;61(10):2993-3006. doi: 10.1007/s10620-016-4214-2. | Irrelevant Topic |
| 834 | Anderson JC, Baron JA, Ahnen DJ, Barry EL, Bostick RM, Burke CA, et al. Factors associated with shorter colonoscopy surveillance intervals for patients with low-risk colorectal adenomas and effects on outcome. Gastroenterology. 2017;152(8):1933-1943.e1935. doi: 10.1053/j.gastro.2017.02.010. | Irrelevant Topic |
| 835 | Anderson JC, Butterly LF, Robinson CM, Goodrich M, Weiss JE. Impact of fair bowel preparation quality on adenoma and serrated polyp detection: data from the New Hampshire Colonoscopy Registry by using a standardized preparation-quality rating. Gastrointest Endosc. 2014;80(3):463-470. doi: 10.1016/j.gie.2014.03.021. | Irrelevant Topic |
| 836 | Anderson JC, Calderwood AH, Christensen BC, Robinson CM, Amos CI, Butterly L. Smoking and other risk factors in individuals with synchronous conventional high-risk adenomas and clinically significant serrated polyps. Am J Gastroenterol. 2018;113(12):1828-1835. doi: 10.1038/s41395-018-0393-0. | Irrelevant Topic |
| 837 | Anderson JC, Messina CR, Cohn W, Gottfried E, Ingber S, Bernstein G, et al. Factors predictive of difficult colonoscopy. Gastrointest Endosc. 2001;54(5):558-562. doi: 10.1067/mge.2001.118950. | Irrelevant Topic |
| 838 | Anderson JC, Robinson CM, Butterly LF. Young adults and metachronous neoplasia: risks for future advanced adenomas and large serrated polyps compared with older adults. Gastrointest Endosc. 2020;91(3):669-675. doi: 10.1016/j.gie.2019.11.023. | Irrelevant Topic |
| 839 | Anderson JC, Robinson CM, Hisey WM, Edwards DK, Kneedler BL, Berger BM, et al. Colorectal neoplasia detection in individuals with positive multitarget stool DNA tests: data from the New Hampshire Colonoscopy Registry. J Clin Gastroenterol. 2022;56(5):419-425. doi: 10.1097/mcg.0000000000001554. | Irrelevant Topic |
| 840 | Anderson R, Burr NE, Valori R. Causes of post-colonoscopy colorectal cancers based on World Endoscopy Organization system of analysis. Gastroenterology. 2020;158(5):1287-1299.e1282. doi: 10.1053/j.gastro.2019.12.031. | Irrelevant Topic |
| 841 | Andrealli A, Paggi S, Amato A, Rondonotti E, Imperiali G, Lenoci N, et al. Educational strategies for colonoscopy bowel prep overcome barriers against split-dosing: a randomized controlled trial. United European Gastroenterology Journal. 2018;6(2):283-289. doi: 10.1177/2050640617717157. | Irrelevant Topic |
| 842 | Anklesaria AB, Ivanina EA, Chudy-Onwugaje KO, Tin K, Levine CM, Homel P, et al. The effect of obesity on the quality of bowel preparation for colonoscopy: results from a large observational study. J Clin Gastroenterol. 2019;53(6):e214-e220. doi: 10.1097/mcg.0000000000001045. | Irrelevant Topic |
| 843 | Archer T, Corfe B, Dear K, Cole A, Foley S, Andreyev HJN, et al. Can an educational video improve the adequacy of bowel preparation for patients undergoing their first colonoscopy? Results of the EBOPS RCT. Endosc Int Open. 2023;12(3):E402‐E412. doi: 10.1055/a-2262-4023. | Irrelevant Topic |
| 844 | Arcovedo R, Larsen C, Reyes HS. Patient factors associated with a faster insertion of the colonoscope. Surgical Endoscopy and Other Interventional Techniques. 2007;21(6):885-888. doi: 10.1007/s00464-006-9116-5. | Irrelevant Topic |
| 845 | Arias F, Riverso M, Levy SA, Armstrong R, Estores DS, Tighe P, et al. Pilot study: neurocognitive disorders and colonoscopy in older adults. Anesth Analg. 2019;129(3):e89-e93. doi: 10.1213/ane.0000000000004212. | Irrelevant Topic |
| 846 | Arjunan S, Reddy N, Tandan M, Banerjee R. The frequency of bowel opening can be one of the predictors of quality of bowel preparation. J Gastroenterol Hepatol. 2013;28:118-119. | Irrelevant Topic |
| 847 | Armelao F, Paternolli C, Franceschini G, Franch R, Orlandi PG, Miori G, et al. Colonoscopic findings in first-degree relatives of patients with colorectal cancer: a population-based screening program. Gastrointest Endosc. 2011;73(3):527-534.e522. doi: 10.1016/j.gie.2010.12.025. | Irrelevant Topic |
| 848 | Armstrong D, Hollingworth R, MacIntosh D, Chen Y, Daniels S, Gittens S, et al. Point-of-care, peer-comparator colonoscopy practice audit: the Canadian Association of Gastroenterology Quality Program - Endoscopy. Canadian Journal of Gastroenterology and Hepatology. 2011;25(1):13-20. doi: 10.1155/2011/320904. | Irrelevant Topic |
| 849 | Arroja B, Cremers I, Ramos R, Cardoso C, Rego AC, Caldeira A, et al. Acute lower gastrointestinal bleeding management in Portugal: a multicentric prospective 1-year survey. European Journal of Gastroenterology & Hepatology. 2011;23(4):317-322. doi: 10.1097/MEG.0b013e328344ccb5. | Irrelevant Topic |
| 850 | Aslinia F, Uradomo L, Steele A, Greenwald BD, Raufman JP. Quality assessment of colonoscopic cecal intubation: an analysis of 6 years of continuous practice at a university hospital. Am J Gastroenterol. 2006;101(4):721-731. doi: 10.1111/j.1572-0241.2006.00494.x. | Irrelevant Topic |
| 851 | Atkin W, Brenner A, Martin J, Wooldrage K, Shah U, Lucas F, et al. The clinical effectiveness of different surveillance strategies to prevent colorectal cancer in people with intermediate-grade colorectal adenomas: a retrospective cohort analysis, and psychological and economic evaluations. Health Technology Assessment. 2017;21(25). doi: 10.3310/hta21250. | Irrelevant Topic |
| 852 | Atkin W, Wooldrage K, Brenner A, Martin J, Shah U, Perera S, et al. Adenoma surveillance and colorectal cancer incidence: a retrospective, multicentre, cohort study. Lancet Oncology. 2017;18(6):823-834. doi: 10.1016/S1470-2045(17)30187-0. | Irrelevant Topic |
| 853 | Auwyang JA, Setyoko S. Factors causing poor bowel preparation in colonoscopy. J Gastroenterol Hepatol. 2014;29:71-72. | Irrelevant Topic |
| 854 | Avalos DJ, Sussman DA, Lara LF, Sarkis FS, Castro FJ. Effect of diet liberalization on bowel preparation. South Med J. 2017;110(6):399-407. doi: 10.14423/smj.0000000000000662. | Irrelevant Topic |
| 855 | Badger SA, Gilliland R, Neilly PJ. The effectiveness of flexible sigmoidoscopy as the primary method for investigating colorectal symptoms in low-risk patients. Surg Endosc. 2005;19(10):1349-1352. doi: 10.1007/s00464-004-2215-2. | Irrelevant Topic |
| 856 | Badiani S, Hernandez ST, Karandikar S, Roy-Choudhury S. CT colonography to exclude colorectal cancer in symptomatic patients. European Radiology. 2011;21(10):2029-2038. doi: 10.1007/s00330-011-2151-1. | Irrelevant Topic |
| 857 | Baek SK, Bae OS, Jang BK. Endoscopic management of delayed perforation of the rectum caused by a peritoneal dialysis catheter. Surg Laparosc Endosc Percutan Tech. 2011;21(1):e44-47. doi: 10.1097/SLE.0b013e3182093fb4. | Irrelevant Topic |
| 858 | Bagshaw PF, Tuck AS, Aramowicz JM, Cox B, Frizelle FA, Church JM. Assessing guidelines on the need for colonoscopy after initial flexible sigmoidoscopy in young patients with outlet-type rectal bleeding. Diseases of the colon and rectum. 2024;67(1):160-167. doi: 10.1097/DCR.0000000000002947. | Irrelevant Topic |
| 859 | Baile-Maxía S, Mangas-Sanjuan C, Sala-Miquel N, Barquero C, Belda G, García-Del-Castillo G, et al. Incidence, characteristics, and predictive factors of post-colonoscopy colorectal cancer. United European Gastroenterol J. 2024;12(3):309-318. doi: 10.1002/ueg2.12512. | Irrelevant Topic |
| 860 | Bane-Maxie S, Amlani B, Martínez RJ. Bowel-cleansing efficacy of the 1L polyethylene glycol-based bowel preparation NER1006 (PLENVU) in patient subgroups in two phase III trials. Therap Adv Gastroenterol. 2021;14. doi: 10.1177/17562848211020286. | Irrelevant Topic |
| 861 | Barakat M, Panchal A, Abdelfatah MM, Elhanafi S, Carr-Locke DL, Othman MO. Morning versus afternoon adenoma detection rate: a systematic review and meta-analysis. European Journal of Gastroenterology & Hepatology. 2020;32(4):467-474. doi: 10.1097/MEG.0000000000001596. | Irrelevant Topic |
| 862 | Beaulieu D, Barkun AN, Dubé C, Tinmouth J, Hallé P, Martel M. Endoscopy reporting standards. Canadian Journal of Gastroenterology and Hepatology. 2013;27(5):286-292. doi: 10.1155/2013/145894. | Irrelevant Topic |
| 863 | Becq A, Chandnani M, Bharadwaj S, Baran B, Ernest-Suarez K, Gabr M, et al. Effectiveness of a deep-learning polyp detection system in prospectively collected colonoscopy videos with variable bowel preparation quality. J Clin Gastroenterol. 2020;54(6):554-557. doi: 10.1097/mcg.0000000000001272. | Irrelevant Topic |
| 864 | Belderbos TDG, Grobbee EJ, van Oijen MGH, Meijssen MAC, Ouwendijk RJT, Tang TJ, et al. Comparison of cecal intubation and adenoma detection between hospitals can provide incentives to improve quality of colonoscopy. Endoscopy. 2015;47(8):703-709. doi: 10.1055/s-0034-1391968. | Irrelevant Topic |
| 865 | Ben-Horin S, Bar-Meir S, Avidan B. The impact of colon cleanliness assessment on endoscopists' recommendations for follow-up colonoscopy. Am J Gastroenterol. 2007;102(12):2680-2685. doi: 10.1111/j.1572-0241.2007.01486.x. | Irrelevant Topic |
| 866 | Ben-Horin S, Bar-Meir S, Avidan B. The outcome of a second preparation for colonoscopy after preparation failure in the first procedure. Gastrointest Endosc. 2009;69(3):626-630. doi: 10.1016/j.gie.2008.08.027. | Irrelevant Topic |
| 867 | Beran A, Guardiola J, Mohamed MF, Aboursheid T, Rex DK, Beckelic ME. A post-hoc analysis of flavored polyethylene glycol and sulfate solution in adults with known predictors that may impact quality of bowel preparation for colonoscopy. Gastrointest Endosc. 2025;101(5). | Irrelevant Topic |
| 868 | Beran A, Sharma S, Ghazaleh S, Lee-Smith W, Aziz M, Kamal F, et al. Predictors of fecal microbiota transplant failure in Clostridioides difficile infection: an updated meta-analysis. J Clin Gastroenterol. 2023;57(4):389-399. doi: 10.1097/MCG.0000000000001667. | Irrelevant Topic |
| 869 | Bernstein C, Thorn M, Monsees K, Spell R, O'Connor JB. A prospective study of factors that determine cecal intubation time at colonoscopy. Gastrointest Endosc. 2005;61(1):72-75. doi: 10.1016/s0016-5107(04)02461-7. | Irrelevant Topic |
| 870 | Bessissow T, Van Keerberghen CA, Van Oudenhove L, Ferrante M, Vermeire S, Rutgeerts P, et al. Anxiety is associated with impaired tolerance of colonoscopy preparation in inflammatory bowel disease and controls. J Crohns Colitis. 2013;7(11):e580-587. doi: 10.1016/j.crohns.2013.04.011. | Irrelevant Topic |
| 871 | Bond AD, Burkitt MD, Sawbridge D, Corfe BM, Probert CS. Correlation between faecal tumour M2 pyruvate kinase and colonoscopy for the detection of adenomatous neoplasia in a secondary care cohort. J Gastrointestin Liver Dis. 2016;25(1):71-77. doi: 10.15403/jgld.2014.1121.251.m2p. | Irrelevant Topic |
| 872 | Bond JH. Should the quality of preparation impact postcolonoscopy follow-up recommendations? Am J Gastroenterol. 2007;102(12):2686-2687. doi: 10.1111/j.1572-0241.2007.01483.x. | Irrelevant Topic |
| 873 | Boo SJ, Yang DH, Jung KW, Kim KJ, Ye BD, Byeon JS, et al. Can rectal effluent predict the quality of bowel preparation at colonoscopy? J Gastroenterol Hepatol. 2011;26:113-113. | Irrelevant Topic |
| 874 | Borjas-Almaguer OD, Jimenez-Rodriguez AR, Cuellar-Monterrubio JE, Atilano-Díaz A, González JAG, Garcia-Compean D, et al. Digital rectal examination as a predictor of bowel preparation in patients undergoing colonoscopy. Gastrointest Endosc. 2019;89(6):AB439-AB439. doi: 10.1016/j.gie.2019.03.1237. | Irrelevant Topic |
| 875 | Bosch S, Acharjee A, Quraishi MN, Bijnsdorp IV, Rojas P, Bakkali A, et al. Integration of stool microbiota, proteome and amino acid profiles to discriminate patients with adenomas and colorectal cancer. Gut Microbes. 2022;14(1):2139979. doi: 10.1080/19490976.2022.2139979. | Irrelevant Topic |
| 876 | Bosch S, Acharjee A, Quraishi MN, Rojas P, Bakkali A, Jansen EE, et al. The potential of fecal microbiota and amino acids to detect and monitor patients with adenoma. Gut Microbes. 2022;14(1):2038863. doi: 10.1080/19490976.2022.2038863. | Irrelevant Topic |
| 877 | Bourquin V, Ponte B, Zellweger M, Levy M, Hadengue A, Moll S. [Phosphate nephropathy: how to avoid it?]. Rev Med Suisse. 2011;7(317):2227-2228, 2230-2221. | Irrelevant Topic |
| 878 | Bozkurt H, Sert OZ, Olmez T, Keklikkiran ZZ, Uzun O, Gulmez S, et al. The risk of post-polypectomy bleeding among patients receiving antithrombotic agents: a prospective observational study. Sao Paulo medical journal. 2021;139(3):218‐225. doi: 10.1590/1516-3180.2020.0305.R1.10122020. | Irrelevant Topic |
| 879 | Brajcich BC, Yang AD, Keswani RN, Kreutzer L, Molt PL, Rossi MB, et al. The quality of screening colonoscopy in rural and underserved areas. Surg Endosc. 2022;36(7):4845-4853. doi: 10.1007/s00464-021-08833-z. | Irrelevant Topic |
| 880 | Brenner H, Chang-Claude J, Jansen L, Seiler CM, Hoffmeister M. Role of colonoscopy and polyp characteristics in colorectal cancer after colonoscopic polyp detection: a population-based case-control study. Ann Intern Med. 2012;157(4):225-232. doi: 10.7326/0003-4819-157-4-201208210-00002. | Irrelevant Topic |
| 881 | Brinkmann M, von Holt I, Diedrich L, Krauth C, Seidel G, Dreier M. Attributes characterizing colorectal cancer screening tests that influence preferences of individuals eligible for screening in Germany: a qualitative study. Patient Preference and Adherence. 2022;16:2051-2066. doi: 10.2147/PPA.S365429. | Irrelevant Topic |
| 882 | Bronzwaer MES, Depla A, van Lelyveld N, Spanier BWM, Oosterhout YH, van Leerdam ME, et al. Quality assurance of colonoscopy within the Dutch national colorectal cancer screening program. Gastrointest Endosc. 2019;89(1):1-13. doi: 10.1016/j.gie.2018.09.011. | Irrelevant Topic |
| 883 | Bruzzi JF, Brennan DD, Fenlon HM. Trends in CT colonography. Curr Gastroenterol Rep. 2001;3(5):437-445. doi: 10.1007/s11894-001-0088-2. | Irrelevant Topic |
| 884 | Bryant RV, Schoeman SN, Schoeman MN. Shorter preparation to procedure interval for colonoscopy improves quality of bowel cleansing. Intern Med J. 2013;43(2):162-168. doi: 10.1111/j.1445-5994.2012.02963.x. | Irrelevant Topic |
| 885 | Bugajski M, Wieszczy P, Hoff G, Rupinski M, Regula J, Kaminski MF. Modifiable factors associated with patient-reported pain during and after screening colonoscopy. Gut. 2018;67(11):1958-1964. doi: 10.1136/gutjnl-2017-313905. | Irrelevant Topic |
| 886 | Burch J, Collins B. Chapter 10: investigating the gut. Virtual colonoscopy. Oxford Handbook of Gastrointestinal Nursing. New York, New York: Oxford University Press / New York; 2021. p. 229-229. | Irrelevant Topic |
| 887 | Burnett-Hartman AN, Hua X, Rue TC, Golchin N, Kessler L, Rowhani-Rahbar A. Risk interval analysis of emergency room visits following colonoscopy in patients with inflammatory bowel disease. Plos One. 2019;14(1):e0210262. doi: 10.1371/journal.pone.0210262. | Irrelevant Topic |
| 888 | Butterly LF, Siegel RL, Fedewa S, Robinson CM, Jemal A, Anderson JC. Colonoscopy outcomes in average-risk screening equivalent young adults: data from the New Hampshire Colonoscopy Registry. Am J Gastroenterol. 2021;116(1):171-179. doi: 10.14309/ajg.0000000000000820. | Irrelevant Topic |
| 889 | Cadoni S, Falt P, Sanna S, Argiolas M, Fanari V, Gallittu P, et al. Impact of colonoscopy insertion techniques on adenoma detection. Dig Dis Sci. 2016;61(7):2068-2075. doi: 10.1007/s10620-016-4053-1. | Irrelevant Topic |
| 890 | Caiado AHM, Nahas SC, Oliveira IRS, Ueda SKN, Nahas CSR, Rocha MS, et al. Accuracy of CT colonography with mannitol preparation for detecting synchronous lesions in obstructive colorectal cancer. Eur J Radiol. 2025;188:112151. doi: 10.1016/j.ejrad.2025.112151. | Irrelevant Topic |
| 891 | Cakar M, Kanbay M, Sarlak H, Akhan M, Gok M, Unal HU, et al. Findings of biopsy-proven chronicity and end-stage renal failure associated with oral sodium phosphate solution. Renal Failure. 2012;34(4):499-501. doi: 10.3109/0886022X.2011.654145. | Irrelevant Topic |
| 892 | Calderwood AH, Holub JL, Greenwald DA. Recommendations for follow-up interval after colonoscopy with inadequate bowel preparation in a national colonoscopy quality registry. Gastrointest Endosc. 2022;95(2):360-367.e362. doi: 10.1016/j.gie.2021.09.027. | Irrelevant Topic |
| 893 | Calderwood AH, Tosteson TD, Wang Q, Onega T, Walter LC. Association of life expectancy with surveillance colonoscopy findings and follow-up recommendations in older adults. JAMA Internal Medicine. 2023;183(5):426-434. doi: 10.1001/jamainternmed.2023.0078. | Irrelevant Topic |
| 894 | Capuñay CM, Carrascosa PM, Bou-Khair A, Castagnino N, Ninomiya I, Carrascosa JM. Low radiation dose multislice CT colonography in children: experience after 100 studies. Eur J Radiol. 2005;56(3):398-402. doi: 10.1016/j.ejrad.2005.06.002. | Irrelevant Topic |
| 895 | Cardin F, Minicuci N, Campigotto F, Andreotti A, Granziaera E, Donà B, et al. Difficult colonoscopies in the propofol era. BMC Surg. 2012;12 Suppl 1(Suppl 1):S9. doi: 10.1186/1471-2482-12-s1-s9. | Irrelevant Topic |
| 896 | Casais M, Rosa Diez G, Bravo S, Mansilla EN, Pérez S, Petkoff B, et al. [Is the preparation with sodium phosphate purgative for bowel cleansing safe in low-risk patients?]. Acta Gastroenterol Latinoam. 2010;40(1):54-60. | Irrelevant Topic |
| 897 | Casais MN, Rosa-Diez G, Pérez S, Mansilla EN, Bravo S, Bonofiglio FC. Hyperphosphatemia after sodium phosphate laxatives in low risk patients: prospective study. World J Gastroenterol. 2009;15(47):5960-5965. doi: 10.3748/wjg.15.5960. | Irrelevant Topic |
| 898 | Castellví J, Pi F, Sueiras A, Vallet J, Bollo J, Tomas A, et al. Colonoscopic perforation: useful parameters for early diagnosis and conservative treatment. Int J Colorectal Dis. 2011;26(9):1183-1190. doi: 10.1007/s00384-011-1211-y. | Irrelevant Topic |
| 899 | Cha JM, Kozarek RA, La Selva D, Gluck M, Ross A, Chiorean M, et al. Risks and benefits of colonoscopy in patients 90 years or older, compared with younger patients. Clin Gastroenterol Hepatol. 2016;14(1):80-86.e81. doi: 10.1016/j.cgh.2015.06.036. | Irrelevant Topic |
| 900 | Chan AOO, Lee LNW, Chan ACW, Ho WN, Chan QWL, Lau S, et al. Predictive factors for colonoscopy complications. Hong Kong Medical Journal. 2015;21(1):23-29. | Irrelevant Topic |
| 901 | Chan M, Birnstein E, Patel N, Chan L, Laine L, Kline M. Time interval between purgative completion and colonoscopy start time is an independent predictor of bowel preparation quality among Hispanic patients at a large county hospital. American Journal of Gastroenterology. 2011;106:S432-S432. | Irrelevant Topic |
| 902 | Chan WK, Azmi N, Mahadeva S, Goh KL. Split-dose vs same-day reduced-volume polyethylene glycol electrolyte lavage solution for morning colonoscopy. World Journal of Gastroenterology. 2014;20(39):14488‐14494. doi: 10.3748/wjg.v20.i39.14488. | Irrelevant Topic |
| 903 | Chan WK, Saravanan A, Manikam J, Goh KL, Mahadeva S. Appointment waiting times and education level influence the quality of bowel preparation in adult patients undergoing colonoscopy. BMC Gastroenterol. 2011;11:86. doi: 10.1186/1471-230x-11-86. | Irrelevant Topic |
| 904 | Chan YC, Chen CL, Wang CC, Lin CC, Yong CC, Chiu KW, et al. Extremity risk factors of sepsis for gastrointestinal endoscopy in patients with liver cirrhosis. Bmc Gastroenterology. 2022;22(1). doi: 10.1186/s12876-022-02124-0. | Irrelevant Topic |
| 905 | Chang JY, Moon CM, Lee HJ, Yang HJ, Jung Y, Kim SW, et al. Predictors for missed colorectal neoplasia at repeat colonoscopy in patients with suboptimal bowel preparation at index colonoscopy. Gastrointest Endosc. 2017;85(5):AB376-AB376. doi: 10.1016/j.gie.2017.03.870. | Irrelevant Topic |
| 906 | Chang JY, Moon CM, Lee HJ, Yang HJ, Jung Y, Kim SW, et al. Predictive factors for missed adenoma on repeat colonoscopy in patients with suboptimal bowel preparation on initial colonoscopy: a KASID multicenter study. Plos One. 2018;13(4):e0195709. doi: 10.1371/journal.pone.0195709. | Irrelevant Topic |
| 907 | Changela K, Malieckal A, Izzy MJ, Bhalani K, Lane D, Singhal S, et al. Mo1086 the predictors and outcomes of poor bowel preparation during colonoscopy: an analysis from colonoscopy outcome in minority population (COMP) registry. Gastroenterology. 2014;146(5):S553-S553. | Irrelevant Topic |
| 908 | Chatrenet P, Friocourt P, Ramain JP, Cherrier M, Maillard JB. Colonoscopy in the elderly: a study of 200 cases. Eur J Med. 1993;2(7):411-413. | Irrelevant Topic |
| 909 | Chawla T, Hurrell C, Keough V, Lindquist CM, Mohammed MF, Samson C, et al. Canadian Association of Radiologists practice guidelines for computed tomography colonography. Canadian Association of Radiologists Journal. 2024;75(1):54-68. doi: 10.1177/08465371231182975. | Irrelevant Topic |
| 910 | Chen HM, Chen CC, Chen CC, Wang SC, Wang CL, Huang CH, et al. Gut microbiome changes in overweight male adults following bowel preparation. BMC Genomics. 2018;19(Suppl 10):904. doi: 10.1186/s12864-018-5285-6. | Irrelevant Topic |
| 911 | Chen HX, Gao C, Li HY, Li CK, Wang C, Bai ZH, et al. Factors of easy and difficult cecal intubation during unsedated colonoscopy. Revista Espanola De Enfermedades Digestivas. 2023;115(10):546-552. doi: 10.17235/reed.2023.9283/2022. | Irrelevant Topic |
| 912 | Chen HY, Tu MH, Chen MY. Using a mobile health app (ColonClean) to enhance the effectiveness of bowel preparation: development and usability study. JMIR Hum Factors. 2025;12:e58479. doi: 10.2196/58479. | Irrelevant Topic |
| 913 | Chen L, Kang XY, Ren G, Luo H, Zhang LH, Wang LM, et al. Individualized intervention based on a preparation-related prediction model improves adequacy of bowel preparation: a prospective, multi-center, randomized, controlled study. Digestive and Liver Disease. 2024;56(3):436-443. doi: 10.1016/j.dld.2023.09.001. | Irrelevant Topic |
| 914 | Chen LA, Santos S, Jandorf L, Christie J, Castillo A, Winkel G, et al. A program to enhance completion of screening colonoscopy among urban minorities. Clinical Gastroenterology and Hepatology. 2008;6(4):443-450. doi: 10.1016/j.cgh.2007.12.009. | Irrelevant Topic |
| 915 | Chen SS, Zhang TT, Zhu SE, Zhou Y. A study on the related influencing factors of the quality of bowel preparation and the compliance of middle-aged and elderly patients for colonoscopy. Current Medical Research and Opinion. 2024;40(9):1545-1554. doi: 10.1080/03007995.2024.2384589. | Irrelevant Topic |
| 916 | Chen TA, Wong HY, Lin CK, Yu HC, Hsu PI, Lo GH, et al. High-dose bisacodyl plus water lavage compared with oral sodium phosphate as bowel preparation for outpatient colonoscopy. J Chin Med Assoc. 2009;72(8):402-407. doi: 10.1016/s1726-4901(09)70396-x. | Irrelevant Topic |
| 917 | Cheng CL, Liu NJ, Tang JH, Kuo YL, Hung HL, Tsui YN, et al. Predictors of suboptimal bowel preparation using 3-L of polyethylene glycol for an outpatient colonoscopy: a prospective observational study. Dig Dis Sci. 2017;62(2):345-351. doi: 10.1007/s10620-016-4343-7. | Irrelevant Topic |
| 918 | Cheng CL, Liu NJ, Tang JH, Kuo YL, Lin CH, Lien JM, et al. Risk of renal injury after the use of polyethylene glycol for outpatient colonoscopy: a prospective observational study. J Clin Gastroenterol. 2019;53(10):e444-e450. doi: 10.1097/mcg.0000000000001140. | Irrelevant Topic |
| 919 | Cheng CL, Tang JH, Hsieh YH, Kuo YL, Fang KC, Tseng CW, et al. Comparing right-sided colon adenoma and serrated polyp miss rates with water exchange and CO2 insufflation: a randomized controlled trial. Am J Gastroenterol. 2024;120(7):1615-1623. doi: 10.14309/ajg.0000000000003168. | Irrelevant Topic |
| 920 | Cheng CL, Tang JH, Hsieh YH, Kuo YL, Fang KC, Tseng CW, et al. Comparing right-sided colon adenoma and serrated polyp miss rates with water exchange and CO2 insufflation: a randomized controlled trial. American Journal of Gastroenterology. 2025;120(7):1615-1623. doi: 10.14309/ajg.0000000000003168. | Irrelevant Topic |
| 921 | Cheng P, Ma RJ, Wang SL, Fang J, Zhong ZR, Bai Y, et al. Effect of the high-FODMAP diet on bowel preparation for colonoscopy: a multicenter, prospective cohort study. Gastroenterol Res Pract. 2020;2020. doi: 10.1155/2020/1612040. | Irrelevant Topic |
| 922 | Cheng Y, Zhong C, Wu W, Xi X, Luo M, Chen Y, et al. Association between anxiety, depression, and bowel air bubbles at colonoscopy: a prospective observational study. Ann Palliat Med. 2021;10(3):3247-3257. doi: 10.21037/apm-21-540. | Irrelevant Topic |
| 923 | Chiang TH, Tan JH, Chang CC, Fang KC. Seizure from water intoxication following bowel preparation: a case report. BMC Nephrol. 2022;23(1):402. doi: 10.1186/s12882-022-03035-8. | Irrelevant Topic |
| 924 | Childers RE, Laird A, Newman L, Keyashian K. The role of a nurse telephone call to prevent no-shows in endoscopy. Gastrointest Endosc. 2016;84(6):1010-+. doi: 10.1016/j.gie.2016.05.052. | Irrelevant Topic |
| 925 | Cho SH, Kim HS, Park BS, Son GM, Park SB, Yun MS. Usefulness of intraoperative colonoscopy and synchronous scoring system for determining the integrity of the anastomosis in left-sided colectomy: a single-center retrospective cohort study. BMC Surg. 2025;25(1):116. doi: 10.1186/s12893-025-02836-6. | Irrelevant Topic |
| 926 | Choi CH. Lumpy or hard stool can be a simple predictor for inadequate bowel preparation prior to colonoscopy. American Journal of Gastroenterology. 2015;110:S644-S644. | Irrelevant Topic |
| 927 | Choi HI, Lee M, Cha JM. Obesity and body mass index are not associated with suboptimal quality of bowel preparation for colonoscopy. Dig Dis Sci. 2025;70(11):3671-3678. doi: 10.1007/s10620-025-09195-5. | Irrelevant Topic |
| 928 | Choi JM, Seo JY, Lee J, Han YM, Jin EH, Bae JH, et al. Longer withdrawal time is more important than excellent bowel preparation in colonoscopy of adequate bowel preparation. Dig Dis Sci. 2021;66(4):1168-1174. doi: 10.1007/s10620-020-06321-3. | Irrelevant Topic |
| 929 | Choi NK, Lee J, Chang Y, Jung SY, Kim YJ, Lee SM, et al. Polyethylene glycol bowel preparation does not eliminate the risk of acute renal failure: a population-based case-crossover study. Endoscopy. 2013;45(3):208-213. doi: 10.1055/s-0032-1326031. | Irrelevant Topic |
| 930 | Choi YI, Lee JJ, Chung JW, Kim KO, Kim YJ, Kim JH, et al. Efficacy and patient tolerability profiles of probiotic solution with bisacodyl versus conventional cleansing solution for bowel preparation: a prospective, randomized, controlled trial. J Clin Med. 2020;9(10). doi: 10.3390/jcm9103286. | Irrelevant Topic |
| 931 | Chorev N, Chadad B, Segal N, Shemesh I, Mor M, Plaut S, et al. Preparation for colonoscopy in hospitalized patients. Dig Dis Sci. 2007;52(3):835-839. doi: 10.1007/s10620-006-9591-5. | Irrelevant Topic |
| 932 | Chou CK, Chang CY, Chang CC, Chang LC, Hsu WF, Chen CY, et al. Controlled dietary restriction with a prepackaged low-residue diet before colonoscopy offers better-quality bowel cleansing and allows the use of a smaller volume of purgatives: a randomized multicenter trial. Dis Colon Rectum. 2016;59(10):975-983. doi: 10.1097/dcr.0000000000000675. | Irrelevant Topic |
| 933 | Chou MY, Hsu PI, Chou SL, Chou YM, Wang FW, Hsueh KC, et al. Factors related to incomplete flexible sigmoidoscopy among adult Chinese in Taiwan. J Chin Med Assoc. 2007;70(9):361-366. doi: 10.1016/s1726-4901(08)70021-2. | Irrelevant Topic |
| 934 | Christie JP, Marrazzo J, 3rd. "Mini-perforation" of the colon--not all postpolypectomy perforations require laparotomy. Dis Colon Rectum. 1991;34(2):132-135. doi: 10.1007/bf02049986. | Irrelevant Topic |
| 935 | Chu L, Liang JH, Wu SY, Jin X, Li HF, Tong XW. Comparative evaluation of enema alone and in combination with oral polyethylene glycol for bowel preparation before transvaginal pelvic floor reconstruction in elderly patients: a retrospective cohort study. Journal of Obstetrics and Gynaecology. 2022;42(6):2406-2410. doi: 10.1080/01443615.2022.2069002. | Irrelevant Topic |
| 936 | Chua HC, Pham S, Lombardi DA, Hot E, Mody L. Patient satisfaction scale following a laxative for antibiotic washout prior to oral microbiome therapy. Adv Ther. 2025;42(1):490-499. doi: 10.1007/s12325-024-03065-8. | Irrelevant Topic |
| 937 | Chun YJ, Yoon NR, Park JM, Lim CH, Cho YK, Lee IS, et al. Prospective assessment of risk of bacteremia following colorectal stent placement. Dig Dis Sci. 2012;57(4):1045-1049. doi: 10.1007/s10620-011-1962-x. | Irrelevant Topic |
| 938 | Chung GE, Lim SH, Yang SY, Song JH, Kang HY, Kang SJ, et al. Factors that determine prolonged cecal intubation time during colonoscopy: impact of visceral adipose tissue. Scand J Gastroenterol. 2014;49(10):1261-1267. doi: 10.3109/00365521.2014.950695. | Irrelevant Topic |
| 939 | Chung SY, Park SH, Lee SS, Lee JH, Kim AY, Park SK, et al. Comparison between CT colonography and double-contrast barium enema for colonic evaluation in patients with renal insufficiency. Korean J Radiol. 2012;13(3):290-299. doi: 10.3348/kjr.2012.13.3.290. | Irrelevant Topic |
| 940 | Chung YW, Han DS, Park KH, Kim KO, Park CH, Hahn T, et al. Patient factors predictive of inadequate bowel preparation using polyethylene glycol: a prospective study in Korea. J Clin Gastroenterol. 2009;43(5):448-452. doi: 10.1097/MCG.0b013e3181662442. | Irrelevant Topic |
| 941 | Chung YW, Han DS, Yoo KS, Park CK. Patient factors predictive of pain and difficulty during sedation-free colonoscopy: a prospective study in Korea. Dig Liver Dis. 2007;39(9):872-876. doi: 10.1016/j.dld.2007.04.019. | Irrelevant Topic |
| 942 | Clayton DB, Palmer WC, Robison SW, Heckman MG, Chimato NT, Harnois DM, et al. Colonoscopy bowel preparation quality improvement for patients with decompensated cirrhosis undergoing evaluation for liver transplantation. Clin Transplant. 2016;30(10):1236-1241. doi: 10.1111/ctr.12809. | Irrelevant Topic |
| 943 | Colic E, Marcussen N. [Acute phosphate nephropathy as a complication to bowel cleansing with oral sodium phosphate]. Ugeskr Laeger. 2011;173(50):3270-3271. | Irrelevant Topic |
| 944 | Collatuzzo G, Boffetta P, Radaelli F, Cadoni S, Hassan C, Frazzoni L, et al. Incidence, risk and protective factors of symptoms after colonoscopy. Dig Liver Dis. 2022;54(12):1698-1705. doi: 10.1016/j.dld.2022.08.025. | Irrelevant Topic |
| 945 | Colvin H, Lukram A, Sohail I, Chung KT, Jehangir E, Berry J, et al. The performance of routine computed tomography for the detection of colorectal cancer. Ann R Coll Surg Engl. 2013;95(7):473-476. doi: 10.1308/003588413x13629960049072. | Irrelevant Topic |
| 946 | Connor A, Tolan D, Hughes S, Carr N, Tomson C. Consensus guidelines for the safe prescription and administration of oral bowel-cleansing agents. Gut. 2012;61(11):1525-1532. doi: 10.1136/gutjnl-2011-300861. | Irrelevant Topic |
| 947 | Coronado GD, Johnson ES, Leo MC, Schneider JL, Smith D, Mummadi R, et al. Patient randomized trial of a targeted navigation program to improve rates of follow-up colonoscopy in community health centers. Contemp Clin Trials. 2020;89:105920. doi: 10.1016/j.cct.2019.105920. | Irrelevant Topic |
| 948 | Cottle C, Eluri S, Devault KR, Francis DL, Pang MY, Koop AH. Bowel preparation score is predictive of abnormal acid exposure in patients undergoing concurrent wireless ambulatory pH monitoring and colonoscopy. Gastroenterology. 2025;169(1). | Irrelevant Topic |
| 949 | Cowie BS, Buckley AB, Kluger R, Phan TD. The cardiovascular effects of crystalloid administration in endoscopy patients. Anaesth Intensive Care. 2019;47(1):45-51. doi: 10.1177/0310057x18811761. | Irrelevant Topic |
| 950 | Crispin A, Birkner B, Munte A, Nusko G, Mansmann U. Process quality and incidence of acute complications in a series of more than 230,000 outpatient colonoscopies. Endoscopy. 2009;41(12):1018-1025. doi: 10.1055/s-0029-1215214. | Irrelevant Topic |
| 951 | Crockett SD, Nagtegaal ID. Terminology, molecular features, epidemiology, and management of serrated colorectal neoplasia. Gastroenterology. 2019;157(4):949-966.e944. doi: 10.1053/j.gastro.2019.06.041. | Irrelevant Topic |
| 952 | Dale V, Essex H, Bloor K, Haritakis M, Jefferson L, Atkin K, et al. Patient experience of colon capsule endoscopy in clinical practice: a structured, comparative patient survey. BMJ Open Gastroenterol. 2025;12(1). doi: 10.1136/bmjgast-2025-001944. | Irrelevant Topic |
| 953 | Daly JM, Xu YH, Crockett SD, Hoffman RM, Levy BT. Is cognitive impairment associated with inadequate bowel preparation for colonoscopy? Journal of the American Board of Family Medicine. 2025;38(3):423-430. doi: 10.3122/jabfm.2024.240338R1. | Irrelevant Topic |
| 954 | Dang JT, Moolla M, Dang TT, Shaw A, Tian C, Karmali S, et al. Sodium phosphate is superior to polyethylene glycol in constipated patients undergoing colonoscopy: a systematic review and meta-analysis. Surg Endosc. 2021;35(2):900-909. doi: 10.1007/s00464-020-07464-0. | Irrelevant Topic |
| 955 | Daniels G, Schmelzer M, Handy N, Baker K. A national study comparing the tolerability and effectiveness of colon-cleansing preparations. Gastroenterol Nurs. 2012;35(3):182-191. doi: 10.1097/SGA.0b013e318255b94a. | Irrelevant Topic |
| 956 | De Cristofaro E, Marafini I, Mancone R, Fiorillo M, Franchin M, Mattogno A, et al. Preventable predictive factors of post-colonoscopy colorectal cancer in inflammatory bowel disease. Journal of Crohns & Colitis. 2024;19(1). doi: 10.1093/ecco-jcc/jjae115. | Irrelevant Topic |
| 957 | De Cristofaro E, Marafini I, Mancone R, Fiorillo M, Franchin M, Mattogno A, et al. Preventable predictive factors of post-colonoscopy colorectal cancer in inflammatory bowel disease. J Crohns Colitis. 2025;19(1). doi: 10.1093/ecco-jcc/jjae115. | Irrelevant Topic |
| 958 | de Meij TGJ, de Groot EFJ, Peeters CFW, de Boer NKH, Kneepkens CMF, Eck A, et al. Variability of core microbiota in newly diagnosed treatment-naïve paediatric inflammatory bowel disease patients. Plos One. 2018;13(8):e0197649. doi: 10.1371/journal.pone.0197649. | Irrelevant Topic |
| 959 | de Wijkerslooth TR, de Haan MC, Stoop EM, Bossuyt PM, Thomeer M, Essink-Bot ML, et al. Burden of colonoscopy compared to non-cathartic CT-colonography in a colorectal cancer screening programme: randomised controlled trial. Gut. 2012;61(11):1552‐1559. doi: 10.1136/gutjnl-2011-301308. | Irrelevant Topic |
| 960 | Deane C, Walker C, Ryan B, O'Connor A, O'Donnell S, Breslin N, et al. High diagnostic yield despite a lower completion rates for inpatient versus outpatient colon and pan-intestinal capsule endoscopy: a nested case-control study. Bmc Gastroenterology. 2023;23(1). doi: 10.1186/s12876-022-02561-x. | Irrelevant Topic |
| 961 | Deepak P, Ehrenpreis ED. Lower body weight and female gender: hyperphosphatemia risk factors after sodium phosphate preparations. World J Gastroenterol. 2011;17(21):2681-2682. doi: 10.3748/wjg.v17.i21.2681. | Irrelevant Topic |
| 962 | Denberg TD, Melhado TV, Coombes JM, Beaty BL, Berman K, Byers TE, et al. Predictors of nonadherence to screening colonoscopy. J Gen Intern Med. 2005;20(11):989-995. doi: 10.1111/j.1525-1497.2005.00164.x. | Irrelevant Topic |
| 963 | D'Haens G, Ferrante M, Vermeire S, Baert F, Noman M, Moortgat L, et al. Fecal calprotectin is a surrogate marker for endoscopic lesions in inflammatory bowel disease. Inflamm Bowel Dis. 2012;18(12):2218-2224. doi: 10.1002/ibd.22917. | Irrelevant Topic |
| 964 | Dhaliwal A, Buddam A, Chandan S, Bhat I, Singh S, Sayles H, et al. Development of a new bowel preparation prediction model based on patient's questionnaire: to proceed or not to proceed with colonoscopy? A study from Veterans Affairs hospital to stratify high risk population. American Journal of Gastroenterology. 2019;114:S99-S99. doi: 10.14309/01.ajg.0000590180.58499.da. | Irrelevant Topic |
| 965 | Dhaliwal AJ, Chandan S, Malik S, Sayles H, Antoniak D, Hewlett A, et al. Pre-colonoscopy Boston bowel preparation score assessment prediction model: to proceed or not to proceed with colonoscopy? A study from Veterans Affairs hospital. Gastroenterology. 2020;158(6):S1245-S1246. | Irrelevant Topic |
| 966 | Di Nardo G, Esposito F, Ziparo C, Strisciuglio C, Vassallo F, Di Serafino M, et al. Faecal calprotectin and ultrasonography as non-invasive screening tools for detecting colorectal polyps in children with sporadic rectal bleeding: a prospective study. Ital J Pediatr. 2020;46(1):66. doi: 10.1186/s13052-020-00828-1. | Irrelevant Topic |
| 967 | Dias E, Santos-Antunes J, GonÃ Alves D, Macedo G. Predictive factors and surgical impact of colonoscopy accuracy for localization of colorectal malignancy. Chirurgia (Bucur). 2022;117(5):535-543. doi: 10.21614/chirurgia.2791. | Irrelevant Topic |
| 968 | Dierick NR, Nicholson BD, Fanshawe TR, Sundaralingam P, Kostalas SN. Serrated polyposis syndrome: defining the epidemiology and predicting the risk of dysplasia. Bmc Gastroenterology. 2024;24(1). doi: 10.1186/s12876-024-03247-2. | Irrelevant Topic |
| 969 | Dinçer B, Ömeroğlu S, Güven O, Akgün İE, Celayir MF, Gürbulak EK, et al. Factors predict prolonged colonoscopy before the procedure: prospective registry study. Surg Endosc. 2024;38(10):5704-5711. doi: 10.1007/s00464-024-11075-4. | Irrelevant Topic |
| 970 | Ding YM, Lin XC. Outcomes and safety of colonoscopy in elderly patients aged 80 years and older: a retrospective cohort study. PeerJ. 2025;13. doi: 10.7717/peerj.20404. | Irrelevant Topic |
| 971 | Djinbachian R, Lafontaine ML, Dufault T, Medawar E, Boivin M, Bouin M, et al. Rates of synchronous advanced neoplasia and colorectal cancer in patients with colonic serrated lesions. Surg Endosc. 2023;37(7):5150-5157. doi: 10.1007/s00464-023-09974-z. | Irrelevant Topic |
| 972 | Dong H, Ren Y, Jiang B. Risk factors associated with missed colorectal lesions in colonoscopy and impact of colonoscopy with anesthesia on miss rate. Scand J Gastroenterol. 2021;56(4):484-491. doi: 10.1080/00365521.2021.1879248. | Irrelevant Topic |
| 973 | Dong-won L, Ja Seol K, Seonghee K, Seung Young K, Jong Jin H, Sung Woo J, et al. Association between bowel habits and quality of bowel preparation for colonoscopy. Medicine. 2017;96(29):1-5. doi: 10.1097/MD.0000000000007319. | Irrelevant Topic |
| 974 | Dore MP, Tufano MO, Pes GM, Cuccu M, Farina V, Manca A, et al. Tissue resonance interaction accurately detects colon lesions: a double-blind pilot study. World J Gastroenterol. 2015;21(25):7851-7859. doi: 10.3748/wjg.v21.i25.7851. | Irrelevant Topic |
| 975 | Doykov D, Andonov V. Risk factors and incidence of poor bowel preparation in elderly patients: prospective study. Folia Med (Plovdiv). 2019;61(3):370-376. doi: 10.3897/folmed.61.e39409. | Irrelevant Topic |
| 976 | Drago L, Valentina C, Fabio P. Gut microbiota, dysbiosis and colon lavage. Dig Liver Dis. 2019;51(9):1209-1213. doi: 10.1016/j.dld.2019.06.012. | Irrelevant Topic |
| 977 | Drewes JL, Rifkin SB, McMann M, Glass S, Spence E, Wensel CR, et al. Epidemiology of bacterial biofilms on polyps and normal tissues in a screening colonoscopy cohort. Gut Microbes. 2025;17(1):2452233. doi: 10.1080/19490976.2025.2452233. | Irrelevant Topic |
| 978 | Drügg Hahn G, Lakatos PL, Maedler-Kron C, Marcus V, Afif W, Wild G, et al. Defining fecal calprotectin cutoffs that predict endoscopic and histologic remission in patients with ulcerative colitis. Inflamm Bowel Dis. 2025;31(11):2977-2985. doi: 10.1093/ibd/izaf008. | Irrelevant Topic |
| 979 | Dubeau MF, Iacucci M, Beck PL, Moran GW, Kaplan GG, Ghosh S, et al. Drug-induced inflammatory bowel disease and IBD-like conditions. Inflammatory Bowel Diseases. 2013;19(2):445-456. doi: 10.1002/ibd.22990. | Irrelevant Topic |
| 980 | Dykes C, Cash BD. Key safety issues of bowel preparations for colonoscopy and importance of adequate hydration. Gastroenterol Nurs. 2008;31(1):30-35; quiz 36-37. doi: 10.1097/01.SGA.0000310933.54551.ca. | Irrelevant Topic |
| 981 | Early D, Larue S, Weinstock L, Kushnir V, Gyawali P, Sullivan S, et al. Impact of tilt-down positioning compared with left lateral positioning on ease of colonoscope insertion during colonoscopy. J Clin Gastroenterol. 2020;54(6):558-560. doi: 10.1097/mcg.0000000000001318. | Irrelevant Topic |
| 982 | Eckardt VF, Kanzier G, Willems D, Eckardt AJ, Bernhard G. Colonoscopy without premedication versus barium enema: a comparison of patient discomfort. Gastrointest Endosc. 1996;44(2):177-180. doi: 10.1016/s0016-5107(96)70136-0. | Irrelevant Topic |
| 983 | Edwards JT, Mendelson RM, Fritschi L, Foster NM, Wood C, Murray D, et al. Colorectal neoplasia screening with CT colonography in average-risk asymptomatic subjects: community-based study. Radiology. 2004;230(2):459-464. doi: 10.1148/radiol.2302021422. | Irrelevant Topic |
| 984 | Ehrenpreis ED. Increased serum phosphate levels and calcium fluxes are seen in smaller individuals after a single dose of sodium phosphate colon cleansing solution: a pharmacokinetic analysis. Aliment Pharmacol Ther. 2009;29(11):1202-1211. doi: 10.1111/j.1365-2036.2009.03987.x. | Irrelevant Topic |
| 985 | Elvas L, Brito D, Areia M, Carvalho R, Alves S, Saraiva S, et al. Impact of personalised patient education on bowel preparation for colonoscopy: prospective randomised controlled trial. GE Port J Gastroenterol. 2017;24(1):22‐30. doi: 10.1159/000450594. | Irrelevant Topic |
| 986 | Emara MH, Mazid U, Elshaer YA, Elkerdawy MA, Malik DF, Mahros AM. Trauma to the solid abdominal organs: the missed dark box of colonoscopy. World J Gastroenterol. 2024;30(7):624-630. doi: 10.3748/wjg.v30.i7.624. | Irrelevant Topic |
| 987 | Enestvedt BK, Brian Fennerty M, Zaman A, Eisen GM. MiraLAX vs. Golytely: is there a significant difference in the adenoma detection rate? Aliment Pharmacol Ther. 2011;34(7):775-782. doi: 10.1111/j.1365-2036.2011.04795.x. | Irrelevant Topic |
| 988 | English NC, Smith BP, Abdullah A, Gupta P, Oslock WM, Jones BA, et al. Socioecological determinants of health and the quality of colonoscopy in rural Alabama. Diseases of the Colon & Rectum. 2025;68(1):107-118. doi: 10.1097/DCR.0000000000003543. | Irrelevant Topic |
| 989 | Ennaifer R, Elleuch N, Sabbagh S, Romdhane H, Hefaiedh R, Ben Nejma H, et al. Quality indicators for colonoscopy in a Tunisian endoscopy unit. Tunis Med. 2015;93(3):138-141. | Irrelevant Topic |
| 990 | Epstein M, Halonen J, Sharma P. Bowel preparation with 1L polyethylene glycol and ascorbate NER1006 doubles the chance to detect three or more adenomas in overweight or obese males. Endosc Int Open. 2021;9(9):E1324‐E1334. doi: 10.1055/a-1499-6681. | Irrelevant Topic |
| 991 | Erben V, Carr PR, Guo F, Weigl K, Hoffmeister M, Brenner H. Individual and joint associations of genetic risk and healthy lifestyle score with colorectal neoplasms among participants of screening colonoscopy. Cancer Prevention Research. 2021;14(6):649-657. doi: 10.1158/1940-6207.CAPR-20-0576. | Irrelevant Topic |
| 992 | Erdogdu UE, Çayci HM, Tardu A, Arslan U, Demirci H, Yildirim Ç. Relationship between health literacy and quality of colonoscopy bowel preparation. Turkish Journal of Gastroenterology. 2020;31(11):799-804. doi: 10.5152/tjg.2020.19529. | Irrelevant Topic |
| 993 | Ergen WF, Pasricha T, Hubbard FJ, Higginbotham T, Givens T, Slaughter JC, et al. Providing hospitalized patients with an educational booklet increases the quality of colonoscopy bowel preparation. Clinical Gastroenterology and Hepatology. 2016;14(6):858-864. doi: 10.1016/j.cgh.2015.11.015. | Irrelevant Topic |
| 994 | Ergin GY, Ergin M, Özçelik M. Incidence and risk factors of orthostatic hypotension and postural tachycardia following sedated colonoscopy: a prospective observational study. Diagnostics. 2025;15(23). doi: 10.3390/diagnostics15233009. | Irrelevant Topic |
| 995 | Essex R, Cucos M, Dibley L. The impact of language and ethnicity on preparation for endoscopy: a prospective audit of an East London hospital ward. Journal of Evaluation in Clinical Practice. 2021;27(4):877-884. doi: 10.1111/jep.13490. | Irrelevant Topic |
| 996 | Evans B, Ellsmere J, Hossain I, Ennis M, O'Brien E, Bacque L, et al. Colonoscopy skills improvement training improves patient comfort during colonoscopy. Surg Endosc. 2022;36(6):4588-4592. doi: 10.1007/s00464-021-08753-y. | Irrelevant Topic |
| 997 | Evans B, Pace D, Borgaonkar M, Harnett J, Mine-Goldring M, Ge MM, et al. Effect of an educational intervention on colonoscopy quality outcomes. Surgical Endoscopy and Other Interventional Techniques. 2020;34(11):5142-5147. doi: 10.1007/s00464-019-07304-w. | Irrelevant Topic |
| 998 | Exsteen BB, Katzenstein TL, Faurholt-Jepsen D, Bjerregaard NC, Bader-Larsen K, Jensen-Fangel S, et al. Evaluation of the Danish colorectal cancer screening program among people with cystic fibrosis. J Clin Gastroenterol. 2025;59(8):753-757. doi: 10.1097/mcg.0000000000002083. | Irrelevant Topic |
| 999 | Facciorusso A, Buccino VR, Tonti P, Licinio R, Del Prete V, Neve V, et al. Impact of fellow participation on colon adenoma detection rates: a multicenter randomized trial. Gastrointest Endosc. 2020;92(6):1228-1235. doi: 10.1016/j.gie.2020.05.015. | Irrelevant Topic |
| 1000 | Fadel MG, Murray J, Woodfield G, Belluomo I, Laponogov I, Parker A, et al. Non-invasive breath testing to detect colorectal cancer: protocol for a multicentre, case-control development and validation study (COBRA2 study). BMC Cancer. 2025;25(1). doi: 10.1186/s12885-025-14520-2. | Irrelevant Topic |
| 1001 | Fang J, Fu HY, Ma D, Wang D, Liu YP, Wang YF, et al. Constipation, fiber intake and non-compliance contribute to inadequate colonoscopy bowel preparation: a prospective cohort study. J Dig Dis. 2016;17(7):458-463. doi: 10.1111/1751-2980.12376. | Irrelevant Topic |
| 1002 | Fang L, Zhou X, Wu M, Wang P, Xu Y. Relationship between perioperative e-health literacy and the quality of bowel preparation: a propensity score–matched, retrospective, observational study. Journal of Perianesthesia Nursing. 2025;40(6):1557-1566. doi: 10.1016/j.jopan.2025.03.004. | Irrelevant Topic |
| 1003 | Fang LY, Zhou XX, Wu MF, Wang PP, Xu YC. Relationship between perioperative e-health literacy and the quality of bowel preparation: a propensity score-matched, retrospective, observational study. Journal of Perianesthesia Nursing. 2025;40(6):1557-1566. doi: 10.1016/j.jopan.2025.03.004. | Irrelevant Topic |
| 1004 | Farrar WD, Sawhney MS, Nelson DB, Lederle FA, Bond JH. Colorectal cancers found after a complete colonoscopy. Clin Gastroenterol Hepatol. 2006;4(10):1259-1264. doi: 10.1016/j.cgh.2006.07.012. | Irrelevant Topic |
| 1005 | Fatima H, Johnson CS, Rex DK. Patients' description of rectal effluent and quality of bowel preparation at colonoscopy. Gastrointest Endosc. 2010;71(7):1244-1252.e1242. doi: 10.1016/j.gie.2009.11.053. | Irrelevant Topic |
| 1006 | Fayad NF, Kahi CJ, Abd El-Jawad KH, Shin AS, Shah S, Lane KA, et al. Association between body mass index and quality of split bowel preparation. Clin Gastroenterol Hepatol. 2013;11(11):1478-1485. doi: 10.1016/j.cgh.2013.05.037. | Irrelevant Topic |
| 1007 | Feng LA, Xu JX, Ji XT, Chen LP, Xing S, Liu B, et al. Development and validation of a three-dimensional deep learning-based system for assessing bowel preparation on colonoscopy video. Front Med (Lausanne). 2023;10. doi: 10.3389/fmed.2023.1296249. | Irrelevant Topic |
| 1008 | Fernandes C, Pinho R, Ribeiro I, Silva J, Ponte A, Carvalho J. Risk factors for polyp retrieval failure in colonoscopy. United European Gastroenterol J. 2015;3(4):387-392. doi: 10.1177/2050640615572041. | Irrelevant Topic |
| 1009 | Fevrier HB, Liu LY, Herrinton LJ, Li D. A transparent and adaptable method to extract colonoscopy and pathology data using natural language processing. Journal of Medical Systems. 2020;44(9). doi: 10.1007/s10916-020-01604-8. | Irrelevant Topic |
| 1010 | Fischbach W, Elsome R, Amlani B. Characteristics of right-sided colonic neoplasia and colonoscopy barriers limiting their early detection and prognosis: a review of the literature. Expert Rev Gastroenterol Hepatol. 2018;12(6):585-596. doi: 10.1080/17474124.2018.1478728. | Irrelevant Topic |
| 1011 | Flaherty DT, Winrich E, Eisa M, Poddar T, Pooler A, Kong MY, et al. Medical risk factors for inadequate bowel preparation prior to colonoscopy. Gastroenterology. 2022;162(7):S1029-S1029. | Irrelevant Topic |
| 1012 | Flanagan PK, Ahmed MS, Tin SM, Sarkar S. Service implications of implementing guidance for oral bowel cleansing agents in colonoscopy. J R Coll Physicians Edinb. 2011;41(2):100-105. doi: 10.4997/jrcpe.2011.202. | Irrelevant Topic |
| 1013 | Fletcher JG, Johnson CD, Welch TJ, MacCarty RL, Ahlquist DA, Reed JE, et al. Optimization of CT colonography technique: prospective trial in 180 patients. Radiology. 2000;216(3):704-711. doi: 10.1148/radiology.216.3.r00au41704. | Irrelevant Topic |
| 1014 | Fletcher JG, Silva AC, Fidler JL, Cernigliaro JG, Manduca A, Limburg PJ, et al. Noncathartic CT colonography: image quality assessment and performance in a screening cohort. AJR Am J Roentgenol. 2013;201(4):787-794. doi: 10.2214/ajr.12.9225. | Irrelevant Topic |
| 1015 | Flor N, Mezzanzanica M, Rigamonti P, Rocco EG, Bosari S, Ceretti AP, et al. Contrast-enhanced computed tomography colonography in preoperative distinction between T1-T2 and T3-T4 staging of colon cancer. Academic radiology. 2013;20(5):590-595. doi: 10.1016/j.acra.2013.01.008. | Irrelevant Topic |
| 1016 | Fok KC, Turner IB, Teoh WC, Levy RL. Obesity does not affect sodium picosulphate bowel preparation. Intern Med J. 2012;42(12):1324-1329. doi: 10.1111/j.1445-5994.2012.02865.x. | Irrelevant Topic |
| 1017 | Fostier R, Tziatzios G, Facciorusso A, Papaefthymiou A, Arvanitakis M, Triantafyllou K, et al. Models and scores to predict adequacy of bowel preparation before colonoscopy. Best Pract Res Clin Gastroenterol. 2023;67:101859. doi: 10.1016/j.bpg.2023.101859. | Irrelevant Topic |
| 1018 | Franco F, Najum PH, Rodríguez PC. Quality of bowel preparation and inadequate cleansing predictors in patients undergoing colonoscopy prior preparation with polyethylene glycol: inpatient versus outpatient. Gastrointest Endosc. 2016;83(5):AB227-AB227. doi: 10.1016/j.gie.2016.03.301. | Irrelevant Topic |
| 1019 | Frazzoni L, La Marca M, Di Giorgio V, Laterza L, Bazzoli F, Hassan C, et al. Endoscopic surveillance after surgery for colorectal cancer. Minerva Medica. 2023;114(2):224-236. doi: 10.23736/S0026-4806.20.06732-4. | Irrelevant Topic |
| 1020 | Frazzoni L, Radaelli F, Spada C, Mussetto A, Frazzoni M, Laterza L, et al. The diagnostic yield of colonoscopy in hospitalized patients. An observational multicenter prospective study. Dig Liver Dis. 2021;53(2):224-230. doi: 10.1016/j.dld.2020.10.029. | Irrelevant Topic |
| 1021 | Freitas M, Silva VM, Xavier S, Carvalho PB, Rosa B, Moreira MJ, et al. Prolonged gastric transit time in small-bowel capsule endoscopy: which patients are at risk and what are the implications? Turkish Journal of Gastroenterology. 2023;34(3):227-233. doi: 10.5152/tjg.2023.22191. | Irrelevant Topic |
| 1022 | Friedman AC, Downing D, Chino J, Krupinski E, Kilian C, Lance P. Feasibility of remote CT colonography at two rural Native American medical centers. AJR Am J Roentgenol. 2010;195(5):1110-1117. doi: 10.2214/ajr.10.4383. | Irrelevant Topic |
| 1023 | Frigerio G, Imperiali G, Lenoci N, Sangiovanni A, Spinzi GC, Strocchi E, et al. High doses of Senna for colon cleansing. Giornale italiano di endoscopia digestiva. 1996;19(3):181‐184. | Irrelevant Topic |
| 1024 | Fuccio L, Collatuzzo G, Frazzoni L, Cadoni S, Anderloni A, Laterza L, et al. Impact of colonoscopy on working productivity: a prospective multicenter observational study. Gastrointest Endosc. 2022;95(3):550-561.e558. doi: 10.1016/j.gie.2021.11.039. | Irrelevant Topic |
| 1025 | Fuccio L, Frazzoni L, Spada C, Mussetto A, Fabbri C, Manno M, et al. How to identify hospitalized patients at higher risk of inadequate colon cleansing for colonoscopy: an observational multicentre prospective study and a predictive model. Digestive and Liver Disease. 2020;52:S14-S15. | Irrelevant Topic |
| 1026 | Fuccio L, Spada C, Frazzoni L, Paggi S, Vitale G, Laterza L, et al. Higher adenoma recurrence rate after left- versus right-sided colectomy for colon cancer. Gastrointest Endosc. 2015;82(2):337-343. doi: 10.1016/j.gie.2014.12.057. | Irrelevant Topic |
| 1027 | Fudman DI, Singal AG, Cooper MG, Lee M, Murphy CC. Prevalence of forceps polypectomy of nondiminutive polyps is substantial but modifiable. Clin Gastroenterol Hepatol. 2022;20(7):1508-1515. doi: 10.1016/j.cgh.2021.11.031. | Irrelevant Topic |
| 1028 | Gan T, Yang JL, Wu JC, Wang YP, Yang L. When and why a colonoscopist should discontinue colonoscopy by himself? World J Gastroenterol. 2015;21(25):7834-7841. doi: 10.3748/wjg.v21.i25.7834. | Irrelevant Topic |
| 1029 | Gandhi K, Tofani C, Sokach C, Patel D, Kastenberg D, Daskalakis C. Patient characteristics associated with quality of colonoscopy preparation: a systematic review and meta-analysis. Clin Gastroenterol Hepatol. 2018;16(3):357-369.e310. doi: 10.1016/j.cgh.2017.08.016. | Irrelevant Topic |
| 1030 | Gao H, Liu CQ, Yuan X, Lu HP, Xia YH, Zhang ZX, et al. Face-to-face instruction and personalized regimens improve the quality of inpatient bowel preparation for colonoscopy. Dig Dis Sci. 2022;67(8):3592-3600. doi: 10.1007/s10620-021-07290-x. | Irrelevant Topic |
| 1031 | García-Borruel MM, Molina AJH, Perálvarez MLR, Rincón EM, Medrano IP, Ruiz FJS, et al. Post-colonoscopy colorectal cancer: characteristics and predictive factors. Medicina Clinica. 2018;150(1):1-7. doi: 10.1016/j.medcli.2017.04.015. | Irrelevant Topic |
| 1032 | Garcia-Planella E, Mañosa M, Cabré E, Marín L, Gordillo J, Zabana Y, et al. Fecal calprotectin levels are closely correlated with the absence of relevant mucosal lesions in postoperative Crohn's disease. Inflamm Bowel Dis. 2016;22(12):2879-2885. doi: 10.1097/mib.0000000000000960. | Irrelevant Topic |
| 1033 | Gausman V, Quarta G, Lee MH, Chtourmine N, Ganotisi C, Nanton-Gonzalez F, et al. A theory-based educational pamphlet with low-residue diet improves colonoscopy attendance and bowel preparation quality. J Clin Gastroenterol. 2020;54(2):164-169. doi: 10.1097/MCG.0000000000001151. | Irrelevant Topic |
| 1034 | Gautam V, Khanna R, Sood V, Lal BB, Laroia ST, Bhatia V, et al. Splenic-Z-score predicts severe portal hypertensive changes on colonoscopy in infants and children with biliary atresia. J Pediatr Gastroenterol Nutr. 2025;81(2):183-191. doi: 10.1002/jpn3.70079. | Irrelevant Topic |
| 1035 | Gavin DR, Valori RM, Anderson JT, Donnelly MT, Williams JG, Swarbrick ET. The national colonoscopy audit: a nationwide assessment of the quality and safety of colonoscopy in the UK. Gut. 2013;62(2):242-249. doi: 10.1136/gutjnl-2011-301848. | Irrelevant Topic |
| 1036 | Gawron AJ, Horner B, Zurbuchen R, Boynton K, Fang JC. A comprehensive intervention to enhance inpatient colon preparation quality for colonoscopy. Minerva Gastroenterology. 2023;69(3):351-358. doi: 10.23736/S2724-5985.21.02766-5. | Irrelevant Topic |
| 1037 | Gay G, Delvaux M, Frederic M, Fassler I. Could the colonic capsule PillCam Colon be clinically useful for selecting patients who deserve a complete colonoscopy? Results of clinical comparison with colonoscopy in the perspective of colorectal cancer screening. Am J Gastroenterol. 2010;105(5):1076-1086. doi: 10.1038/ajg.2009.624. | Irrelevant Topic |
| 1038 | Ghanouni A, Halligan S, Plumb A, Boone D, Wardle J, von Wagner C. Non- or full-laxative CT colonography vs. endoscopic tests for colorectal cancer screening: a randomised survey comparing public perceptions and intentions to undergo testing. European Radiology. 2014;24(7):1477-1486. doi: 10.1007/s00330-014-3187-9. | Irrelevant Topic |
| 1039 | Ghanouni A, Halligan S, Taylor SA, Boone D, Plumb A, Stoffel S, et al. Quantifying public preferences for different bowel preparation options prior to screening CT colonography: a discrete choice experiment. Bmj Open. 2014;4(4). doi: 10.1136/bmjopen-2013-004327. | Irrelevant Topic |
| 1040 | Gimeno-García AZ, Alayón-Miranda S, Benítez-Zafra F, Hernández-Negrín D, Nicolás-Pérez D, Pérez Cabañas C, et al. Design and validation of an artificial intelligence system to detect the quality of colon cleansing before colonoscopy. Gastroenterol Hepatol. 2024;47(5):481-490. doi: 10.1016/j.gastrohep.2023.12.009. | Irrelevant Topic |
| 1041 | Gimeno-García AZ, Benítez-Zafra F, Hernández A, Hernandez-Negrín D, Nicolás-Pérez D, Hernández G, et al. Agreement between the perception of colon cleansing reported by patients and colon cleansing assessed by a validated colon cleansing scale. Gastroenterol Hepatol. 2024;47(2):130-139. doi: 10.1016/j.gastrohep.2023.02.009. | Irrelevant Topic |
| 1042 | Gimeno-García AZ, Benítez-Zafra F, Nicolás-Pérez D, Hernández-Guerra M. Colon bowel preparation in the era of artificial intelligence: is there potential for enhancing colon bowel cleansing? Medicina (Kaunas). 2023;59(10). doi: 10.3390/medicina59101834. | Irrelevant Topic |
| 1043 | Gimeno-García AZ, de la Barreda-Heuser R, Reygosa C, Hernández A, Amaral C, Mascareño I, et al. Increasing the low residue diet to 3 days does not improve the bowel cleansing in hard to prepare patients: post hoc analysis of a randomized controlled trial. Gastroenterol Hepatol. 2021;44(3):183-190. doi: 10.1016/j.gastrohep.2020.06.016. | Irrelevant Topic |
| 1044 | Gimeno-García AZ, Hernández G, Dorta JLB, Reygosa C, de la Barreda R, Hernandez-Bustabad A, et al. An enhanced high-volume preparation for colonoscopy is not better than a conventional low-volume one in patients at risk of poor bowel cleansing: a randomized controlled trial. Front Med (Lausanne). 2021;8. doi: 10.3389/fmed.2021.654847. | Irrelevant Topic |
| 1045 | Gimeno-García AZ, Hernández-Pérez A, Nicolás-Pérez D, Hernández-Guerra M. Artificial intelligence applied to colonoscopy: is it time to take a step forward? Cancers (Basel). 2023;15(8). doi: 10.3390/cancers15082193. | Irrelevant Topic |
| 1046 | Gimeno-García AZ, Sacramento-Luis D, Ashok-Bhagchandani R, Nicolás-Pérez D, Hernández-Guerra M. Interventions to improve bowel cleansing in colonoscopy. Expert Rev Gastroenterol Hepatol. 2025;19(1):39-51. doi: 10.1080/17474124.2025.2450699. | Irrelevant Topic |
| 1047 | Gimeno-García AZ, Sacramento-Luis D, Cámara-Suárez M, Díaz-Beunza M, Delgado-Martín R, Cubas-Cubas AT, et al. Comparative study of predictive models for the detection of patients at high risk of inadequate colonic cleansing. J Pers Med. 2024;14(1). doi: 10.3390/jpm14010102. | Irrelevant Topic |
| 1048 | Gkolfakis P, Kapizioni C, Tziatzios G, Facciorusso A, Frazzoni L, Thomopoulos K, et al. Comparative performance and external validation of three different scores in predicting inadequate bowel preparation among Greek inpatients undergoing colonoscopy. Ann Gastroenterol. 2023;36(1):25-31. doi: 10.20524/aog.2023.0757. | Irrelevant Topic |
| 1049 | Gkolfakis P, Tziatzios G, Papanikolaou IS, Triantafyllou K. Strategies to improve inpatients' quality of bowel preparation for colonoscopy: a systematic review and meta-analysis. Gastroenterol Res Pract. 2019;2019. doi: 10.1155/2019/5147208. | Irrelevant Topic |
| 1050 | Gönczi L, Kürti Z, Golovics P, Vegh Z, Lovász B, Dorkó A, et al. Indications, diagnoses and quality markers in upper and lower endoscopies in 2010 and 2011 at the 1st Department of Medicine, Semmelweis University, Budapest. Orvosi Hetilap. 2016;157(52):2074-2081. doi: 10.1556/650.2016.30602. | Irrelevant Topic |
| 1051 | Gordon M, Nigam GB, Sinopoulou V, Al Bakir I, Bateman AC, Din S, et al. Update from 2010 (standard operating procedure): protocol for the 2024 British Society of Gastroenterology guidelines on colorectal surveillance in inflammatory bowel disease. BMJ Open Gastroenterol. 2024;11(1). doi: 10.1136/bmjgast-2024-001541. | Irrelevant Topic |
| 1052 | Gornall RJ, Talbot R. Can flexible sigmoidoscopy predict need for colorectal surgery in ovarian carcinoma? Eur J Gynaecol Oncol. 1999;20(1):13-15. | Irrelevant Topic |
| 1053 | Gory I, Brown G, Wilson J, Kemp W, Paul E, Roberts SK. Increased risk of colorectal neoplasia in adult patients with cystic fibrosis: a matched case-control study. Scand J Gastroenterol. 2014;49(10):1230-1236. doi: 10.3109/00365521.2014.936035. | Irrelevant Topic |
| 1054 | Gottlieb K, Daperno M, Usiskin K, Sands BE, Ahmad H, Howden CW, et al. Endoscopy and central reading in inflammatory bowel disease clinical trials: achievements, challenges and future developments. Gut. 2021;70(2):418-426. doi: 10.1136/gutjnl-2020-320690. | Irrelevant Topic |
| 1055 | Gottlieb K, Requa J, Karnes W, Chandra Gudivada R, Shen J, Rael E, et al. Central reading of ulcerative colitis clinical trial videos using neural networks. Gastroenterology. 2021;160(3):710-719.e712. doi: 10.1053/j.gastro.2020.10.024. | Irrelevant Topic |
| 1056 | Govani SN, Elliott EE, Menees SB, Judd SL, Saini SD, Anastassiades CP, et al. Predictors of sub-optimal bowel preparation for screening colonoscopy. Gastroenterology. 2011;140(5):S560-S560. | Irrelevant Topic |
| 1057 | Gow-Lee B, Gaumnitz J, Alsadhan M, Garg G, Amoafo L, Zhang Y, et al. Cirrhosis and portal hypertension worsen bowel preparation for screening colonoscopy. J Clin Gastroenterol. 2025;59(1):82-89. doi: 10.1097/mcg.0000000000001990. | Irrelevant Topic |
| 1058 | Grant LA, Griffin N, Shaw A. Two-year audit of computed tomographic colonography in a teaching hospital: are we meeting the standard? Colorectal Dis. 2010;12(4):373-379. doi: 10.1111/j.1463-1318.2009.01824.x. | Irrelevant Topic |
| 1059 | Grobbee EJ, Kapidzic A, van Vuuren AJ, van Leerdam M, Lansdorp-Vogelaar I, Looman CW, et al. Second-look colonoscopies and the impact on capacity in FIT-based colorectal cancer screening. Am J Gastroenterol. 2015;110(7):1072-1077. doi: 10.1038/ajg.2015.157. | Irrelevant Topic |
| 1060 | Grode LB, Dragnes Brix L. Factors causing incomplete colonoscopy reported by the endoscopist: a population-based study. Gastroenterol Nurs. 2025;48(3):153-160. doi: 10.1097/sga.0000000000000874. | Irrelevant Topic |
| 1061 | Groza AL, Miutescu B, Tefas C, Popa A, Ratiu I, Sirli R, et al. Evaluating the efficacy of resect-and-discard and resect-and-retrieve strategies for diminutive colonic polyps. Life-Basel. 2024;14(4). doi: 10.3390/life14040532. | Irrelevant Topic |
| 1062 | Guardiola-Arévalo A, Navacerada AG, García-Alonso FJ, Checa PB, Becerra RP, Guerra I, et al. Randomized clinical trial evaluating the effect of a visual educational leaflet on the preparation of colonoscopies in hospitalized patients. Revista Espanola De Enfermedades Digestivas. 2019;111(12):946-952. doi: 10.17235/reed.2019.6317/2019. | Irrelevant Topic |
| 1063 | Gumurdulu Y, Serin E, Ozer B, Gokcel A, Boyacioglu S. Age as a predictor of hyperphosphatemia after oral phosphosoda administration for colon preparation. J Gastroenterol Hepatol. 2004;19(1):68-72. doi: 10.1111/j.1440-1746.2004.03253.x. | Irrelevant Topic |
| 1064 | Guo BM, Zuo XL, Li Z, Liu J, Xu N, Li XH, et al. Improving the quality of bowel preparation through an app for inpatients undergoing colonoscopy: a randomized controlled trial. Journal of Advanced Nursing. 2020;76(4):1037-1045. doi: 10.1111/jan.14295. | Irrelevant Topic |
| 1065 | Guo C, Wang J, Li L, Cui J, Liu H, Yang G. Effects of the combined use of linaclotide and oral sulfate solution in bowel preparation for patients with chronic constipation undergoing colonoscopy: protocol of a prospective, randomised, controlled, single-blind clinical trial from a single centre in China. Bmj Open. 2025;15(5):e099687. doi: 10.1136/bmjopen-2025-099687. | Irrelevant Topic |
| 1066 | Guo XF, Yu XA, Hu JC, Lin DZ, Deng JX, Su ML, et al. Endoscopic management of delayed bleeding after polypectomy of small colorectal polyps: two or more clips may be safe. Gastroenterol Rep (Oxf). 2022;10. doi: 10.1093/gastro/goab051. | Irrelevant Topic |
| 1067 | Gupta A, Saini SD, Naylor KB. Increased driving distance to screening colonoscopy negatively affects bowel preparation quality: an observational study. J Gen Intern Med. 2021;36(6):1666-1672. doi: 10.1007/s11606-020-06464-z. | Irrelevant Topic |
| 1068 | Gupta AK, Brenner DE, Turgeon DK. Early detection of colon cancer - new tests on the horizon. Molecular Diagnosis & Therapy. 2008;12(2):77-85. doi: 10.1007/BF03256273. | Irrelevant Topic |
| 1069 | Gupta T, Mandot A, Desai D, Abraham P, Joshi A, Shah S. Comparison of two schedules (previous evening versus same morning) of bowel preparation for colonoscopy. Endoscopy. 2007;39(8):706-709. doi: 10.1055/s-2007-966375. | Irrelevant Topic |
| 1070 | Gupta V, East JE. Optimal endoscopic treatment and surveillance of serrated polyps. Gut and Liver. 2020;14(4):423-429. doi: 10.5009/gnl19202. | Irrelevant Topic |
| 1071 | Gurudu SR, Ratuapli S, Heigh R, DiBaise J, Leighton J, Crowell M. Quality of bowel cleansing for afternoon colonoscopy is influenced by time of administration. American Journal of Gastroenterology. 2010;105(11):2318-2322. doi: 10.1038/ajg.2010.235. | Irrelevant Topic |
| 1072 | Gwag M, Yoo J. Relationship between health literacy and knowledge, compliance with bowel preparation, and bowel cleanliness in older patients undergoing colonoscopy. International Journal of Environmental Research and Public Health. 2022;19(5). doi: 10.3390/ijerph19052676. | Irrelevant Topic |
| 1073 | Hadjiliadis D, Khoruts A, Zauber AG, Hempstead SE, Maisonneuve P, Lowenfels AB. Cystic fibrosis colorectal cancer screening consensus recommendations. Gastroenterology. 2018;154(3):736-745.e714. doi: 10.1053/j.gastro.2017.12.012. | Irrelevant Topic |
| 1074 | Hahn GD, Lakatos PL, Maedler-Kron C, Marcus V, Afif W, Wild G, et al. Defining fecal calprotectin cutoffs that predict endoscopic and histologic remission in patients with ulcerative colitis. Inflammatory Bowel Diseases. 2025;31(11):2977-2985. doi: 10.1093/ibd/izaf008. | Irrelevant Topic |
| 1075 | Han JY, Moosvi Z, Duh E, Park S, Albers GC, Samarasena JB, et al. Oral IBGard™ before colonoscopy: a single-center double-blinded, randomized, placebo-controlled trial. Dig Dis Sci. 2021;66(5):1611‐1619. doi: 10.1007/s10620-020-06383-3. | Irrelevant Topic |
| 1076 | Hande KA. Measuring endoscopic performance for colorectal cancer prevention quality improvement in a gastroenterology practice. Nursing Clinics of North America. 2014;49(1):15-27. doi: 10.1016/j.cnur.2013.11.007. | Irrelevant Topic |
| 1077 | Hansmann A, Burling D. Essential requirements of a CT colonography service. European Journal of Radiology. 2013;82(8):1187-1191. doi: 10.1016/j.ejrad.2012.03.018. | Irrelevant Topic |
| 1078 | Harewood GC, Wright CA, Baron TH. Assessment of patients' perceptions of bowel preparation quality at colonoscopy. Am J Gastroenterol. 2004;99(5):839-843. doi: 10.1111/j.1572-0241.2004.04176.x. | Irrelevant Topic |
| 1079 | Hart L, Chavannes M, Kherad O, Maedler C, Mourad N, Marcus V, et al. Faecal calprotectin predicts endoscopic and histological activity in clinically quiescent ulcerative colitis. J Crohns Colitis. 2020;14(1):46-52. doi: 10.1093/ecco-jcc/jjz107. | Irrelevant Topic |
| 1080 | Hartmann D, Bassler B, Schilling D, Adamek HE, Jakobs R, Pfeifer B, et al. Colorectal polyps: detection with dark-lumen MR colonography versus conventional colonoscopy. Radiology. 2006;238(1):143-149. doi: 10.1148/radiol.2381041756. | Irrelevant Topic |
| 1081 | Hasan AG, Brown WR. Colonic cleansing for colonoscopy: a risk to be taken seriously. Gastrointest Endosc. 2011;73(3):616-618. doi: 10.1016/j.gie.2010.11.050. | Irrelevant Topic |
| 1082 | Hassall E, Lobe TE. Risks of oral sodium phosphate for precolonoscopy bowel preparation in children. Gastrointest Endosc. 2007;65(7):1102-1104; author reply 1104-1105. doi: 10.1016/j.gie.2007.02.046. | Irrelevant Topic |
| 1083 | Hassan C, Manning J, Alvarez Gonzalez MA, Sharma P, Epstein M, Bisschops R. Improved detection of colorectal adenomas by high-quality colon cleansing. Endosc Int Open. 2020;8(7):E928‐E937. doi: 10.1055/a-1167-1359. | Irrelevant Topic |
| 1084 | Hassan C, Wysocki PT, Fuccio L, Seufferlein T, Dinis-Ribeiro M, Brandao C, et al. Endoscopic surveillance after surgical or endoscopic resection for colorectal cancer: European Society of Gastrointestinal Endoscopy (ESGE) and European Society of Digestive Oncology (ESDO) guideline. Endoscopy. 2019;51(3):266-277. doi: 10.1055/a-0831-2522. | Irrelevant Topic |
| 1085 | Hatoum HT, Lin SJ, Joseph RE, Dahdal DN. Validation of a patient satisfaction scale in patients undergoing bowel preparation prior to colonoscopy. Patient. 2016;9(1):27-34. doi: 10.1007/s40271-015-0154-8. | Irrelevant Topic |
| 1086 | Hawkins AT, Wise PE, Chan T, Lee JT, Glyn T, Wood V, et al. Diverticulitis: an update from the age old paradigm. Curr Probl Surg. 2020;57(10):100862. doi: 10.1016/j.cpsurg.2020.100862. | Irrelevant Topic |
| 1087 | Haydel JM, Xu AA, Mansour NM. High volume, low volume, or pills, which way should we go? A review of bowel preparation for colonoscopy. Curr Opin Gastroenterol. 2024;40(1):21-26. doi: 10.1097/mog.0000000000000983. | Irrelevant Topic |
| 1088 | Hayes A, Buffum M, Hughes J. Diabetic colon preparation comparison study. Gastroenterol Nurs. 2011;34(5):377-382. doi: 10.1097/SGA.0b013e31822c3a24. | Irrelevant Topic |
| 1089 | Hayman AV, Guihan M, Fisher MJ, Murphy D, Anaya BC, Parachuri R, et al. Colonoscopy is high yield in spinal cord injury. J Spinal Cord Med. 2013;36(5):436-442. doi: 10.1179/2045772313y.0000000091. | Irrelevant Topic |
| 1090 | He B, Xin Y, Li R, Lin FC, Zhang GM, Zhu HJ. Bowel preparation experiences and needs before follow-up colonoscopy in older adult postoperative colorectal cancer patients: a qualitative study. World J Gastrointest Surg. 2024;16(9). doi: 10.4240/wjgs.v16.i9.2968. | Irrelevant Topic |
| 1091 | He DG, Chen JG, Jiang XF, Chen H, Huang JN, Chen ZX. Risk factors for synchronous high-risk polyps in patients with colorectal cancer. Front Surg. 2024;11. doi: 10.3389/fsurg.2024.1424809. | Irrelevant Topic |
| 1092 | Heher EC, Thier SO, Rennke H, Humphreys BD. Adverse renal and metabolic effects associated with oral sodium phosphate bowel preparation. Clin J Am Soc Nephrol. 2008;3(5):1494-1503. doi: 10.2215/cjn.02040408. | Irrelevant Topic |
| 1093 | Herity L, Wolf L, Altepeter T, deFonseka A, Meyer J, Munoz M, et al. Syncope in patients using colonoscopy preparation products containing sodium picosulfate, magnesium oxide, and anhydrous citric acid... Digestive Disease Week, May 18-21, 2024, Washington, D.C. Gastrointest Endosc. 2024;99(6):AB315-AB315. doi: 10.1016/j.gie.2024.04.1232. | Irrelevant Topic |
| 1094 | Hernández G, Gimeno-García AZ, Quintero E. Strategies to optimise the quality of bowel cleansing. Gastroenterol Hepatol. 2019;42(5):326-338. doi: 10.1016/j.gastrohep.2019.01.010. | Irrelevant Topic |
| 1095 | Hernández G, Gimeno-García AZ, Quintero E. Strategies to improve inadequate bowel preparation for colonoscopy. Front Med (Lausanne). 2019;6. doi: 10.3389/fmed.2019.00245. | Irrelevant Topic |
| 1096 | Hernandez PV, Horsley-Silva JL, Snyder DL, Baffy N, Atia M, Koepke L, et al. Effect of bowel preparation volume in inpatient colonoscopy. Results of a prospective, randomized, comparative pilot study. Bmc Gastroenterology. 2020;20(1):227. doi: 10.1186/s12876-020-01373-1. | Irrelevant Topic |
| 1097 | Heron V, Martel M, Bessissow T, Chen YI, Désilets E, Dube C, et al. Comparison of the Boston bowel preparation scale with an auditable application of the US Multi-Society task force guidelines. J Can Assoc Gastroenterol. 2019;2(2):57-62. doi: 10.1093/jcag/gwy027. | Irrelevant Topic |
| 1098 | Heron V, Parmar R, Ménard C, Martel M, Barkun AN. Validating bowel preparation scales. Endosc Int Open. 2017;5(12):E1179‐E1188. doi: 10.1055/s-0043-119749. | Irrelevant Topic |
| 1099 | Heymann TD, Chopra K, Nunn E, Coulter L, Westaby D, Murray-Lyon IM. Bowel preparation at home: prospective study of adverse effects in elderly people. Bmj. 1996;313(7059):727-728. doi: 10.1136/bmj.313.7059.727. | Irrelevant Topic |
| 1100 | Higashimori A, Maeda N, Nakatani M, Yamamoto I, Yanagida T, Kin D, et al. Importance of bowel habits in predicting inadequate bowel preparation: a prospective observational study. Dig Dis Sci. 2023;68(11):4148-4155. doi: 10.1007/s10620-023-08089-8. | Irrelevant Topic |
| 1101 | Higashimori A, Natsumi M, Nakatani M, Nakamura Y, Hashimoto A, Morimoto K, et al. Age-specific risk factors of inadequate bowel preparation for colonoscopy: a prospective questionnaire-based observational study. Gastrointest Endosc. 2020;91(6):AB555-AB555. | Irrelevant Topic |
| 1102 | Hijos-Mallada G, Lué A, Velamazan R, Saura N, Abril C, Lorenzo M, et al. The addition of other fecal biomarkers does not improve the diagnostic accuracy of immunochemical fecal occult blood test alone in a colorectal cancer screening cohort. Front Med (Lausanne). 2021;8. doi: 10.3389/fmed.2021.665786. | Irrelevant Topic |
| 1103 | Hisada H, Tsuji Y, Cho R, Miura Y, Mizutani H, Ohki D, et al. Feasibility of colorectal endoscopic submucosal dissection in elderly patients: the impact of sarcopenia. Digestion. 2024;105(3):175-185. doi: 10.1159/000536267. | Irrelevant Topic |
| 1104 | Ho LH, Montealegre JR, Al-Arabi S, Jibaja-Weiss ML, Suarez MG. Impact of colonoscopy preparation video on Boston bowel preparation scale score. Gastroenterology Nursing. 2019;42(3):251-258. doi: 10.1097/SGA.0000000000000391. | Irrelevant Topic |
| 1105 | Ho SB, Hovsepians R, Gupta S. Optimal bowel cleansing for colonoscopy in the elderly patient. Drugs Aging. 2017;34(3):163-172. doi: 10.1007/s40266-017-0436-z. | Irrelevant Topic |
| 1106 | Hoff G, Vatn M, Gjone E, Larsen S, Sauar J. Epidemiology of polyps in the rectum and sigmoid colon. Design of a population screening study. Scand J Gastroenterol. 1985;20(3):351-355. doi: 10.3109/00365528509091663. | Irrelevant Topic |
| 1107 | Hoffman A, Murthy S, Pompetzki L, Rey JW, Goetz M, Tresch A, et al. Intraprocedural bowel cleansing with the JetPrep cleansing system improves adenoma detection. World J Gastroenterol. 2015;21(26):8184-8194. doi: 10.3748/wjg.v21.i26.8184. | Irrelevant Topic |
| 1108 | Hoffmanová I, Kraml P, Anděl M. Renal risk associated with sodium phosphate medication: safe in healthy individuals, potentially dangerous in others. Expert Opin Drug Saf. 2015;14(7):1097-1110. doi: 10.1517/14740338.2015.1044970. | Irrelevant Topic |
| 1109 | Holleran TJ, Nigam A, Houlihan BK, Berkey SE, FitzGerald JF, Ayscue JM, et al. Incidental polyp on colectomy specimen is linked with higher odds of polyp on interval colonoscopy. Journal of Surgical Research. 2021;268:158-167. doi: 10.1016/j.jss.2021.06.028. | Irrelevant Topic |
| 1110 | Holt EW, Yimam KK, Ma H, Shaw RE, Sundberg RA, Verhille MS. Patient tolerability of bowel preparation is associated with polyp detection rate during colonoscopy. J Gastrointestin Liver Dis. 2014;23(2):135-140. doi: 10.15403/jgld.2014.1121.232.ewh1. | Irrelevant Topic |
| 1111 | Hookey L, Bertiger G, Johnson KL, Boules M, Ando M, Dandal DN. Efficacy, safety, and tolerability of a ready-to-drink bowel preparation: subanalysis by age from a phase III, assessor-blinded study. Therap Adv Gastroenterol. 2020;13. doi: 10.1177/1756284820902878. | Irrelevant Topic |
| 1112 | Hookey L, Bertiger G, Lee Johnson K, Ayala J, Seifu Y, Brogadir SP. Efficacy and safety of a ready-to-drink bowel preparation for colonoscopy: a randomized, controlled, non-inferiority trial. Therap Adv Gastroenterol. 2019;12:1756284819851510. doi: 10.1177/1756284819851510. | Irrelevant Topic |
| 1113 | Hopkins RL, Parsons D, Hoyo L, Jacobson BC. Evaluating the practice of canceling colonoscopies for presumed inadequate bowel preparation. Gastrointest Endosc. 2020;92(2):382-386. doi: 10.1016/j.gie.2020.03.3750. | Irrelevant Topic |
| 1114 | Hotta N, Ohmiya N, Hiraga H, Nakaji K, Osawa S, Omori T, et al. Nationwide multicenter prospective study on the usefulness, safety, and acceptability of colon capsule endoscopy in Japan. Gastrointest Endosc. 2025;101(5):1051-1063.e1057. doi: 10.1016/j.gie.2024.11.004. | Irrelevant Topic |
| 1115 | Hovsepians R, Liu L, Yang M, Maples M, Groessl EJ, Gupta S, et al. Sa1070 predictive factors for identifying patients with inadequate bowel preparation... Digestive Disease Week (DDW) 2017 American Society for Gastrointestinal Endoscopy (ASGE) Program and Abstracts, Chicago, Illinois, 6-9 May 2017. Gastrointest Endosc. 2017;85:AB179-AB179. doi: 10.1016/j.gie.2017.03.389. | Irrelevant Topic |
| 1116 | Hsieh YH, Kuo CS, Tseng KC, Lin HJ. Factors that predict cecal insertion time during sedated colonoscopy: the role of waist circumference. J Gastroenterol Hepatol. 2008;23(2):215-217. doi: 10.1111/j.1440-1746.2006.04818.x. | Irrelevant Topic |
| 1117 | Hsu CM, Lin WP, Su MY, Chiu CT, Ho YP, Chen PC. Factors that influence cecal intubation rate during colonoscopy in deeply sedated patients. J Gastroenterol Hepatol. 2012;27(1):76-80. doi: 10.1111/j.1440-1746.2011.06795.x. | Irrelevant Topic |
| 1118 | Huang JS, Choi L, Lin TK, Lightdale JR, Walsh CM. Numbers matter: how pediatric endoscopy quality varies with annual procedural volume. J Pediatr Gastroenterol Nutr. 2025;81(6):1488-1495. doi: 10.1002/jpn3.70212. | Irrelevant Topic |
| 1119 | Huang L. Diabetes-specific bowel preparation: evidence synthesis on risk factors and multimodal interventions. Gastrointestinal Nursing. 2025;23(Suppl 8):S6-S12. doi: 10.12968/gasn.2025.0082. | Irrelevant Topic |
| 1120 | Huang X, Zhang S, Sui X, Wei J, Zhang H, Wang R, et al. Efficacy of re-examination across left colon on adenoma detection: a multicenter randomized controlled trial. SSRN. 2025. doi: 10.2139/ssrn.5345101. | Irrelevant Topic |
| 1121 | Huang Z, Cheng W, Chao K, Tang J, Li M, Guo Q, et al. Baseline and postinduction intestinal ultrasound findings predict long-term transmural and mucosal healing in patients with Crohn's disease. Inflammatory Bowel Diseases. 2024;30(10):1767-1775. doi: 10.1093/ibd/izad251. | Irrelevant Topic |
| 1122 | Hundt S, Haug U, Brenner H. Comparative evaluation of immunochemical fecal occult blood tests for colorectal adenoma detection. Ann Intern Med. 2009;150(3):162-169. doi: 10.7326/0003-4819-150-3-200902030-00005. | Irrelevant Topic |
| 1123 | Hurlstone DP, Brown S, Cross SS, Shorthouse AJ, Sanders DS. High magnification chromoscopic colonoscopy or high frequency 20 MHz mini probe endoscopic ultrasound staging for early colorectal neoplasia: a comparative prospective analysis. Gut. 2005;54(11):1585-1589. doi: 10.1136/gut.2005.069849. | Irrelevant Topic |
| 1124 | Hussar DA. New therapeutic agents marketed in the second half of 2012: part 2. Pharmacy Today. 2013;19(3):73-84. doi: 10.1016/S1042-0991(15)31506-1. | Irrelevant Topic |
| 1125 | Hwang KL, Chen WT, Hsiao KH, Chen HC, Huang TM, Chiu CM, et al. Prospective randomized comparison of oral sodium phosphate and polyethylene glycol lavage for colonoscopy preparation. World J Gastroenterol. 2005;11(47):7486-7493. doi: 10.3748/wjg.v11.i47.7486. | Irrelevant Topic |
| 1126 | Iacucci M, Cannatelli R, Parigi TL, Nardone OM, Tontini GE, Labarile N, et al. A virtual chromoendoscopy artificial intelligence system to detect endoscopic and histologic activity/remission and predict clinical outcomes in ulcerative colitis. Endoscopy. 2022;55(4):332-341. doi: 10.1055/a-1960-3645. | Irrelevant Topic |
| 1127 | Ianiro G, Valerio L, Masucci L, Pecere S, Bibbò S, Quaranta G, et al. Predictors of failure after single faecal microbiota transplantation in patients with recurrent Clostridium difficile infection: results from a 3-year, single-centre cohort study. Clin Microbiol Infect. 2017;23(5):337.e331-337.e333. doi: 10.1016/j.cmi.2016.12.025. | Irrelevant Topic |
| 1128 | Ikeda T, Yoshizaki T, Eguchi T, Kinugasa H, Okada A. Efficacy of specimen pasting after cold snare polypectomy for pathological evaluation of horizontal margins. Endosc Int Open. 2022;10(5):E572‐E579. doi: 10.1055/a-1784-6723. | Irrelevant Topic |
| 1129 | Iqbal CW, Cullinane DC, Schiller HJ, Sawyer MD, Zietlow SP, Farley DR. Surgical management and outcomes of 165 colonoscopic perforations from a single institution. Arch Surg. 2008;143(7):701-706; discussion 706-707. doi: 10.1001/archsurg.143.7.701. | Irrelevant Topic |
| 1130 | Ishibashi K, Urabe Y, Uda T, Sako Y, Gurita T, Masuda S, et al. Effectiveness and safety of cold snare polypectomy in intensive downstaging polypectomy for colorectal polyps in patients with familial adenomatous polyposis. Digestion. 2025. doi: 10.1159/000548434. | Irrelevant Topic |
| 1131 | Iussich G, Correale L, Senore C, Hassan C, Segnan N, Campanella D, et al. Computer-aided detection for computed tomographic colonography screening: a prospective comparison of a double-reading paradigm with first-reader computer-aided detection against second-reader computer-aided detection. Investigative radiology. 2014;49(3):173‐182. doi: 10.1097/RLI.0000000000000009. | Irrelevant Topic |
| 1132 | J VS, Müller-Steden B. Screening colonoscopy and colorectal cancer: a single center long term study. Z Gastroenterol. 2016;54(4):312-315. doi: 10.1055/s-0041-110808. | Irrelevant Topic |
| 1133 | Jackson CS, Haq T, Olafsson S. Push enteroscopy has a 96% cecal intubation rate in colonoscopies that failed because of redundant colons. Gastrointest Endosc. 2011;74(2):341-346. doi: 10.1016/j.gie.2011.04.015. | Irrelevant Topic |
| 1134 | Jacob A, Schafer A, Yong J, Tonkin D, Rodda D, Eteuati J, et al. Endocuff Vision-assisted colonoscopy: a randomized controlled trial. ANZ J Surg. 2019;89(5):E174-e178. doi: 10.1111/ans.15067. | Irrelevant Topic |
| 1135 | Jacobs CC, Jaber JF, Ladna M, Ponniah S, Madhok I, Ruiz N, et al. Factors associated with inpatient endoscopy delay and its impact on hospital length-of-stay and 30-day readmission. Clin Gastroenterol Hepatol. 2021;19(12):2648-2655. doi: 10.1016/j.cgh.2021.06.009. | Irrelevant Topic |
| 1136 | Jacobson BC, Anderson JC, Burke CA, Dominitz JA, Gross SA, May FP, et al. Optimizing bowel preparation quality for colonoscopy: consensus recommendations by the US Multi-Society Task Force on Colorectal Cancer. Gastrointest Endosc. 2025;101(4):702-732. doi: 10.1016/j.gie.2025.02.010. | Irrelevant Topic |
| 1137 | Jain D, Anand S, Singhal S. Use of modifiable and non-modifiable factors to predict bowel preparation quality prior to colonoscopy. American Journal of Gastroenterology. 2014;109:S678-S678. | Irrelevant Topic |
| 1138 | Jamil KM, Jacomb-Hood JH, Fidler HM. Investigating the frail elderly patient with lower bowel symptoms: what do we do now and can we improve? Clin Med (Lond). 2013;13(1):37-41. doi: 10.7861/clinmedicine.13-1-37. | Irrelevant Topic |
| 1139 | Jang HW, Cheon JH, Nam CM, Moon CM, Lee JH, Jeon SM, et al. Factors affecting insertion time for colonoscopy performed under intramuscular analgesia in patients with history of colorectal resection. Surg Endosc. 2011;25(7):2316-2322. doi: 10.1007/s00464-010-1555-3. | Irrelevant Topic |
| 1140 | Jang HW, Park SJ, Hong SP, Cheon JH, Kim WH, Kim TI. Risk factors for recurrent high-risk polyps after the removal of high-risk polyps at initial colonoscopy. Yonsei Med J. 2015;56(6):1559-1565. doi: 10.3349/ymj.2015.56.6.1559. | Irrelevant Topic |
| 1141 | Janssen L, van Linschoten RCA, West RL, Gilissen LPL, Romberg-Camps M, Brandts L, et al. Predicting mucosal inflammation in IBD patients using patient-reported symptom scores and a faecal calprotectin home test: protocol for a multicentre prospective validation study. Bmj Open. 2024;14(10):e076290. doi: 10.1136/bmjopen-2023-076290. | Irrelevant Topic |
| 1142 | Jaruvongvanich V, Sempokuya T, Laoveeravat P, Ungprasert P. Risk factors associated with longer cecal intubation time: a systematic review and meta-analysis. Int J Colorectal Dis. 2018;33(4):359-365. doi: 10.1007/s00384-018-3014-x. | Irrelevant Topic |
| 1143 | Jensch S, Bipat S, Peringa J, de Vries AH, Heutinck A, Dekker E, et al. CT colonography with limited bowel preparation: prospective assessment of patient experience and preference in comparison to optical colonoscopy with cathartic bowel preparation. Eur Radiol. 2010;20(1):146-156. doi: 10.1007/s00330-009-1517-0. | Irrelevant Topic |
| 1144 | Jensch S, de Vries AH, Peringa J, Bipat S, Dekker E, Baak LC, et al. CT colonography with limited bowel preparation: performance characteristics in an increased-risk population. Radiology. 2008;247(1):122-132. doi: 10.1148/radiol.2471070439. | Irrelevant Topic |
| 1145 | Jha AK, Chaudhary M, Jha P, Kumar U, Dayal VM, Jha SK, et al. Polyethylene glycol plus bisacodyl: a safe, cheap, and effective regimen for colonoscopy in the South Asian patients. JGH open: an open access journal of gastroenterology and hepatology. 2018;2(6):249‐254. doi: 10.1002/jgh3.12077. | Irrelevant Topic |
| 1146 | Jia H, Guo XG, Kang XY, Zhang XF, Pan YL. Pre-procedure model predicts bowel preparation quality prior colonoscopy. Gastrointest Endosc. 2020;91(6):AB546-AB547. | Irrelevant Topic |
| 1147 | Jia H, Wang LM, Luo H, Yao SW, Wang XP, Zhang LH, et al. Difficult colonoscopy score identifies the difficult patients undergoing unsedated colonoscopy. Bmc Gastroenterology. 2015;15. doi: 10.1186/s12876-015-0273-7. | Irrelevant Topic |
| 1148 | Jiao FF, Liu ZF, Shen YC, Cao JJ, Gao Y, Wang H, et al. [Influencing factors for the quality of bowel preparation with polyethylene glycol electrolyte powder combined with diet control before colonoscopy in children]. Zhongguo Dang Dai Er Ke Za Zhi. 2022;24(4):366-371. doi: 10.7499/j.issn.1008-8830.2111019. | Irrelevant Topic |
| 1149 | John GK, Thuluvath AJ, Carrier H, Ahuja NK, Gupta E, Stein E. Poor health literacy and medication burden are significant predictors for inadequate bowel preparation in an urban tertiary care setting. J Clin Gastroenterol. 2019;53(9):e382-e386. doi: 10.1097/MCG.0000000000001177. | Irrelevant Topic |
| 1150 | Johnson CD, Chen MH, Toledano AY, Heiken JP, Dachman A, Kuo MD, et al. Accuracy of CT colonography for detection of large adenomas and cancers. N Engl J Med. 2008;359(12):1207-1217. doi: 10.1056/NEJMoa0800996. | Irrelevant Topic |
| 1151 | Johnson DA, Barclay RL, Mergener K, Weiss G, König T, Beck J, et al. Plasma Septin9 versus fecal immunochemical testing for colorectal cancer screening: a prospective multicenter study. Plos One. 2014;9(6):e98238. doi: 10.1371/journal.pone.0098238. | Irrelevant Topic |
| 1152 | Johnson MR, Grubber J, Grambow SC, Maciejewski ML, Dunn-Thomas T, Provenzale D, et al. Physician non-adherence to colonoscopy interval guidelines in the Veterans Affairs healthcare system. Gastroenterology. 2015;149(4):938-951. doi: 10.1053/j.gastro.2015.06.026. | Irrelevant Topic |
| 1153 | Jover R, Herráiz M, Alarcón O, Brullet E, Bujanda L, Bustamante M, et al. Clinical practice guidelines: quality of colonoscopy in colorectal cancer screening. Endoscopy. 2012;44(4):444-451. doi: 10.1055/s-0032-1306690. | Irrelevant Topic |
| 1154 | Jover R, Zapater P, Polanía E, Bujanda L, Lanas A, Hermo JA, et al. Modifiable endoscopic factors that influence the adenoma detection rate in colorectal cancer screening colonoscopies. Gastrointest Endosc. 2013;77(3):381-389.e381. doi: 10.1016/j.gie.2012.09.027. | Irrelevant Topic |
| 1155 | Jung H, Lee JI, Huh CW, Kim MJ, Youn YN, Choi YH, et al. Withdrawal time of 8 minutes is associated with higher adenoma detection rates in surveillance colonoscopy after surgery for colorectal cancer. Surgical Endoscopy and Other Interventional Techniques. 2021;35(5):2354-2361. doi: 10.1007/s00464-020-07653-x. | Irrelevant Topic |
| 1156 | Jung JH, Kim JW, Lee HW, Park MY, Paik WH, Bae WK, et al. Acute hemorrhagic rectal ulcer syndrome: comparison with non-hemorrhagic rectal ulcer lower gastrointestinal bleeding. J Dig Dis. 2017;18(9):521-528. doi: 10.1111/1751-2980.12513. | Irrelevant Topic |
| 1157 | Jung YH. Taking a long and hard look at quality predictors of bowel preparation for colonoscopy. Dig Dis Sci. 2023;68(11):4069-4070. doi: 10.1007/s10620-023-08090-1. | Irrelevant Topic |
| 1158 | Jung YS, Lee CK, Kim HJ, Eun CS, Han DS, Park DI. Randomized controlled trial of sodium phosphate tablets vs polyethylene glycol solution for colonoscopy bowel cleansing. World Journal of Gastroenterology. 2014;20(42):15845‐15851. doi: 10.3748/wjg.v20.i42.15845. | Irrelevant Topic |
| 1159 | Juul FE, Garborg K, Nesbakken E, Løberg M, Wieszczy P, Cubiella J, et al. Rates of repeated colonoscopies to clean the colon from low-risk and high-risk adenomas: results from the EPoS trials. Gut. 2023;72(5):951-957. doi: 10.1136/gutjnl-2022-327696. | Irrelevant Topic |
| 1160 | Jyala A, Ghazanfar H, Kandhi SD, Qasim A, Sosa F, Lajara P, et al. Predictors of quality of bowel preparation for in-patient colonoscopy: a retrospective study in a tertiary care hospital in South Bronx, NY. Gastrointest Endosc. 2024;99(6):AB434-AB435. | Irrelevant Topic |
| 1161 | Kaan HL, Khor V, Liew WC, Loh TF, Leong SW, Teo SL, et al. The efficacy of on-demand sedation colonoscopy: a STROBE-compliant retrospective cohort study. Surg Endosc. 2022;36(2):930-935. doi: 10.1007/s00464-021-08351-y. | Irrelevant Topic |
| 1162 | Kabir C, Salazar Leon M, Ndiaye C, Flicker M. Effect of acuity level and patient characteristics on bowel preparation quality: a retrospective cohort study of inpatient colonoscopies. BMC Gastroenterol. 2023;23(1):126. doi: 10.1186/s12876-023-02751-1. | Irrelevant Topic |
| 1163 | Kamiński MF, Hassan C, Bisschops R, Pohl J, Pellisé M, Dekker E, et al. Advanced imaging for detection and differentiation of colorectal neoplasia: European Society of Gastrointestinal Endoscopy (ESGE) guideline. Endoscopy. 2014;46(5):435-449. doi: 10.1055/s-0034-1365348. | Irrelevant Topic |
| 1164 | Kaminski MF, Regula J. Colorectal cancer screening by colonoscopy - current issues. Digestion. 2007;76(1):20-25. doi: 10.1159/000108390. | Irrelevant Topic |
| 1165 | Kan WC, Wang HY, Chien CC, Tan CK, Lin CY, Su SB. Intermediate bioelectrolyte changes after phospho-soda or polyethylene glycol precolonoscopic laxatives in a population undergoing health examinations. Nephrol Dial Transplant. 2012;27(2):752-757. doi: 10.1093/ndt/gfr189. | Irrelevant Topic |
| 1166 | Kang X, Zhao L, Zhu Z, Leung F, Wang L, Wang X, et al. Same-day single dose of 2 liter polyethylene glycol is not inferior to the standard bowel preparation regimen in low-risk patients: a randomized, controlled trial. Am J Gastroenterol. 2018;113(4):601-610. doi: 10.1038/ajg.2018.25. | Irrelevant Topic |
| 1167 | Kang XY, Lou LJ, Wang ZY, Chen ZQ, Chen L, Wang XP, et al. Patient-related factors related to prolonged preparation-to-colonoscopy interval and insufficient purgative intake before colonoscopy: a post-hoc observational study. J Dig Dis. 2023;24(2):133-141. doi: 10.1111/1751-2980.13170. | Irrelevant Topic |
| 1168 | Kao D, Lalor E, Sandha G, Fedorak RN, van der Knoop B, Doornweerd S, et al. A randomized controlled trial of four precolonoscopy bowel cleansing regimens. Canadian Journal of Gastroenterology and Hepatology. 2011;25(12):657-662. doi: 10.1155/2011/486084. | Irrelevant Topic |
| 1169 | Karling P, Lundgren D, Eklöf V, Palmqvist R, Hultdin J. Improved monitoring of inflammatory activity in patients with ulcerative colitis by combination of faecal tests for haemoglobin and calprotectin. Scand J Clin Lab Invest. 2019;79(5):341-346. doi: 10.1080/00365513.2019.1622148. | Irrelevant Topic |
| 1170 | Kashin SV, Zavyalov DV, Sidneva AV. The effect of alverine citrate plus simethicone (Meteospazmyl) on effectiveness and tolerability of bowel preparation for colonoscopy. Russian Journal of Evidence-Based Gastroenterology. 2023;12(2):34‐44. doi: 10.17116/dokgastro20231202134. | Irrelevant Topic |
| 1171 | Kashiwagi K, Inoue N, Yoshida T, Bessyo R, Yoneno K, Imaeda H, et al. Polyp detection rate in transverse and sigmoid colon significantly increases with longer withdrawal time during screening colonoscopy. Plos One. 2017;12(3):e0174155. doi: 10.1371/journal.pone.0174155. | Irrelevant Topic |
| 1172 | Katsidzira L, Gangaidzo IT, Mapingure MP, Matenga JA. Retrospective study of colorectal cancer in Zimbabwe: colonoscopic and clinical correlates. World J Gastroenterol. 2015;21(8):2374-2380. doi: 10.3748/wjg.v21.i8.2374. | Irrelevant Topic |
| 1173 | Kazarian ES, Carreira FS, Toribara NW, Denberg TD. Colonoscopy completion in a large safety net health care system. Clin Gastroenterol Hepatol. 2008;6(4):438-442. doi: 10.1016/j.cgh.2007.12.003. | Irrelevant Topic |
| 1174 | Kealey SM, Dodd JD, MacEneaney PM, Gibney RG, Malone DE. Minimal preparation computed tomography instead of barium enema/colonoscopy for suspected colon cancer in frail elderly patients: an outcome analysis study. Clin Radiol. 2004;59(1):44-52. doi: 10.1016/j.crad.2003.08.001. | Irrelevant Topic |
| 1175 | Keppel GA, Ike B, Leroux BG, Ko LK, Osterhage KP, Jacobs JD, et al. Colonoscopy outreach for rural communities (CORC): a study protocol of a pragmatic randomized controlled trial of a patient navigation program to improve colonoscopy completion for colorectal cancer screening. Contemporary Clinical Trials. 2024;141. doi: 10.1016/j.cct.2024.107539. | Irrelevant Topic |
| 1176 | Khamees N, Al-Ani A, Tamimi TA, Sarhan O, Matouq Y, Laswi D, et al. Epidemiology and clinical characteristics of colorectal cancer and advanced adenoma: a single center experience in Jordan. BMC Gastroenterol. 2025;25(1):120. doi: 10.1186/s12876-024-03531-1. | Irrelevant Topic |
| 1177 | Khan S, Ahmed J, Lim M, Owais A, McNaught C, Mainprize K, et al. Colonoscopy in the octogenarian population: diagnostic and survival outcomes from a large series of patients. Surgeon. 2011;9(4):195-199. doi: 10.1016/j.surge.2010.09.003. | Irrelevant Topic |
| 1178 | Kherad O, Restellini S, Martel M, Barkun AN. Polyethylene glycol versus sodium picosulfalte bowel preparation in the setting of a colorectal cancer screening program. Can J Gastroenterol Hepatol. 2015;29(7):384-390. doi: 10.1155/2015/350587. | Irrelevant Topic |
| 1179 | Kiesslich R, Goetz M, Lammersdorf K, Schneider C, Burg J, Stolte M, et al. Chromoscopy-guided endomicroscopy increases the diagnostic yield of intraepithelial neoplasia in ulcerative colitis. Gastroenterology. 2007;132(3):874-882. doi: 10.1053/j.gastro.2007.01.048. | Irrelevant Topic |
| 1180 | Kim B, Kim BC, Kim J, Oh HJ, Ryu KH, Park BJ, et al. Quality of bowel preparation for colonoscopy in patients with a history of abdomino-pelvic surgery: retrospective cohort study. Yonsei Med J. 2019;60(1):73-78. doi: 10.3349/ymj.2019.60.1.73. | Irrelevant Topic |
| 1181 | Kim D, Jung JY, Oh HS, Jee SR, Park SJ, Lee SH, et al. Comparison of sampling methods in assessing the microbiome from patients with ulcerative colitis. BMC Gastroenterol. 2021;21(1):396. doi: 10.1186/s12876-021-01975-3. | Irrelevant Topic |
| 1182 | Kim D, Wang JHY, Gupta A, Wlodarczyk J, Williams B, Cologne K, et al. Patient compliance with bowel preparation for elective colorectal surgery. Journal of Surgical Research. 2024;302:697-705. doi: 10.1016/j.jss.2024.07.058. | Irrelevant Topic |
| 1183 | Kim DB, Lee KM, Kang SG, Jung SH. Factors affecting proximal colon cleansing based on bowel movement kinetics: a prospective observational study. Gastroenterol Res Pract. 2019;2019. doi: 10.1155/2019/7032971. | Irrelevant Topic |
| 1184 | Kim DH, Park Y, Kim B, Kim SW, Park SJ, Hong SP, et al. Fecal calprotectin as a non-invasive biomarker for intestinal involvement of Behcet's disease. J Gastroenterol Hepatol. 2017;32(3):595-601. doi: 10.1111/jgh.13530. | Irrelevant Topic |
| 1185 | Kim DJ, Kim HW, Park SB, Kang DH, Choi CW, Hong JB, et al. Efficacy of cap-assisted colonoscopy according to lesion location and endoscopist training level. World Journal of Gastroenterology. 2015;21(20):6261-6270. doi: 10.3748/wjg.v21.i20.6261. | Irrelevant Topic |
| 1186 | Kim G, Jang EC, Kim YS, Yoon SA, Kim SS, Yoo SJ, et al. Sigmoid volvulus occurring during bowel preparation period before colonoscopy in a hemodialysis patient. Clin Nephrol. 2006;66(2):149-150. doi: 10.5414/cnp66149. | Irrelevant Topic |
| 1187 | Kim HG. Is elderly age a simple predictive factor for inadequate bowel preparation before colonoscopy? Gut Liver. 2016;10(4):489-490. doi: 10.5009/gnl16203. | Irrelevant Topic |
| 1188 | Kim HG, Jeon SR, Lee TH, Ko BM, Kim JO, Lee JS. Rescreening colonoscopy practice after a negative index colonoscopy in a clinical setting; cross-sectional study for interval and outcomes of rescreening colonoscopy. Hepatogastroenterology. 2014;61(136):2266-2271. | Irrelevant Topic |
| 1189 | Kim HH, Park SJ, Park MI, Moon W, Kim SE. Transparent-cap-fitted colonoscopy shows higher performance with cecal intubation time in difficult cases. World Journal of Gastroenterology. 2012;18(16):1953-1958. doi: 10.3748/wjg.v18.i16.1953. | Irrelevant Topic |
| 1190 | Kim HS, Kim TI, Kim WH, Kim YH, Kim HJ, Yang SK, et al. Risk factors for immediate postpolypectomy bleeding of the colon: a multicenter study. Am J Gastroenterol. 2006;101(6):1333-1341. doi: 10.1111/j.1572-0241.2006.00638.x. | Irrelevant Topic |
| 1191 | Kim HS, Park DH, Kim JW, Jee MG, Baik SK, Kwon SO, et al. Effectiveness of walking exercise as a bowel preparation for colonoscopy: a randomized controlled trial. American Journal of Gastroenterology. 2005;100(9):1964‐1969. doi: 10.1111/j.1572-0241.2005.40373.x. | Irrelevant Topic |
| 1192 | Kim HY. Cecal intubation time in screening colonoscopy. Medicine (Baltimore). 2021;100(19):e25927. doi: 10.1097/md.0000000000025927. | Irrelevant Topic |
| 1193 | Kim JH, Kim JH, Chun J, Lee C, Im JP, Kim JS. Early versus late bedside endoscopy for gastrointestinal bleeding in critically ill patients. Korean Journal of Internal Medicine. 2018;33(2):304-312. doi: 10.3904/kjim.2016.182. | Irrelevant Topic |
| 1194 | Kim JS, Kang SH, Moon HS, Lee ES, Kim SH, Sung JK, et al. Impact of bowel preparation quality on adenoma identification during colonoscopy and optimal timing of surveillance. Dig Dis Sci. 2015;60(10):3092-3099. doi: 10.1007/s10620-015-3737-2. | Irrelevant Topic |
| 1195 | Kim JS, Lee KM, Kim SW, Kim EJ, Lim CH, Oh ST, et al. Preoperative colonoscopy through the colonic stent in patients with colorectal cancer obstruction. World J Gastroenterol. 2014;20(30):10570-10576. doi: 10.3748/wjg.v20.i30.10570. | Irrelevant Topic |
| 1196 | Kim JW, Han JH, Boo SJ, Ko OB, Park SK, Park SH, et al. Rescue bowel preparation: same day 2 L polyethylene glycol addition, not superior to bisacodyl addition 7 days later. Dig Dis Sci. 2014;59(9):2215-2221. doi: 10.1007/s10620-014-3125-3. | Irrelevant Topic |
| 1197 | Kim KO, Jeong DE, Hwang MJ, Lee SH, Jang BI, Kim TN. Sa1081 risk factors of inadequate bowel preparation before colonoscopy in outpatients clinics: a single center prospective study. Gastrointest Endosc. 2016;83:AB228-AB228. doi: 10.1016/j.gie.2016.03.304. | Irrelevant Topic |
| 1198 | Kim MH, Moon HS, Kwon IS, Kim JS, Kang SH, Sung JK, et al. The incidence and risk factors of sessile serrated adenomas in left side colon cancer patients after curative surgery. Medicine (Baltimore). 2020;99(29):e20799. doi: 10.1097/md.0000000000020799. | Irrelevant Topic |
| 1199 | Kim MJ, Kim Y, Kim JH, Youn YH, Chun J. Balancing safety and effectiveness in colonoscopy for older adults: a narrative review. Intest Res. 2025;23(4):443-454. doi: 10.5217/ir.2025.00092. | Irrelevant Topic |
| 1200 | Kim S, Choi J, Kim TH, Kong SH, Suh YS, Im JP, et al. Effect of previous gastrectomy on the performance of postoperative colonoscopy. J Gastric Cancer. 2016;16(3):167-176. doi: 10.5230/jgc.2016.16.3.167. | Irrelevant Topic |
| 1201 | Kim S, Park S, Kang Y, Koh H. Combining faecal calprotectin and sigmoidoscopy can predict mucosal healing in paediatric ulcerative colitis. Eur J Gastroenterol Hepatol. 2020;32(1):17-21. doi: 10.1097/meg.0000000000001550. | Irrelevant Topic |
| 1202 | Kim SH, Choi BI, Han JK, Lee JM, Eun HW, Lee JY, et al. CT colonography in a Korean population with a high residue diet: comparison between wet and dry preparations. Clin Radiol. 2006;61(6):483-494. doi: 10.1016/j.crad.2006.01.012. | Irrelevant Topic |
| 1203 | Kim SJ, Lee J, Baek DY, Lee JH, Hong R. Early gastric neoplasms are significant risk factor for colorectal adenoma: a prospective case-control study. Medicine (Baltimore). 2022;101(32):e29956. doi: 10.1097/md.0000000000029956. | Irrelevant Topic |
| 1204 | Kim YH, Seo EH, Lee JS, Lee SH, Park HS, Choi SH, et al. Inadequate bowel cleansing efficacy of split-dose polyethylene glycol for colonoscopy in type 2 diabetic patients: a prospective and blinded study. J Clin Gastroenterol. 2017;51(3):240‐246. doi: 10.1097/MCG.0000000000000536. | Irrelevant Topic |
| 1205 | Kim YS, Kim N, Kim SH, Park MJ, Lim SH, Yim JY, et al. The efficacy of intravenous contrast-enhanced 16-raw multidetector CT colonography for detecting patients with colorectal polyps in an asymptomatic population in Korea. J Clin Gastroenterol. 2008;42(7):791-798. doi: 10.1097/MCG.0b013e31811edcb7. | Irrelevant Topic |
| 1206 | Kimpel CC, Bonnet K, Schlundt D. The patient experience of an inadequate-quality bowel preparation during the colonoscopy process: a qualitative study. Gastroenterology Nursing. 2022;45(4):244-253. doi: 10.1097/SGA.0000000000000654. | Irrelevant Topic |
| 1207 | King-Marshall EC, Mueller N, Dailey A, Barnett TE, George TJ, Jr., Sultan S, et al. "It is just another test they want to do": patient and caregiver understanding of the colonoscopy procedure. Patient Educ Couns. 2016;99(4):651-658. doi: 10.1016/j.pec.2015.10.021. | Irrelevant Topic |
| 1208 | Kitazume Y, Takenaka K, Ohtsuka K, Kimura K, Ogihara Y, Fujioka T, et al. Crohn disease: magnetic resonance enterocolonography features of endoscopic ulcer stages reclassified with the healing process and the relationships to prognoses. Japanese Journal of Radiology. 2021;39(5):459-476. doi: 10.1007/s11604-020-01082-5. | Irrelevant Topic |
| 1209 | Klasko-Foster LB, Keller MM, Kiviniemi MT. Is it disgusting or am I just easily disgusted? The relation between situational disgust, dispositional disgust, and colonoscopy intentions. Eur J Cancer Care (Engl). 2020;29(5):e13244. doi: 10.1111/ecc.13244. | Irrelevant Topic |
| 1210 | Klepp P, Tollisen A, Røseth A, Cvancarova Småstuen M, Andersen SN, Vatn M, et al. Real-life chromoendoscopy for dysplasia surveillance in ulcerative colitis. World J Gastroenterol. 2018;24(35):4069-4076. doi: 10.3748/wjg.v24.i35.4069. | Irrelevant Topic |
| 1211 | Kluge MA, Williams JL, Wu CK, Jacobson BC, Iiischroy PC, Lieberman DA, et al. Inadequate Boston Bowel Preparation Scale scores predict the risk of missed neoplasia on the next colonoscopy. Gastrointest Endosc. 2018;87(3):744-751. doi: 10.1016/j.gie.2017.06.012. | Irrelevant Topic |
| 1212 | Kmochova K, Grega T, Ngo O, Vojtechova G, Majek O, Urbanek P, et al. Comparison of four bowel cleansing agents for colonoscopy and the factors affecting their efficacy. A prospective, randomized study. J Gastrointestin Liver Dis. 2021;30(2):213-220. doi: 10.15403/jgld-3401. | Irrelevant Topic |
| 1213 | Ko CW, Dominitz JA, Green P, Kreuter W, Baldwin LM. Utilization and predictors of early repeat colonoscopy in Medicare beneficiaries. American Journal of Gastroenterology. 2010;105(12):2670-2679. doi: 10.1038/ajg.2010.344. | Irrelevant Topic |
| 1214 | Ko CW, Riffle S, Shapiro JA, Saunders MD, Lee SD, Tung BY, et al. Incidence of minor complications and time lost from normal activities after screening or surveillance colonoscopy. Gastrointest Endosc. 2007;65(4):648-656. doi: 10.1016/j.gie.2006.06.020. | Irrelevant Topic |
| 1215 | Koido S, Ohkusa T, Nakae K, Yokoyama T, Shibuya T, Sakamoto N, et al. Factors associated with incomplete colonoscopy at a Japanese academic hospital. World J Gastroenterol. 2014;20(22):6961-6967. doi: 10.3748/wjg.v20.i22.6961. | Irrelevant Topic |
| 1216 | Kojecky V, Matous J, Keil R, Dastych M, Zadorova Z, Varga M, et al. The optimal bowel preparation intervals before colonoscopy: a randomized study comparing polyethylene glycol and low-volume solutions. Digestive and Liver Disease. 2018;50(3):271‐276. doi: 10.1016/j.dld.2017.10.010. | Irrelevant Topic |
| 1217 | Köklü S, Yüksel O, Onur I, Unverdi S, Biyikoğlu I, Akbal E, et al. Ileocolonic involvement in Behçet's disease: endoscopic and histological evaluation. Digestion. 2010;81(4):214-217. doi: 10.1159/000264643. | Irrelevant Topic |
| 1218 | Komeda Y, Suzuki N, Sarah M, Thomas-Gibson S, Vance M, Fraser C, et al. Factors associated with failed polyp retrieval at screening colonoscopy. Gastrointest Endosc. 2013;77(3):395-400. doi: 10.1016/j.gie.2012.10.007. | Irrelevant Topic |
| 1219 | Komori S, Akiyama J, Tatsuno N, Yamada E, Izumi A, Hamada M, et al. Prevalence and risk factors of constipation symptoms among patients undergoing colonoscopy: a single-center cross-sectional study. Digestion. 2024;105(4):299-309. doi: 10.1159/000539366. | Irrelevant Topic |
| 1220 | Korsten MA, Spungen AM, Rosman AR, Ancha HR, Post JB, Shaw S, et al. A prospective assessment of renal impairment after preparation for colonoscopy: oral sodium phosphate appears to be safe in well-hydrated subjects with normal renal status. Dig Dis Sci. 2010;55(7):2021-2029. doi: 10.1007/s10620-009-1013-z. | Irrelevant Topic |
| 1221 | Kothari ST, Huang RJ, Shaukat A, Agrawal D, Buxbaum JL, Fehmi SMA, et al. ASGE review of adverse events in colonoscopy. Gastrointest Endosc. 2019;90(6):863-+. doi: 10.1016/j.gie.2019.07.033. | Irrelevant Topic |
| 1222 | Krishnan P, Sofi AA, Dempsey R, Alaradi O, Nawras A. Body mass index predicts cecal insertion time: the higher, the better. Dig Endosc. 2012;24(6):439-442. doi: 10.1111/j.1443-1661.2012.01296.x. | Irrelevant Topic |
| 1223 | Kumar A, Bae E, Osemi E, Fayemiwo O, Shenoy V, Singh P, et al. Endoscopic disease activity and biologic therapy are independent predictors of suboptimal bowel preparation in patients with inflammatory bowel disease undergoing colonoscopy: a multicenter analysis. American Journal of Gastroenterology. 2022;117(10):S509-S510. | Irrelevant Topic |
| 1224 | Kumar A, Lin L, Bernheim O, Bagiella E, Jandorf L, Itzkowitz SH, et al. Effect of functional status on the quality of bowel preparation in elderly patients undergoing screening and surveillance colonoscopy. Gut Liver. 2016;10(4):569-573. doi: 10.5009/gnl15230. | Irrelevant Topic |
| 1225 | Kumar S, Bennett WE, Bozic MA, Croffie JM, Ferrell E, Hon EC, et al. Inadequate bowel preparation in pediatric colonoscopy-prospective study of potential causes. Journal of Pediatric Gastroenterology and Nutrition. 2021;73(3):325-328. doi: 10.1097/MPG.0000000000003178. | Irrelevant Topic |
| 1226 | Kunnackal John G, Thuluvath AJ, Carrier H, Ahuja NK, Gupta E, Stein E. Poor health literacy and medication burden are significant predictors for inadequate bowel preparation in an urban tertiary care setting. J Clin Gastroenterol. 2019;53(9):e382-e386. doi: 10.1097/mcg.0000000000001177. | Irrelevant Topic |
| 1227 | Kunz L, Gillespie D. A comparison of bowel preparations for colonoscopy in constipated adults. Gastroenterol Nurs. 2017;40(5):364-372. doi: 10.1097/sga.0000000000000257. | Irrelevant Topic |
| 1228 | Kurlander JE, Sultan S, Saini SD. The clinical and economic effects of targeted bowel preparation: results of a decision analysis. J Clin Gastroenterol. 2018;52(10):853-858. doi: 10.1097/mcg.0000000000000845. | Irrelevant Topic |
| 1229 | Kushnir VM, Bhat P, Chokshi RV, Lee A, Borg BB, Gyawali CP, et al. The impact of opiate pain medications and psychoactive drugs on the quality of colon preparation in outpatient colonoscopy. Digestive and Liver Disease. 2014;46(1):56-61. doi: 10.1016/j.dld.2013.07.020. | Irrelevant Topic |
| 1230 | Kutyla MJ, O'Connor S, Hourigan LF, Kendall B, Whaley A, Meeusen V, et al. An evidence-based approach towards targeted patient education to improve bowel preparation for colonoscopy. J Clin Gastroenterol. 2020;54(8):707-713. doi: 10.1097/mcg.0000000000001286. | Irrelevant Topic |
| 1231 | Kwon SH, Choo JW, Kim HG, Jeon SR, Lee BH, Lee TH, et al. Advanced neoplasm detection and its associated factors in colonoscopic surveillance of endoscopically resected early colorectal cancer. Korean J Gastroenterol. 2013;62(4):219-226. doi: 10.4166/kjg.2013.62.4.219. | Irrelevant Topic |
| 1232 | Labenz J, Borkenstein DP, Heil FJ, Madisch A, Tappe U, Schmidt H, et al. Application of a multispecies probiotic reduces gastro-intestinal discomfort and induces microbial changes after colonoscopy. Front Oncol. 2023;12. doi: 10.3389/fonc.2022.1078315. | Irrelevant Topic |
| 1233 | Ladas SD, Karamanolis G, Ben-Soussan E. Colonic gas explosion during therapeutic colonoscopy with electrocautery. World J Gastroenterol. 2007;13(40):5295-5298. doi: 10.3748/wjg.v13.i40.5295. | Irrelevant Topic |
| 1234 | Lagares-Garcia JA, Kurek S, Collier B, Diaz F, Schilli R, Richey J, et al. Colonoscopy in octogenarians and older patients. Surg Endosc. 2001;15(3):262-265. doi: 10.1007/s004640000339. | Irrelevant Topic |
| 1235 | Laghi A. Computed tomography colonography in 2014: an update on technique and indications. World J Gastroenterol. 2014;20(45):16858-16867. doi: 10.3748/wjg.v20.i45.16858. | Irrelevant Topic |
| 1236 | Laique SN, Hayat U, Sarvepalli S, Vaughn B, Ibrahim M, McMichael J, et al. Application of optical character recognition with natural language processing for large-scale quality metric data extraction in colonoscopy reports. Gastrointest Endosc. 2021;93(3):750-757. doi: 10.1016/j.gie.2020.08.038. | Irrelevant Topic |
| 1237 | Laiyemo AO, Burnside C, Laiyemo MA, Kwagyan J, Williams CD, Idowu KA, et al. Beverage intake preference and bowel preparation laxative taste preference for colonoscopy. World J Gastrointest Pharmacol Ther. 2015;6(3):84-88. doi: 10.4292/wjgpt.v6.i3.84. | Irrelevant Topic |
| 1238 | Laiyemo AO, Doubeni C, Pinsky PF, Doria-Rose VP, Sanderson AK, 2nd, Bresalier R, et al. Factors associated with inadequate colorectal cancer screening with flexible sigmoidoscopy. Cancer Epidemiol. 2012;36(4):395-399. doi: 10.1016/j.canep.2011.10.013. | Irrelevant Topic |
| 1239 | Laiyemo AO, Kwagyan J, Williams CD, Rogers J, Kibreab A, Jack MA, et al. Using patients' social network to improve compliance to outpatient screening colonoscopy appointments among blacks: a randomized clinical trial. American Journal of Gastroenterology. 2019;114(10):1671‐1677. doi: 10.14309/ajg.0000000000000387. | Irrelevant Topic |
| 1240 | Lam AY, Li Y, Gregory DL, Prinz J, O'Reilly J, Manka M, et al. Association between improved adenoma detection rates and interval colorectal cancer rates after a quality improvement program. Gastrointest Endosc. 2020;92(2):355-+. doi: 10.1016/j.gie.2020.02.016. | Irrelevant Topic |
| 1241 | Lam TYT, Hui AJ, Sia F, Wong MY, Lee CCP, Chung KW, et al. Short message service reminders reduce outpatient colonoscopy nonattendance rate: a randomized controlled study. J Gastroenterol Hepatol. 2021;36(4):1044-1050. doi: 10.1111/jgh.15218. | Irrelevant Topic |
| 1242 | Lam TYT, Wu PI, Tang RSY, Tse YK, Lau JYW, Wu JCY, et al. Nurse-led reinforced education by mobile messenger improves the quality of bowel preparation of colonoscopy in a population-based colorectal cancer screening program: a randomized controlled trial. Int J Nurs Stud. 2022;133:104301. doi: 10.1016/j.ijnurstu.2022.104301. | Irrelevant Topic |
| 1243 | Lanas Á, Balaguer F, Sánchez-Luengo M, Hijos-Mallada G, Hernández-Mesa G, Piñero M, et al. Fecal occult blood and calprotectin testing to prioritize primary care patients for colonoscopy referral: the advantage study. United European Gastroenterol J. 2023;11(7):692-699. doi: 10.1002/ueg2.12446. | Irrelevant Topic |
| 1244 | Lau LHS, Ho JCL, Lai JCT, Ho AHY, Wu CWK, Lo VWH, et al. Effect of real-time computer-aided polyp detection system (ENDO-AID) on adenoma detection in endoscopists-in-training: a randomized trial. Clinical Gastroenterology and Hepatology. 2024;22(3):630‐641.e634. doi: 10.1016/j.cgh.2023.10.019. | Irrelevant Topic |
| 1245 | Layton JB, Klemmer PJ, Christiansen CF, Bomback AS, Baron JA, Sandler RS, et al. Sodium phosphate does not increase risk for acute kidney injury after routine colonoscopy, compared with polyethylene glycol. Clin Gastroenterol Hepatol. 2014;12(9):1514-1521.e1513. doi: 10.1016/j.cgh.2014.01.034. | Irrelevant Topic |
| 1246 | Lazzaroni M, Bianchi Porro G. Preparation, premedication, and surveillance. Endoscopy. 2001;33(2):103-108. doi: 10.1055/s-2001-11665. | Irrelevant Topic |
| 1247 | Lebwohl B, Wang TC, Neugut AI. Socioeconomic and other predictors of colonoscopy preparation quality. Dig Dis Sci. 2010;55(7):2014-2020. doi: 10.1007/s10620-009-1079-7. | Irrelevant Topic |
| 1248 | Lee D. E/e' elevation as a predictor of post-colonoscopy left ventricular dysfunction in patients underwent sodium phosphate (NaP) bowel preparation for colonoscopy. European heart journal. 2017;38:1101-1101. | Irrelevant Topic |
| 1249 | Lee DW, Koo JS, Kang S, Kim SY, Hyun JJ, Jung SW, et al. Association between bowel habits and quality of bowel preparation for colonoscopy. Medicine (Baltimore). 2017;96(29):e7319. doi: 10.1097/md.0000000000007319. | Irrelevant Topic |
| 1250 | Lee E, Shafer LA, Walker JR, Waldman C, Michaud V, Yang CY, et al. Information experiences, needs, and preferences of colonoscopy patients: a pre-colonoscopy survey. Medicine. 2019;98(20). doi: 10.1097/MD.0000000000015738. | Irrelevant Topic |
| 1251 | Lee HH, Oh JS, Park JM, Chun HJ, Kim TH, Cheung DY, et al. Transcatheter embolization effectively controls acute lower gastrointestinal bleeding without localizing bleeding site prior to angiography. Scand J Gastroenterol. 2018;53(9):1089-1096. doi: 10.1080/00365521.2018.1501512. | Irrelevant Topic |
| 1252 | Lee J, Jeong SJ, Kim TH, Park YE, Choi JH, Heo NY, et al. Efficacy of mosapride citrate with a split dose of polyethylene glycol plus ascorbic acid for bowel preparation in elderly patients: a randomized controlled trial. Medicine (Madr). 2020;99(2):e18702. doi: 10.1097/MD.0000000000018702. | Irrelevant Topic |
| 1253 | Lee J, Wallace MB. State of the art: the impact of artificial intelligence in endoscopy 2020. Curr Gastroenterol Rep. 2021;23(5):7. doi: 10.1007/s11894-021-00810-9. | Irrelevant Topic |
| 1254 | Lee JK, Jensen CD, Levin TR, Zauber AG, Doubeni CA, Zhao WK, et al. Accurate identification of colonoscopy quality and polyp findings using natural language processing. J Clin Gastroenterol. 2019;53(1):e25-e30. doi: 10.1097/mcg.0000000000000929. | Irrelevant Topic |
| 1255 | Lee JW, Woo D, Kim KO, Kim ES, Kim SK, Lee HS, et al. Deep learning model using stool pictures for predicting endoscopic mucosal inflammation in patients with ulcerative colitis. American Journal of Gastroenterology. 2025;120(1):213-224. doi: 10.14309/ajg.0000000000002978. | Irrelevant Topic |
| 1256 | Lee KJ, Park HJ, Kim HS, Baik KH, Kim YS, Park SC, et al. Electrolyte changes after bowel preparation for colonoscopy: a randomized controlled multicenter trial. World Journal of Gastroenterology. 2015;21(10):3041‐3048. doi: 10.3748/wjg.v21.i10.3041. | Irrelevant Topic |
| 1257 | Lee SK, Kim TI, Shin SJ, Kim BC, Kim WH. Impact of prior abdominal or pelvic surgery on colonoscopy outcomes. J Clin Gastroenterol. 2006;40(8):711-716. doi: 10.1097/00004836-200609000-00010. | Irrelevant Topic |
| 1258 | Lee SP, Park E, Kim HV, Sung IK, Kim JH, Lee SY, et al. Does 2 L polyethylene glycol plus ascorbic acid increase the risk of renal impairment compared to 4 L polyethylene glycol? Dig Dis Sci. 2016;61(11):3207-3214. doi: 10.1007/s10620-016-4297-9. | Irrelevant Topic |
| 1259 | Lee SZ, Bryant R, Lidums I, Shin S, Nind G, Goodsall T. Longer runway time predicts failure of inpatient bowel preparation for elective colonoscopy. J Gastroenterol Hepatol. 2020;35:236-236. | Irrelevant Topic |
| 1260 | Lee TJW, Rees CJ, Blanks RG, Moss SM, Nickerson C, Wright KC, et al. Colonoscopic factors associated with adenoma detection in a national colorectal cancer screening program. Endoscopy. 2014;46(3):203-211. doi: 10.1055/s-0033-1358831. | Irrelevant Topic |
| 1261 | Lee YJ, Kim ES, Park KS, Cho KB, Jang BK, Chung WJ, et al. Education for ward nurses influences the quality of inpatient's bowel preparation for colonoscopy. Medicine. 2015;94(34). doi: 10.1097/MD.0000000000001423. | Irrelevant Topic |
| 1262 | Lefere PA, Gryspeerdt SS, Dewyspelaere J, Baekelandt M, Van Holsbeeck BG. Dietary fecal tagging as a cleansing method before CT colonography: initial results polyp detection and patient acceptance. Radiology. 2002;224(2):393‐403. doi: 10.1148/radiol.2241011222. | Irrelevant Topic |
| 1263 | Lei, II, Parisi I, Bhandare A, Perez FP, Lee T, Shehkar C, et al. Factors predicting conversion from colon capsule endoscopy to conventional optical endoscopy-findings from the CESCAIL study. BMC Gastroenterol. 2025;25(1):363. doi: 10.1186/s12876-025-03828-9. | Irrelevant Topic |
| 1264 | Leung FW, Aljebreen AM. Unsedated colonoscopy: is it feasible? Saudi J Gastroenterol. 2011;17(4):289-292. doi: 10.4103/1319-3767.82588. | Irrelevant Topic |
| 1265 | Leung JW, Mann SK, Siao-Salera R, Ransibrahmanakul K, Lim B, Cabrera H, et al. A randomized, controlled comparison of warm water infusion in lieu of air insufflation versus air insufflation for aiding colonoscopy insertion in sedated patients undergoing colorectal cancer screening and surveillance. Gastrointest Endosc. 2009;70(3):505-510. doi: 10.1016/j.gie.2008.12.253. | Irrelevant Topic |
| 1266 | Lhewa DY, Strate LL. Pros and cons of colonoscopy in management of acute lower gastrointestinal bleeding. World Journal of Gastroenterology. 2012;18(11):1185-1190. doi: 10.3748/wjg.v18.i11.1185. | Irrelevant Topic |
| 1267 | Li JH, Ma XY, Yi Y, Li LR, Xu ZY, Chang Y. Association between serum ferritin levels and metabolic-associated fatty liver disease in adults: a cross-sectional study based on the NHANES. Current Medical Science. 2024;44(3):494-502. doi: 10.1007/s11596-024-2868-0. | Irrelevant Topic |
| 1268 | Li JW, Wu CCH, Lee JWJ, Liang RY, Soon GST, Wang LM, et al. Real-world validation of a computer-aided diagnosis system for prediction of polyp histology in colonoscopy: a prospective multicenter study. American Journal of Gastroenterology. 2023;118(8):1353-1364. doi: 10.14309/ajg.0000000000002282. | Irrelevant Topic |
| 1269 | Li Y, Khamou M, Schaarschmidt BM, Umutlu L, Forsting M, Demircioglu A, et al. Comparison of 18F-FDG PET-MR and fecal biomarkers in the assessment of disease activity in patients with ulcerative colitis. British Journal of Radiology. 2020;93(1112):20200167. doi: 10.1259/bjr.20200167. | Irrelevant Topic |
| 1270 | Li YL, Guan X, Dou LZ, Liu Y, Huang HY, Huang SK, et al. [The clinical value of multi-target stool fecal immunochemical test-DNA in early screening and diagnosis for colorectal cancer]. Zhonghua Yi Xue Za Zhi. 2022;102(33):2607-2613. doi: 10.3760/cma.j.cn112137-20220430-00974. | Irrelevant Topic |
| 1271 | Li YY, Jia XY, Liu BZ, Qi YM, Zhang XB, Ji R, et al. Randomized controlled trial: standard versus supplemental bowel preparation in patients with Bristol stool form 1 and 2. Plos One. 2017;12(2). doi: 10.1371/journal.pone.0171563. | Irrelevant Topic |
| 1272 | Liang CM, Chiu YC, Wu KL, Tam W, Tai WC, Hu ML, et al. Impact factors for difficult cecal intubation during colonoscopy. Surg Laparosc Endosc Percutan Tech. 2012;22(5):443-446. doi: 10.1097/SLE.0b013e3182611c69. | Irrelevant Topic |
| 1273 | Liang JQ, Zeng Y, Kwok G, Cheung CP, Suen BY, Ching JYL, et al. Novel microbiome signatures for non-invasive diagnosis of adenoma recurrence after colonoscopic polypectomy. Alimentary pharmacology & therapeutics. 2022;55(7):847‐855. doi: 10.1111/apt.16799. | Irrelevant Topic |
| 1274 | Liang Y, Xin W, Yang Y, Li X. Application of care bundles in bowel preparation for colonoscopy in children. Ann Palliat Med. 2019;8(4):476-482. doi: 10.21037/apm.2019.09.01. | Irrelevant Topic |
| 1275 | Liao FQ, Huang YF, Lai YK, Xie JF. The status quo of short videos as a source of health information regarding bowel preparation before colonoscopy. Frontiers in public health. 2024;12. doi: 10.3389/fpubh.2024.1309632. | Irrelevant Topic |
| 1276 | Liao Y, Li S, Chen H, Chen C, Huang J, Lin F, et al. A validated tool of combining risk factors with fecal immunochemical test for selecting colonoscopy in asymptomatic Chinese population. European Journal of Cancer Prevention. 2023;32(3):275-280. doi: 10.1097/CEJ.0000000000000622. | Irrelevant Topic |
| 1277 | Lieberman DA, Holub JL, Morris CD, Logan J, Williams JL, Carney P. Low rate of large polyps (>9 mm) within 10 years after an adequate baseline colonoscopy with no polyps. Gastroenterology. 2014;147(2):343-350. doi: 10.1053/j.gastro.2014.04.020. | Irrelevant Topic |
| 1278 | Liedenbaum MH, van Rijn AF, de Vries AH, Dekker HM, Thomeer M, van Marrewijk CJ, et al. Using CT colonography as a triage technique after a positive faecal occult blood test in colorectal cancer screening. Gut. 2009;58(9):1242-1249. doi: 10.1136/gut.2009.176867. | Irrelevant Topic |
| 1279 | Lien YH. Is bowel preparation before colonoscopy a risky business for the kidney? Nat Clin Pract Nephrol. 2008;4(11):606-614. doi: 10.1038/ncpneph0939. | Irrelevant Topic |
| 1280 | Lim JH, Lee WY, Yun SH, Kim HC, Cho YB, Huh JW, et al. Comparison of oncologic outcomes between incomplete obstructive colon cancer and non-obstructive colon cancer by tumor location. Front Oncol. 2022;12. doi: 10.3389/fonc.2022.914299. | Irrelevant Topic |
| 1281 | Lim SW, Seo YW, Sinn DH, Kim JY, Chang DK, Kim JJ, et al. Impact of previous gastric or colonic resection on polyethylene glycol bowel preparation for colonoscopy. Surg Endosc. 2012;26(6):1554-1559. doi: 10.1007/s00464-011-2068-4. | Irrelevant Topic |
| 1282 | Lim TZ, Lau J, Wong GJ, Tan LYT, Chang YJ, Natarajan K, et al. Factors predicting improved compliance towards colonoscopy in individuals with positive faecal immunochemical test (FIT). Cancer Medicine. 2021;10(21):7735-7746. doi: 10.1002/cam4.4275. | Irrelevant Topic |
| 1283 | Lin JS, Perdue LA, Henrikson NB, Bean SI, Blasi PR. Screening for colorectal cancer: updated evidence report and systematic review for the US Preventive Services Task Force. JAMA-Journal of the American Medical Association. 2021;325(19):1978-1997. doi: 10.1001/jama.2021.4417. | Irrelevant Topic |
| 1284 | Lin YC, Ko HJ, Yu LY, Chen MJ, Wang HY, Shih SC, et al. Interaction of colorectal neoplasm risk factors and association with metabolic health status focusing on normal waist-to-hip ratio in adults. Cancers (Basel). 2024;16(9). doi: 10.3390/cancers16091617. | Irrelevant Topic |
| 1285 | Liu A, Yan S, Wang H, Lin Y, Wu J, Fu L, et al. Ward nurses-focused educational intervention improves the quality of bowel preparation in inpatients undergoing colonoscopy: a CONSORT-compliant randomized controlled trial. Medicine (Madr). 2020;99(36):e20976. doi: 10.1097/MD.0000000000020976. | Irrelevant Topic |
| 1286 | Liu C, Song X, Hao H. Educational video followed by retelling bowel preparation process to improve colonoscopy bowel preparation quality: a prospective nursing intervention study. Med Sci Monit. 2018;24:6029-6037. doi: 10.12659/msm.909572. | Irrelevant Topic |
| 1287 | Liu FX, Wang L, Yan WJ, Zou LC, Cao YA, Lin XC. Cleansing efficacy and safety of bowel preparation protocol using sodium picosulfate/magnesium citrate considering subjective experiences: an observational study. World J Clin Cases. 2021;9(15). doi: 10.12998/wjcc.v9.i15.3586. | Irrelevant Topic |
| 1288 | Livovsky DM, Horesh A, Koslowsky B, Milgrom YM, Goldin E, Wengrower D. Risk factors for low quality bowel cleansing in hospitalized and ambulatory patients undergoing colonoscopy, a large retrospective study. Gastrointest Endosc. 2017;85(5):AB263-AB264. doi: 10.1016/j.gie.2017.03.596. | Irrelevant Topic |
| 1289 | Livovsky DM, Horesh A, Koslowsky B, Milgrom YM, Goldin E, Wengrower D. Sa1745 risk factors for low quality bowel cleansing in hospitalized and ambulatory patients undergoing colonoscopy, a large retrospective study... Digestive Disease Week (DDW) 2017 American Society for Gastrointestinal Endoscopy (ASGE) Program and Abstracts, Chicago, Illinois, 6–9 May 2017. Gastrointest Endosc. 2017;85:AB263-AB264. doi: 10.1016/j.gie.2017.03.596. | Irrelevant Topic |
| 1290 | Lobatón T, López-García A, Rodríguez-Moranta F, Ruiz A, Rodríguez L, Guardiola J. A new rapid test for fecal calprotectin predicts endoscopic remission and postoperative recurrence in Crohn's disease. Journal of Crohn's and Colitis. 2013;7(12):e641-e651. doi: 10.1016/j.crohns.2013.05.005. | Irrelevant Topic |
| 1291 | Lopes S, Andrade P, Rodrigues-Pinto E, Afonso J, Macedo G, Magro F. Fecal marker levels as predictors of need for endoscopic balloon dilation in Crohn's disease patients with anastomotic strictures. World J Gastroenterol. 2017;23(35):6482-6490. doi: 10.3748/wjg.v23.i35.6482. | Irrelevant Topic |
| 1292 | López-Kostner F, Zárate AJ, Ponce A, Kronberg U, Kawachi H, Okada T, et al. [Results of a multicentric colorectal cancer screening program in Chile]. Rev Med Chil. 2018;146(6):685-692. doi: 10.4067/s0034-98872018000600685. | Irrelevant Topic |
| 1293 | Loraine A. Bowel preparation agent inducing profound shock precolonoscopy. BMJ Case Rep. 2020;13(3). doi: 10.1136/bcr-2019-233406. | Irrelevant Topic |
| 1294 | Lorenzo-Zúñiga V, Sábado F, Pantaleón MA, Machlab S, Carral D, Gómez-Rodríguez BJ, et al. Effectiveness and safety of a 1-L polyethylene glycol and ascorbic acid preparation for colonoscopy in routine clinical practice in Spain. Revista Espanola De Enfermedades Digestivas. 2024;116(11):599-605. doi: 10.17235/reed.2024.10426/2024. | Irrelevant Topic |
| 1295 | Lu KC. Optimizing bowel preparation for colonoscopy: what are the predictors of an inadequate preparation? Discussion. American Journal of Surgery. 2015;209(5):792-792. | Irrelevant Topic |
| 1296 | Lu X, Xie L, Zhao W, Zhang C, Luo X, Zhou Y. Prediction of hypoglycemia in diabetic patients during colonoscopy preparation. Experimental and Clinical Endocrinology and Diabetes. 2022;131(5):274-281. doi: 10.1055/a-2044-0685. | Irrelevant Topic |
| 1297 | Lu XH, Xie LQ, Zhao WE, Zhang CB, Luo XX, Zhou Y. Prediction of hypoglycemia in diabetic patients during colonoscopy preparation. Experimental and Clinical Endocrinology & Diabetes. 2023;131(05):274-281. doi: 10.1055/a-2044-0685. | Irrelevant Topic |
| 1298 | Lu Y, Zhou X, Chen H, Ding C, Si X. Establishment of a model for predicting delayed post-polypectomy bleeding: a real-world retrospective study. Front Med (Lausanne). 2022;9. doi: 10.3389/fmed.2022.1035646. | Irrelevant Topic |
| 1299 | Ma GT, Fang XJ. The safety and effects of high- and low-volume polyethylene glycol bowel preparation methods before colonoscopy on bowel cleanliness: a systematic review and meta-analysis. J Gastrointest Oncol. 2023;14(4):1759-1769. doi: 10.21037/jgo-23-581. | Irrelevant Topic |
| 1300 | Machlab S, Francia E, Mascort J, García-Iglesias P, Mendive JM, Riba F, et al. Risks, indications and technical aspects of colonoscopy in elderly or frail patients. Position paper of the Societat Catalana de Digestologia, the Societat Catalana de Geriatria i Gerontologia and the Societat Catalana de Medicina de Familia i Comunitaria. Gastroenterol Hepatol. 2024;47(1):107-117. doi: 10.1016/j.gastrohep.2023.05.004. | Irrelevant Topic |
| 1301 | MacPhail ME, Hardacker KA, Tiwari A, Vemulapalli KC, Rex DK. Intraprocedural cleansing work during colonoscopy and achievable rates of adequate preparation in an open-access endoscopy unit. Gastrointest Endosc. 2015;81(3):525-530. doi: 10.1016/j.gie.2014.05.002. | Irrelevant Topic |
| 1302 | Madhoun MF, Chaudrey KK, Chisholm SS, Ahmed A, Frost B, Tierney WM. Efficacy and tolerability of various bowel preparations in diabetic patients: a randomized controlled trial. Endosc Int Open. 2018;6(10):E1157‐E1163. doi: 10.1055/a-0650-3908. | Irrelevant Topic |
| 1303 | Mahadev S, Green PHR, Lebwohl B. Rates of suboptimal preparation for colonoscopy differ markedly between providers: impact on adenoma detection rates. J Clin Gastroenterol. 2015;49(9):746-750. doi: 10.1097/MCG.0000000000000210. | Irrelevant Topic |
| 1304 | Maida M, Sinagra E, Morreale GC, Sferrazza S, Scalisi G, Schillaci D, et al. Effectiveness of very low-volume preparation for colonoscopy: a prospective, multicenter observational study. World Journal of Gastroenterology. 2020;26(16):1950‐1961. doi: 10.3748/wjg.v26.i16.1950. | Irrelevant Topic |
| 1305 | Maida M, Vassallo R, Vitello A, Zullo A, Venezia L, Facciorusso A. Impact of bowel cleansing on polyp and adenoma detection rate: post-hoc analysis of a randomized clinical trial. Cancers (Basel). 2025;17(9). doi: 10.3390/cancers17091421. | Irrelevant Topic |
| 1306 | Maida M, Vitello A, Zullo A, Ramai D, Facciorusso A, Vassallo R. Gender differences in quality of bowel preparation for colonoscopy: post hoc analysis of a randomized controlled trial. J Clin Gastroenterol. 2025;59(4):344-349. doi: 10.1097/mcg.0000000000002024. | Irrelevant Topic |
| 1307 | Maione F, Manigrasso M, Milone M, Gennarelli N, Maione R, Cantore G, et al. Could atmospheric temperature impact on adequate colon cleansing for colonoscopy? An observational, single-institution study. Int J Colorectal Dis. 2023;38(1):96. doi: 10.1007/s00384-023-04393-x. | Irrelevant Topic |
| 1308 | Maisonneuve P, Lowenfels AB. Cancer in cystic fibrosis: a narrative review of prevalence, risk factors, screening, and treatment challenges: adult cystic fibrosis series. Chest. 2022;161(2):356-364. doi: 10.1016/j.chest.2021.09.003. | Irrelevant Topic |
| 1309 | Malhotra A, Shah N, Depasquale J, Baddoura W, Spira R, Rector T. Use of Bristol Stool Form Scale to predict the adequacy of bowel preparation - a prospective study. Colorectal Dis. 2016;18(2):200-204. doi: 10.1111/codi.13084. | Irrelevant Topic |
| 1310 | Mandaliya R, Baig K, Barnhill M, Murugesan V, Som A, Mohammed U, et al. Significant variation in the detection rates of proximal serrated polyps among academic gastroenterologists, community gastroenterologists, and colorectal surgeons in a single tertiary care center. Dig Dis Sci. 2019;64(9):2614-2621. doi: 10.1007/s10620-019-05664-w. | Irrelevant Topic |
| 1311 | Manes G, Amato A, Arena M, Pallotta S, Radaelli F, Masci E. Efficacy and acceptability of sodium picosulphate/magnesium citrate vs low-volume polyethylene glycol plus ascorbic acid for colon cleansing: a randomized controlled trial. Colorectal Dis. 2013;15(9):1145-1153. doi: 10.1111/codi.12246. | Irrelevant Topic |
| 1312 | Manes G, Fontana P, Morganti D, De Nucci G, Ardizzone S. Colon cleansing for colonoscopy in patients with IBD colitis: efficacy and acceptability of 2 liter PEG plus bisacodyl vs 4 liter PEG. Digestive and Liver Disease. 2015;47:e76. | Irrelevant Topic |
| 1313 | Manes G, Repici A, Hassan C. Randomized controlled trial comparing efficacy and acceptability of split- and standard dose sodium picosulfate plus magnesium citrate for bowel cleansing prior to colonoscopy. Endoscopy. 2014;46(8):662-669. doi: 10.1055/s-0034-1365800. | Irrelevant Topic |
| 1314 | Mangira D, Ket S, Dwyer J, Secomb R, Reynolds J, Brown G. Augmentation with pre-emptive macrogol-based osmotic laxative does not significantly improve standard bowel preparation in unselected patients: a randomized trial. JGH Open. 2019;3(5):374‐380. doi: 10.1002/jgh3.12170. | Irrelevant Topic |
| 1315 | Mangla S, Liao CH, Reddy KG, Hanan IM, Kupfer S. Predictors of inadequate bowel preparation in a diverse population undergoing colonoscopy. Gastroenterology. 2010;138(5):S191-S191. | Irrelevant Topic |
| 1316 | Mankaney GN, Ando M, Dahdal DN, Burke CA. Safety and efficacy of sodium picosulfate, magnesium oxide, and citric acid bowel preparation in patients with baseline renal impairment or diabetes: subanalysis of a randomized, controlled trial. Therap Adv Gastroenterol. 2021;14:17562848211024458. doi: 10.1177/17562848211024458. | Irrelevant Topic |
| 1317 | Manta R, Tremolaterra F, Arezzo A, Verra M, Galloro G, Dioscoridi L, et al. Complications during colonoscopy: prevention, diagnosis, and management. Techniques in Coloproctology. 2015;19(9):505-513. doi: 10.1007/s10151-015-1344-z. | Irrelevant Topic |
| 1318 | Maralcan G, Başkonuş I, Aybasti N, Gökalp A. The use of fibrin glue in the treatment of fistula-in-ano: a prospective study. Surg Today. 2006;36(2):166-170. doi: 10.1007/s00595-005-3121-5. | Irrelevant Topic |
| 1319 | Marchildon M, Jackson J, Rankin J. Factors influencing inpatient bowel preparation: a scoping review. J Gastroenterol Hepatol. 2024;39(12):2487-2503. doi: 10.1111/jgh.16721. | Irrelevant Topic |
| 1320 | Marciani L, Garsed KC, Hoad CL, Fields A, Fordham I, Pritchard SE, et al. Stimulation of colonic motility by oral PEG electrolyte bowel preparation assessed by MRI: comparison of split vs single dose. Neurogastroenterology and Motility. 2014;26(10):1426-1436. doi: 10.1111/nmo.12403. | Irrelevant Topic |
| 1321 | Marín Gabriel JC, Rodríguez Muñoz S, de la Cruz Bértolo J, Carretero Gómez JF, Muñoz Yagüe MT, Manzano Alonso ML, et al. Electrolytic disturbances and colonoscopy: bowel lavage solutions, age and procedure. Rev Esp Enferm Dig. 2003;95(12):863-875. | Irrelevant Topic |
| 1322 | Markowitz GS, Nasr SH, Klein P, Anderson H, Stack JI, Alterman L, et al. Renal failure due to acute nephrocalcinosis following oral sodium phosphate bowel cleansing. Hum Pathol. 2004;35(6):675-684. doi: 10.1016/j.humpath.2003.12.005. | Irrelevant Topic |
| 1323 | Marmo R, Rotondano G, Riccio G, Marone A, Bianco MA, Stroppa I, et al. Effective bowel cleansing before colonoscopy: a randomized study of split-dosage versus non-split dosage regimens of high-volume versus low-volume polyethylene glycol solutions. Gastrointest Endosc. 2010;72(2):313‐320. doi: 10.1016/j.gie.2010.02.048. | Irrelevant Topic |
| 1324 | McMahon PM, Gazelle GS. Colorectal cancer screening issues: a role for CT colonography? Abdom Imaging. 2002;27(3):235-243. doi: 10.1107/s00261-001-0164-y. | Irrelevant Topic |
| 1325 | McNabb-Baltar J, Dorreen A, Al Dhahab H, Fein M, Xiong X, M OB, et al. Age is the only predictor of poor bowel preparation in the hospitalized patient. Can J Gastroenterol Hepatol. 2016;2016:2139264. doi: 10.1155/2016/2139264. | Irrelevant Topic |
| 1326 | Megna B, Weiss J, Ley D, Saha S, Pfau P, Grimes I, et al. Clear liquid diet before bowel preparation predicts successful chromoendoscopy in patients with inflammatory bowel disease. Gastrointest Endosc. 2019;89(2):373-+. doi: 10.1016/j.gie.2018.09.039. | Irrelevant Topic |
| 1327 | Menees SB, Elliott E, Govani S, Anastassiades C, Judd S, Urganus A, et al. The impact of bowel cleansing on follow-up recommendations in average-risk patients with a normal colonoscopy. Am J Gastroenterol. 2014;109(2):148-154. doi: 10.1038/ajg.2013.243. | Irrelevant Topic |
| 1328 | Menees SB, Elliott E, Govani S, Anastassiades C, Schoenfeld P. Adherence to recommended intervals for surveillance colonoscopy in average-risk patients with 1 to 2 small (<1 cm) polyps on screening colonoscopy. Gastrointest Endosc. 2014;79(4):551-557. doi: 10.1016/j.gie.2014.01.029. | Irrelevant Topic |
| 1329 | Menees SB, Kim HM, Schoenfeld P. Split-dose bowel preparation improves adequacy of bowel preparation and gastroenterologists' adherence to National Colorectal Cancer Screening and Surveillance Guidelines. World J Gastroenterol. 2018;24(6):716-724. doi: 10.3748/wjg.v24.i6.716. | Irrelevant Topic |
| 1330 | Menees SB, Kim HM, Wren P, Zikmund-Fisher BJ, Elta GH, Foster S, et al. Patient compliance and suboptimal bowel preparation with split-dose bowel regimen in average-risk screening colonoscopy. Gastrointest Endosc. 2014;79(5):811-820.e813. doi: 10.1016/j.gie.2014.01.024. | Irrelevant Topic |
| 1331 | Miaomiao XU, Xiurong FU. Research progress on risk prediction of bowel preparation failure in colonoscopy patients. Chinese Evidence-based Nursing. 2022;8(11):1469-1472. doi: 10.12102/j.issn.2095-8668.2022.11.009. | Irrelevant Topic |
| 1332 | Miller SJ, Itzkowitz SH, Shah B, Jandorf L. Bowel prep quality in patients of low socioeconomic status undergoing screening colonoscopy with patient navigation. Health Educ Behav. 2016;43(5):537-542. doi: 10.1177/1090198115606907. | Irrelevant Topic |
| 1333 | Min LI, Xueping LU, Weiwei B, Taohua Z, Lili WEI. Construction and evaluation of a risk prediction model for hypoglycemia in colonoscopy patients. Chinese Journal of Nursing. 2024;59(1):64-69. doi: 10.3761/j.issn.0254-1769.2024.01.009. | Irrelevant Topic |
| 1334 | Mishra R, Kaufman D, Mattern J, 3rd, Dutta SK. Severe hyperphosphatemia and hypocalcemia caused by bowel preparation for colonoscopy using oral sodium phosphate in end-stage renal disease. Endoscopy. 2005;37(12):1259-1260. doi: 10.1055/s-2005-921155. | Irrelevant Topic |
| 1335 | Misra SP, Dwivedi M, Sharma K. Colon tumors and colonoscopy. Endoscopy. 2011;43(11):985-989. doi: 10.1055/s-0031-1291436. | Irrelevant Topic |
| 1336 | Mitsuhashi S, Azari J, Dioguardi V, Bilello J, Tang M, Kastenberg D. Age matters: adenoma detection rates in average-risk screening patients aged 45 to 49 compared with those aged 50 to 54. J Clin Gastroenterol. 2024;58(9):896-901. doi: 10.1097/mcg.0000000000001953. | Irrelevant Topic |
| 1337 | Mitsuzaki K, Iinuma G, Morimoto T, Miyake M, Tomimatsu H. Computed tomographic colonography with a reduced dose of laxative using a novel barium sulfate contrast agent in Japan. Jpn J Radiol. 2019;37(3):245-254. doi: 10.1007/s11604-018-0800-x. | Irrelevant Topic |
| 1338 | Mizrahi M, Mintz Y, Rivkind A, Kisselgoff D, Libson E, Brezis M, et al. A prospective study assessing the efficacy of abdominal computed tomography scan without bowel preparation in diagnosing intestinal wall and luminal lesions in patients presenting to the emergency room with abdominal complaints. World J Gastroenterol. 2005;11(13):1981-1986. doi: 10.3748/wjg.v11.i13.1981. | Irrelevant Topic |
| 1339 | Mizuno Y, Shimura T, Nukui T, Uno K, Nishigaki R, Kojima Y, et al. Useful bowel preparation with ultralow-volume (500 mL) polyethylene glycol for colonoscopy: a retrospective study. Dig Dis. 2025;43(2):246-252. doi: 10.1159/000543858. | Irrelevant Topic |
| 1340 | Moen S, Vuik FER, Voortman T, Kuipers EJ, Spaander MCW. Predictors of gastrointestinal transit times in colon capsule endoscopy. Clin Transl Gastroenterol. 2022;13(6):e00498. doi: 10.14309/ctg.0000000000000498. | Irrelevant Topic |
| 1341 | Mohammad Azmi N, Abdul Jalal MI, Mohd Ashar SH, Mohd Nazri MI, Jie Y, Ganeson N, et al. A prospective single-masked, non-inferiority, parallel-group randomized controlled trial of the efficacy of a ChatGPT-based AI chatbot to improve Boston bowel preparation scores for colonoscopy preparation: a trial protocol. Plos One. 2025;20(10):e0334349. doi: 10.1371/journal.pone.0334349. | Irrelevant Topic |
| 1342 | Mohammed RA, Lafi SY. Effect of body size on quality of bowel preparation among patients experiencing colonoscopy. Gastroenterol Nurs. 2021;44(2):122-128. doi: 10.1097/sga.0000000000000557. | Irrelevant Topic |
| 1343 | Mooiweer E, Fidder HH, Siersema PD, Laheij RJ, Oldenburg B. Fecal calprotectin testing can identify ineffective colorectal cancer surveillance procedures in patients with longstanding colitis. Inflamm Bowel Dis. 2014;20(6):1079-1084. doi: 10.1097/mib.0000000000000054. | Irrelevant Topic |
| 1344 | Mooiweer E, Fidder HH, Siersema PD, Laheij RJ, Oldenburg B. Fecal hemoglobin and calprotectin are equally effective in identifying patients with inflammatory bowel disease with active endoscopic inflammation. Inflamm Bowel Dis. 2014;20(2):307-314. doi: 10.1097/01.MIB.0000438428.30800.a6. | Irrelevant Topic |
| 1345 | Mooiweer E, Severs M, Schipper ME, Fidder HH, Siersema PD, Laheij RJ, et al. Low fecal calprotectin predicts sustained clinical remission in inflammatory bowel disease patients: a plea for deep remission. J Crohns Colitis. 2015;9(1):50-55. doi: 10.1093/ecco-jcc/jju003. | Irrelevant Topic |
| 1346 | Mooiweer E, van der Meulen-de Jong AE, Ponsioen CY, van der Woude CJ, van Bodegraven AA, Jansen JM, et al. Incidence of interval colorectal cancer among inflammatory bowel disease patients undergoing regular colonoscopic surveillance. Clin Gastroenterol Hepatol. 2015;13(9):1656-1661. doi: 10.1016/j.cgh.2015.04.183. | Irrelevant Topic |
| 1347 | Moon SY, Kim BC, Sohn DK, Han KS, Kim B, Hong CW, et al. Predictors for difficult cecal insertion in colonoscopy: the impact of obesity indices. World J Gastroenterol. 2017;23(13):2346-2354. doi: 10.3748/wjg.v23.i13.2346. | Irrelevant Topic |
| 1348 | Moon W. Optimal and safe bowel preparation for colonoscopy. Clin Endosc. 2013;46(3):219-223. doi: 10.5946/ce.2013.46.3.219. | Irrelevant Topic |
| 1349 | Moore M, Sharma P. Updates in artificial intelligence in gastroenterology endoscopy in 2020. Current Opinion in Gastroenterology. 2021;37(5):428-433. doi: 10.1097/MOG.0000000000000774. | Irrelevant Topic |
| 1350 | Moreira Ruiz L. Surveillance colonoscopy: risk of colorectal tumors. Gastroenterol Hepatol. 2013;36 Suppl 2:80-85. doi: 10.1016/s0210-5705(13)70057-8. | Irrelevant Topic |
| 1351 | Muñoz García-Borruel M, Hervás Molina AJ, Rodríguez Perálvarez ML, Moreno Rincón E, Pérez Medrano I, Serrano Ruiz FJ, et al. Post-colonoscopy colorectal cancer: characteristics and predictive factors. Med Clin (Barc). 2018;150(1):1-7. doi: 10.1016/j.medcli.2017.04.015. | Irrelevant Topic |
| 1352 | Munson GW, Harewood GC, Francis DL. Time of day variation in polyp detection rate for colonoscopies performed on a 3-hour shift schedule. Gastrointest Endosc. 2011;73(3):467-475. doi: 10.1016/j.gie.2010.07.025. | Irrelevant Topic |
| 1353 | Mussetto A, Frazzoni L, Paggi S, Dari S, Laterza L, Radaelli F, et al. Split dosing with a low-volume preparation is not inferior to split dosing with a high-volume preparation for bowel cleansing in patients with a history of colorectal resection: a randomized trial. Endoscopy. 2015;47(10):917-924. doi: 10.1055/s-0034-1391987. | Irrelevant Topic |
| 1354 | Nadeem M, Parava P, Bitar H, Hanafi M, Mahmood S, Saleem R, et al. Does finding cecal adenoma increase the risk of discovering more and advanced adenomas in the remainder of the colon? Southern Medical Journal. 2022;115(9):693-697. doi: 10.14423/SMJ.0000000000001444. | Irrelevant Topic |
| 1355 | Nadimi ES, Braun JM, Schelde-Olesen B, Khare S, Gogineni VC, Blanes-Vidal V, et al. Towards full integration of explainable artificial intelligence in colon capsule endoscopy's pathway. Sci Rep. 2025;15(1):5960. doi: 10.1038/s41598-025-89648-z. | Irrelevant Topic |
| 1356 | Nagarajan KV, Bhat N. Imaging colonic polyps in 2024. Indian J Gastroenterol. 2024;43(5):954-965. doi: 10.1007/s12664-024-01679-y. | Irrelevant Topic |
| 1357 | Nagata K, Endo S, Honda T, Yasuda T, Hirayama M, Takahashi S, et al. Accuracy of CT colonography for detection of polypoid and nonpolypoid neoplasia by gastroenterologists and radiologists: a nationwide multicenter study in Japan. American Journal of Gastroenterology. 2017;112(1):163‐171. doi: 10.1038/ajg.2016.478. | Irrelevant Topic |
| 1358 | Nagata K, Iida N, Kanazawa H, Fujiwara M, Mogi T, Mitsushima T, et al. Effect of listening to music and essential oil inhalation on patients undergoing screening CT colonography: a randomized controlled trial. European Journal of Radiology. 2014;83(12):2172‐2176. doi: 10.1016/j.ejrad.2014.09.016. | Irrelevant Topic |
| 1359 | Nagata K, Okawa T, Honma A, Endo S, Kudo S, Yoshida H. Full-laxative versus minimum-laxative fecal-tagging CT colonography using 64-detector row CT: prospective blinded comparison of diagnostic performance, tagging quality, and patient acceptance. Academic radiology. 2009;16(7):780-789. doi: 10.1016/j.acra.2008.12.027. | Irrelevant Topic |
| 1360 | Nagata N, Sakamoto K, Arai T, Niikura R, Shimbo T, Shinozaki M, et al. Predictors for cecal insertion time: the impact of abdominal visceral fat measured by computed tomography. Dis Colon Rectum. 2014;57(10):1213-1219. doi: 10.1097/dcr.0000000000000203. | Irrelevant Topic |
| 1361 | Nagata N, Takeuchi T, Masuoka H, Aoki R, Ishikane M, Iwamoto N, et al. Human gut microbiota and its metabolites impact immune responses in COVID-19 and its complications. Gastroenterology. 2023;164(2):272-288. doi: 10.1053/j.gastro.2022.09.024. | Irrelevant Topic |
| 1362 | Nagrath N, Phull PS. Variation in caecal intubation rates between screening and symptomatic patients. United European Gastroenterol J. 2014;2(4):295-300. doi: 10.1177/2050640614536898. | Irrelevant Topic |
| 1363 | Nalankilli K, Gibson DJ, Anwar S, Con D, Chen H, Secomb R, et al. Split-dose 1 L polyethylene glycol (PEG) with ascorbate is non-inferior to split-dose PEG with sodium picosulfate and magnesium citrate with similar tolerability: a randomized study. JGH Open. 2021;5(9):1026‐1032. doi: 10.1002/jgh3.12626. | Irrelevant Topic |
| 1364 | Nalluri-Butz H, Bobel MC, Nugent J, Boatman S, Emanuelson R, Melton-Meaux G, et al. A pilot study demonstrating the impact of surgical bowel preparation on intestinal microbiota composition following colon and rectal surgery. Scientific Reports. 2022;12(1). doi: 10.1038/s41598-022-14819-1. | Irrelevant Topic |
| 1365 | Nally DM, Ballester AW, Valentelyte G, Kavanagh DO. The contribution of endoscopy quality measures to the development of interval colorectal cancers in the screening population: a systematic review. Int J Colorectal Dis. 2019;34(1):123-140. doi: 10.1007/s00384-018-3182-8. | Irrelevant Topic |
| 1366 | Nam J, Lee J, Kim J, Kang H, Jang D, Lim Y, et al. Factors for cecal intubation time during colonoscopy in women: impact of surgical history. Saudi Journal of Gastroenterology. 2019;25(6):377-383. doi: 10.4103/sjg.SJG_9_19. | Irrelevant Topic |
| 1367 | Nam SJ, Kim YJ, Keum B, Lee JM, Kim SH, Choi HS, et al. Impact of diet restriction on bowel preparation for colonoscopy. Medicine (Baltimore). 2018;97(41):e12645. doi: 10.1097/md.0000000000012645. | Irrelevant Topic |
| 1368 | Nam SY, Choi IJ, Park KW, Ryu KH, Kim BC, Sohn DK, et al. Risk of hemorrhagic gastropathy associated with colonoscopy bowel preparation using oral sodium phosphate solution. Endoscopy. 2010;42(2):109-113. doi: 10.1055/s-0029-1243797. | Irrelevant Topic |
| 1369 | Napolitano D, Lo Cascio A, Bozzetti M, Povoli A, Grubissa S, Molino L, et al. Patient satisfaction with IBD undergoing colonoscopy: a multicenter cross-sectional study. J Clin Med. 2025;14(8). doi: 10.3390/jcm14082562. | Irrelevant Topic |
| 1370 | Nass KJ, van der Vlugt M, Elfrink AKE, van den Brand CL, Wilschut JA, Fockens P, et al. Case-mix adjustment to compare colonoscopy performance between endoscopy centers: a nationwide registry study. Endoscopy. 2022;54(5):455-462. doi: 10.1055/a-1637-9651. | Irrelevant Topic |
| 1371 | Nayor J, Saltzman JR, Campbell EJ, Perencevich ML, Jajoo K, Richter JM. Impact of physician compliance with colonoscopy surveillance guidelines on interval colorectal cancer. Gastrointest Endosc. 2017;85(6):1263-1270. doi: 10.1016/j.gie.2016.10.041. | Irrelevant Topic |
| 1372 | Nee J, Chippendale RZ, Feuerstein JD. Screening for colon cancer in older adults: risks, benefits, and when to stop. Mayo Clin Proc. 2020;95(1):184-196. doi: 10.1016/j.mayocp.2019.02.021. | Irrelevant Topic |
| 1373 | Neilson LJ, Thirugnanasothy S, Rees CJ. Colonoscopy in the very elderly. Br Med Bull. 2018;127(1):33-41. doi: 10.1093/bmb/ldy018. | Irrelevant Topic |
| 1374 | Neri E, Mantarro A, Faggioni L, Scalise P, Bemi P, Pancrazi F, et al. CT colonography with rectal iodine tagging: feasibility and comparison with oral tagging in a colorectal cancer screening population. European Journal of Radiology. 2015;84(9):1701-1707. doi: 10.1016/j.ejrad.2015.05.025. | Irrelevant Topic |
| 1375 | Neumann H, Latorre M, Zimmerman T, Lang G, Samarasena J, Gross S, et al. A multicenter, prospective, inpatient feasibility study to evaluate the use of an intra-colonoscopy cleansing device to optimize colon preparation in hospitalized patients: the REDUCE study. BMC Gastroenterol. 2021;21(1):232. doi: 10.1186/s12876-021-01817-2. | Irrelevant Topic |
| 1376 | Niedermaier T, Amitay EL, Gies A, Weigl K, Hoffmeister M, Brenner H. Impact of inadequate bowel cleansing on colonoscopic findings in routine screening practice. Clin Transl Gastroenterol. 2020;11. | Irrelevant Topic |
| 1377 | Niikura R, Nagata N, Yamada A, Honda T, Hasatani K, Ishii N, et al. Efficacy and safety of early vs elective colonoscopy for acute lower gastrointestinal bleeding. Gastroenterology. 2020;158(1):168-+. doi: 10.1053/j.gastro.2019.09.010. | Irrelevant Topic |
| 1378 | Niv Y. Polyp detection rate may predict adenoma detection rate: a meta-analysis. European Journal of Gastroenterology & Hepatology. 2018;30(3):247-251. doi: 10.1097/MEG.0000000000001062. | Irrelevant Topic |
| 1379 | Nsibi S, Nakhli A, Ben Mehrez C, Hemdani N, Benzarti Z, Bouchabou B, et al. Conventional regimen versus split-dose regimen in bowel preparation: results of a randomized controlled trial. Tunisie Medicale. 2025;103(5):632‐636. doi: 10.62438/tunismed.v103i5.5514. | Irrelevant Topic |
| 1380 | Nusrat S, Mahmood S, Bitar H, Tierney WM, Bielefeldt K, Madhoun MF. The impact of chronic opioid use on colonoscopy outcomes. Dig Dis Sci. 2015;60(4):1016-1023. doi: 10.1007/s10620-015-3639-3. | Irrelevant Topic |
| 1381 | Obuch JC, Ahnen DJ. Colorectal cancer genetics is changing everything. Gastroenterology Clinics of North America. 2016;45(3):459-+. doi: 10.1016/j.gtc.2016.04.005. | Irrelevant Topic |
| 1382 | O'Connell BM, Crockett SD. The clinical impact of serrated colorectal polyps. Clinical Epidemiology. 2017;9:113-125. doi: 10.2147/CLEP.S106257. | Irrelevant Topic |
| 1383 | Ogata N, Maeda Y, Misawa M, Takenaka K, Takabayashi K, Iacucci M, et al. Artificial intelligence-assisted video colonoscopy for disease monitoring of ulcerative colitis: a prospective study. Journal of Crohn's and Colitis. 2025;19(1). doi: 10.1093/ecco-jcc/jjae080. | Irrelevant Topic |
| 1384 | Ogino N, Aridome G, Oshima J, Shibata M, Watanabe T, Kume K, et al. Serum albumin concentrations predict hypovolaemia caused by polyethylene glycol plus ascorbic acid prior to colonoscopy in elderly patients. Drugs Aging. 2016;33(5):355-363. doi: 10.1007/s40266-016-0355-4. | Irrelevant Topic |
| 1385 | Oh SY, Sohn CI, Sung IK, Park DI, Kang MS, Yoo TW, et al. Factors affecting the technical difficulty of colonoscopy. Hepatogastroenterology. 2007;54(77):1403-1406. | Irrelevant Topic |
| 1386 | Ohmiya N, Hotta N, Mitsufuji S, Nakamura M, Omori T, Maeda K, et al. Multicenter feasibility study of bowel preparation with castor oil for colon capsule endoscopy. Dig Endosc. 2019;31(2):164-172. doi: 10.1111/den.13259. | Irrelevant Topic |
| 1387 | Oliva S, Veraldi S, Russo G, Aloi M, Rizzello F, Gionchetti P, et al. Pan-enteric capsule endoscopy to characterize Crohn's disease phenotypes and predict clinical outcomes in children and adults: the Bomiro study. Inflammatory Bowel Diseases. 2025;31(3):636-646. doi: 10.1093/ibd/izae052. | Irrelevant Topic |
| 1388 | Ooi ET, Menon SK, Menon J. A randomized prospective study to investigate the electrolyte abnormalities associated with oral sodium phosphate (NaP) for bowel preparation of patients for colonoscopy. J Gastroenterol Hepatol. 2011;26:122. doi: 10.1111/j.1440-1746.2011.06898.x. | Irrelevant Topic |
| 1389 | Ori Y, Herman M, Tobar A, Chernin G, Gafter U, Chagnac A, et al. Acute phosphate nephropathy-an emerging threat. American Journal of the Medical Sciences. 2008;336(4):309-314. doi: 10.1097/MAJ.0b013e318167410c. | Irrelevant Topic |
| 1390 | O'Shea A, Foran AT, Murray TE, Thornton E, Dunne R, Lee MJ, et al. Quality of same-day CT colonography following incomplete optical colonoscopy. European Radiology. 2020;30(12):6508-6516. doi: 10.1007/s00330-020-06979-3. | Irrelevant Topic |
| 1391 | O'Shea A, Murray T, Morrin MM, Lee MJ, Thornton E. Incidence of clinically significant perforation at low dose non-contrast CT and its value prior to same day CT colonography following incomplete colonoscopy. Abdom Radiol (NY). 2020;45(4):1044-1048. doi: 10.1007/s00261-019-02062-8. | Irrelevant Topic |
| 1392 | Oussalah A, Laurent V, Bruot O, Bressenot A, Bigard MA, Régent D, et al. Diffusion-weighted magnetic resonance without bowel preparation for detecting colonic inflammation in inflammatory bowel disease. Gut. 2010;59(8):1056-1065. doi: 10.1136/gut.2009.197665. | Irrelevant Topic |
| 1393 | Ow TW, Angelica B, Burn S, Chu M, Lee SZ, Lin R, et al. Comparing the resource implications of old and new colorectal adenoma surveillance guidelines in Australia. Internal Medicine Journal. 2024;54(2):250-257. doi: 10.1111/imj.16149. | Irrelevant Topic |
| 1394 | Ozdemir S, Gulpinar K, Ozis SE, Sahli Z, Kesikli SA, Korkmaz A, et al. The effects of preoperative oral antibiotic use on the development of surgical site infection after elective colorectal resections: a retrospective cohort analysis in consecutively operated 90 patients. International Journal of Surgery. 2016;33:102-108. doi: 10.1016/j.ijsu.2016.07.060. | Irrelevant Topic |
| 1395 | Ozsunar Y, Coskun G, Delibaş N, Uz B, Yükselen V. Diagnostic accuracy and tolerability of contrast enhanced CT colonoscopy in symptomatic patients with increased risk for colorectal cancer. Eur J Radiol. 2009;71(3):513-518. doi: 10.1016/j.ejrad.2008.05.013. | Irrelevant Topic |
| 1396 | Padmanabhan H, Rothnie A, Higgins A, Grewal A, Arndtz K, Nevill AM, et al. Preassessment interview improves the efficacy and safety of bowel preparation for colonoscopy. Can J Gastroenterol Hepatol. 2016;2016:7591637. doi: 10.1155/2016/7591637. | Irrelevant Topic |
| 1397 | Paggi S, Radaelli F, Senore C, Maselli R, Amato A, Andrisani G, et al. Linked-color imaging versus white-light colonoscopy in an organized colorectal cancer screening program. Gastrointest Endosc. 2020;92(3):723‐730. doi: 10.1016/j.gie.2020.05.044. | Irrelevant Topic |
| 1398 | Paik N, Kim ER, Kim TJ, Hong SN, Chang DK, Kim YH. Usefulness of personal bowel habits as a predictive factor for inadequate bowel preparation for colonoscopy: a prospective questionnaire-based observational study. Gut Liver. 2019;13(2):169-175. doi: 10.5009/gnl18236. | Irrelevant Topic |
| 1399 | Paik N, Kim MJ. Prediction of bowel preparation scale for colonoscopy according to the personal bowel habits - a prospective study. Gastrointest Endosc. 2018;87(6):AB151-AB151. | Irrelevant Topic |
| 1400 | Pan YL, Chen L, Kang XY, Wang LM, Zhao JH, Zhang RC, et al. Superiority of a procedure-related model for predicting inadequate bowel preparation in patients undergoing colonoscopy: a multicenter prospective study. Gastrointest Endosc. 2021;93(6):AB41-AB41. | Irrelevant Topic |
| 1401 | Panteris V, Haringsma J, Kuipers EJ. Colonoscopy perforation rate, mechanisms and outcome: from diagnostic to therapeutic colonoscopy. Endoscopy. 2009;41(11):941-951. doi: 10.1055/s-0029-1215179. | Irrelevant Topic |
| 1402 | Papastergiou V, Papasavvas S, Mathou N, Giannakopoulos A, Evgenidi A, Karagiannis JA, et al. A delayed onset of bowel activity after the start of conventional polyethylene glycol predicts inadequate colon cleansing before colonoscopy: a prospective observational study. United European Gastroenterol J. 2016;4(2):199-206. doi: 10.1177/2050640615608572. | Irrelevant Topic |
| 1403 | Parente F, Vailati C, Bargiggia S, Manes G, Fontana P, Masci E, et al. 2-litre polyethylene glycol-citrate-simethicone plus bisacodyl versus 4-litre polyethylene glycol as preparation for colonoscopy in chronic constipation. Digestive and Liver Disease. 2015;47(10):857‐863. doi: 10.1016/j.dld.2015.06.008. | Irrelevant Topic |
| 1404 | Park DI, Kim HJ, Park JH, Cho YK, Sohn CI, Jeon WK, et al. Factors affecting abdominal pain during colonoscopy. Eur J Gastroenterol Hepatol. 2007;19(8):695-699. doi: 10.1097/01.meg.0000219097.32811.24. | Irrelevant Topic |
| 1405 | Park HJ, Chae MH, Kim HS, Kim JW, Kim MY, Baik SK, et al. Colon transit time may predict inadequate bowel preparation in patients with chronic constipation. Intest Res. 2015;13(4):339-345. doi: 10.5217/ir.2015.13.4.339. | Irrelevant Topic |
| 1406 | Park JH, Moon HS, Kwon IS, Kim JS, Kang SH, Lee ES, et al. Quality of preoperative colonoscopy affects missed postoperative adenoma detection in colorectal cancer patients. Dig Dis Sci. 2020;65(7):2063-2070. doi: 10.1007/s10620-019-05912-z. | Irrelevant Topic |
| 1407 | Park Y, Jeon TJ, Park JY, Park SJ, Cheon JH, Kim TI, et al. Comparison of clipping with and without epinephrine injection for the prevention of post-polypectomy bleeding in pedunculated colon polyps. J Gastroenterol Hepatol. 2015;30(10):1499-1506. doi: 10.1111/jgh.12994. | Irrelevant Topic |
| 1408 | Pathipati MP, Silvernale CJ, Barshop KG, Ha JB, Richter JM, Staller KD. Rectal evacuation disorders are associated with poor bowel preparation in patients with chronic constipation: results from two centers. J Clin Gastroenterol. 2022;56(5):438-443. doi: 10.1097/mcg.0000000000001593. | Irrelevant Topic |
| 1409 | Pecere S, Senore C, Hassan C, Riggi E, Segnan N, Pennazio M, et al. Accuracy of colon capsule endoscopy for advanced neoplasia. Gastrointest Endosc. 2020;91(2):406-+. doi: 10.1016/j.gie.2019.09.041. | Irrelevant Topic |
| 1410 | Peller M, Keswani RN, Sakuraba A, Micic D. Bariatric bypass surgery is a risk factor for incomplete colonoscopy preparation. Dig Dis Sci. 2022;67(7):3185-3191. doi: 10.1007/s10620-021-07274-x. | Irrelevant Topic |
| 1411 | Peng L, Balavarca Y, Niedermaier T, Weigl K, Hoffmeister M, Brenner H. Risk-adapted cutoffs in colorectal cancer screening by fecal immunochemical tests. Am J Gastroenterol. 2020;115(7):1110-1116. doi: 10.14309/ajg.0000000000000579. | Irrelevant Topic |
| 1412 | Petroianu A, Alberti LR, de Lima DC, Hauter HL, Rodrigues KC, Mendes JC. [Colonoscopic findings in asymptomatic people]. Arq Gastroenterol. 2009;46(3):173-178. doi: 10.1590/s0004-28032009000300006. | Irrelevant Topic |
| 1413 | Phisalprapa P, Ngorsuraches S, Wanishayakorn T, Kositamongkol C, Supakankunti S, Chaiyakunapruk N. Estimating the preferences and willingness-to-pay for colorectal cancer screening: an opportunity to incorporate the perspective of population at risk into policy development in Thailand. J Med Econ. 2021;24(1):226-233. doi: 10.1080/13696998.2021.1877145. | Irrelevant Topic |
| 1414 | Pickhardt PJ, Taylor AJ, Kim DH, Reichelderfer M, Gopal DV, Pfau PR. Screening for colorectal neoplasia with CT colonography: initial experience from the 1st year of coverage by third-party payers. Radiology. 2006;241(2):417-425. doi: 10.1148/radiol.2412052007. | Irrelevant Topic |
| 1415 | Pilonis ND, Bugajski M, Wieszczy P, Franczyk R, Didkowska J, Wojciechowska U, et al. Long-term colorectal cancer incidence and mortality after a single negative screening colonoscopy. Ann Intern Med. 2020;173(2):81-91. doi: 10.7326/m19-2477. | Irrelevant Topic |
| 1416 | Pilz JB, Portmann S, Peter S, Beglinger C, Degen L. Colon capsule endoscopy compared to conventional colonoscopy under routine screening conditions. Bmc Gastroenterology. 2010;10. doi: 10.1186/1471-230X-10-66. | Irrelevant Topic |
| 1417 | Pipek B, Valentová H, Fojtík P, Urban O. Faecal microbiota transplantation in the treatment of recurrent intestinal Clostridioides difficile infection - a ten-year single-center experience. Cas Lek Cesk. 2022;161(3-4):126-130. | Irrelevant Topic |
| 1418 | Pirró N, Ouaissi M, Sielezneff I, Fakhro A, Pieyre A, Consentino B, et al. [Feasibility of colorectal surgery without colonic preparation. A prospective study]. Ann Chir. 2006;131(8):442-446. doi: 10.1016/j.anchir.2006.03.016. | Irrelevant Topic |
| 1419 | Poerregaard A, Wewer AV, Becker PU, Bendtsen F, Krasilnikoff PA, Matzen P. [Pediatric colonoscopy]. Ugeskr Laeger. 1998;160(14):2105-2108. | Irrelevant Topic |
| 1420 | Pohl J, Halphen M, Kloess HR, Fischbach W. Impact of the quality of bowel cleansing on the efficacy of colonic cancer screening: a prospective, randomized, blinded study. Plos One. 2015;10(5). doi: 10.1371/journal.pone.0126067. | Irrelevant Topic |
| 1421 | Polatkan SAV, Gunay-Polatkan S, Isik O, Sigirli D. Using the cardiac-electrophysiological balance index to predict arrhythmia risk after colonoscopy. Medicina (Kaunas). 2024;61(1). doi: 10.3390/medicina61010013. | Irrelevant Topic |
| 1422 | Ponte A, Pinho R, Rodrigues A, Silva J, Rodrigues J, Sousa M, et al. Predictive factors of an incomplete examination and inadequate small-bowel cleanliness during capsule endoscopy. Revista Espanola De Enfermedades Digestivas. 2018;110(10):605-611. doi: 10.17235/reed.2018.4834/2017. | Irrelevant Topic |
| 1423 | Pontone S, Angelini R, Standoli M, Patrizi G, Culasso F, Pontone P, et al. Low-volume plus ascorbic acid vs high-volume plus simethicone bowel preparation before colonoscopy. World Journal of Gastroenterology. 2011;17(42):4689-4695. doi: 10.3748/wjg.v17.i42.4689. | Irrelevant Topic |
| 1424 | Pontone S, Palma R, Panetta C, Eberspacher C, Angelini R, Pironi D, et al. Polyethylene glycol-based bowel preparation before colonoscopy for selected inpatients: a pilot study. J Dig Dis. 2018;19(1):40-47. doi: 10.1111/1751-2980.12571. | Irrelevant Topic |
| 1425 | Provenzale D, Jasperson K, Ahnen DJ, Aslanian H, Bray T, Cannon JA, et al. Colorectal cancer screening, version 1.2015. J Natl Compr Canc Netw. 2015;13(8):959-968; quiz 968. doi: 10.6004/jnccn.2015.0116. | Irrelevant Topic |
| 1426 | Pu L, Lu K, Ovenden A, Rana K, Singh G, Krishnamurthi S, et al. Effect of time of day and specialty on polyp detection rates in Australia. J Gastroenterol Hepatol. 2019;34(5):899-906. doi: 10.1111/jgh.14566. | Irrelevant Topic |
| 1427 | Pullens HJM, Leenders M, Schipper MEI, van Oijen MGH, Siersema PD. No decrease in the rate of early or missed colorectal cancers after colonoscopy with polypectomy over a 10-year period: a population-based analysis. Clinical Gastroenterology and Hepatology. 2015;13(1):140-147. doi: 10.1016/j.cgh.2014.04.032. | Irrelevant Topic |
| 1428 | Qian Y, Dong W, Hu M, Wang L, Zou J, Wu S, et al. Construction and validation of a nomogram model for risk of inadequate bowel preparation in patients undergoing colonoscopy. Chinese Journal of Gastroenterology. 2025;30(5):290-295. doi: 10.3969/j.issn.1008-7125.2025.05.006. | Irrelevant Topic |
| 1429 | Quach DT, Hiyama T, Nguyen TA, Ly HQ, Tanaka S. Asia-Pacific colorectal screening score: a useful tool to stratify risk for colorectal advanced neoplasms in Vietnamese patients with irritable bowel syndrome. J Gastroenterol Hepatol. 2018;33(1):150-155. doi: 10.1111/jgh.13821. | Irrelevant Topic |
| 1430 | Quintero E, Alarcón-Fernández O, Jover R. Colonoscopy quality control as a requirement of colorectal cancer screening. Gastroenterol Hepatol. 2013;36(9):597-605. doi: 10.1016/j.gastrohep.2013.02.005. | Irrelevant Topic |
| 1431 | Radaelli F, Meucci G, Imperiali G, Spinzi G, Strocchi E, Terruzzi V, et al. High-dose senna compared with conventional PEG-ES lavage as bowel preparation for elective colonoscopy: a prospective, randomized, investigator-blinded trial. Am J Gastroenterol. 2005;100(12):2674-2680. doi: 10.1111/j.1572-0241.2005.00335.x. | Irrelevant Topic |
| 1432 | Radaelli F, Meucci G, Sgroi G, Minoli G. Technical performance of colonoscopy: the key role of sedation/analgesia and other quality indicators. Am J Gastroenterol. 2008;103(5):1122-1130. doi: 10.1111/j.1572-0241.2007.01778.x. | Irrelevant Topic |
| 1433 | Radaelli F, Paggi S, Hassan C, Senore C, Fasoli R, Anderloni A, et al. Split-dose preparation for colonoscopy increases adenoma detection rate: a randomised controlled trial in an organised screening programme. Gut. 2017;66(2):270‐277. doi: 10.1136/gutjnl-2015-310685. | Irrelevant Topic |
| 1434 | Radaelli F, Paggi S, Repici A, Gullotti G, Cesaro P, Rotondano G, et al. Barriers against split-dose bowel preparation for colonoscopy. Gut. 2017;66(8):1428-1433. doi: 10.1136/gutjnl-2015-311049. | Irrelevant Topic |
| 1435 | Ramakrishnan K, Scheid DC. Predictors of incomplete flexible sigmoidoscopy. J Am Board Fam Pract. 2003;16(6):478-484. doi: 10.3122/jabfm.16.6.478. | Irrelevant Topic |
| 1436 | Ramakrishnan S, Goswami P, Dow F, Ocakkuran S, Sadigh D. Severe hyponatraemia following underwater colonoscopy and polypectomy in an elderly woman: a multifactorial case involving bowel prep, procedural stress, and occult malignancy. Cureus. 2025;17(8):e90810. doi: 10.7759/cureus.90810. | Irrelevant Topic |
| 1437 | Ramprasad C, Ng S, Zhang Y, Liang PS. Low-residue diet for colonoscopy in veterans: risk factors for inadequate bowel preparation and patient satisfaction and compliance. Plos One. 2020;15(5):e0233346. doi: 10.1371/journal.pone.0233346. | Irrelevant Topic |
| 1438 | Ramprasad C, Saini D, Krasnovsky L, del Carmen H, Chandra R, McGregor R, et al. Text message system for the prediction of colonoscopy bowel preparation adequacy prior to colonoscopy: an artificial intelligence-image classification algorithm based on images of stool output. Gastroenterology. 2023;164(6):S264-S264. | Irrelevant Topic |
| 1439 | Rasmussen M, Bohlbro K, Qvist N. Oral sodium phosphate compared with water enemas combined with bisacodyl as bowel preparation for elective colonoscopy. Scand J Gastroenterol. 2003;38(10):1090-1094. | Irrelevant Topic |
| 1440 | Rasmussen MS, Hansen L, Deding U, Ellebæk MB, Kjeldsen J, Bjørsum-Meyer T. Applicability of colon capsule endoscopy for monitoring ulcerative colitis: a systematic review. Scand J Gastroenterol. 2025;60(4):336-342. doi: 10.1080/00365521.2025.2475081. | Irrelevant Topic |
| 1441 | Rasschacrt G, Galdon MG, Vandevelde J, Eisendrath P. Continuous ADR50 monitoring through automated linkage between endoscopy and pathology: a quality improvement initiative in a Brussels public hospital. Acta Gastro-Enterologica Belgica. 2022;85(2):259-266. doi: 10.51821/85.2.9706. | Irrelevant Topic |
| 1442 | Rastogi A, Early DS, Gupta N, Bansal A, Singh V, Ansstas M, et al. A multicenter, prospective, randomized controlled trial comparing standard definition white light (SDWL), high definition white light (HDWL) and narrow band imaging (NBI) colonoscopy for the detection of colon polyps and real time prediction of histology. Gastrointest Endosc. 2010;71(5):AB142. doi: 10.1016/j.gie.2010.03.142. | Irrelevant Topic |
| 1443 | Rawl SM, Perkins SM, Tong Y, Katz ML, Carter-Bawa L, Imperiale TF, et al. Patient navigation plus tailored digital video disc increases colorectal cancer screening among low-income and minority patients who did not attend a scheduled screening colonoscopy: a randomized trial. Annals of Behavioral Medicine. 2024;58(5):314-327. doi: 10.1093/abm/kaae013. | Irrelevant Topic |
| 1444 | Real Noval H, Fernández Fernández J, Castelo Álvarez E, Fernández Mallo J. [Do patients with diverticula have a higher risk of developing polyps and colorectal cancer? Study in a Spanish population]. Rev Esp Salud Publica. 2025;99. | Irrelevant Topic |
| 1445 | Rebhun J, Pagani W, Xia YL, Shuja A. Effect of the weekend on bowel preparation quality in outpatient colonoscopies. Dig Dis Sci. 2022;67(4):1231-1237. doi: 10.1007/s10620-021-07037-8. | Irrelevant Topic |
| 1446 | Rebhun J, Yellen M, Xia YL, Shuja A. Do gastroenterologists have a "weak" day? Impact of day of the week on the quality of outpatient screening colonoscopies. J Clin Gastroenterol. 2022;56(9):764-771. doi: 10.1097/MCG.0000000000001599. | Irrelevant Topic |
| 1447 | Reddy P, Mencin A, Lebwohl B. Risk factors for suboptimal bowel preparation for colonoscopy in pediatric patients. J Pediatr Gastroenterol Nutr. 2021;73(1):e1-e6. doi: 10.1097/mpg.0000000000003114. | Irrelevant Topic |
| 1448 | Rees CJ, Rajasekhar PT, Rutter MD, Dekker E. Quality in colonoscopy: European perspectives and practice. Expert Review of Gastroenterology & Hepatology. 2014;8(1):29-47. doi: 10.1586/17474124.2014.858599. | Irrelevant Topic |
| 1449 | Reumkens A, Masclee AA, Winkens B, van Deursen CT, Sanduleanu S, Bakker CM. Prevalence of hypokalemia before and after bowel preparation for colonoscopy in high-risk patients. Gastrointest Endosc. 2017;86(4):673-679. doi: 10.1016/j.gie.2017.01.040. | Irrelevant Topic |
| 1450 | Reumkens A, Masclee AAM, Bakker CM. Postcolonoscopy mortality: bowel preparation to blame? Gastrointest Endosc. 2017;86(4):744-745. doi: 10.1016/j.gie.2017.01.008. | Irrelevant Topic |
| 1451 | Reuterskiöld MH, Lasson A, Svensson E, Kilander A, Stotzer PO, Hellström M. Diagnostic performance of computed tomography colonography in symptomatic patients and in patients with increased risk for colorectal disease. Acta Radiol. 2006;47(9):888-898. doi: 10.1080/02841850600964966. | Irrelevant Topic |
| 1452 | Rex DK. Colonoscopy. Endoscopy. 2013;45(9):756-761. doi: 10.1055/s-0033-1344630. | Irrelevant Topic |
| 1453 | Rex DK. Polyp detection at colonoscopy: endoscopist and technical factors. Best Pract Res Clin Gastroenterol. 2017;31(4):425-433. doi: 10.1016/j.bpg.2017.05.010. | Irrelevant Topic |
| 1454 | Ribeiro T, Saraiva MJM, Afonso J, Cardoso P, Mendes F, Martins M, et al. Design of a convolutional neural network as a deep learning tool for the automatic classification of small-bowel cleansing in capsule endoscopy. Medicina-Lithuania. 2023;59(4). doi: 10.3390/medicina59040810. | Irrelevant Topic |
| 1455 | Rice K, Gressard L, DeGroff A, Gersten J, Robie J, Leadbetter S, et al. Increasing colonoscopy screening in disparate populations: results from an evaluation of patient navigation in the New Hampshire Colorectal Cancer Screening Program. Cancer. 2017;123(17):3356-3366. doi: 10.1002/cncr.30761. | Irrelevant Topic |
| 1456 | Rivas JM, Perez A, Hernandez M, Schneider A, Castro FJ. Efficacy of morning-only 4 liter sulfa free polyethylene glycol vs 2 liter polyethylene glycol with ascorbic acid for afternoon colonoscopy. World Journal of Gastroenterology. 2014;20(30):10620‐10627. doi: 10.3748/wjg.v20.i30.10620. | Irrelevant Topic |
| 1457 | Rivero-Sánchez L, Pellisé M. [Bowel preparation for colonoscopy. Any significant progress on the horizon?]. Gastroenterol Hepatol. 2015;38(4):287-300. doi: 10.1016/j.gastrohep.2014.10.008. | Irrelevant Topic |
| 1458 | Robbins EC, Wooldrage K, Stenson I, Pack K, Duffy S, Weller D, et al. Heterogeneity in colorectal cancer incidence among people recommended 3-yearly surveillance post-polypectomy: a validation study. Endoscopy. 2021;53(4):402-410. doi: 10.1055/a-1217-0155. | Irrelevant Topic |
| 1459 | Robinson P, Burnett H, Nicholson DA. The use of minimal preparation computed tomography for the primary investigation of colon cancer in frail or elderly patients. Clin Radiol. 2002;57(5):389-392. doi: 10.1053/crad.2001.0866. | Irrelevant Topic |
| 1460 | Rodrigues RV, Claro I, Lage P, Rosa I, Ferreira S, da Silva JP, et al. Colorectal cancer surveillance in Portuguese families with Lynch syndrome: a cohort study. Int J Colorectal Dis. 2018;33(6):695-702. doi: 10.1007/s00384-018-3002-1. | Irrelevant Topic |
| 1461 | Roepstorff S, Hadi SA, Rasmussen M. Full spectrum endoscopy (FUSE) versus standard forward-viewing endoscope (SFV) in a high-risk population. Scand J Gastroenterol. 2017;52(11):1298-1303. doi: 10.1080/00365521.2017.1363278. | Irrelevant Topic |
| 1462 | Romero RV, Mahadeva S. Factors influencing quality of bowel preparation for colonoscopy. World J Gastrointest Endosc. 2013;5(2):39-46. doi: 10.4253/wjge.v5.i2.39. | Irrelevant Topic |
| 1463 | Ross FA, Park JH, Mansouri D, Little C, Di Rollo DG, Combet E, et al. The role of faecal calprotectin in the identification of colorectal neoplasia in patients attending for screening colonoscopy. Colorectal Dis. 2022;24(2):188-196. doi: 10.1111/codi.15942. | Irrelevant Topic |
| 1464 | Rostom A, Dube C, Bishay K, Antonova L, Heitman SJ, Hilsden R. A randomized clinical prospective trial comparing split-dose picosulfate/magnesium citrate and polyethylene glycol for colonoscopy preparation. Plos One. 2019;14(3):e0211136. doi: 10.1371/journal.pone.0211136. | Irrelevant Topic |
| 1465 | Rotondano G, Rispo A, Bottiglieri ME, De Luca L, Lamanda R, Orsini L, et al. Quality of bowel cleansing in hospitalized patients undergoing colonoscopy: a multicentre prospective regional study. Dig Liver Dis. 2015;47(8):669-674. doi: 10.1016/j.dld.2015.04.013. | Irrelevant Topic |
| 1466 | Rouphael C, Lopez R, McMichael J, Burke CA. ≥3 nonadvanced adenomas are more common in the era of contemporary colonoscopy and not associated with metachronous advanced neoplasia. J Clin Gastroenterol. 2021;55(4):343-349. doi: 10.1097/mcg.0000000000001364. | Irrelevant Topic |
| 1467 | Ruiz-Rodriguez J, Fuente CRD, Nieto MAT, Juanas PB, Núñez IR, Cuevas CM, et al. Uncovering missed opportunities-a root-cause analysis of post-colonoscopy colorectal cancer in a tertiary care setting. Revista Espanola De Enfermedades Digestivas. 2025;117(12). doi: 10.17235/reed.2025.11320/2025. | Irrelevant Topic |
| 1468 | Russmann S, Lamerato L, Marfatia A, Motsko SP, Pezzullo JC, Olds G, et al. Risk of impaired renal function after colonoscopy: a cohort study in patients receiving either oral sodium phosphate or polyethylene glycol. Am J Gastroenterol. 2007;102(12):2655-2663. doi: 10.1111/j.1572-0241.2007.01610.x. | Irrelevant Topic |
| 1469 | Rutter CM, Knudsen AB, Marsh TL, Doria-Rose VP, Johnson E, Pabiniak C, et al. Validation of models used to inform colorectal cancer screening guidelines: accuracy and implications. Medical Decision Making. 2016;36(5):604-614. doi: 10.1177/0272989X15622642. | Irrelevant Topic |
| 1470 | Saar B, Rösch T, Rummeny EJ. Colorectal cancer screening: a challenge for magnetic resonance colonography. Top Magn Reson Imaging. 2002;13(6):427-434. doi: 10.1097/00002142-200212000-00006. | Irrelevant Topic |
| 1471 | Sachdeva R, Tsai S, El Zein M, Tieu A, Abdelgelil A, Besharati S, et al. Predictors of incomplete optical colonoscopy using computed tomographic colonography. Saudi Journal of Gastroenterology. 2016;22(1):43-49. doi: 10.4103/1319-3767.173758. | Irrelevant Topic |
| 1472 | Sadalla S, Lisotti A, Fuccio L, Fusaroli P. Colonoscopy-related colonic ischemia. World J Gastroenterol. 2021;27(42):7299-7310. doi: 10.3748/wjg.v27.i42.7299. | Irrelevant Topic |
| 1473 | Sadeghi A, Peysepar R, Kafash N, Shahrokh S, Ahmadzadeh A. AI-driven prediction of the optimal number of bowel preparation sachets for adequate preparation before colonoscopy. Gastroenterol Hepatol Bed Bench. 2025;18(SI):121-124. doi: 10.22037/ghfbb.v18iSpecialIssue.3196. | Irrelevant Topic |
| 1474 | Safder S, Demintieva Y, Rewalt M, Elitsur Y. Stool consistency and stool frequency are excellent clinical markers for adequate colon preparation after polyethylene glycol 3350 cleansing protocol: a prospective clinical study in children. Gastrointest Endosc. 2008;68(6):1131-1135. doi: 10.1016/j.gie.2008.04.026. | Irrelevant Topic |
| 1475 | Sagami S, Kobayashi T, Kikkawa N, Umeda S, Nakano M, Toyonaga T, et al. Combination of colonoscopy and magnetic resonance enterography is more useful for clinical decision making than colonoscopy alone in patients with complicated Crohn's disease. Plos One. 2019;14(2):e0212404. doi: 10.1371/journal.pone.0212404. | Irrelevant Topic |
| 1476 | Sahebally SM. Predicting suboptimal bowel preparation: taking it up a PEG. Dig Dis Sci. 2017;62(2):289-291. doi: 10.1007/s10620-016-4385-x. | Irrelevant Topic |
| 1477 | Sajid MS, Caswell JF, Abbas MA, Baig MK, McFall MR. Improving the view during flexible sigmoidoscopy: a systematic review of published randomized, controlled trials comparing the use of oral bowel preparation versus enema bowel preparation. Updates Surg. 2015;67(3):247-256. doi: 10.1007/s13304-015-0295-2. | Irrelevant Topic |
| 1478 | Sali L, Falchini M, Bonanomi AG, Castiglione G, Ciatto S, Mantellini P, et al. CT colonography after incomplete colonoscopy in subjects with positive faecal occult blood test. World J Gastroenterol. 2008;14(28):4499-4504. doi: 10.3748/wjg.14.4499. | Irrelevant Topic |
| 1479 | Sali L, Falchini M, Della Monica P, Regge D, Bonanomi AG, Castiglione G, et al. CT colonography before colonoscopy in subjects with positive faecal occult blood test. Preliminary experience. Radiol Med. 2010;115(8):1267-1278. doi: 10.1007/s11547-010-0569-1. | Irrelevant Topic |
| 1480 | Sali L, Grazzini G, Carozzi F, Castiglione G, Falchini M, Mallardi B, et al. Screening for colorectal cancer with FOBT, virtual colonoscopy and optical colonoscopy: study protocol for a randomized controlled trial in the Florence district (SAVE study). Trials. 2013;14:74. doi: 10.1186/1745-6215-14-74. | Irrelevant Topic |
| 1481 | Samarasena JB, Muthusamy VR, Jamal MM. Split-dosed MiraLAX/Gatorade is an effective, safe, and tolerable option for bowel preparation in low-risk patients: a randomized controlled study. Am J Gastroenterol. 2012;107(7):1036-1042. doi: 10.1038/ajg.2012.115. | Irrelevant Topic |
| 1482 | Sanaka MR, Deepinder F, Thota PN, Lopez R, Burke CA. Adenomas are detected more often in morning than in afternoon colonoscopy. Am J Gastroenterol. 2009;104(7):1659-1664; quiz 1665. doi: 10.1038/ajg.2009.249. | Irrelevant Topic |
| 1483 | Sanaka MR, Shah N, Mullen KD, Ferguson DR, Thomas C, McCullough AJ. Afternoon colonoscopies have higher failure rates than morning colonoscopies. Am J Gastroenterol. 2006;101(12):2726-2730. doi: 10.1111/j.1572-0241.2006.00887.x. | Irrelevant Topic |
| 1484 | Sánchez A, Roos VH, Navarro M, Pineda M, Caballol B, Moreno L, et al. Quality of colonoscopy is associated with adenoma detection and postcolonoscopy colorectal cancer prevention in Lynch syndrome. Clin Gastroenterol Hepatol. 2022;20(3):611-621.e619. doi: 10.1016/j.cgh.2020.11.002. | Irrelevant Topic |
| 1485 | Sano W, Fujimori T, Ichikawa K, Sunakawa H, Utsumi T, Iwatate M, et al. Clinical and endoscopic evaluations of sessile serrated adenoma/polyps with cytological dysplasia. Journal of Gastroenterology and Hepatology (Australia). 2018;33(8):1454-1460. doi: 10.1111/jgh.14099. | Irrelevant Topic |
| 1486 | Sano Y, Chiu HM, Li XB, Khomvilai S, Pisespongsa P, Co JT, et al. Standards of diagnostic colonoscopy for early-stage neoplasia: recommendations by an Asian private group. Dig Endosc. 2019;31(3):227-244. doi: 10.1111/den.13330. | Irrelevant Topic |
| 1487 | Saunders JH, Miskovic D, Bowman C, Panto P, Menon A. Colorectal cancer is reliably excluded in the frail and elderly population by minimal preparation CT. Tech Coloproctol. 2014;18(2):137-143. doi: 10.1007/s10151-013-1045-4. | Irrelevant Topic |
| 1488 | Scalvini D, Bezzio C, Maimaris S, Lenti MV, Francesca L, Cappellini A, et al. A multicenter study on bowel preparation in inflammatory bowel disease patients: comparison between 1L-PEG-ASC and 2L-PEG regimens in an outpatient setting. Eur J Gastroenterol Hepatol. 2025;37(10):1127-1134. doi: 10.1097/meg.0000000000003005. | Irrelevant Topic |
| 1489 | Scalvini D, Lenti MV, Maimaris S, Lusetti F, Alimenti E, Fazzino E, et al. Superior bowel preparation quality for colonoscopy with 1L-PEG compared to 2L-PEG and picosulphate: data from a large real-world retrospective outpatient cohort. Dig Liver Dis. 2024;56(11):1906-1913. doi: 10.1016/j.dld.2024.04.026. | Irrelevant Topic |
| 1490 | Schaefer M, Littrell E, Khan A, Patterson ME. Estimated GFR decline following sodium phosphate enemas versus polyethylene glycol for screening colonoscopy: a retrospective cohort study. Am J Kidney Dis. 2016;67(4):609-616. doi: 10.1053/j.ajkd.2015.11.018. | Irrelevant Topic |
| 1491 | Schick BA, McLean CA, Driman DK. Negative colorectal polyp biopsies: the utility of cutting deeper levels. Virchows Archiv. 2015;467(6):635-640. doi: 10.1007/s00428-015-1866-9. | Irrelevant Topic |
| 1492 | Schmidt PN, Hendel JW. [Modest risk of phosphate nephropathy by bowel cleansing]. Ugeskr Laeger. 2010;172(42):2891-2894. | Irrelevant Topic |
| 1493 | Schoen RE, Pinsky PF, Weissfeld JL, Yokochi LA, Church T, Laiyemo AO, et al. Colorectal cancers not detected by screening flexible sigmoidoscopy in the Prostate, Lung, Colorectal, and Ovarian Cancer Screening Trial. Gastrointest Endosc. 2012;75(3):612-620. doi: 10.1016/j.gie.2011.10.024. | Irrelevant Topic |
| 1494 | Schulz C, Müller J, Sauter J, Miehlke S, Schmöcker C, Hartmann D, et al. Superiority of a split-dose regimen of sodium picosulfate/magnesium citrate (SPMC) in comparison to a prior-day schedule (AM/PM) for colonoscopy preparation. A randomized single-blinded study. Journal of Gastrointestinal and Liver Diseases. 2016;25(3):295‐302. doi: 10.15403/jgld.2014.1121.253.mag. | Irrelevant Topic |
| 1495 | Scott RG, Edwards JT, Fritschi L, Foster NM, Mendelson RM, Forbes GM. Community-based screening by colonoscopy or computed tomographic colonography in asymptomatic average-risk subjects. American Journal of Gastroenterology. 2004;99(6):1145‐1151. doi: 10.1111/j.1572-0241.2004.30253.x. | Irrelevant Topic |
| 1496 | Se YO, Chong IS, In KS, Dong IP, Mun SK, Tae WY, et al. Factors affecting the technical difficulty of colonoscopy. Hepato-Gastroenterology. 2007;54(77):1403-1406. | Irrelevant Topic |
| 1497 | Segarajasingam DS, Pawlik J, Forbes GM. Informed consent in direct access colonoscopy. J Gastroenterol Hepatol. 2007;22(12):2081-2085. doi: 10.1111/j.1440-1746.2007.04461.x. | Irrelevant Topic |
| 1498 | Segura JM, Molina E, Herrera A, Berges MA, Erdozain JC, Arjonilla A, et al. Hidrocolonic ultrasonography in the detection of tumoral processes in the inferior gastrointestinal tract. Rev Esp Enferm Dig. 1998;90(11):779-787. | Irrelevant Topic |
| 1499 | Selby W. Can clinical features predict the likelihood of finding abnormalities when using capsule endoscopy in patients with GI bleeding of obscure origin? Gastrointest Endosc. 2004;59(7):782-787. doi: 10.1016/s0016-5107(04)00168-3. | Irrelevant Topic |
| 1500 | Sengupta N. The role of colonoscopy and endotherapy in the management of lower gastrointestinal bleeding. Best Practice & Research Clinical Gastroenterology. 2019;42-43. doi: 10.1016/j.bpg.2019.05.002. | Irrelevant Topic |
| 1501 | Senore C, Correale L, Regge D, Hassan C, Iussich G, Silvani M, et al. Flexible sigmoidoscopy and CT colonography screening: patients' experience with and factors for undergoing screening-insight from the Proteus Colon Trial. Radiology. 2018;286(3):873-883. doi: 10.1148/radiol.2017170228. | Irrelevant Topic |
| 1502 | Senore C, Ederle A, Fantin A, Andreoni B, Bisanti L, Grazzini G, et al. Acceptability and side-effects of colonoscopy and sigmoidoscopy in a screening setting. Journal of Medical Screening. 2011;18(3):128‐134. doi: 10.1258/jms.2011.010135. | Irrelevant Topic |
| 1503 | Seo SI, Kang JG, Kim HS, Jang MK, Kim HY, Shin WG. Efficacy and tolerability of 2-L polyethylene glycol with ascorbic acid versus sodium picosulfate with magnesium citrate: a randomized controlled trial. Int J Colorectal Dis. 2018;33(5):541-548. doi: 10.1007/s00384-018-2989-7. | Irrelevant Topic |
| 1504 | Seoane A, Font X, Pérez JC, Pérez R, Enriquez CF, Parrilla M, et al. Evaluation of an educational telephone intervention strategy to improve non-screening colonoscopy attendance: a randomized controlled trial. World Journal of Gastroenterology. 2020;26(47):7568‐7583. doi: 10.3748/wjg.v26.i47.7568. | Irrelevant Topic |
| 1505 | Seol DC, Hong SN, Kim JH, Sung IK, Park HS, Lee JH, et al. Change in renal function after sodium phosphate preparation for screening colonoscopy. World J Gastroenterol. 2010;16(16):2010-2016. doi: 10.3748/wjg.v16.i16.2010. | Irrelevant Topic |
| 1506 | Sewitch MJ, Fallone CA, Ghali P, Lee GE. What patients want in a smartphone app that supports colonoscopy preparation: qualitative study to inform a user-centered smartphone app. Jmir Mhealth and Uhealth. 2019;7(7). doi: 10.2196/12242. | Irrelevant Topic |
| 1507 | Sey MSL, von Renteln D, Sultanian R, McDonald C, Martel M, Barkun A. Multicentre endoscopist-blinded randomised clinical trial to compare two bowel preparations after a colonoscopy with inadequate cleansing: a study protocol. Bmj Open. 2019;9(7). doi: 10.1136/bmjopen-2019-029573. | Irrelevant Topic |
| 1508 | Shafer LA, Walker JR, Waldman C, Yang C, Michaud V, Bernstein CN, et al. Factors associated with anxiety about colonoscopy: the preparation, the procedure, and the anticipated findings. Dig Dis Sci. 2018;63(3):610-618. doi: 10.1007/s10620-018-4912-z. | Irrelevant Topic |
| 1509 | Shanahan JJ, LeBlanc DM, Courage ER, Benesch MGK, Hickey KE, Hartwig KA, et al. Characteristics of interval colorectal cancer: a Canadian retrospective population-level analysis from Newfoundland and Labrador. Curr Oncol. 2022;29(12):9150-9162. doi: 10.3390/curroncol29120716. | Irrelevant Topic |
| 1510 | Shao Y, Shan X, Li S, Xu Y. Hypoglycemia during bowel preparation for colonoscopy in outpatients: a cross-sectional study. Gastroenterol Nurs. 2025;48(3):191-202. doi: 10.1097/sga.0000000000000868. | Irrelevant Topic |
| 1511 | Shavakhi A, Kianinia M, Torabi G, Nemati A, Saeidian B, Hoseinzadeh M, et al. High dose Senna or poly ethylene glycol (PEG) for elective colonoscopy preparation: a prospective randomized investigator-blinded clinical trial. Journal of research in medical sciences. 2011;16(2). | Irrelevant Topic |
| 1512 | Shi YH, Liu JL, Cheng CC, Li WL, Sun H, Zhou XL, et al. Construction and validation of machine learning-based predictive model for colorectal polyp recurrence one year after endoscopic mucosal resection. World Journal of Gastroenterology. 2025;31(11). doi: 10.3748/wjg.v31.i11.102387. | Irrelevant Topic |
| 1513 | Shin JG, Kim HW, Park SB, Choi CW, Kang DH, Kim SJ, et al. Polyp missing rate and its associated risk factors of referring hospitals for endoscopic resection of advanced colorectal neoplasia. Medicine. 2017;96(19). doi: 10.1097/MD.0000000000006742. | Irrelevant Topic |
| 1514 | Shin SY, Ga KS, Kim IY, Park YM, Jung DH, Kim JH, et al. Predictive factors for inadequate bowel preparation using low-volume polyethylene glycol (PEG) plus ascorbic acid for an outpatient colonoscopy. Sci Rep. 2019;9(1):19715. doi: 10.1038/s41598-019-56107-5. | Irrelevant Topic |
| 1515 | Shin SY, Park JJ, Ga KS, Kim IY, Park YM, Jung DH, et al. Predictive factors for inadequate bowel preparation using low-volume polyethylene glycol plus ascorbic acid for an outpatient colonoscopy: a single-center prospective observation study. J Gastroenterol Hepatol. 2018;33:249-249. | Irrelevant Topic |
| 1516 | Shweikeh F, Hong GR, Rogers K, Mouchli M, Hoscheit M, Lembo A. Colonoscopy utilization in elderly patients with dementia: characteristics, complications, and charges in a national matched-cohort analysis. Dig Dis Sci. 2024;69(5):1613-1625. doi: 10.1007/s10620-024-08363-3. | Irrelevant Topic |
| 1517 | Sica DA, Carl D, Zfass AM. Acute phosphate nephropathy--an emerging issue. Am J Gastroenterol. 2007;102(9):1844-1847. doi: 10.1111/j.1572-0241.2007.01047.x. | Irrelevant Topic |
| 1518 | Siddiki H, Ravi S, Al-Qaisi MT, Fath AR, Ramirez F, Crowell MD, et al. Comparison of small versus large volume split dose preparation for colonoscopy: a study of colonoscopy outcomes. Dig Dis Sci. 2018;63(9):2413-2418. doi: 10.1007/s10620-018-5102-8. | Irrelevant Topic |
| 1519 | Siddique DA, Jansson-Knodell CL, Gupta A, Howard G, Bohm ME, Siwiec RM, et al. Clinical presentation of small intestinal bacterial overgrowth from aerodigestive tract bacteria versus colonic-type bacteria: a comparison study. Dig Dis Sci. 2023;68(8):3390-3399. doi: 10.1007/s10620-023-07999-x. | Irrelevant Topic |
| 1520 | Siddiqui AA, Yang K, Spechler SJ, Cryer B, Davila R, Cipher D, et al. Duration of the interval between the completion of bowel preparation and the start of colonoscopy predicts bowel-preparation quality. Gastrointest Endosc. 2009;69(3 Pt 2):700-706. doi: 10.1016/j.gie.2008.09.047. | Irrelevant Topic |
| 1521 | Singh H, Kaita L, Taylor G, Nugent Z, Bernstein C. Practice and documentation of performance of colonoscopy in a central Canadian health region. Canadian Journal of Gastroenterology. 2014;28(4):185-190. doi: 10.1155/2014/635932. | Irrelevant Topic |
| 1522 | Singh H, Nugent Z, Demers AA, Bernstein CN. Rate and predictors of early/missed colorectal cancers after colonoscopy in Manitoba: a population-based study. Am J Gastroenterol. 2010;105(12):2588-2596. doi: 10.1038/ajg.2010.390. | Irrelevant Topic |
| 1523 | Singhatas P, Sangdee N, Kositchaiwat S, Sumboonanonda K, Suwanthanma W, Sumritpradit P, et al. Effect of bowel preparation with oral sodium phosphate on serum potassium level in patients undergoing colonoscopy under IV anesthesia. J Med Assoc Thai. 2009;92(12):1616-1620. | Irrelevant Topic |
| 1524 | Slee TM, Vleming LJ, Valentijn RM. Renal failure due to acute phosphate nephropathy. Neth J Med. 2008;66(10):438-441. | Irrelevant Topic |
| 1525 | Smith SG, von Wagner C, McGregor LM, Curtis LM, Wilson EA, Serper M, et al. The influence of health literacy on comprehension of a colonoscopy preparation information leaflet. Dis Colon Rectum. 2012;55(10):1074-1080. doi: 10.1097/DCR.0b013e31826359ac. | Irrelevant Topic |
| 1526 | Sninsky JA, Toups JV, Cotton CC, Peery AF, Arora S. An electronic medical record prediction model to identify inadequate bowel preparation in patients at outpatient colonoscopy. Tech Innov Gastrointest Endosc. 2024;26(2):130-137. doi: 10.1016/j.tige.2023.12.008. | Irrelevant Topic |
| 1527 | So H, Boo SJ, Seo H, Lee HS, Lee H, Park SH, et al. Patient descriptions of rectal effluents may help to predict the quality of bowel preparation with photographic examples. Intest Res. 2015;13(2):153-159. doi: 10.5217/ir.2015.13.2.153. | Irrelevant Topic |
| 1528 | Sofi AA, Nawras AT, Pai C, Samuels Q, Silverman AL. Lubiprostone plus PEG electrolytes versus placebo plus PEG electrolytes for outpatient colonoscopy preparation: a randomized, double-blind placebo-controlled trial. Am J Ther. 2015;22(2):105-110. doi: 10.1097/MJT.0b013e31826b7a1f. | Irrelevant Topic |
| 1529 | Song J, Li HL, Qi XF, Chen CX, Xu YM. Efficacy and safety of lubiprostone combined with polyethylene glycol electrolyte powder for bowel preparation in patients classified by risk level: a randomised trial. Front Oncol. 2025;15:1620794. doi: 10.3389/fonc.2025.1620794. | Irrelevant Topic |
| 1530 | Song JH, Kim ER. Strategies to improve screening colonoscopy quality for the prevention of colorectal cancer. Korean J Intern Med. 2024;39(4):547-554. doi: 10.3904/kjim.2023.334. | Irrelevant Topic |
| 1531 | Song YH, Xu RX, Zhang Y, Xing MX, Xu LD, Li KK, et al. Value of magnifying chromoendoscopy and magnifying optical enhancement technology in classifying colorectal polyps: a prospective controlled study. Gastroenterol Res Pract. 2021;2021. doi: 10.1155/2021/5533657. | Irrelevant Topic |
| 1532 | Sorser SA, Konanki V, Hursh A, Hagglund K, Lyons H. 1-day bowel preparation with polyethylene glycol 3350 is as effective and safe as a 3-day preparation for colonoscopy in children. BMC research notes. 2014;7:648. doi: 10.1186/1756-0500-7-648. | Irrelevant Topic |
| 1533 | Sosna J, Morrin MM, Copel L, Raptopoulos V, Kruskal JB. Computed tomography colonography (virtual colonoscopy): update on technique, applications, and future developments. Surg Technol Int. 2003;11:102-110. | Irrelevant Topic |
| 1534 | Stallmach A, Schmidt C, Watson A, Kiesslich R. An unmet medical need: advances in endoscopic imaging of colorectal neoplasia. J Biophotonics. 2011;4(7-8):482-489. doi: 10.1002/jbio.201100027. | Irrelevant Topic |
| 1535 | Steiner JF, Nguyen AP, Schuster KS, Goodrich G, Barrow J, Steiner CA, et al. Associations between missed colonoscopy appointments and multiple prior adherence behaviors in an integrated healthcare system: an observational study. J Gen Intern Med. 2024;39(1):36-44. doi: 10.1007/s11606-023-08355-5. | Irrelevant Topic |
| 1536 | Stewart BT, Keck JO, Duncan AV, Santamaria NM, Allen P. Difficult or incomplete flexible sigmoidoscopy: implications for a screening programme. Aust N Z J Surg. 1999;69(1):19-21. doi: 10.1046/j.1440-1622.1999.01499.x. | Irrelevant Topic |
| 1537 | Stolpman DR, Solem CA, Eastlick D, Adlis S, Shaw MJ. A randomized controlled trial comparing a low-residue diet versus clear liquids for colonoscopy preparation: impact on tolerance, procedure time, and adenoma detection rate. J Clin Gastroenterol. 2014;48(10):851‐855. doi: 10.1097/MCG.0000000000000167. | Irrelevant Topic |
| 1538 | Strate LL, Gralnek IM. ACG clinical guideline: management of patients with acute lower gastrointestinal bleeding. American Journal of Gastroenterology. 2016;111(4):459-+. doi: 10.1038/ajg.2016.41. | Irrelevant Topic |
| 1539 | Strauss AT, Yeh J, Martinez DA, Yenokyan G, Yoder J, Nehra R, et al. A patient-centered framework for health systems engineering in gastroenterology: improving inpatient colonoscopy bowel preparation. Bmc Gastroenterology. 2021;21(1). doi: 10.1186/s12876-021-01661-4. | Irrelevant Topic |
| 1540 | Subramaniam K, Ang PW, Neeman T, Fadia M, Taupin D. Post-colonoscopy colorectal cancers identified by probabilistic and deterministic linkage: results in an Australian prospective cohort. Bmj Open. 2019;9(6):e026138. doi: 10.1136/bmjopen-2018-026138. | Irrelevant Topic |
| 1541 | Sugrue C, Piggott R, Mahmood A, Khan W, Barry K, Waldron R. Understanding patient predictive factors of inadequate bowel preparation for colonoscopy: an observational prospective study. Irish Journal of Medical Science. 2012;181:S178-S178. | Irrelevant Topic |
| 1542 | Suki M, Abu Baker F, Pery S, Levin M, Nephrin S, Beshara A, et al. Effect of doctor's and assisting nurse's years of experience on polyp detection rate: a retrospective cohort. Israel Medical Association Journal. 2023;25(1):13-17. | Irrelevant Topic |
| 1543 | Sultan K, Trindade AJ. Concise commentary: is splitting the difference? Identifying risk factors associated with suboptimal bowel preparation for colonoscopy among IBD patients. Dig Dis Sci. 2022;67(10):4602-4603. doi: 10.1007/s10620-022-07533-5. | Irrelevant Topic |
| 1544 | Sun DL. Applying machine learning to predict bowel preparation adequacy in elderly patients for colonoscopy. J Gastroenterol Hepatol. 2024;39:197-197. | Irrelevant Topic |
| 1545 | Sung J, Ho KY, Chiu HM, Ching J, Travis S, Peled R. The use of Pillcam Colon in assessing mucosal inflammation in ulcerative colitis: a multicenter study. Endoscopy. 2012;44(8):754-758. doi: 10.1055/s-0032-1309819. | Irrelevant Topic |
| 1546 | Supan W, Ongiem A, Noitasaeng P, Bunnag P, Kaosombatwattana U, Supapueng O, et al. Incidence of complications in high-risk patients undergoing combined esophagogastroduodenoscopy and colonoscopy with total intravenous anesthesia: a prospective cohort study. Ther Clin Risk Manag. 2025;21:1395-1408. doi: 10.2147/tcrm.S541750. | Irrelevant Topic |
| 1547 | Sweetser S, Baron TH. Optimizing bowel cleansing for colonoscopy. Mayo Clin Proc. 2015;90(4):520-526. doi: 10.1016/j.mayocp.2015.01.015. | Irrelevant Topic |
| 1548 | Takahashi Y, Tanaka H, Kinjo M, Sakumoto K. Prospective evaluation of factors predicting difficulty and pain during sedation-free colonoscopy. Dis Colon Rectum. 2005;48(6):1295-1300. doi: 10.1007/s10350-004-0940-1. | Irrelevant Topic |
| 1549 | Takano R, Osawa S, Uotani T, Tani S, Ishida N, Tamura S, et al. Evaluating mucosal healing using colon capsule endoscopy predicts outcome in patients with ulcerative colitis in clinical remission. World J Clin Cases. 2018;6(15):952-960. doi: 10.12998/wjcc.v6.i15.952. | Irrelevant Topic |
| 1550 | Takeuchi Y, Inoue T, Hanaoka N, Higashino K, Iishi H, Chatani R, et al. Autofluorescence imaging with a transparent hood for detection of colorectal neoplasms: a prospective, randomized trial. Gastrointest Endosc. 2010;72(5):1006‐1013. doi: 10.1016/j.gie.2010.06.055. | Irrelevant Topic |
| 1551 | Talukdar R, Kamal A, Akshintala VS, Goud R, Lakhtakia S, Ramchandani MK, et al. Fluid type and volume reduce risk of post-ERCP pancreatitis and length of hospital stay in high-risk patients: a secondary analysis of the INDIEH trial. Endosc Int Open. 2020;8(7):E834‐E839. doi: 10.1055/a-1149-1359. | Irrelevant Topic |
| 1552 | Tamai N, Sumiyama K. Optimal bowel preparation for colonoscopy. Dig Endosc. 2025;37(2):139-146. doi: 10.1111/den.14914. | Irrelevant Topic |
| 1553 | Tang DM, Simmons K, Friedenberg FK. Anti-hypertensive therapy and risk factors associated with hypotension during colonoscopy under conscious sedation. J Gastrointestin Liver Dis. 2012;21(2):165-170. | Irrelevant Topic |
| 1554 | Tarhini H, Alrazim A, Ghusn W, Hosni M, Kerbage A, Soweid A, et al. Impact of sedation type on adenoma detection rate by colonoscopy. Clin Res Hepatol Gastroenterol. 2022;46(7):101981. doi: 10.1016/j.clinre.2022.101981. | Irrelevant Topic |
| 1555 | Taveira F, Areia M, Elvas L, Alves S, Brito D, Saraiva S, et al. A 3-day low-fibre diet does not improve colonoscopy preparation results compared to a 1-day diet: a randomized, single-blind, controlled trial. United European Gastroenterol J. 2019;7(10):1321-1329. doi: 10.1177/2050640619883176. | Irrelevant Topic |
| 1556 | Taylor SA, Bomanji JB, Manpanzure L, Robinson C, Groves AM, Dickson J, et al. Nonlaxative PET/CT colonography: feasibility, acceptability, and pilot performance in patients at higher risk of colonic neoplasia. J Nucl Med. 2010;51(6):854-861. doi: 10.2967/jnumed.109.072728. | Irrelevant Topic |
| 1557 | Tee HP, Corte C, Al-Ghamdi H, Prakoso E, Darke J, Chettiar R, et al. Prospective randomized controlled trial evaluating cap-assisted colonoscopy vs standard colonoscopy. World Journal of Gastroenterology. 2010;16(31):3905‐3910. doi: 10.3748/wjg.v16.i31.3905. | Irrelevant Topic |
| 1558 | Tekin YE, Günes A, Eren E. The relationship between e-health literacy and quality of bowel preparation and predictors in patients undergoing colonoscopy. Nurs Health Sci. 2025;27(4). doi: 10.1111/nhs.70255. | Irrelevant Topic |
| 1559 | Ten Hove JR, Shah SC, Shaffer SR, Bernstein CN, Castaneda D, Palmela C, et al. Consecutive negative findings on colonoscopy during surveillance predict a low risk of advanced neoplasia in patients with inflammatory bowel disease with long-standing colitis: results of a 15-year multicentre, multinational cohort study. Gut. 2019;68(4):615-622. doi: 10.1136/gutjnl-2017-315440. | Irrelevant Topic |
| 1560 | Teng TY, Khor SN, Kailasam M, Cheah WK, Lau CCL. Morning colonoscopies are associated with improved adenoma detection rates. Surgical Endoscopy and Other Interventional Techniques. 2016;30(5):1796-1803. doi: 10.1007/s00464-015-4448-7. | Irrelevant Topic |
| 1561 | Teramoto-Matsubara O, Lopez-Alvarenga JC, Vargas JA, Sobrino SR, Hernandez ME, Armienta R, et al. Predictive factors for efficacy and safety of a clean bowel preparation for screening colonoscopy in ambulatory patients. Gastrointest Endosc. 2009;69(5):AB226-AB226. doi: 10.1016/j.gie.2009.03.545. | Irrelevant Topic |
| 1562 | Theunissen F, Lantinga MA, ter Borg PCJ, Ouwendijk RJT, Siersema PD, Bruno MJ, et al. Efficacy of different bowel preparation regimen volumes for colorectal cancer screening and compliance with European Society of Gastrointestinal Endoscopy performance measures. United European Gastroenterology Journal. 2023;11(5):448-457. doi: 10.1002/ueg2.12386. | Irrelevant Topic |
| 1563 | Thorndal C, Skau B, Al-Najami I, Kaalby L, Baatrup G, Koulaouzidis A, et al. Selected comorbidities increases the likelihood of an incomplete colonoscopy during colorectal cancer screening. Scand J Gastroenterol. 2025;60(4):283-291. doi: 10.1080/00365521.2025.2465623. | Irrelevant Topic |
| 1564 | Thornton JG, Morris AM, Thornton JD, Flowers CR, McCashland TM. Racial variation in colorectal polyp and tumor location. J Natl Med Assoc. 2007;99(7):723-728. | Irrelevant Topic |
| 1565 | Tomer O, Shapira Y, Kriger-Sharabi O, Mawasi N, Melzer E, Epshtein J, et al. An Israeli national survey on ischemic colitis induced by pre-colonoscopy bowel preparation (R1). Acta Gastroenterol Belg. 2022;85(1):94-96. doi: 10.51821/88.1.8676. | Irrelevant Topic |
| 1566 | Tontini GE, Rimondi A, Pessarelli T, Ciprandi G, Kurihara H, Sorge A, et al. Clinical features and risk factors for colorectal gas explosion during digestive endoscopy and surgery: a systematic review. Surgical Endoscopy and Other Interventional Techniques. 2025;39(1):384-393. doi: 10.1007/s00464-024-11370-0. | Irrelevant Topic |
| 1567 | Triantafyllou K, Gkolfakis P, Skamnelos A, Diamantopoulou G, Dagas A, Tziatzios G, et al. Impact of simple, specific, verbal instructions on the quality of bowel preparation in hospitalized patients undergoing colonoscopy: a multicenter randomized controlled trial. Endosc Int Open. 2021;9(3):E378‐E387. doi: 10.1055/a-1339-0913. | Irrelevant Topic |
| 1568 | Triantafyllou K, Tziatzios G, Sioulas AD, Beintaris I, Gouloumi AR, Panayiotides IG, et al. Diagnostic yield of scope retroflexion in the right colon: a prospective cohort study. Digestive and Liver Disease. 2016;48(2):176-181. doi: 10.1016/j.dld.2015.11.024. | Irrelevant Topic |
| 1569 | Troelsen FS, Sørensen HT, Pedersen L, Erichsen R. Risk of a post-colonoscopy colorectal cancer in patients with type 2 diabetes: a Danish population-based cohort study. BMJ Open Gastroenterol. 2021;8(1). doi: 10.1136/bmjgast-2021-000786. | Irrelevant Topic |
| 1570 | Tseng CW, Hsieh YH, Koo M, Leung FW. Comparing right colon adenoma detection rate during water exchange and air insufflation: a double-blind randomized controlled trial. Techniques in Coloproctology. 2022;26(1):35-44. doi: 10.1007/s10151-021-02537-1. | Irrelevant Topic |
| 1571 | Tsunashima H, Sato K, Onda T, Kuniyoshi N, Matsumoto K, Sekine K, et al. An evaluation of predictive factors for bowel preparation before colonoscopy using the Boston Bowel Preparation Scale. J Gastroenterol Hepatol. 2018;33:244-244. | Irrelevant Topic |
| 1572 | Tudyka V, Blomqvist L, Beets-Tan RGH, Boelens PG, Valentini V, Van De Velde CJ, et al. EURECCA consensus conference highlights about colon & rectal cancer multidisciplinary management: the radiology experts review. European Journal of Surgical Oncology. 2014;40(4):469-475. doi: 10.1016/j.ejso.2013.10.029. | Irrelevant Topic |
| 1573 | Tumino E, Sacco R, Bertini M, Bertoni M, Parisi G, Capria A. Endotics system vs colonoscopy for the detection of polyps. World Journal of Gastroenterology. 2010;16(43):5452‐5456. doi: 10.3748/wjg.v16.i43.5452. | Irrelevant Topic |
| 1574 | Ukwade D, Hernandez LJ, Modi Z, Raza HS, Siegel M, Nwachukwu D, et al. GLP-1 receptor agonist increase retained gastric contents on EGD and same-day colonoscopy reduces this risk. Front Med (Lausanne). 2025;12:1638981. doi: 10.3389/fmed.2025.1638981. | Irrelevant Topic |
| 1575 | Underwood JA, Bekal P, Levitan O, Burleson DB, Johnson DA. Hygieacare prep for colonoscopy consistently provides 97% adequacy, and is agnostic to predictors of poor oral bowel prep. Gastrointest Endosc. 2020;91(6):AB573-AB574. | Irrelevant Topic |
| 1576 | Unger RZ, Amstutz SP, Seo DH, Huffman M, Rex DK. Willingness to undergo split-dose bowel preparation for colonoscopy and compliance with split-dose instructions. Dig Dis Sci. 2010;55(7):2030-2034. doi: 10.1007/s10620-009-1092-x. | Irrelevant Topic |
| 1577 | Utano K, Aizawa M, Isohata N, Utano Y, Endo S, Togashi K. The potential of CT colonography for colorectal cancer screening in Japan. Jpn J Radiol. 2025;43(9):1417-1426. doi: 10.1007/s11604-025-01798-2. | Irrelevant Topic |
| 1578 | Valciukiene J, Lastauskiene E, Laurinaviciene A, Jakubauskas M, Kryzauskas M, Valkiuniene RB, et al. Interaction of human gut microbiota and local immune system in progression of colorectal adenoma (MIMICA-1): a protocol for a prospective, observational cohort study. Front Oncol. 2025;14. doi: 10.3389/fonc.2024.1495635. | Irrelevant Topic |
| 1579 | Vale Rodrigues R, Claro I, Lage P, Rosa I, Ferreira S, Pereira da Silva J, et al. Colorectal cancer surveillance in Portuguese families with Lynch syndrome: a cohort study. Int J Colorectal Dis. 2018;33(6):695-702. doi: 10.1007/s00384-018-3002-1. | Irrelevant Topic |
| 1580 | van Enckevort CCG, de Graaf APJ, Hollema H, Sluiter WJ, Kleibeuker JH, Koornstra JJ. Predictors of colorectal neoplasia after polypectomy: based on initial and consecutive findings. Netherlands Journal of Medicine. 2014;72(3):139-145. | Irrelevant Topic |
| 1581 | van Heijningen EMB, Lansdorp-Vogelaar I, Kuipers EJ, Dekker E, Lesterhuis W, Ter Borg F, et al. Features of adenoma and colonoscopy associated with recurrent colorectal neoplasia based on a large community-based study. Gastroenterology. 2013;144(7):1410-1418. doi: 10.1053/j.gastro.2013.03.002. | Irrelevant Topic |
| 1582 | van Riswijk MLM, van Keulen KE, Neumann H, Siersema PD. An intraprocedural bowel cleansing system for difficult-to-prepare patients-a multicenter prospective feasibility study. United European Gastroenterol J. 2024;12(1):56-65. doi: 10.1002/ueg2.12501. | Irrelevant Topic |
| 1583 | van Riswijk MLM, van Keulen KE, Siersema PD. Efficacy of ultra-low volume (≤1 L) bowel preparation fluids: systematic review and meta-analysis. Digestive Endoscopy. 2022;34(1):13-32. doi: 10.1111/den.14015. | Irrelevant Topic |
| 1584 | van Tuyl SA, den Ouden H, Stolk MF, Kuipers EJ. Optimal preparation for video capsule endoscopy: a prospective, randomized, single-blind study. Endoscopy. 2007;39(12):1037‐1040. doi: 10.1055/s-2007-966988. | Irrelevant Topic |
| 1585 | Varughese S, Kumar AR, George A, Castro FJ. Morning-only one-gallon polyethylene glycol improves bowel cleansing for afternoon colonoscopies: a randomized endoscopist-blinded prospective study. American Journal of Gastroenterology. 2010;105(11):2368-2374. doi: 10.1038/ajg.2010.271. | Irrelevant Topic |
| 1586 | Veldhuijzen G, van Esch AA, Klemt-Kropp M, Terhaar Sive Droste JS, Drenth JPH. E-patient counseling trial (E-PACO): computer based education versus nurse counseling for patients to prepare for colonoscopy. J Vis Exp. 2019;(150). doi: 10.3791/58798. | Irrelevant Topic |
| 1587 | Verma S, Fogel J, Beyda DJ, Bernstein B, Notar-Francesco V, Mohanty SR. Chronic methadone use, poor bowel visualization and failed colonoscopy: a preliminary study. World J Gastroenterol. 2012;18(32):4350-4356. doi: 10.3748/wjg.v18.i32.4350. | Irrelevant Topic |
| 1588 | Voiosu T, Ratiu I, Voiosu A, Iordache T, Schipor A, Baicus C, et al. Time for individualized colonoscopy bowel-prep regimens? A randomized controlled trial comparing sodium picosulphate and magnesium citrate versus 4-liter split-dose polyethylene glycol. Journal of Gastrointestinal and Liver Diseases. 2013;22(2):129‐134. | Irrelevant Topic |
| 1589 | Voiosu T, Tanţău A, Voiosu A, Benguş A, Mocanu C, Smarandache B, et al. Preparation regimen is more important than patient-related factors: a randomized trial comparing a standard bowel preparation before colonoscopy with an individualized approach. Rom J Intern Med. 2017;55(1):36-43. doi: 10.1515/rjim-2016-0047. | Irrelevant Topic |
| 1590 | von Atzingen AC, Tiferes DA, Deak E, Matos D, D'Ippolito G. Using computed tomography colonography in patients at high risk of colorectal cancer - a prospective study in a university hospital in South America. Clinics (Sao Paulo). 2014;69(11):723-730. doi: 10.6061/clinics/2014(11)03. | Irrelevant Topic |
| 1591 | Vos FM, Tielbeek JA, Naziroglu RE, Li Z, Schueffler P, Mahapatra D, et al. Computational modeling for assessment of IBD: to be or not to be? Annu Int Conf IEEE Eng Med Biol Soc. 2012;2012:3974-3977. doi: 10.1109/embc.2012.6346837. | Irrelevant Topic |
| 1592 | Voska M, Zavoral M, Grega T, Majek O, Martinek J, Tacheci I, et al. Accuracy of colon capsule endoscopy for colorectal neoplasia detection in individuals referred for a screening colonoscopy. Gastroenterol Res Pract. 2019;2019. doi: 10.1155/2019/5975438. | Irrelevant Topic |
| 1593 | Wadhwa V, Jobanputra Y, Al Taii H, Thota PN, Lopez R, Gurudu SR, et al. Proposal of high-risk adenoma detection rate as an impactful, complementary quality indicator of colonoscopy. Surg Endosc. 2020;34(1):325-331. doi: 10.1007/s00464-019-06770-6. | Irrelevant Topic |
| 1594 | Wagner MS, Burgess J, Britt RC. Barriers to colonoscopy in an uninsured patient population-a quality improvement project. Am Surg. 2019;85(1):111-114. | Irrelevant Topic |
| 1595 | Wanders LK, van Doorn SC, Fockens P, Dekker E. Quality of colonoscopy and advances in detection of colorectal lesions: a current overview. Expert Review of Gastroenterology & Hepatology. 2015;9(4):417-430. doi: 10.1586/17474124.2015.972940. | Irrelevant Topic |
| 1596 | Wang BB, Hu L, Hu XY, Han D, Wu J. Risk factors associated with hypokalemia during postanesthesia recovery and its impact on outcomes in gynecological patients: a propensity score matching study. Current Medical Science. 2024;44(2):261-272. doi: 10.1007/s11596-024-2848-4. | Irrelevant Topic |
| 1597 | Wang CL, Huang ZP, Chen K, Yan FH, Zhu LL, Shan YQ, et al. Adenoma miss rate determined by very shortly repeated colonoscopy: retrospective analysis of data from a single tertiary medical center in China. Medicine (Baltimore). 2018;97(38):e12297. doi: 10.1097/md.0000000000012297. | Irrelevant Topic |
| 1598 | Wang D, Zhang SY, Jiang YN, Ren YT, Kuai DY, Zhao RH, et al. Correlation between colonoscopy difficulty and personality traits: study protocol for a prospective, observational, multicentre study. Bmj Open. 2024;14(12). doi: 10.1136/bmjopen-2024-090606. | Irrelevant Topic |
| 1599 | Wang DC, Wang AX, Ding XW, Zhou R. Combined water exchange and cap-assisted colonoscopy reduces cecal intubation time and patient discomfort in patients with unsedated colonoscopy: a randomized controlled trial. Bmc Gastroenterology. 2025;26(1). doi: 10.1186/s12876-025-04557-9. | Irrelevant Topic |
| 1600 | Wang F, Huang XX, Wang ZJ, Yan ZW, Wang SL, Pan P, et al. One-day versus three-day low-residue diet bowel preparation regimens before colonoscopy: a meta-analysis of randomized controlled trials. J Gastroenterol Hepatol. 2024;39(5):787-795. doi: 10.1111/jgh.16466. | Irrelevant Topic |
| 1601 | Wang LL, Zhang Y, Li JY, Ran Y, Wang XH, Ma XQ, et al. Efficacy of polyethylene glycol electrolyte powder combined with linaclotide for colon cleansing in patients with chronic constipation undergoing colonoscopy: a multicenter, single-blinded, randomized controlled trial. Clin Transl Gastroenterol. 2024;15(6). doi: 10.14309/ctg.0000000000000708. | Irrelevant Topic |
| 1602 | Wang Q, Shen KX, Fei BY, Luo H, Li RQ, Wang ZM, et al. A predictive model for early death in elderly colorectal cancer patients: a population-based study. Front Oncol. 2023;13. doi: 10.3389/fonc.2023.1278137. | Irrelevant Topic |
| 1603 | Wang SL, Wang Q, Yao J, Zhao SB, Wang LS, Li ZS, et al. Effect of WeChat and short message service on bowel preparation: an endoscopist-blinded, randomized controlled trial. European journal of gastroenterology & hepatology. 2019;31(2):170‐177. doi: 10.1097/MEG.0000000000001303. | Irrelevant Topic |
| 1604 | Wang XB, Guo HY, Tang Y, Chen L, Wang XF. Establishment and evaluation of a nomogram predicting risks of missed diagnoses of colorectal polyps. Bmc Gastroenterology. 2022;22(1). doi: 10.1186/s12876-022-02415-6. | Irrelevant Topic |
| 1605 | Wang YP, Jheng YC, Sung KY, Lin HE, Hsin IF, Chen PH, et al. Use of U-Net convolutional neural networks for automated segmentation of fecal material for objective evaluation of bowel preparation quality in colonoscopy. Diagnostics. 2022;12(3). doi: 10.3390/diagnostics12030613. | Irrelevant Topic |
| 1606 | Wang YR, Cangemi JR, Loftus EV, Jr., Picco MF. Increased odds of interval left-sided colorectal cancer after flexible sigmoidoscopy compared with colonoscopy in older patients in the United States: a population-based analysis of the SEER-Medicare linked database, 2001-2005. Mayo Clin Proc. 2013;88(5):471-478. doi: 10.1016/j.mayocp.2013.02.010. | Irrelevant Topic |
| 1607 | Watanabe S, Sato A, Kobayashi K, Miyakawa A, Uchida H, Machida T, et al. Colonoscopic observation time as a predictor of stigmata of recent hemorrhage identification in colonic diverticular hemorrhage. Scand J Gastroenterol. 2023;58(3):304-309. doi: 10.1080/00365521.2022.2121939. | Irrelevant Topic |
| 1608 | Wehrmann T, Riphaus A. Sedation, surveillance, and preparation. Endoscopy. 2009;41(1):86‐90. doi: 10.1055/s-2008-1077781. | Irrelevant Topic |
| 1609 | Weingarden AR, Treiger O, Ulsh L, Limketkai B, Goldenberg D, Okafor P, et al. Delivery of fecal material to terminal ileum is associated with long-term success of fecal microbiota transplantation. Dig Dis Sci. 2023;68(5):2006-2014. doi: 10.1007/s10620-022-07761-9. | Irrelevant Topic |
| 1610 | Weir MA, Fleet JL, Vinden C, Shariff SZ, Liu K, Song H, et al. Hyponatremia and sodium picosulfate bowel preparations in older adults. Am J Gastroenterol. 2014;109(5):686-694. doi: 10.1038/ajg.2014.20. | Irrelevant Topic |
| 1611 | Weiss JB, Cetel NS, Weiss DE. Colonoscopy has disadvantages. Cleveland Clinic Journal of Medicine. 2019;86(12):768-770. doi: 10.3949/ccjm.86c.12002. | Irrelevant Topic |
| 1612 | Wells CD, Heigh RI, Sharma VK, Crowell MD, Gurudu SR, Leighton JA, et al. Comparison of morning versus afternoon cecal intubation rates. Bmc Gastroenterology. 2007;7. doi: 10.1186/1471-230X-7-19. | Irrelevant Topic |
| 1613 | Wen S, He L, Zhao X, Li Y, Lin X, Fu Z, et al. Risk factors and prediction model for delayed bleeding after cold snare polypectomy: a retrospective study. Int J Colorectal Dis. 2024;39(1). doi: 10.1007/s00384-024-04687-8. | Irrelevant Topic |
| 1614 | Westerhof J, Weersma RK, Koornstra JJ. Risk factors for incomplete small-bowel capsule endoscopy. Gastrointest Endosc. 2009;69(1):74-80. doi: 10.1016/j.gie.2008.04.034. | Irrelevant Topic |
| 1615 | Wexner SD, Beck DE, Baron TH, Fanelli RD, Hyman N, Shen B, et al. A consensus document on bowel preparation before colonoscopy: prepared by a Task Force from the American Society of Colon and Rectal Surgeons (ASCRS), the American Society for Gastrointestinal Endoscopy (ASGE), and the Society of American Gastrointestinal and Endoscopic Surgeons (SAGES). Surg Endosc. 2006;20(7):1161. doi: 10.1007/s00464-006-3037-1. | Irrelevant Topic |
| 1616 | White TJ, Avery GR, Kennan N, Syed AM, Hartley JE, Monson JR. Virtual colonoscopy vs conventional colonoscopy in patients at high risk of colorectal cancer--a prospective trial of 150 patients. Colorectal Dis. 2009;11(2):138-145. doi: 10.1111/j.1463-1318.2008.01554.x. | Irrelevant Topic |
| 1617 | Wi JY, Kim SH, Lee JY, Kim SG, Han JK, Choi BI. Electronic cleansing for CT colonography: does it help CAD software performance in a high-risk population for colorectal cancer? Eur Radiol. 2010;20(8):1905-1916. doi: 10.1007/s00330-010-1765-z. | Irrelevant Topic |
| 1618 | Wieszczy P, Kaminski MF, Franczyk R, Loberg M, Kobiela J, Rupinska M, et al. Colorectal cancer incidence and mortality after removal of adenomas during screening colonoscopies. Gastroenterology. 2020;158(4):875-883.e875. doi: 10.1053/j.gastro.2019.09.011. | Irrelevant Topic |
| 1619 | Wijnands AM, Te Groen M, Peters Y, Kaptein AA, Oldenburg B, Hoentjen F, et al. Patients prioritize a low-volume bowel preparation in colitis-associated colorectal cancer surveillance: a discrete choice experiment. Inflamm Bowel Dis. 2022;28(7):1053-1060. doi: 10.1093/ibd/izab221. | Irrelevant Topic |
| 1620 | Williams H, Dierick NR, Lee C, Sundaralingam P, Kostalas SN. Determinants of high sessile serrated lesion detection: role of faecal occult blood test and colonoscopy quality indicators. World J Gastrointest Endosc. 2025;17(8):109176. doi: 10.4253/wjge.v17.i8.109176. | Irrelevant Topic |
| 1621 | Windpessl M, Schwarz C, Wallner M. "Bowel prep hyponatremia" - a state of acute water intoxication facilitated by low dietary solute intake: case report and literature review. BMC Nephrol. 2017;18(1):54. doi: 10.1186/s12882-017-0464-2. | Irrelevant Topic |
| 1622 | Winrich E, Flaherty DT, Eisa M, Poddar T, Pooler A, Kong MY, et al. Racial and economic risk factors for inadequate bowel preparation prior to colonoscopy. Gastroenterology. 2022;162(7):S469-S469. | Irrelevant Topic |
| 1623 | Wolf HJ, Dwyer A, Ahnen DJ, Pray SL, Rein SM, Morwood KD, et al. Colon cancer screening for Colorado's underserved: a community clinic/academic partnership. Am J Prev Med. 2015;48(3):264-270. doi: 10.1016/j.amepre.2014.09.016. | Irrelevant Topic |
| 1624 | Wong MCS, Ching JYL, Chan VCW, Lam TYT, Luk AKC, Tang RSY, et al. Determinants of bowel preparation quality and its association with adenoma detection: a prospective colonoscopy study. Medicine (Baltimore). 2016;95(2):e2251. doi: 10.1097/md.0000000000002251. | Irrelevant Topic |
| 1625 | Wong MCS, Leung EYM, Chun SCC, Wang HHX, Huang J. Prediction of advanced colorectal neoplasia based on metabolic parameters among symptomatic patients. Journal of Gastroenterology and Hepatology (Australia). 2023;38(9):1576-1586. doi: 10.1111/jgh.16271. | Irrelevant Topic |
| 1626 | Woo YM, Crail S, Curry G, Geddes CC. A life threatening complication after ingestion of sodium phosphate bowel preparation. Bmj. 2006;333(7568):589-590. doi: 10.1136/bmj.333.7568.589. | Irrelevant Topic |
| 1627 | Wu KL, Rayner CK, Chuah SK, Chiu KW, Lu CC, Chiu YC. Impact of low-residue diet on bowel preparation for colonoscopy. Dis Colon Rectum. 2011;54(1):107-112. doi: 10.1007/DCR.0b013e3181fb1e52. | Irrelevant Topic |
| 1628 | Xiang L, Zhan Q, Zhao XH, Wang YD, An SL, Xu YZ, et al. Risk factors associated with missed colorectal flat adenoma: a multicenter retrospective tandem colonoscopy study. World J Gastroenterol. 2014;20(31):10927-10937. doi: 10.3748/wjg.v20.i31.10927. | Irrelevant Topic |
| 1629 | Xu HX, He Z, Liu YL, Xu H, Liu PF. Application of linaclotide in bowel preparation for colonoscopy in patients with constipation: a prospective randomized controlled study. J Gastroenterol Hepatol. 2024;39(12):2752-2759. doi: 10.1111/jgh.16734. | Irrelevant Topic |
| 1630 | Xu Q, He Z. Effect of different working periods on missed diagnosis of colorectal polyps in colonoscopy. BMC Gastroenterol. 2024;24(1):286. doi: 10.1186/s12876-024-03365-x. | Irrelevant Topic |
| 1631 | Xu QQ, Tu H, Xiang SJ, Tan QH, Wang X. The effect of intravenous infusion on the rapid recovery of elderly patients treated with painless colonoscopy and the value of ultrasonic measurement of the inferior vena cava diameter in guiding intravenous infusion. Annals of Palliative Medicine. 2021;10(1):61-73. doi: 10.21037/apm-20-2217. | Irrelevant Topic |
| 1632 | Xu Y, Ou S, Yuan X, Yao J, Yang T. Construction and validation of risk scoring model for inadequate bowel preparation in elderly patients undergoing colonoscopy. Therapeutics and Clinical Risk Management. 2025;21:885-894. doi: 10.2147/TCRM.S503389. | Irrelevant Topic |
| 1633 | Xue JH, Xie YH, Zou TH, Qian Y, Kang ZR, Zhou CB, et al. Fecal Fusobacterium nucleatum as a predictor for metachronous colorectal adenoma after endoscopic polypectomy. Journal of Gastroenterology and Hepatology (Australia). 2021;36(10):2841-2849. doi: 10.1111/jgh.15559. | Irrelevant Topic |
| 1634 | Yadlapati R, Gluskin AB, Gregory DL, Johnston ER, Ciolino JD, Keswani RN. Sa1067 black inpatients are at higher risk for inadequate inpatient bowel preparation... 2016 DDW (Digestive Disease Week) ASGE (American Society for Gastrointestinal Endoscopy) Program and Abstracts 21 May 2016-24 May 2016, San Diego, California. Gastrointest Endosc. 2016;83:AB223-AB223. doi: 10.1016/j.gie.2016.03.290. | Irrelevant Topic |
| 1635 | Yamada A, Kanda T, Sugimoto T. A case of severe acute hyponatremia after colonoscopy with polyethylene glycol plus ascorbic acid bowel preparation. CEN Case Rep. 2023;12(2):254-258. doi: 10.1007/s13730-022-00760-9. | Irrelevant Topic |
| 1636 | Yamamoto H, Yano T, Araki A, Esaki M, Ohtsuka K, Ohmiya N, et al. Guidelines for endoscopic balloon dilation in treating Crohn's disease-associated small intestinal strictures (supplement to the Clinical Practice Guidelines for Enteroscopy). Digestive Endoscopy. 2022;34(7):1278-1296. doi: 10.1111/den.14429. | Irrelevant Topic |
| 1637 | Yan H, Huang H, Yang D, Chen Z, Liu C, Huang Z, et al. 3 L split-dose polyethylene glycol is superior to 2 L polyethylene glycol in colonoscopic bowel preparation in relatively high-BMI (≥ 24 kg/m2) individuals: a multicenter randomized controlled trial. Bmc Gastroenterology. 2023;23(1):427. doi: 10.1186/s12876-023-03068-9. | Irrelevant Topic |
| 1638 | Yang C, Sriranjan V, Abou-Setta AM, Poluha W, Walker JR, Singh H. Anxiety associated with colonoscopy and flexible sigmoidoscopy: a systematic review. Am J Gastroenterol. 2018;113(12):1810-1818. doi: 10.1038/s41395-018-0398-8. | Irrelevant Topic |
| 1639 | Yang D, Hillman SL, Harris AM, Sinicrope PS, Devens ME, Ahlquist DA. Patient perceptions of stool DNA testing for pan-digestive cancer screening: a survey questionnaire. World Journal of Gastroenterology. 2014;20(17):4972-4979. doi: 10.3748/wjg.v20.i17.4972. | Irrelevant Topic |
| 1640 | Yang K, Siddiqui A, Spechler SJ, Cryer BI, Davila RE, Harford W. Duration of the interval between completion of the bowel preparation and start of the colonoscopy as a better predictor of bowel preparation quality than colonoscopy start time alone. Gastrointest Endosc. 2008;67(5):AB76-AB76. doi: 10.1016/j.gie.2008.03.020. | Irrelevant Topic |
| 1641 | Yao RBC, Gala KS, Ghusn W, Abboud DM, Wallace FK, Vargas EJ. Effect of glucagon-like peptide-1 receptor agonists on bowel preparation for colonoscopy. American Journal of Gastroenterology. 2024;119(6):1154-1157. doi: 10.14309/ajg.0000000000002564. | Irrelevant Topic |
| 1642 | Yokota M, Muto J, Hashida K, Nagahisa Y, Okabe M, Kitagawa H, et al. The necessity of intensive surveillance colonoscopy for patients with a remaining right colon after resection of colorectal cancer: a retrospective cohort study. Surgery Today. 2022;52(3):502-509. doi: 10.1007/s00595-021-02372-9. | Irrelevant Topic |
| 1643 | Yoo IK, Jeen YT, Choi SJ, Choi HS, Keum B, Kim ES, et al. Evaluation of bowel preparation quality in patients with a history of colorectal resection. Turk J Gastroenterol. 2019;30(3):278-283. doi: 10.5152/tjg.2018.17517. | Irrelevant Topic |
| 1644 | Yumuşak Ergin G, Ergin M, Özçelik M. Incidence and risk factors of orthostatic hypotension and postural tachycardia following sedated colonoscopy: a prospective observational study. Diagnostics (Basel). 2025;15(23). doi: 10.3390/diagnostics15233009. | Irrelevant Topic |
| 1645 | Yurgelun MB. Next-generation strategies for hereditary colorectal cancer risk assessment. Journal of Clinical Oncology. 2015;33(5):388-U321. doi: 10.1200/JCO.2014.58.9895. | Irrelevant Topic |
| 1646 | Zad M, Do C, Johnston L, Alansari M. Duration between the completion of bowel preparation and the start of colonoscopy predicts bowel preparation quality. J Gastroenterol Hepatol. 2018;33:257-257. | Irrelevant Topic |
| 1647 | Zakko L, Visrodia K, Allen J, Chan DK, Lutzke LS, Wang KK. Can visual inspection of colonic effluent by healthcare providers predict adequacy of bowel preparation for effective colonoscopy? Gastrointest Endosc. 2017;85(5):AB166-AB166. doi: 10.1016/j.gie.2017.03.362. | Irrelevant Topic |
| 1648 | Zakko L, Visrodia K, Allen J, Clemens M, Lutzke L, Wang KK. No stool out, then it's still in: inability to evacuate stool immediately prior to colonoscopy is a predictor for inadequate bowel preparation. American Journal of Gastroenterology. 2017;112:S263-S263. doi: 10.14309/00000434-201710001-00500. | Irrelevant Topic |
| 1649 | Zalis ME, Perumpillichira JJ, Magee C, Kohlberg G, Hahn PF. Tagging-based, electronically cleansed CT colonography: evaluation of patient comfort and image readability. Radiology. 2006;239(1):149-159. doi: 10.1148/radiol.2383041308. | Irrelevant Topic |
| 1650 | Zeng L, Chua EG, Xiong Y, Ding S, Ai H, Hou Z, et al. Actual and estimated adenoma detection rates: a 2-year monocentric colonoscopic screening outcome in Shenzhen, China. JGH Open. 2020;4(4):707-712. doi: 10.1002/jgh3.12322. | Irrelevant Topic |
| 1651 | Zhai CY, Huang QY, Chai NL, Zhang WG, Linghu EQ. Prediction of inadequate bowel preparation using total and segmental colon transit time in patients with chronic constipation: some different outcomes. Gastroenterol Res Pract. 2019;2019. doi: 10.1155/2019/2328054. | Irrelevant Topic |
| 1652 | Zhan Q, Xiang L, Zhao X, An S, Wang Y, Xu Y, et al. A novel quality scoring system for the evaluation of individual colonoscopy: a multicenter retrospective study. J Gastroenterol Hepatol. 2016;31(1):172-179. doi: 10.1111/jgh.13031. | Irrelevant Topic |
| 1653 | Zhang C, Chen X, Tang B, Shan J, Qin J, He J, et al. A novel ultra-low-volume regimen combining 1 L polyethylene glycol and linaclotide versus 2 L polyethylene glycol for colonoscopy cleansing in low-risk individuals: a randomized controlled trial. Gastrointest Endosc. 2023;97(5):952‐961.e951. doi: 10.1016/j.gie.2022.12.015. | Irrelevant Topic |
| 1654 | Zhang J, Xiao Y, Yin A, Cao Z, Li J, Liu S, et al. Lactulose combined with polyethylene glycol for bowel preparation in patients of different risks: single-center prospective randomized controlled trial. Chinese Journal of Digestive Endoscopy. 2021;38(12):980‐984. doi: 10.3760/cma.j.cn321463-20210412-00240. | Irrelevant Topic |
| 1655 | Zhang YY, Ding CY, Li J, Hu XY, Wang YF, Tang W. Impact of prepackaged low-residue diet on bowel preparation for colonoscopy: a meta-analysis. Gastroenterology Nursing. 2021;44(2):E29-E37. doi: 10.1097/SGA.0000000000000588. | Irrelevant Topic |
| 1656 | Zhang YY, Vimala R, Chui PL, Hilmi IN. Clinical trial: effect of abdominal vibration combined with walking exercise programme on bowel preparation in older patients with constipation. Aliment Pharmacol Ther. 2025;61(9):1447-1457. doi: 10.1111/apt.70103. | Irrelevant Topic |
| 1657 | Zhang Z, Chen XJ, Cao TT, Ning YM, Wang HZ, Wang F, et al. Polyps are detected more often in early colonoscopies. Scandinavian Journal of Gastroenterology. 2023;58(9):1085-1090. doi: 10.1080/00365521.2023.2202293. | Irrelevant Topic |
| 1658 | Zhao S, Wang S, Pan P, Xia T, Chang X, Yang X, et al. Magnitude, risk factors, and factors associated with adenoma miss rate of tandem colonoscopy: a systematic review and meta-analysis. Gastroenterology. 2019;156(6):1661-1674.e1611. doi: 10.1053/j.gastro.2019.01.260. | Irrelevant Topic |
| 1659 | Zhao Y, Xie F, Bai X, Yang A, Wu D. Educational virtual reality videos in improving bowel preparation quality and satisfaction of outpatients undergoing colonoscopy: protocol of a randomised controlled trial. Bmj Open. 2019;9(8):e029483. doi: 10.1136/bmjopen-2019-029483. | Irrelevant Topic |
| 1660 | Zhou HB, Zhang XF, Yang JF. Correlation of cleanliness among different bowel segments during colonoscopy: a retrospective study. Gastroenterol Res Pract. 2020;2020. doi: 10.1155/2020/5363827. | Irrelevant Topic |
| 1661 | Zhou MRJ, Lebwohl B, Krigel A. Patient and physician factors associated with adenoma and sessile serrated lesion detection rates. Dig Dis Sci. 2020;65(11):3123-3131. doi: 10.1007/s10620-020-06419-8. | Irrelevant Topic |
| 1662 | Liu JY, Jin CY, Lai LC, et al. Construction and validation of a nomogram prediction model for the risk of bowel preparation failure in hospitalized patients undergoing colonoscopy. Chinese Evidence-Based Nursing. 2024;10(13):2343–2349. doi: 10.12102/j.issn.2095-8668.2024.13.012. | The population does not match |

**S8 Table 2.** Full-text articles excluded with reasons (n = 108)

| **No.** | **Reference** | **Reason for exclusion** |
| --- | --- | --- |
| 1 | Bai XD, Lu Q, Liu JK. Analysis of bowel cleaning status and its influencing factors in patients undergoing colonoscopy in a tertiary hospital in Beijing[J]. Chinese Journal of Modern Nursing, 2018, 24(23): 2752-2756. doi: 10.3760/cma.j.issn.1674-2907.2018.23.006. | Analysis of risk factors only |
| 2 | Li GQ, Guan Y, Sun YY, et al. Clinical correlation analysis of risk factors affecting the quality of bowel preparation[J]. Medical Frontier, 2017, 7(29): 67-69. doi: 10.3969/j.issn.2095-1752.2017.29.059. | Analysis of risk factors only |
| 3 | Chen HD, Cui YQ, Wang B, et al. Analysis of influencing factors of bowel preparation in patients undergoing colonoscopy[J]. Medical Frontier, 2021, 11(25): 173-174. | Analysis of risk factors only |
| 4 | Chen PS, Ma ZJ, Zhang HS, et al. Influencing factors of poor bowel preparation before colonoscopy in elderly patients[J]. Modern Digestion & Intervention, 2017, 22(3): 358-360. doi: 10.3969/j.issn.1672-2159.2017.03.021. | Analysis of risk factors only |
| 5 | Li C, Guo HZ, Liu XJ, et al. Current status and risk analysis of poor bowel preparation for colonoscopy[J]. Chinese Medical Record, 2025, 26(8): 109-112. doi: 10.3969/j.issn.1672-2566.2025.08.032. | Analysis of risk factors only |
| 6 | Chen WP, Tang LY. Bowel preparation methods, influencing factors and nursing measures for elderly patients undergoing colonoscopy[J]. Chinese Journal of Science and Technology Database (Citation Edition) Medicine and Health, 2023(7). | Analysis of risk factors only |
| 7 | Chen YH, Huang MQ, Yang F. Analysis of influencing factors of bowel preparation quality before colonoscopy[J]. Chinese Journal of Pharmacoepidemiology, 2018, 27(8): 525-528. | Analysis of risk factors only |
| 8 | Liang R, Yang ZL, Yan XX, et al. Current status and influencing factors of bowel preparation before colonoscopy in elderly patients[J]. Chinese Nursing Management, 2019, 19(2): 182-187. doi: 10.3969/j.issn.1672-1756.2019.02.006. | Analysis of risk factors only |
| 9 | Dai XW, Zhang BM. Study on quality and influencing factors of bowel preparation before colonoscopy in inpatients[J]. Special Health, 2024(22): 126-127. | Analysis of risk factors only |
| 10 | Sun W, Wu W. Analysis of influencing factors of bowel preparation quality before colonoscopy in elderly outpatients[J]. Modern Medicine, 2021, 49(2): 218-222. doi: 10.3969/j.issn.1671-7562.2021.02.018. | Analysis of risk factors only |
| 11 | Qin ZJ. Discussion on bowel cleaning effect and influencing factors of colonoscopy[J]. Diet Science, 2022(7): 37-39. doi: 10.3969/j.issn.1008-9489.2022.07.013. | Analysis of risk factors only |
| 12 | Song J, Qu GY, Lin YY, et al. Meta-analysis of influencing factors for poor bowel preparation in elderly patients undergoing colonoscopy. Chinese Journal of Gerontology. 2020;40(3):548–552. doi: 10.3969/j.issn.1005-9202.2020.03.031. | Analysis of risk factors only |
| 13 | Ma X, Shi HY, Wan J, et al. Main factors affecting bowel preparation quality in hospitalized elderly patients undergoing colonoscopy diagnosis and treatment and analysis of reference thresholds. Medical Journal of the Chinese People’s Armed Police Forces. 2021;32(8):658–661. doi: 10.3969/j.issn.1004-3594.2021.08.004. | Analysis of risk factors only |
| 14 | Liang GD, Liu GX, Zhang TT, et al. Analysis of factors associated with bowel preparation quality in elderly patients. Practical Geriatrics. 2017;31(12):1148–1150. doi: 10.3969/j.issn.1003-9198.2017.12.015. | Analysis of risk factors only |
| 15 | Qin JS, Shi Y. Factors influencing bowel preparation quality in 313 hospitalized elderly patients. Guizhou Medical Journal. 2023;47(2):208–209. doi: 10.3969/j.issn.1000-744X.2023.02.022. | Analysis of risk factors only |
| 16 | Wang ZQ. Factors influencing bowel preparation quality before colonoscopy in patients with chronic constipation and nursing countermeasures. Chinese Journal of Coloproctology. 2023;43(5):58–60. doi: 10.3969/j.issn.1000-1174.2023.05.020. | Analysis of risk factors only |
| 17 | Han XY, Huang S, Xu MM, et al. Investigation and analysis of bowel cleansing effect and influencing factors before colonoscopy. Today Nurse. 2018;25(9):45–48. | Analysis of risk factors only |
| 18 | Ji L, Bai JJ, Gu YM, et al. Investigation and analysis of bowel cleansing effect and influencing factors before colonoscopy. Journal of Nursing Science. 2015;30(24):33–35. doi: 10.3870/j.issn.1001-4152.2015.24.033. | Absence of definitions of inadequate bowel preparation |
| 19 | He DD, He LZ, Liu R, et al. Analysis of factors associated with bowel cleanliness before electronic colonoscopy. Journal of Practical Clinical Nursing Science. 2017;2(4):11–13. | Absence of definitions of inadequate bowel preparation |
| 20 | He XD. Analysis of factors influencing bowel preparation quality before rectoscopy in elderly patients. Doctor. 2025;10(7):111–113. doi: 10.3969/j.issn.2096-2665.2025.07.037. | Analysis of risk factors only |
| 21 | He WT, Zhang LL, Lin YB, et al. Influencing factors and improvement measures for bowel preparation failure in elderly patients undergoing colonoscopy. Nursing of Integrated Traditional Chinese and Western Medicine. 2021;7(12):178–180. | Analysis of risk factors only |
| 22 | Huang HZ, Chen XY, Huo GL, et al. Influencing factors and countermeasures for bowel preparation quality before colonoscopy. International Medicine and Health Guidance News. 2011;17(10):1257–1259. doi: 10.3760/cma.j.issn.1007-1245.2011.10.038. | Analysis of risk factors only |
| 23 | Li P, Jin XW, Li YL, et al. Evidence summary for the prevention and management of inadequate bowel preparation in adult patients undergoing colonoscopy. Journal of Nursing. 2021;28(9):22–28. doi: 10.16460/j.issn1008-9969.2021.09.022. | Analysis of risk factors only |
| 24 | Wang YB, Zhang CQ, Zhang KL, et al. Analysis of factors influencing bowel preparation quality related to colonoscopy diagnosis and treatment in elderly patients. Journal of Third Military Medical University. 2017;39(18):1871–1874. doi: 10.16016/j.1000-5404.201703053. | Analysis of risk factors only |
| 25 | Li XY, Jia CL. Analysis of risk factors for poor bowel preparation in elderly inpatients undergoing colonoscopy. Chinese Journal of Coloproctology. 2024;44(5):52–54. doi: 10.3969/j.issn.1000-1174.2024.05.013. | Analysis of risk factors only |
| 26 | Li XH. Influencing factors and nursing intervention for bowel preparation before colonoscopy in patients from Tibetan areas. China Health Care & Nutrition. 2016;26(12):276. | Analysis of risk factors only |
| 27 | Sun C, He WY, Wang C, et al. Influencing factors of bowel preparation quality in hospitalized patients undergoing colonoscopy diagnosis and treatment. Chinese General Practice. 2019;22(32):3932–3938. doi: 10.12114/j.issn.1007-9572.2019.00.206. | Analysis of risk factors only |
| 28 | Liu MX. Related factors influencing bowel preparation quality and intervention measures. Chinese Journal of Modern Nursing. 2007;(20). | Analysis of risk factors only |
| 29 | Xun LJ, Xi MN, Song RM, et al. Influencing factors and nursing countermeasures for bowel preparation quality before colonoscopy in elderly patients. Journal of Clinical and Pathological Research. 2021;41(4):860–865. doi: 10.3978/j.issn.2095-6959.2021.04.020. | Analysis of risk factors only |
| 30 | Chen HS, Tao YX, Cen C. Research progress on bowel preparation regimens before colonoscopy. Medical Innovation of China. 2023;20(3):152–156. doi: 10.3969/j.issn.1674-4985.2023.03.037. | Analysis of risk factors only |
| 31 | Yang X, Zhao DM, Yang YQ, et al. Investigation of bowel preparation status and influencing factors before colonoscopy in elderly patients. Nursing Practice and Research. 2020;17(23):15–17. doi: 10.3969/j.issn.1672-9676.2020.23.005. | Analysis of risk factors only |
| 32 | Zhang ZM. Bowel preparation status and influencing factors in elderly patients undergoing colonoscopy. Nursing Practice and Research. 2020;17(22):1–4. doi: 10.3969/j.issn.1672-9676.2020.22.001. | Analysis of risk factors only |
| 33 | Zhang XW, Liu J. Analysis of the current status and influencing factors of bowel preparation quality before colonoscopy in elderly patients. Medicine and Health. 2022;(5). | Analysis of risk factors only |
| 34 | Shen JQ, Fu YJ. Influencing factors and intervention measures for bowel preparation efficacy before painless colonoscopy in elderly patients. Medical Journal of West China. 2010;22(5):959–960. doi: 10.3969/j.issn.1672-3511.2010.05.076. | Analysis of risk factors only |
| 35 | Zhao N. Cause analysis and nursing countermeasures for ineffective bowel preparation before colonoscopy. World Latest Medicine Information. 2021;21(16):14–15. doi: 10.3969/j.issn.1671-3141.2021.16.007. | Absence of definitions of inadequate bowel preparation |
| 36 | Tao H, Feng HX, Li YY, et al. Current status and influencing factors of bowel preparation quality in hospitalized patients undergoing colonoscopy in a tertiary hospital in Nanjing. Modern Medical Journal. 2023;51(5):675–682. doi: 10.3969/j.issn.1671-7562.2023.05.017. | Analysis of risk factors only |
| 37 | Yang RY, He JP, Yuan SQ. Analysis of related factors for poor bowel preparation before colonoscopy in children and adolescents. China Journal of Endoscopy. 2019;25(5):32–36. doi: 10.3969/j.issn.1007-1989.2019.05.007. | Analysis of risk factors only |
| 38 | Wang J. Investigation and analysis of bowel cleansing efficacy and influencing factors before colonoscopy. Today Nurse. 2018;25(9):34–36. doi: 10.3969/j.issn.1006-6411.2018.09.014. | Analysis of risk factors only |
| 39 | Chen YH, Li T, Zhu TT. Application of tramadol combined with atropine in colonoscopy. Chinese Journal of Rural Medicine and Pharmacy. 2017;24(5):64. doi: 10.3969/j.issn.1006-5180.2017.05.041. | Analysis of risk factors only |
| 40 | Zhu YZ, Chen TT. Application effect of rhubarb acupoint application in bowel preparation for patients with type 2 diabetes. Zhejiang Journal of Integrated Traditional Chinese and Western Medicine. 2025;35(9):828–830. doi: 10.3969/j.issn.1005-4561.2025.09.010. | Analysis of risk factors only |
| 41 | Wang TC, He ZX, Fan XM, et al. Research status and progress of risk prediction models for bowel preparation failure before colonoscopy. Chinese Journal of Digestive Endoscopy. 2024;41(9):741–745. doi: 10.3760/cma.j.cn321463-20231118-00178. | Analysis of risk factors only |
| 42 | Xu DL, Zhang T, Liang XY, et al. Multivariate analysis of factors affecting bowel cleanliness rate during colonoscopy and effects of targeted nursing. Heilongjiang Medicine. 2022;46(15):1898–1900. doi: 10.3969/j.issn.1004-5775.2022.15.031. | Analysis of risk factors only |
| 43 | Wen JZ. Analysis of factors influencing bowel preparation quality before colonoscopy in elderly patients. Chinese Journal of Geriatric Care. 2021;19(1):84–87. doi: 10.3969/j.issn.1672-2671.2021.01.028. | Analysis of risk factors only |
| 44 | Xu L, Dai DP. Current status and risk factors of bowel preparation failure in patients undergoing colonoscopy. Chinese Journal of Coloproctology. 2024;44(1):11–14. doi: 10.3969/j.issn.1000-1174.2024.01.004. | Analysis of risk factors only |
| 45 | Zhan KK. Analysis of bowel cleansing efficacy and influencing factors before colonoscopy. Chinese Journal of Coloproctology. 2020;40(11):55–57. doi: 10.3969/j.issn.1000-1174.2020.11.023. | Analysis of risk factors only |
| 46 | Zhou M. Investigation of bowel preparation status and influencing factors in outpatients undergoing colonoscopy. Chinese Journal of Coloproctology. 2019;39(11):50–52. doi: 10.3969/j.issn.1000-1174.2019.11.023. | Analysis of risk factors only |
| 47 | Xia MJ. Analysis of factors influencing bowel preparation quality before colonoscopy in health examination populations. Medicine and Health. 2023;(11). | Analysis of risk factors only |
| 48 | Yang WB, Li H, He LP. Effect of waiting time for colonoscopy on bowel preparation quality. Chinese Journal of Nursing. 2020;55(10):1452–1457. doi: 10.3761/j.issn.0254-1769.2020.10.002. | Analysis of risk factors only |
| 49 | Xu MH, Zhao B, Ma JJ, et al. Current status and influencing factors of bowel preparation in outpatients undergoing colonoscopy. Chinese Journal of Nursing. 2017;52(12):1473–1477. doi: 10.3761/j.issn.0254-1769.2017.12.013. | Analysis of risk factors only |
| 50 | Xun LJ, Wu XX, Song RM, et al. Effect of waiting time before colonoscopy on bowel preparation quality in hospitalized elderly patients. Chinese Journal of Practical Nursing. 2022;38(18):1375–1382. doi: 10.3760/cma.j.cn211501-20220228-00580. | Analysis of risk factors only |
| 51 | Yang H. Influencing factors and nursing intervention for bowel preparation before colonoscopy in elderly patients. Seek Medical and Ask the Medicine. 2012;10(3):326. | Analysis of risk factors only |
| 52 | Xu SL, Tang T, Chen J, et al. Current status and influencing factors of bowel cleansing efficacy in patients undergoing colonoscopy. Chinese Journal of Practical Nursing. 2019;35(29):2256–2262. doi: 10.3760/cma.j.issn.1672-7088.2019.29.004. | Analysis of risk factors only |
| 53 | Zhang YY, Niu ME, Wang QY, et al. Current status and influencing factors of bowel preparation efficacy before colonoscopy. Chinese Journal of Practical Nursing. 2017;33(14):1085–1088. doi: 10.3760/cma.j.issn.1672-7088.2017.14.011. | Analysis of risk factors only |
| 54 | Xu SH, Sui XY, Wan M, et al. Preliminary study on the effect of medication interval on bowel preparation quality in split-dose bowel preparation regimens for colonoscopy. Chinese Journal of Digestive Endoscopy. 2025;42(4):288–293. doi: 10.3760/cma.j.cn321463-20231127-00436. | Analysis of risk factors only |
| 55 | Yang YZ, Lu Q, Wang F, et al. Analysis of factors influencing bowel preparation quality before colonoscopy in health examination populations. Nursing Practice and Research. 2022;19(14):2074–2078. doi: 10.3969/j.issn.1672-9676.2022.14.005. | Analysis of risk factors only |
| 56 | Wu Y, Li Y, Xiao JT, et al. Visualization assessment of bowel preparation adequacy for colonoscopy based on a nomogram. Chinese Journal of Digestive Endoscopy. 2023;40(4):281–287. doi: 10.3760/cma.j.cn321463-20220922-00248. | Analysis of risk factors only |
| 57 | Yuan Yuan, Wang Yapei, Ji Huicong. Analysis of influencing factors of qualified bowel preparation in outpatients undergoing colonoscopy[J]. Chinese Journal of Modern Nursing,2022,28(34):4793-4796.doi:10.3760/cma.j.cn115682-20220504-02149. | Analysis of risk factors only |
| 58 | Yue Qian. Investigation of the current status and influencing factors of bowel preparation quality in outpatients[J]. Chinese Science and Technology Journal Database Medicine,2025(1):163-167. | Analysis of risk factors only |
| 59 | Zhang Heju, Zheng Xiaomeng, Zhang Mei, et al. Analysis of influencing factors of bowel preparation quality in adults undergoing colonoscopy[J]. Xinjiang Medical Journal,2025,55(3):255-260,269. | Analysis of risk factors only |
| 60 | Zhang Hongmao, Peng Shulei, Yan Huan. Current status of bowel preparation before colonoscopy in elderly patients and analysis of influencing factors[J]. Chinese Science and Technology Journal Database Medicine,2020(4):82. | Analysis of risk factors only |
| 61 | Zhang Sijia, Ma Gairong, Wang Jingli, et al. Current status and influencing factors of bowel preparation in outpatients undergoing colonoscopy[J]. Shanghai Nursing,2022,22(12):45-48.doi:10.3969/j.issn.1009-8399.2022.12.009. | Analysis of risk factors only |
| 62 | Qian Chunge. Current status of bowel preparation quality in patients undergoing colonoscopy and its correlation with health literacy[D]. Zhengzhou University,2022. | Analysis of risk factors only |
| 63 | Zhang Yinfang, Chen Jing. Analysis of influencing factors of bowel preparation quality before colonoscopy[J]. Modern Scientific Instruments,2021,38(5):177-181. | Analysis of risk factors only |
| 64 | Zou Mingzhu. Analysis of factors affecting bowel preparation before colonoscopy in elderly people[J]. Diet Health,2021(8):51. | Analysis of risk factors only |
| 65 | Xu Guizhi. Latent profile analysis and influencing factors of bowel preparation quality before colonoscopy in elderly patients[D]. Guangxi Medical University,2025.doi:10.27038/d.cnki.ggxyu.2025.001182. | Analysis of risk factors only |
| 66 | Zhang Shue. Analysis of influencing factors of bowel preparation quality before colonoscopy[D]. Southern Medical University,2023. | Analysis of risk factors only |
| 67 | Zou Lisha, Li Yan, Pan Jian. Investigation of bowel cleansing efficacy and influencing factors before colonoscopy[J]. Today Nurse,2022,29(3):10-12.doi:10.19793/j.cnki.1006-6411.2022.03.003. | Analysis of risk factors only |
| 68 | Sun Donghui. Analysis of influencing factors of inadequate bowel preparation before colonoscopy[D]. Gansu University of Chinese Medicine,2018. | Analysis of risk factors only |
| 69 | He Hao, Zhang Zhe, Yue Lin, et al. Risk factors for bowel preparation failure before colonoscopy in elderly patients and optimization of nursing strategies[J]. Journal of Nurses Training,2024,39(17):1848-1852.doi:10.16821/j.cnki.hsjx.2024.17.010. | Analysis of risk factors only |
| 70 | Xu Menghui. Study on current status and influencing factors of bowel preparation in outpatients undergoing colonoscopy[D]. Hebei Medical University,2018.doi:10.7666/d.D01514855. | Analysis of risk factors only |
| 71 | Ye Lingling, Feng Qing, Li Dan. Analysis of related factors affecting bowel cleansing efficacy in patients undergoing colonoscopy in Hainan[C]. International Digital Medicine Association. Proceedings of the 2017 International Digital Medicine Association Digital Traditional Chinese Medicine Branch,2017:773. | Analysis of risk factors only |
| 72 | Zhan Qiang. Study on influencing factors of colonoscopy quality and individualized intervention and evaluation[D]. Southern Medical University,2015. | Analysis of risk factors only |
| 73 | Agrawal R, Majeed M, Attar BM, Flores E, Haque Z, Aqeel SB, et al. Predictors of poor bowel preparations and colonoscopy cancellations in inpatient colonoscopies, a single center retrospective study. Transl Gastroenterol Hepatol. 2022;7. doi: 10.21037/tgh.2020.02.13. | Analysis of risk factors only |
| 74 | Amitay EL, Niedermaier T, Gies A, Hoffmeister M, Brenner H. Risk factors of inadequate bowel preparation for screening colonoscopy. J Clin Med. 2021;10(12). doi: 10.3390/jcm10122740. | Analysis of risk factors only |
| 75 | Appannagari A, Mangla S, Liao C, Reddy KG, Kupfer SS. Risk factors for inadequate colonoscopy bowel preparations in African Americans and whites at an urban medical center. South Med J. 2014;107(4):220-224. doi: 10.1097/smj.0000000000000087. | Analysis of risk factors only |
| 76 | Bhanthumkomol P, Siramolpiwat S, Vilaichone RK. Incidence and predictors of inadequate bowel preparation before elective colonoscopy in Thai patients. Asian Pac J Cancer Prev. 2014;15(24):10763-10768. doi: 10.7314/apjcp.2014.15.24.10763. | Analysis of risk factors only |
| 77 | Borg BB, Gupta NK, Zuckerman GR, Banerjee B, Gyawali CP. Impact of obesity on bowel preparation for colonoscopy. Clin Gastroenterol Hepatol. 2009;7(6):670-675. doi: 10.1016/j.cgh.2009.02.014. | No. of predictors ＜ 2 |
| 78 | Brljak J, Iveković H, Bilić B, Kovačić I, Markoš P, Brkić T, et al. Predictors of poor bowel preparation for colonoscopy: a single-centre prospective study. Lijec Vjesn. 2015;137(5-6):156-162. | Analysis of risk factors only |
| 79 | Cheng RW, Chiu YC, Wu KL, Rayner CK, Tai WC, Hu ML, et al. Predictive factors for inadequate colon preparation before colonoscopy. Tech Coloproctol. 2015;19(2):111-115. doi: 10.1007/s10151-014-1259-0. | Analysis of risk factors only |
| 80 | Fuccio L, Frazzoni L, Spada C, Mussetto A, Fabbri C, Manno M, et al. Factors that affect adequacy of colon cleansing for colonoscopy in hospitalized patients. Clinical Gastroenterology and Hepatology. 2021;19(2):339-348.e337. doi: 10.1016/j.cgh.2020.02.055. | Analysis of risk factors only |
| 81 | Garber A, Sarvepalli S, Burke CA, Bhatt A, Ibrahim M, McMichael J, et al. Modifiable factors associated with quality of bowel preparation among hospitalized patients undergoing colonoscopy. Journal of Hospital Medicine. 2019;14(5):278-283. doi: 10.12788/jhm.3173. | Analysis of risk factors only |
| 82 | Gimeno-García AZ, González-Suárez B, Ganzo ZA, Fernández OA, Ramos L, Giordano A, et al. Predictive factors for inadequate bowel cleansing in colon capsule endoscopy. Gastroenterol Hepatol. 2022;45(8):605-613. doi: 10.1016/j.gastrohep.2022.01.003. | Analysis of risk factors only |
| 83 | Govani SM, Elliott EE, Menees SB, Judd SL, Saini SD, Anastassiades CP, et al. Predictors of suboptimal bowel preparation in asymptomatic patients undergoing average-risk screening colonoscopy. World J Gastrointest Endosc. 2016;8(17):616-622. doi: 10.4253/wjge.v8.i17.616. | Analysis of risk factors only |
| 84 | Hautefeuille G, Lapuelle J, Chaussade S, Ponchon T, Molard BR, Coulom P, et al. Factors related to bowel cleansing failure before colonoscopy: results of the PACOME study. United European Gastroenterol J. 2014;2(1):22-29. doi: 10.1177/2050640613518200. | Analysis of risk factors only |
| 85 | Jawa H, Mosli M, Alsamadani W, Saeed S, Alodaini R, Aljahdli E, et al. Predictors of inadequate bowel preparation for inpatient colonoscopy. Turk J Gastroenterol. 2017;28(6):460-464. doi: 10.5152/tjg.2017.17196. | Analysis of risk factors only |
| 86 | Jin T, Cheng HX, Hao JY, Li C. Risk factors for inadequate bowel preparation before colonoscopy: a retrospective cohort study. World J Gastrointest Surg. 2025;17(9):109540. doi: 10.4240/wjgs.v17.i9.109540. | Analysis of risk factors only |
| 87 | Kim HG, Jeon SR, Kim MY, Lee TH, Cho JH, Ko BM, et al. How to predict adequate bowel preparation before colonoscopy using conventional polyethylene glycol: prospective observational study based on survey. Dig Endosc. 2015;27(1):87-94. doi: 10.1111/den.12312. | Analysis of risk factors only |
| 88 | Mahmood S, Farooqui SM, Madhoun MF. Predictors of inadequate bowel preparation for colonoscopy: a systematic review and meta-analysis. Eur J Gastroenterol Hepatol. 2018;30(8):819-826. doi: 10.1097/meg.0000000000001175. | Analysis of risk factors only |
| 89 | Maida M, Facciorusso A, Sinagra E, Morreale G, Sferrazza S, Scalisi G, et al. Predictive factors of adequate bowel cleansing for colonoscopy in the elderly: a retrospective analysis of a prospective cohort. Diagnostics. 2022;12(11). doi: 10.3390/diagnostics12112867. | Analysis of risk factors only |
| 90 | Ness RM, Manam R, Hoen H, Chalasani N. Predictors of inadequate bowel preparation for colonoscopy. Am J Gastroenterol. 2001;96(6):1797-1802. doi: 10.1111/j.1572-0241.2001.03874.x. | Analysis of risk factors only |
| 91 | Nguyen DL, Wieland M. Risk factors predictive of poor quality preparation during average risk colonoscopy screening: the importance of health literacy. J Gastrointestin Liver Dis. 2010;19(4):369-372. | Analysis of risk factors only |
| 92 | Occhipinti V, Soriani P, Vavassori S, Annunziata ML, Bagolini F, Cavallaro F, et al. Risk factors for inadequate bowel preparation in patients using high- and low-volume cleansing products. Eur J Gastroenterol Hepatol. 2023;35(2):159-166. doi: 10.1097/meg.0000000000002467. | Analysis of risk factors only |
| 93 | Okamoto N, Tanaka S, Abe H, Miyazaki H, Nakai T, Tsuda K, et al. Predicting inadequate bowel preparation when using sodium picosulfate plus magnesium citrate for colonoscopy: development and validation of a prediction score. Digestion. 2022;103(6):462-469. doi: 10.1159/000527312. | Analysis of risk factors only |
| 94 | Parra Pérez VF, Watanabe Yamamoto J, Nago Nago A, Astete Benavides M, Ulloa CR, Valladares Álvarez G, et al. Factors related to a suboptimal bowel preparation for colonoscopy. Acta Gastroenterol Latinoam. 2015;45(1):24-30. | Analysis of risk factors only |
| 95 | Poola S, Jampala N, Tumin D, Ali E. Factors influencing inpatient colonoscopy bowel preparation quality. Minerva Gastroenterol Dietol. 2020;66(3):194-200. doi: 10.23736/s1121-421x.20.02657-4. | Analysis of risk factors only |
| 96 | Qian CG. Current status of bowel preparation quality in patients undergoing colonoscopy and its correlation with health literacy[D]. Zhengzhou University, 2022. DOI: 10.27466/d.cnki.gzzdu.2022.000517. | No. of predictors ＜ 2 |
| 97 | Schmilovitz-Weiss H, Weiss A, Boaz M, Levin I, Chervinski A, Shemesh E. Predictors of failed colonoscopy in nonagenarians - a single-center experience. J Clin Gastroenterol. 2007;41(4):388-393. doi: 10.1097/01.mcg.0000225666.46050.78. | Analysis of risk factors only |
| 98 | Senol S, Kusak M, Uzunoglu Yildirim K, Gun M, Bidil MG. A prospective randomized study to predict bowel preparation quality prior to colonoscopy: comparison of two stool collection methods for the objective assessment of final rectal effluent clarity. Diagnostics (Basel). 2025;15(13). doi: 10.3390/diagnostics15131717. | Analysis of risk factors only |
| 99 | Serper M, Gawron AJ, Smith SG, Pandit AA, Dahlke AR, Bojarski EA, et al. Patient factors that affect quality of colonoscopy preparation. Clin Gastroenterol Hepatol. 2014;12(3):451-457. doi: 10.1016/j.cgh.2013.07.036. | Analysis of risk factors only |
| 100 | Shahini E, Sinagra E, Vitello A, Ranaldo R, Contaldo A, Facciorusso A, et al. Factors affecting the quality of bowel preparation for colonoscopy in hard-to-prepare patients: evidence from the literature. World J Gastroenterol. 2023;29(11):1685-1707. doi: 10.3748/wjg.v29.i11.1685. | Analysis of risk factors only |
| 101 | Shavrov AA, Koshurnikova AS, Starostina IE, Ibragimov SI, Shavrov AA. Risk factors for inadequate bowel preparation for colonoscopy and comparison of bowel preparation methods in pediatrics. Voprosy Prakticheskoi Pediatrii. 2023;18(2):76‐82. doi: 10.20953/1817-7646-2023-2-76-82. | Analysis of risk factors only |
| 102 | Shi L, Liao FQ, Liao WD, Zhu Y, Chen YX, Shu X. Risk factors for inadequate bowel preparation before colonoscopy: a retrospective cohort study. Bmc Gastroenterology. 2023;23(1). doi: 10.1186/s12876-023-02796-2. | Analysis of risk factors only |
| 103 | Yadlapati R, Johnston E, Gregory D, Ciolino J, Cooper A, Keswani R, et al. Predictors of inadequate inpatient colonoscopy preparation and its association with hospital length of stay and costs. Digestive Diseases & Sciences. 2015;60(11):3482-3490. doi: 10.1007/s10620-015-3761-2. | Analysis of risk factors only |
| 104 | Yee R, Manoharan S, Hall C, Hayashi A. Optimizing bowel preparation for colonoscopy: what are the predictors of an inadequate preparation? Am J Surg. 2015;209(5):787-792; discussion 792. doi: 10.1016/j.amjsurg.2014.12.018. | Analysis of risk factors only |
| 105 | Yong WC, Dong SH, Kwang HP, Kyoung OK, Cheol HP, Hahn T, et al. Patient factors predictive of inadequate bowel preparation using polyethylene glycol: a prospective study in Korea. J Clin Gastroenterol. 2009;43(5):448-452. doi: 10.1097/MCG.0b013e3181662442. | Analysis of risk factors only |
| 106 | Zhang YY, Niu M, Wu ZY, Wang XY, Zhao YY, Gu J. The incidence of and risk factors for inadequate bowel preparation in elderly patients: a prospective observational study. Saudi J Gastroenterol. 2018;24(2):87-92. doi: 10.4103/sjg.SJG_426_17. | Analysis of risk factors only |
| 107 | Zhao Yangyang. Analysis of factors influencing bowel preparation quality before colonoscopy[D]. North China University of Science and Technology,2021. | Irrelevant Topic |
| 108 | Sadeghi A, Rajabnia M, Bagheri M, Jamshidizadeh S, Saberi S, Shahnazi P, et al. Predictive factors of inadequate bowel preparation for elective colonoscopy. Gastroenterol Hepatol Bed Bench. 2022;15(1):66-78. doi: 10.22037/ghfbb.vi.2493. | Analysis of risk factors only |

**S8 Table 3.** Full-text articles included after eligibility assessment (n = 31)

| **No.** | **Reference** |
| --- | --- |
| 1 | Zhang N, Xu M, Chen X. Establishment of a risk prediction model for bowel preparation failure prior to colonoscopy. BMC Cancer. 2024;24(1):341. doi: 10.1186/s12885-024-12081-4. |
| 2 | Yin H, Wang Y, Wang H, Li T, Xu X, Li F, et al. Derivation and validation of a prediction model for inadequate bowel preparation in Chinese outpatients. Sci Rep. 2025;15(1):1430. doi: 10.1038/s41598-025-85816-3. |
| 3 | Li JM, Liu TW, Fu SY, Li Y, Lin YF, Zhang BP. Using the optimal subset method to establish a prediction model for bowel preparation. Chinese Journal of Practical Internal Medicine. 2020;40(03):231-236. doi: 10.19538/j.nk2020030111. |
| 4 | Huang MQ, Han DJ, Zhu HJ, Yang F. Analysis of predictive factors and development of model predicting inadequate intestinal preparation in outpatients undergoing colonoscopy by clinical pharmacists. Pharmaceutical and Clinical Research. 2023;31(2):172–176. doi: 10.13664/j.cnki.pcr.2023.02.007. |
| 5 | Hassan C, Fuccio L, Bruno M, Pagano N, Spada C, Carrara S, et al. A predictive model identifies patients most likely to have inadequate bowel preparation for colonoscopy. Clin Gastroenterol Hepatol. 2012;10(5):501-506. doi: 10.1016/j.cgh.2011.12.037. |
| 6 | Guo SL, Zhu T, Lin WN, Chen XR, Chen ZQ, Xia MY. Risk prediction model for inadequate bowel preparation before colonoscopy: a systematic review. Journal of Nursing (China). 2022;29(1):35–40. doi: 10.16460/j.issn1008-9969.2022.01.035. |
| 7 | Gu F, Xu J, Du L, Liang H, Zhu J, Lin L, et al. The machine learning model for predicting inadequate bowel preparation before colonoscopy: a multicenter prospective study. Clin Transl Gastroenterol. 2024;15(5):e00694. doi: 10.14309/ctg.0000000000000694. |
| 8 | Gkolfakis P, Kapizioni C, Tziatzios G, Facciorusso A, Frazzoni L, Thomopoulos K, et al. Comparative performance and external validation of three different scores in predicting inadequate bowel preparation among Greek inpatients undergoing colonoscopy. Ann Gastroenterol. 2023;36(1):25–31. doi: 10.1155/2019/5147208. |
| 9 | Gimeno-García AZ, Baute JL, Hernandez G, Morales D, Gonzalez-Pérez CD, Nicolás-Pérez D, et al. Risk factors for inadequate bowel preparation: a validated predictive score. Endoscopy. 2017;49(6):536–543. doi: 10.1055/s-0043-101683. |
| 10 | Dik VK, Moons LM, Hüyük M, van der Schaar P, de Vos Tot Nederveen Cappel WH, Ter Borg PC, et al. Predicting inadequate bowel preparation for colonoscopy in participants receiving split-dose bowel preparation: development and validation of a prediction score. Gastrointest Endosc. 2015;81(3):665-672. doi: 10.1016/j.gie.2014.09.066. |
| 11 | Berger A, Cesbron-Métivier E, Bertrais S, Olivier A, Becq A, Boursier J, et al. A predictive score of inadequate bowel preparation based on a self-administered questionnaire: PREPA-CO. Clin Res Hepatol Gastroenterol. 2021;45(4):101693. doi: 10.1016/j.clinre.2021.101693. |
| 12 | Apaer Z, Tian X, Wei R, Feng G, Ling HX. External Validation of a Prediction Model of Inadequate Bowel Preparation in Xinjiang. 2024. doi: 10.3969/j.issn.1006-5709.2024.04.012. |
| 13 | Zhao YY, Li ZQ, Ma X, Gao F. Establishment and validation of nomograms prediction model for bowel preparation quality in inpatients. Modern Interventional Diagnosis and Treatment in Gastroenterology. 2022;27(9):1100–1105. doi: 10.3969/j.issn.1672-2159.2022.09.006. |
| 14 | Zhao XX, Pan YL, Hao JY, Feng J, Cui ZY, Ma HM, et al. Development and validation of a novel scoring system based on a nomogram for predicting inadequate bowel preparation. Clin Transl Oncol. 2024;26(9):2262–2273. doi: 10.1007/s12094-024-03443-2. |
| 15 | Yuan X, Gao H, Liu C, Wang W, Xie J, Zhang Z, et al. External validation of two prediction models for adequate bowel preparation in Asia: a prospective study. Int J Colorectal Dis. 2022;37(6):1223–1229. doi: 10.1007/s00384-022-04156-0. |
| 16 | Xu MM, Fu XR, Zhang N, Wang Y, Shi FZ. Development and validation of a risk score model for inadequate bowel preparation for colonoscopy in elderly patients. Chinese Journal of Nursing. 2022;57(11):1337–1344. doi: 10.3761/j.issn.0254-1769.2022.11.009. |
| 17 | Wu H, Wu R, Zhang Y, Lu X, Zhao W, Xu B, et al. Development and validation of a gut motility based model for predicting bowel preparation quality. Sci Rep. 2025;15(1):28265. doi: 10.1038/s41598-025-13739-0. |
| 18 | Wang JX, Suo LN, Yu FF. Factors influencing quality of bowel preparation before colonoscopy in elderly patients and construction and validation of a risk prediction model for bowel preparation failure. Chinese Journal of Coloproctology. 2024;44(11):51–54. doi: 10.3969/j.issn.1000-1174.2024.11.014. |
| 19 | Wang GH, Chen J, Sheng ZJ, Xi MJ, Zhou YT. Establishing and evaluating a risk prediction model for colonoscopy bowel preparation failure based on automated machine learning. China Journal of Endoscopy. 2024;30(05):36–47. doi: 10.12235/E20230422. |
| 20 | Song ZH, Zhang PK, Gao XZ. Development and validation of a risk nomogram for inadequate bowel preparation. Chinese Journal of Gasttrointestinal Endoscopy(Electronic Edition). 2024;11(2):100–104. doi: 10.3877/cma.j.issn.2095-7157.2024.02.006. |
| 21 | Sninsky JA, Toups JV, Cotton CC, Peery AF, Arora S. An electronic medical record prediction model to identify inadequate bowel preparation in patients at outpatient colonoscopy. Tech Innov Gastrointest Endosc. 2024;26(2):130–137. doi: 10.1016/j.tige.2023.12.008. |
| 22 | Rao W, Peng SY, Xu T, Re ZY, Huang XL. Analysis of predictive factors and model establishment of inadequate bowel preparation. Modern Digestion & Intervention. 2021;26(11):1378–1383. doi: 10.3969/j.issn.1672-2159.2021.11.009. |
| 23 | Okamoto N, Tanaka S, Abe H, Miyazaki H, Nakai T, Tsuda K, et al. Predicting inadequate bowel preparation when using sodium picosulfate plus magnesium citrate for colonoscopy: development and validation of a prediction score. Digestion. 2022;103(6):462–469. doi: 10.1159/000527312. |
| 24 | Malkin D, Cohen DL, Richter V, Ariam E, Vosko S, Shirin H, et al. A novel model to predict inadequate bowel preparation prior to colonoscopy incorporating patients' reactions to drinking the laxative. J Clin Med. 2023;12(23). doi: 10.3390/jcm12237335. |
| 25 | Liu Y, Liu XQ, Yang XN, Wang P, Liu XK, Luo D. Construction and validation of a risk prediction model for bowel preparation failure in colonoscopy patients. Chinese Journal of Nursing. 2024;59(9):1091–1098. doi: 10.3761/j.issn.0254-1769.2024.09.010. |
| 26 | Liu J, Jiang W, Yu Y, Gong J, Chen G, Yang Y, et al. Applying machine learning to predict bowel preparation adequacy in elderly patients for colonoscopy: development and validation of a web-based prediction tool. Ann Med. 2025;57(1):2474172. doi: 10.1080/07853890.2025.2474172. |
| 27 | Guo SL, Zhu T, Lin WN, Chen XR, Chen ZQ, Xia MY, et al. Establishment and validation of risk predictive model for inadequate bowel preparation before colonoscopy in the elderly. Chinese nursing research. 2023;37(3):392–398. doi: 10.12102/j.issn.1009-6493.2023.03.004. |
| 28 | Fuccio L, Frazzoni L, Spada C, Mussetto A, Fabbri C, Manno M, et al. Factors that affect adequacy of colon cleansing for colonoscopy in hospitalized patients. Clin Gastroenterol Hepatol. 2021;19(2):339–348.e337. doi: 10.1016/j.cgh.2020.02.055. |
| 29 | Fang L, Bingbing W, Wang Q, Yinchuan X. Development and validation of a nomogram predictive model and scoring tool for assessing the risk of inadequate bowel preparation in colonoscopy patients over 40 years old: a retrospective observational study. J Clin Nurs. 2025;34(10):4398–4414. doi: 10.1111/jocn.17702. |
| 30 | Chen L, Ren G, Luo H, Zhang L, Wang L, Zhao J, et al. Superiority of a preparation-related model for predicting inadequate bowel preparation in patients undergoing colonoscopy: a multicenter prospective study. J Gastroenterol Hepatol. 2022;37(12):2297–2305. doi: 10.1111/jgh.16010. |
| 31 | Afecto E, Ponte A, Fernandes S, Gomes C, Correia JP, Carvalho J. Validation and application of predictive models for inadequate bowel preparation in colonoscopies in a tertiary hospital population. GE Port J Gastroenterol. 2023;30(2):134–140. doi: 10.1159/000520905. |
